# Site-specific His/Asp phosphoproteomic analysis of prokaryotes reveals putative targets for drug resistance

**DOI:** 10.1186/s12866-017-1034-2

**Published:** 2017-05-25

**Authors:** Shu-Jung Lai, I-Fan Tu, Wan-Ling Wu, Jhih-Tian Yang, Louis Y. P. Luk, Mei-Chin Lai, Yu-Hsuan Tsai, Shih-Hsiung Wu

**Affiliations:** 1 Institute of Biological Chemistry, Academia Sinica, Taipei, Taiwan; 2 PhD Program in Microbial Genomics, National Chung Hsing University and Academia Sinica, Taichung, Taiwan; 3 School of Chemistry, Cardiff University, Cardiff, UK; 4 Department of Life Sciences, National Chung Hsing University, Taichung, Taiwan; 5 Chemical Biology and Molecular Biophysics Program, Taiwan International Graduate Program, Academia Sinica, Taipei, Taiwan; 6 Department of Chemistry, National Taiwan University, Taipei, Taiwan

**Keywords:** Proteomics, Post-translational modification, Histidine phosphorylation, Aspartate phosphorylation, Pathogenic bacteria, Drug resistance

## Abstract

**Background:**

Phosphorylation of amino acid residues on proteins is an important and common post-translational modification in both eukaryotes and prokaryotes. Most research work has been focused on phosphorylation of serine, threonine or tyrosine residues, whereas phosphorylation of other amino acids are significantly less clear due to the controversy on their stability under standard bioanalytical conditions.

**Results:**

Here we applied a shotgun strategy to analyze the histidine and aspartate phosphorylations in different microbes. Our results collectively indicate that histidine and aspartate phosphorylations frequently occur also in proteins that are not part of the two-component systems. Noticeably, a number of the modified proteins are pathogenesis-related or essential for survival in host. These include the zinc ion periplasmic transporter ZnuA in *Acinetobacter baumannii* SK17, the multidrug and toxic compound extrusion (MATE) channel YeeO in *Klebsiella pneumoniae* NTUH-K2044, branched amino acid transporter AzlC in *Vibrio vulnificus* and the RNA-modifying pseudouridine synthase in *Helicobacter pylori*.

**Conclusions:**

In summary, histidine and aspartate phosphorylation is likely to be ubiquitous and to take place in proteins of various functions. This work also sheds light into how these functionally important proteins and potential drug targets might be regulated at a post-translational level.

**Electronic supplementary material:**

The online version of this article (doi:10.1186/s12866-017-1034-2) contains supplementary material, which is available to authorized users.

## Background

Protein phosphorylation is a ubiquitous chemical event found in both eukaryotes and prokaryotes. Regulated by kinases and phosphatases, this type of post-translational modification is highly dynamic, changes protein function in the due course of the cell cycle and plays critical roles in signal transduction. Due to its associations with many human disorders, the molecular networks of mammalian protein phosphorylation have been extensively investigated [1]. On the other hand, protein phosphorylation also plays critical roles in bacterial pathogenesis [2–4]. Since there are numerous unmet clinical needs caused by bacterial infections, it is of fundamental importance to fully expand the research of bacterial phosphorproteomics which likely holds valuable information for future drug development.

In bacteria, various amino acid residues within a protein, including serine, threonine, tyrosine, histidine and aspartate, can be modified with a phosphate group [5, 6]. A plethora of data related to serine, threonine and tyrosine phosphorylations have already been reported. They are shown to be involved in changing metabolic behaviors, inducing capsule formation and initiating sporulation [5]. On the other hand, the knowledge of histidine and aspartate phosphorylations are mostly limited to the two-component systems [7], in which phosphor-relaying signaling events were induced by external environmental stimuli, including changes in pH, osmolality and oxygen, all of which are essential for the bacterial pathogenesis in human. Nevertheless, little is known about any histidine and aspartate phosphorylation events outside of the two-component systems [8].

Protein modification status is often investigated by gel electrophoresis coupled with liquid chromatography (LC) and high-resolution mass spectrometry analysis [9]. As useful biochemical information can be revealed only when the covalently modified residues are isolated and characterized experimentally, the proteomic data available for each type of phosphorylated residues is directly proportional to its chemical stability. Among various types of protein phosphorylations, the stability of acid-labile phosphorylations under standard mass spectrometric conditions remains somewhat controversial. Jensen and co-workers investigated the stability of a peptide containing a phosphorylated histidine and concluded that a fast LC method or non-acidic solvent system is needed for well-resolved electrospray ionization mass spectrometry analysis [10]. In contrast, a study by Hohenester et al. showed that typical proteomic methods that use acidic solvent systems and collision-induced dissociation for fragmentation can be applied [11]. These differences may originate from the fact that peptides with different sequences were used [10, 11]. On the other hand, the analysis of peptides containing a phosphorylated aspartate has not been conducted. In essence, the possibility to study acid-labile phosphorylation by standard mass spectrometry methods has not been ruled out.

Here, we aim to expand the horizon of bacterial phosphoproteomic research and present mass spectrometric analysis of histidine and aspartate phosphorylations in different key prokaryotes at the exponential phase, including pathogenic bacteria (*Acinetobacter baumannii* SK17, *Helicobacter pylori*, *Klebsiella pneumoniae* NTUH-K2044, *Vibrio vulnificus*), cyanobacterium (*Arthrospira platensis* C1), thermophilic bacteria (*Meiothermus taiwanensis* WR220, *Thermus thermophilus* HB27) and methanogenic archaea (*Methanosarcina mazei* N2 M9705, *Methanohalophilus portucalensis* FDF1^T^) (Table 1). Our results showed that, under optimal growing conditions, numerous proteins beyond the two-component systems are phosphorylated at the aspartate and histidine residues. Notably, many proteins that contain aspartate and histidine phosphorylations are involved in cell survival and antibiotic resistance.

**Table 1:** Characterized unique pHis/pAsp sites and phosphopeptides in the nine organisms

	pHis	pAsp	p-peptides
*A. baumannii*	31	15	40
*H. pylori*	22	6	24
*K. pneumoniae*	7	0	7
*V. vulnificus*	30	18	40
*A. platensis*	12	2	14
*M. taiwanensis*	15	11	21
*T. thermophiles*	11	3	11
*M. mazei*	18	9	25
*M. portucalensis*	13	5	15
Total	159	69	197

## Methods

### Bacterial strains and growth conditions

The isolate of cyanobacterium, *A. platensis C1*, was a kind gift from Prof. Apiradee Hongsthong (National Center for Genetic Engineering and Biotechnology, Thailand). Axenic cultures of this bacterium were grown at 35 °C under illumination by 100 μE m^−2^ s^−1^ fluorescent light with continuous stirring in 1.5 L of Zarrouk’s medium [12]. The cultures were grown to the mid-exponential phase (OD_560 nm_ = 0.4).

As previously described [13–15], we cultured and analyzed the phosphoproteome of *A. baumannii* SK17 [15], *K. pneumoniae* NTUH-K2044 [14] and *T. thermophilus* HB27 [13], where were kind gifts from Dr. Te-Li Chen (Taipei Veterans General Hospital, Taiwan), Prof. Jin-Town Wang (National Taiwan University, Taiwan) and Prof. Guang-Huey Lin (Tzu-Chi University, Taiwan), respectively. *A. baumannii* SK17 and *K. pneumoniae* used in this study were originally isolated from human patients [16, 17].

*H. pylori* reference strain 26695 (ATCC 700392) was obtained from the Food Industry Research and Development Institute, Taiwan (BCRC 17219). Cells were grown at 37 °C under a standard microaerobic atmosphere (5% O_2_, 10% CO_2_, 85% N_2_) on Columbia agar base (CAB; Oxoid) containing 10% horse blood for 24 h.

*V. vulnificus* was obtained from the Food Industry Research and Development Institute, Taiwan (BCRC 12B0001). A single clone of this bacterium was grown in LB medium at 37 °C with vigorous shaking. The overnight culture was diluted to OD_600 nm_ = 0.01 into fresh LB medium contained 2% NaCl and 10 nM FeSO_4_. The culture was grown to the mid-exponential phase (OD_600 nm_ = 0.6).

*M. taiwanensis* WR220 was obtained from the Food Industry Research and Development Institute, Taiwan (BCRC 17171). The thermophilic bacterium was grown under aerobic conditions at 55 °C in TM medium [18] to the mid-exponential phase (OD_600 nm_ = 0.8).

For methanogenic archaea, *M. portucalensis* FDF1^T^ was purchased from the Leibniz Institute DSMZ–German Collection of Microorganisms and Cell Cultures (DSM No. 7471), and *M. mazei* N2 M9705 was originally isolated from an aquaculture fishpond near Wang-gong, Taiwan [19] and has been deposited with the Food Industry Research and Development Institute, Taiwan (BCRC 16179). *M. portucalensis* FDF1^T^ was cultured in defined medium containing 120 g L^−1^ NaCl and 20 mM trimethylamine as the sole carbon and energy source [20], whereas *M. mazei* N2 M9705 were cultured in MB medium containing 5 g L^−1^ NaCl and 20 mM of methanol. Sterile medium was prepared under 20% CO_2_ in N_2_ atmosphere by a modification of the Hungate technique [21]. The growth rates were monitored by removing 1 mL of the culture with a N_2_-flushed syringe into a Na_2_S_2_O_3_ containing cuvette [22]. The cultures were grown anaerobically at 37 °C to the mid-exponential phase (OD_540 nm_ = 0.5).

### Protein extraction

Cells at the mid-exponential phase were harvested by centrifugation at 6000 g for 15 min at 4 °C and washed twice with PBS. The resulting pellets were resuspended in freshly prepared lysis buffer containing 25 mM ammonium bicarbonate, phosphatase inhibitor (PhosSTOP, Roche), 6 M urea and 2 M thiourea. Cells were disrupted by sonication on ice. Cellular debris were removed by centrifugation at 12,000 g for 30 min at 4 °C. The supernatant was recovered, and the protein concentrations were determined by the Bradford assay (Bio-Rad).

### In-solution protein digestion

About 10 mg of total protein extracts was reduced with 10 mM dithiothreitol (DTT) at 37 °C for 1 h and was alkylated with 55 mM iodoacetamide at room temperature in the absence of light for 1 h. The sample was diluted to 1:3 (v:v) ratio with 25 mM ammonium bicarbonate buffer (pH 8.5) and then digested with TPCK trypsin (1:50 *w*/w) (Pierce) at 37 °C for overnight. The tryptic peptides were desalted using SDB-XC StageTip with SDB-XC Empore disc membranes (3 M) [23] and eluted in a buffer containing 0.1% trifluoroacetic acid (TFA) and 80% acetonitrile (ACN). The eluates were dried in a SpeedVac concentrator (Thermo Electron Corporation) and stored at −20 °C.

### In-gel protein digestion

About 2 mg of the protein extracts were resolved on a 12.5% SDS-PAGE gel (1.5-mm-thick) and stained with Coomassie Brilliant Blue G-250. The gel was sliced into eight parts, and the individual slices were further diced into small pieces (0.5–1 mm^3^) for in-gel digestion. Each piece was first washed with water and completely destained with 50% ACN in 25 mM ammonium bicarbonate buffer (pH 8.5). ACN was removed from gel slices followed by re-equilibration in 25 mM ammonium bicarbonate buffer (pH 8.5), reduced with 30 mM DTT at 37 °C for 1 h, and then alkylated with 60 mM idoacetamide at room temperature in the dark for 1 h. Before trypsin digestion was performed, 100% ACN covered the gel pieces until the gel pieces became white and shrunken, then dried in a vacuum centrifuge. The gel pieces were partially swollen with 25 mM ammonium bicarbonate buffer (pH 8.5). The TPCK trypsin (1:50 *w*/w) (Pierce) dissolved in the same solution was added to recuperate their original size. The digestion was carried out overnight at 37 °C and stopped by the extraction buffer containing 5% TFA and 50% ACN. The resulting peptides were recovered twice with the extraction buffer by 10 min incubation in the sonication bath. The extracts were dried in a vacuum centrifuge and stored at −20 °C.

### Phosphopeptide enrichment and MS analysis

Phosphopeptides from the digested peptides were enriched by custom-made HAMMOC tips, which were prepared using 0.5 mg TiO_2_ beads (GL Sciences) packed into 10-μL C8-StageTips, as described previously [23, 24]. The HAMMOC tips were washed with solution A (0.1% TFA, 80% ACN) followed by the equilibration of the solution A containing 300 mg/mL lactic acid as a selectivity enhancer (solution B). About 100 μg of the dry tryptic digest was re-dissolved in solution A and diluted with an equal volume of solution B before sample loading. After washing the bound phosphopeptides twice with solution B and solution A individually, 0.5 and 5% piperidine were used for elution. The other enrichment strategy was performed with pre-incubated TiO_2_ beads by loading buffer (1 m glycolic acid in 80% ACN and 5% TFA). Total peptide solution was then mixed and incubated with 2 mg TiO_2_ beads for 1 h at 4 °C. Nonspecific binding peptides were washed with loading buffer and wash buffer (80% ACN and 5% TFA), and the bound phosphopeptides were eluted with 1% NH_4_OH in 40% ACN, pH > 10.5. The eluate was acidified with 20% phosphoric acid to pH 2.5, and desalted using SDB-XC StageTip as described above. The resulting phosphopeptides were concentrated in a vacuum centrifuge before subsequent nanoLC-MS/MS analysis as previously described [13–15].

### MS/MS database searching and phosphorylation site analysis

All previously published (*A. baumannii* SK17, *K. pneumoniae* NTUH-K2044, *T. thermophilus* HB27) [13–15] and newly acquired MS and MS/MS raw data were analyzed using MaxQuant (version 1.5.1.2, http://www.coxdocs.org/doku.php?id=maxquant:start) [25] with the built-in search engine Andromeda [26] for phosphopeptide identification and phosphorylation site analysis. The protein sequences for MS/MS database search consisted of published genome databases from National Center for Biotechnology Information, including *A. baumannii* SK17, *A. platensis* C1, *H. pylori* 26695, *K. pneumoniae* NTUH-K2044, *M. mazei* Go1, *M. taiwanensis* DSM 14542, *T. thermophilus* HB27 and *V. vulnificus* YJ106, and an in-house draft genome sequence of *M. portucalensis* FDF1^T^ containing in total 2131 protein sequences constructed from a well annotated genome of *M. mahii* DSM 5219 [27], which shares 99.58% 16S rRNA sequence identity with *M. portucalensis* FDF1^T^. The protein-encoding genes from *M. portucalensis* FDF1^T^ genome sequence were predicted previously by Glimmer 2.13 [28], GeneMark 2.4, and GeneMark.hmm 2.1 [29] and annotated with the RefSeq Microbial Genomes database [30] using BLASTP in standard settings (*E*-value <10^−5^, identity >40%, and matched length > 30%).

The search criteria used for phosphopeptide and phosphosite analysis were as follows: trypsin digestion; cysteine carboxyamidomethylation (+ 57.0214 Da) as the fixed modification; methionine oxidation (+ 15.9949 Da), phosphorylation of amino acid (Ser, Thr, Tyr, His, Asp) residues, and protein N-terminal acetylation as variable modifications; up to two missed cleavage allowed; minimum seven amino acids per peptide; and mass accuracy of 10 ppm for the parent ion and 0.6 Da for the fragment ions. False discovery rate was estimated from the target-decoy strategy to distinguish correct and incorrect identification. For the identification, false discovery rate was set to 0.01 for sites, peptides and proteins. We calculated the localization probabilities of all phosphorylation sites using the PTM score algorithm as previously described [31]. The phosphorylation site of at least 0.75 probability of phosphorylation is defined as reliable. MS/MS spectra of peptides containing reliable phosphohistidine and phosphoaspartate were manually inspected to confirm unambiguous identification. Only the identified phosphoproteins matched to protein sequences of the corresponding organism are reported.

## Results

**Fig. 1:**
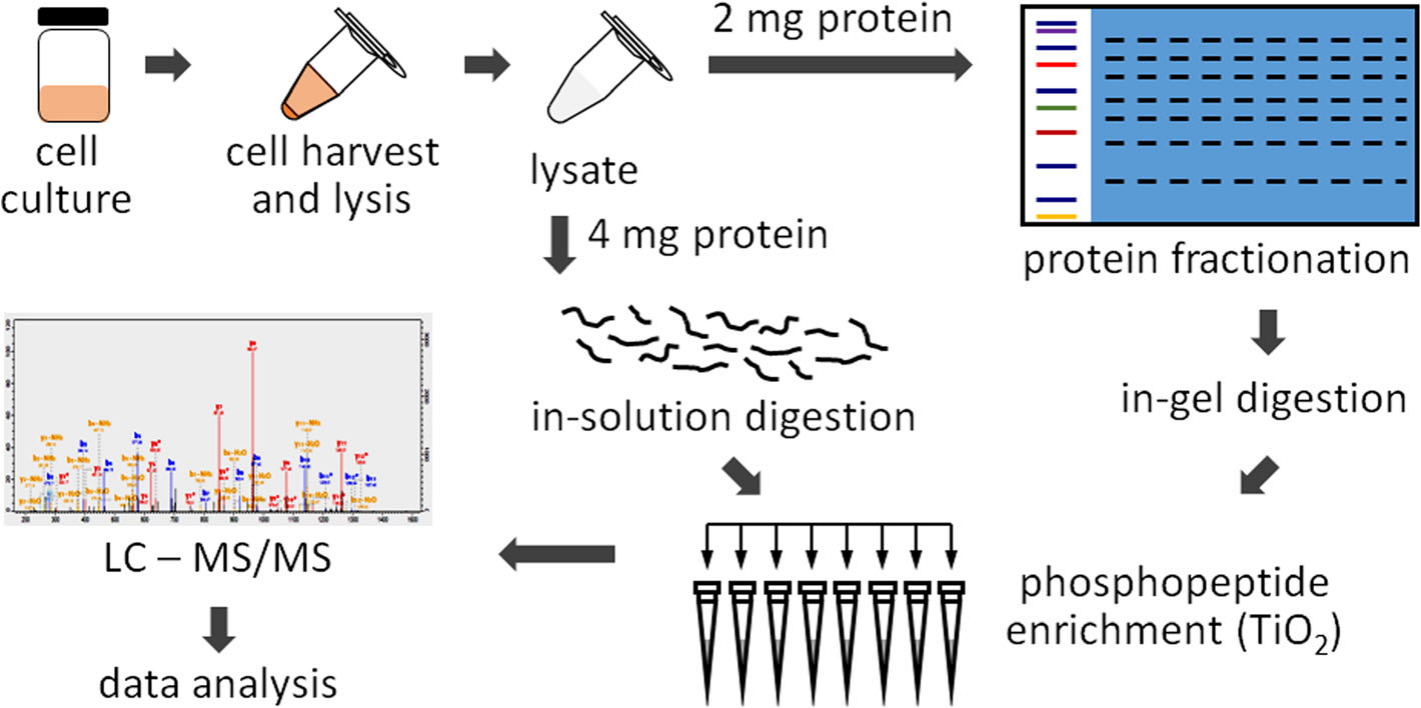
The workflow of phosphoproteomic analysis

We previously performed phosphoproteome analyses of various bacterial species [13–15], and showed that trypsin-digested phosphorylated peptides could be enriched by titanium dioxide (TiO_2_) chromatography [23, 24] followed by LC-MS/MS analysis (Fig. 1). We showed that Ser/Thr/Tyr phosphorylations can be clearly characterized, but phosphorylation of other amino acid residues remains unclear. Nevertheless, histidine and aspartate phosphorylations play critical roles in signaling transductions [7], and thus deciphering the proteome-wide extent of such modifications is of great interest.

In this work, we switched our focus and collected samples from nine different species harvested at the mid-exponential phase. These include the clinically isolated pathogens, *A. baumannii* SK17, *H. pylori* 26695, *K. pneumoniae* NTUH-K2044 and *V. vulnificus*, as well as naturally isolated prokaryotes, including cyanobacterium *A. platensis* C1, thermophiles *M. taiwanensis* WR220 and *T. thermophilus* HB27 and methanogenic archaea *M. mazei* N2 M9705 and *M. portucalensis* FDF1^T^.

**Fig. 2:**
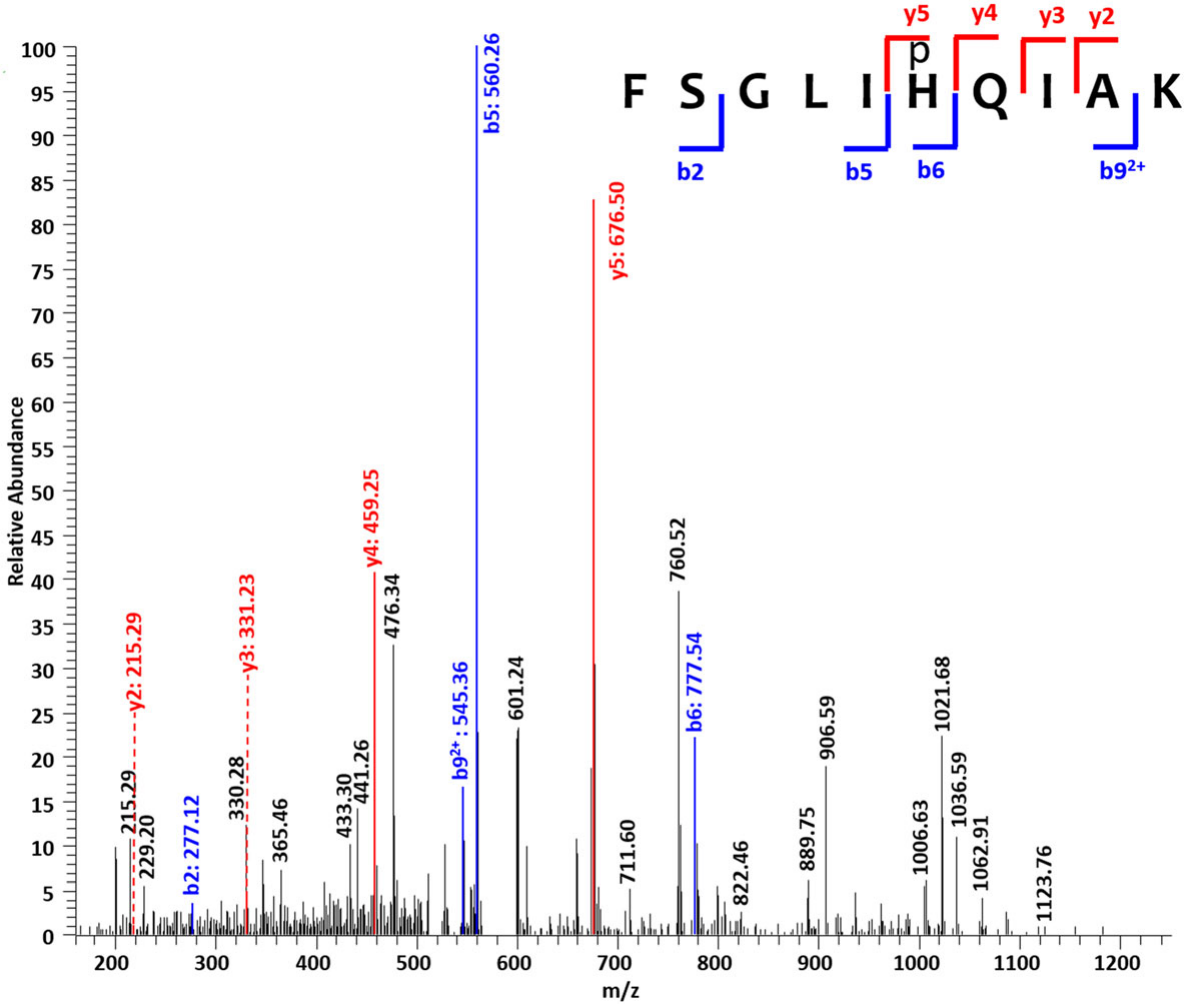
MS/MS spectra of a representative phosphohistidine peptide from *H. pylori*. The presence of y4-, y5, b5- and b6-ions enables pinpointing the phosphorylation at the indicated histidine residue. Spectra of other phosphopeptides can be found in Additional file 1

Upon in-solution and in-gel tryptic digestion, the samples were enriched and analyzed using the shotgun LC-MS/MS approach. The phosphopeptides derived from these samples were scrutinized with at least seven amino acids and a minimum five fragmented b-/y-ions using high-confidence identification (false discovery rate < 0.01, localization probability >0.75, score of phosphopeptides >40). To unambiguously identify phosphorylated histidine and aspartate residues, we also inspected the individual MS/MS spectra (Fig. 2 and Additional file 1) to assure the presence of critical b-/y-ions to support phosphorylation of only the designated histidine or aspartate residue but none of the other amino acids (i.e. Ser, Thr, Tyr, Arg, Lys, Glu, Cys) [5, 6]. For example, for peptide FSGLIpHQIAK (Fig. 2), phosphorylation of the histidine residue is unambiguously supported by the presence of y4-, y5, b5- and b6-ions. Phosphopeptides that lack critical b-/y-ions were discarded during manual inspection. Under these stringent conditions, we found that histidine and aspartate phosphorylations are common phenomena with nearly 200 post-translationally modified peptides identified. We noticed that the majority of the identified phosphopeptides was from in-solution trypsin digestion followed by HAMMOC tip enrichment (Additional file 2).

**Fig. 3:**
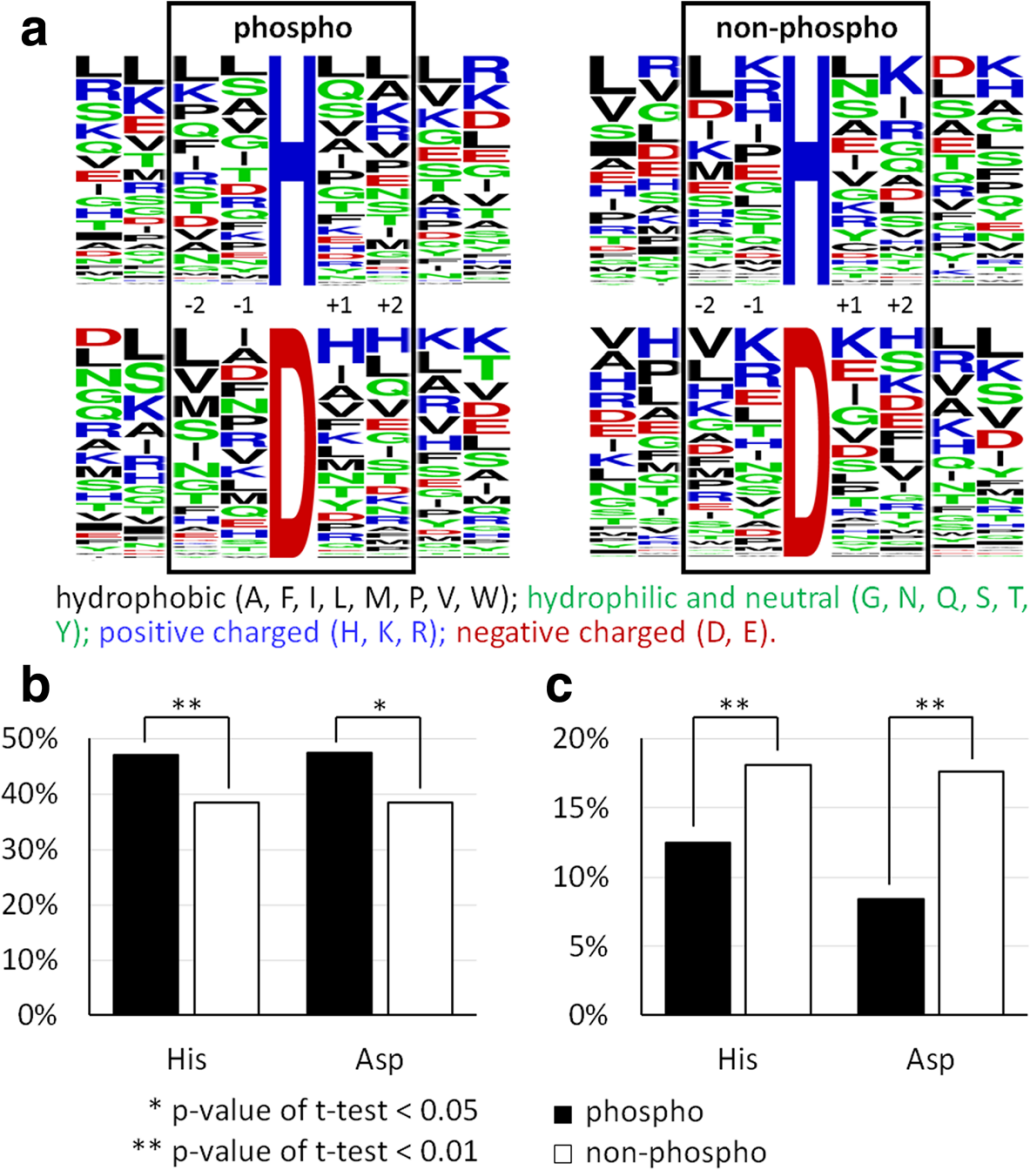
Sequence features of the phosphorylation sites. All His/Asp residues in the identified phosphoproteins were classified as either phosphorylated or non-phosphorylated for the analysis. **a** Relative abundances of amino acids between −4 and +4 positions of the phosphorylated or non-phosphorylated His/Asp. Sequence logos were generated using WebLogo (http://weblogo.berkeley.edu/). Alignment of sequences between −10 and +10 positions is shown in Additional file 3. **b** Probability of hydrophobic amino acids between −2 and +2 positions. **c** Probability of Arg and Lys between −2 and +2 positions

Relative abundances of amino acids flanking the phosphorylated or non-phosphorylated His and Asp residues identified in this study were compared (Fig. 3a and Additional file 3). In the immediate positions (−2, −1, +1, +2), the frequency of hydrophobic amino acid is higher around the phosphorylated versus non-phosphorylated sites (Fig. 3b). On the other hand, the probability of Arg and Lys residues is significantly higher around the non-phosphorylated than phosphorylated sites (Fig. 3c). Since trypsin cleaves exclusively at the C-terminal of Arg and Lys residues [32], sites in close proximity to Arg and Lys will end up close to peptide terminus upon trypsin digestion. Hence, our results indicated that phosphorylated sites are more distant from the peptide terminus. Consequently, we conclude that the identified phosphorylated His and Asp residues preferentially reside in an internal position of the peptide surrounded by hydrophobic amino acid residues.

**Fig. 4:**
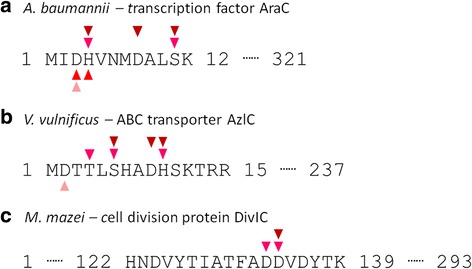
Peptides identified with different modification states. Unambiguously phosphorylation sties characterized by b- and y-ions are indicated. *Triangle* of the same color refers to phosphorylation identified from a single phosphopeptide. **a** AraC of * A. baumannii*. **b** AzlC of * V. vulnificus*. **c** DivlC of * M. mazei*

Interestingly, we also found peptides with different extents of phosphorylations (Fig. 4). Enrichment and subsequent identification of mono-phosphorylated peptides are generally more straightforward in comparison to multi-phosphorylated peptides [9]. Nevertheless, we found peptides with mono- and multi-phosphorylation sites, and some multi-phosphorylated peptides contain more than one phosphorylated histidine and/or aspartate residues (Additional file 2). Noticeably, we found a few peptides of the same amino acid sequence but with different phosphorylation states in several organisms (Fig. 4). The results further highlight the prevalence of protein phosphorylation in bacteria.

As shown in Table 2, the identified phosphoproteins fall in different functional classes. In all organisms, we identified proteins involved in metabolic pathways and cellular processes. We are particularly interested in proteins potentially involving in pathogenesis. Potential candidates include those participating in cellular processes, signal transduction, transport and binding, and transcription. We highlight twelve proteins here and classified our findings based on their functions (Table 3). AzlC in *V. vulnificus* [33], FecA in *H. pylori* [34], YeeO in *K. pneumoniae* [35] and ZnuA in *A. baumanni* [36] are important protein transporters linked to drug resistance. Other putative drug-resistance proteins in the pathogenic bacteria include transcription factor AraC [37], sulfate transport system permease CysW [38], twitching motility protein PilT [39], taurine-binding periplasmic protein TauA [40], inner membrane protein YfiN [41], mobile element protein and plasmid mobilization protein. Lastly, pseudouridine synthase in *H. pylori* is a key nucleic acid-modifying protein that contributes to RNA stability [42].

**Table 2:** Distribution of identified phosphoproteins in the nine organisms by molecular function

	*Ab*	*Hp*	*Kp*	*Vv*	*Ap*	*Mt*	*Tt*	*Mm*	*Mp*
Metabolism (amino acid/carbohydrate/others)	3/-/5	4/-/6	1/-/2	-/5/2	-/2/3	2/3/-	-/4/1	-/4/-	-/-/1
Cellular process/signal transduction/transport and binding	5/1/9	2/1/1	1/-/1	8/3/2	2/1/-	3/3/-	1/1/-	5/2/-	6/-/2
Transcription/translation/protein folding and modification	3/-/-	1/1/1	-/-/-	9/-/1	-/-/2	1/3/1	-/2/-	4/3/-	3/-/-
Unknown	1	6	2	8	4	5	1	4	3
Total number of unique proteins	27	23	7	38	14	21	10	22	15

**Table 3:** Identified phosphoproteins with putative link to drug resistance in pathogenic bacteria

Species	Protein	Functional class	pHis and pAsp sites
*Ab*	Periplasmic-binding zinc ABC transporter ZnuA	Transport/binding	His6
*Ab*	Transcription factor AraC	Transcription	Asp3, His4, Asp8
*Ab*	Sulfate transport system permease CysW	Transport/binding	His15, Asp17
*Ab*	Twitching motility protein PilT	Transport/binding	His73, Asp82
*Ab*	Taurine-binding periplasmic protein TauA	Transport/binding	Asp3
*Ab*	Inner membrane protein YfiN	Transport/binding	Asp224, Asp228
*Ab*	Mobile element protein	Gene mobility	His470
*Ab*	Plasmid mobilization protein	Gene mobility	His129
*Hp*	Iron(III) dicitrate transport protein FecA	Transport/binding	His21, H526
*Hp*	RNA pseudouridine synthase	metabolism	His36, His72
*Kp*	MATE family transport protein YeeO	transport/binding	His5
*Vv*	ABC transporter AzlC	transport/binding	Asp2, Asp9, His10

## Discussion

Protein phosphorylation is inherently linked to bacterial pathogenesis as it plays pivotal roles in signal transduction, modulation of metabolic behavior and confers antibiotic resistance [2–7]. Standard experimental method for phosphoproteomic studies employs TiO_2_-based metal oxide chromatography, which enriches phosphopeptides at low pH, coupled with standard LC-MS/MS characterizations in acidic buffers [9, 23, 24]. While this approach has been used to characterize peptides containing phosphorylated serine, threonine or tyrosine residues, it is rarely used to investigate the phosphorylation events of other amino acids as it remains unclear whether certain modified residues are sufficiently stable under these conditions. In this work, we applied highly stringent criteria for the identification of phosphorylated peptides isolated from various pathogens and demonstrated that the coupling of metal oxide chromatography with LC-MS can be used to identify unprecedented phospho-histidine and -aspartate peptides.

We began our analyses with *K. penumoniae* and *V. vulnificus*, the causative agents of pneumonia and cholera, respectively. Interestingly, two membrane proteins are found to undergo different degrees of phosphorylation. In *K. pneumoniae*, the multidrug and toxic compound extrusion (MATE) protein YeeO was phosphorylated at His5. The biophysical properties of MATEs have been intensively studied over the past few years because of their abilities to deliver antibiotics out of the bacteria via proton/sodium-coupled molecular pumping [33]. Indeed, *K. pneumoniae* YeeO shares 78% sequence identity with the *E. coli* homologue, which has been shown to reduce the host susceptibility to several structurally diverse antibiotics [35]. Though the exact mechanism has not been clearly elucidated, crystallography studies strongly suggest that this V-shaped channel needs to undergo substantial conformational change during molecular pumping, switching from the cytoplasm-opening state to the periplasm-opening state [43]. Since the N-terminal segment is located near the center of the channel on the periplasmic side, adding a negative charge through histidine phosphorylation will likely modify the dynamic behavior of this membrane protein. On the other hand, in *V. vulnificus* the AzlC protein of ATP binding cassette (ABC) transporter was found to be heavily phosphorylated at its N-terminus, including aspartate, histidine, serine and threonine (Fig. 4b). Both MATE and ABC transporters are major families of multidrug resistance transporters [33]. In addition, ABC transporters play significant roles in drug resistance not only in bacteria but also human tumors [33]. Studying the functional role of these post-translational modifications may therefore reveal new therapeutic strategy.

The mortality rate caused by the nosocomial multi-drug resistant *A. baumannii* has increased at an alarming rate during the past decade [44]. A wide range of proteins that contribute to antibiotic resistance are found to be phosphorylated at various positions, including histidine and aspartate. Noticeably, the transcription factor AraC is heavily phosphorylated at its N terminus. This family of transcription factor is widely distributed in diverse prokaryote genera and regulates genes involved in metabolism and virulence [37]. Phosphorylation of AraC was also identified in the nitrogen-fixing bacterium, *Sinorhizobium meliloti* [45]. Among different AraC transcription factors, the N-terminus domain is less conserved and is involved in allosteric regulation and dimerization by the co-inducer [46]. It is likely that AraC in *A. baumannii* is regulated via N-terminus phosphorylation, where the addition of a phosphate group induces other phosphorylation events as well as phosphate group transfer among the residues in vivo (Fig. 4a). Additionally, ZnuA, a key periplasmic transporter in the operon of the Zn storage system, is also heavily phosphorylated at the N-terminus [36]. Proteins in this transport system is attributed to enhance Zn ion chelation to the antibiotic calprotectin, thereby quenching its antibacterial effects. Homologues of other transport and binding proteins which have a direct link to drug resistance are also shown to be phosphorylated in *A. baumannii*. These include sulfate transport system permease CysW [38], twitching motility protein PilT [39], taurine-binding periplasmic protein TauA [40] and inner membrane protein YfiN [41]. Additionally, proteins involved in horizontal gene transfer, mobile element protein and plasmid mobilization protein, also undergo histidine and aspartate phosphorylations.

*H. pylori*, the causative agent of gastric ulcer, has been proven to enhance the development of gastric cancer, a major economic burden worldwide [47]. For the first time, we demonstrate that two non-two-component proteins undergo histidine phosphorylation in this pathogen. One of the modified proteins is FecA, an outer membrane protein responsible for the cellular uptake of iron(III) citrate [34]. Inside the human tissues, the free iron concentration is not sufficient to support bacterial growth and thus iron transport and storage are essential for the survival of *H. pylori* [34]. While the two-component EnvZ-OmpR system is known to regulate FecA at a translational level [48], other regulatory mechanisms of FecA remain unclear. Here, we demonstrated that FecA is likely to be regulated at a post-translational level by phosphorylation at His21 and His526 (Additional file 2). Homology modeling [49] showed that His21 is located at the periplasm side of the β-barrel transport protein, whereas His526 is close to the ligand binding site exposed (Additional file 4). Accordingly, they might involve in an unexplored signaling network with physiologically important cross-talk between these modifications. This work also revealed that pseudouridine synthase in *H. pylori* is phosphorylated at several of the histidine, serine and threonine positions. Site-specific uridine isomerization within a RNA molecule is the most common post-transcriptional modification found in prokaryotes. By forming a hydrolysis-resilient non-canonical C5-glycosidic bond, pseudouridine synthase is capable to enhance the structural stability of the RNA molecules and consequently cellular stability [42]. In this work, we showed that the histidine phosphorylation sites (position 36 and 72) of the pseudouridine synthase are located in the S4 RNA-binding domain. The addition of negative charges by these post-translation modifications may modulate the substrate binding affinity of this enzyme.

By switching our proteomic focus, we have revealed novel protein phosphorylation of over ten pathogenesis-related proteins found in several commonly seen pathogens. As demonstrated by the current and previous work by us and others, enrichment of phosphopeptides by TiO_2_ is highly robust, as it is highly specific toward phosphopeptides with femtomolar sensitivity and large tolerance of salts and detergents [9]. However, there was a controversy on whether the acidic condition used in standard LC-MS (typically 0.1% formic acid in running buffer) is too harsh for certain phosphorylated amino acid residues. It has been shown that phosphorylation of histidine and aspartate are less stable than those of serine, threonine and tyrosine; free phosphohistidine residue has also been shown to degrade in low-pH buffer, whereas free phosphoaspartate residue appears to be unstable in both acidic and alkali conditions [50]. However, the half-lives of the phospho-histidine and -aspartate peptides are most likely to be sequence-dependent. Hydrophobic residues situated next to the phosphorylated residue appear to be more profound in our study (Fig. 3c), thus suggesting that they may protect the post-translationally modified residue from cleavage. Furthermore, individual peptides might adopt specific secondary conformations that slow the hydrolysis process. As an example, phosphohistidine in *Escherichia coli* HPr protein is actually more resilient to hydrolysis at low pH than under neutral conditions [51]. These factors most likely contribute to why phospho-histidine and -aspartate peptides can be characterized in this work. Indeed, in our view it is rather difficult to draw a consensus on the stability of phosphorylated peptides, as their sequences dictate their structures, physical properties and consequently their chemical stabilities.

Better methods to enrich and identify phospho-histidine and aspartate peptides are needed, as phosphopeptides in low abundance or with less chemical stability are unlikely to be found in regular characterization methods. For example, the acid-labile arginine phosphorylation was found only in *Bacillus subtilis* strain with arginine phosphatase knocked out [52]. Hence, genetic removal of other phosphatases might enhance characterizations of different phosphopetides. Performing the MS analysis in neutral buffer may also improve the characterization of histidine phosphorylation, though the experimental condition is currently less refined than that of the acidic counterpart [10]. Lastly, novel chemical biology tools, such as reagents that target and report histidine and aspartate phosphorylations, will likely find uses in the field of phosphoproteomics. This work demonstrates that there is an additional level of protein regulations beyond our current knowledge of microbial molecular biology. Most importantly, this work illustrated new research directions for over ten proteins that are related to pathogen survival and antibiotic resistance. Hence, it will be of paramount interest to identify the functional impact of the identified phosphorylation, as well as the designated kinases responsible for the post-translational modifications. Such information may provide novel insights and targets to combat the current antibiotic resistance crisis.

## Conclusions

We performed site-specific His/Asp phosphoproteomic analysis of nine prokaryotes, including four pathogenic bacteria isolated from hospital patients. The use of highly stringent conditions ensured unambiguous identification of the phosphorylated sites, including histidine and aspartate, which were previously thought to be uncharacterizable under standard acidic LC-MS conditions. This work clearly illustrates that His/Asp phosphorylation events occur beyond two-component systems. Noticeably, for the first time a number of key pathogenesis-related proteins are shown to undergo His and Asp phosphorylations. These findings have shed light on important research directions, which will eventually find uses in combating the emergence of antibiotic resistance and designing new therapeutics in the future.

## Additional files

Additional file 1: MS/MS Spectra. Spectra of the identified phosphopeptides (PDF 4714 kb).

Additional file 2: Table S1. Phospho probabilities, process method, leading protein identifier, UniProt No. (if available), protein description, and protein functional class of the identified phosphopeptides (PDF 539 kb).

Additional file 3: Figure S1. The relative abundances of amino acids between −10 and +10 positions of the phosphorylated or non-phosphorylated His/Asp (PDF 377 kb).

Additional file 4: Figure S2. Homology model of H. pylori FecA (PDF 359 kb).

## Abbreviations

ABC: ATP binding cassette
ACN: Acetonitrile
DTT: Dithiothreitol
LC: Liquid chromatography
MATE: Multidrug and toxic compound extrusion
MS: Mass spectrometry
PBS: Phosphate-buffered saline
TFA: Trifluoroacetic acid
