# Supplementary material for: Site-specific His/Asp phosphoproteomic analysis of prokaryotes reveals putative targets for drug resistance
Source: BMC Microbiol. 2017 May 25;17:123. doi: 10.1186/s12866-017-1034-2 (PMC5445275; doi:10.1186/s12866-017-1034-2)
Supplement: Supplementary file 1 — MS/MS Spectra. Spectra of the identified phosphopeptides (PDF 4714 kb). [file 12866_2017_1034_MOESM1_ESM.pdf]

MS/MS Spectra

Arthrospira platensis C1 (cyanobacterium)

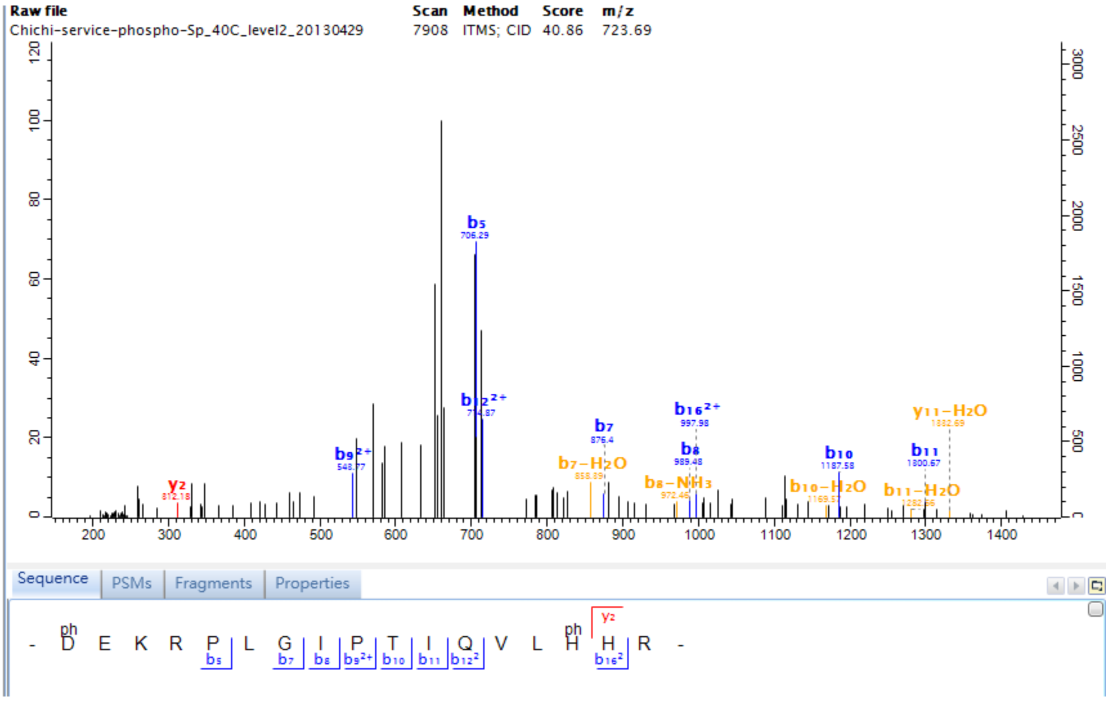

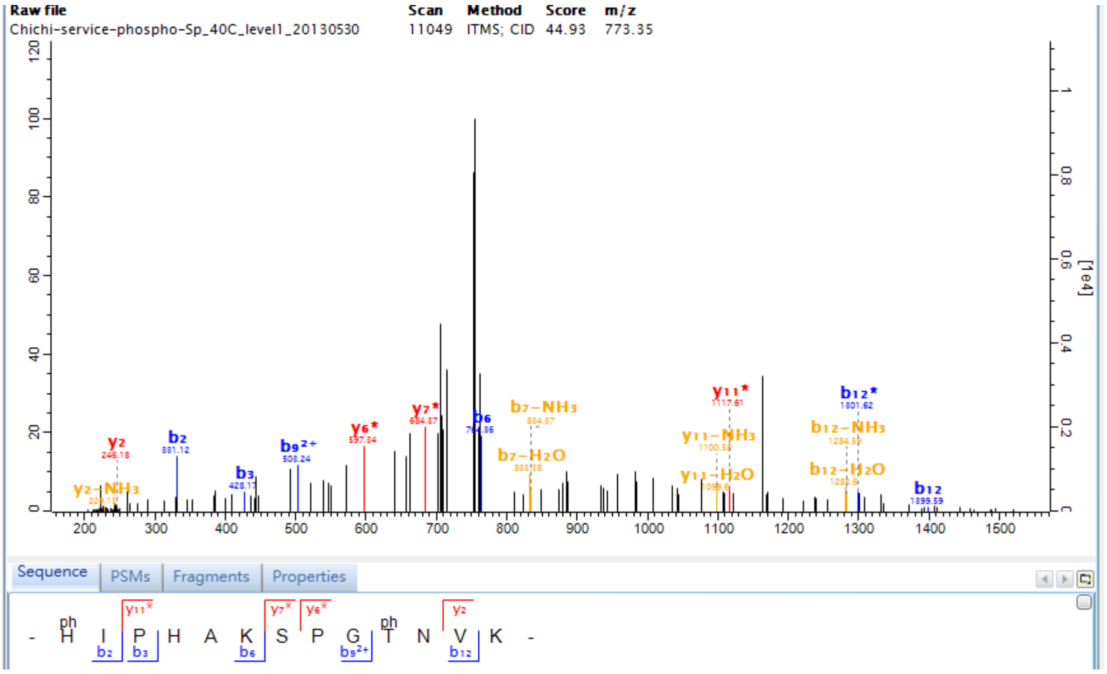

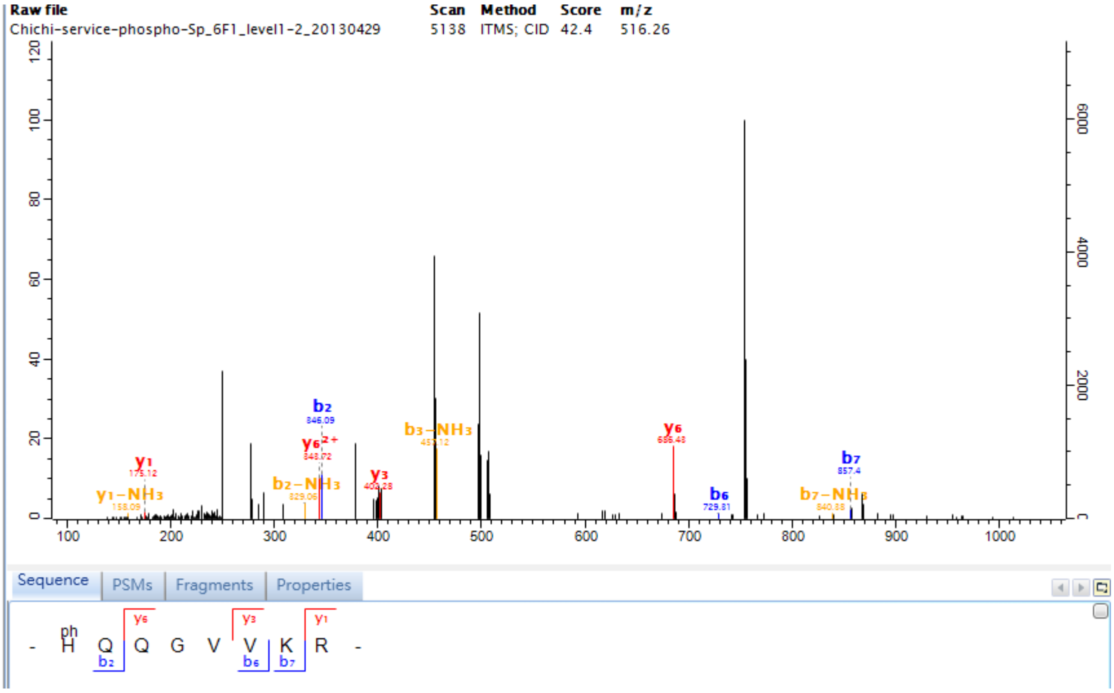
Raw file
Chichi-service-phospho-Sp_6F1_level1-2_20130429
Scan Method Score m/z
5138 ITMS; CID 42.4 516.26
y1-NH3
y1
b2-NH3
b2
y6 2+
y3
b3-NH3
y6
b6
b7-NH3
b7
Sequence
PSMs
Fragments
Properties
- H Q Q G V V K R -
ph

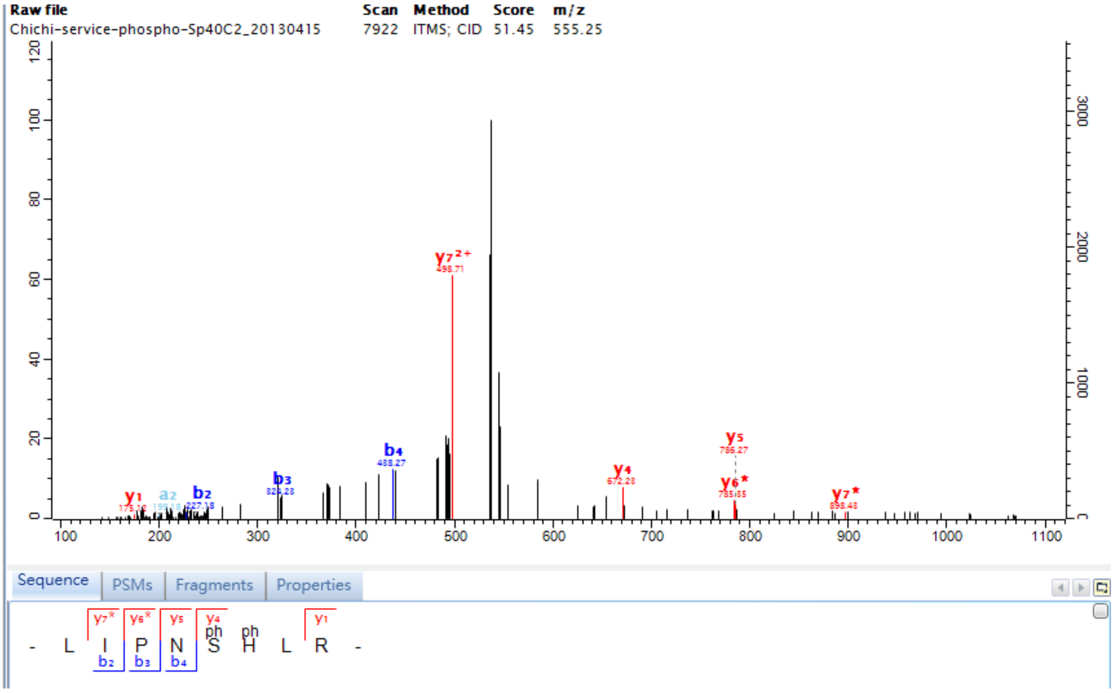
Raw file
Chichi-service-phospho-Sp40C2_20130415
Scan Method Score m/z
7922 ITMS; CID 51.45 555.25
y1
a2
b2
b3
b4
y7 2+
y4
y5
y6*
y7*
Sequence
PSMs
Fragments
Properties
- L I P N S H L R -
ph ph

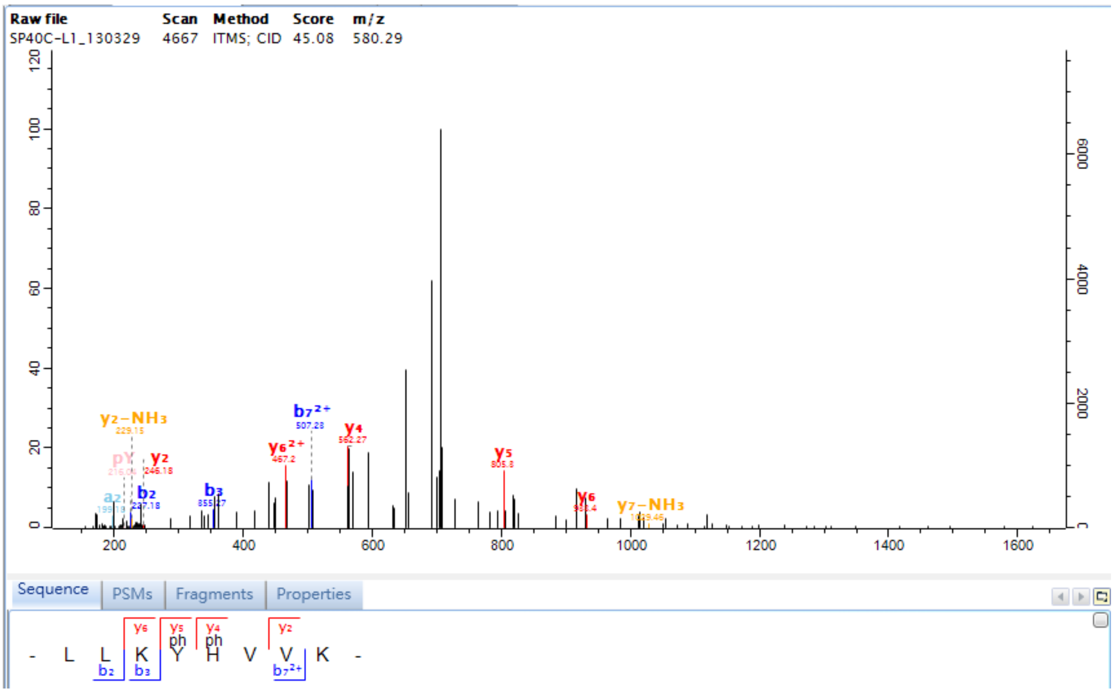
Raw file Scan Method Score m/z
SP40C-L1_130329 4667 ITMS; CID 45.08 580.29
y2-NH3
y2
b2
b3
b7 2+
y6 2+
y4
y5
y6
y7-NH3
Sequence PSMs Fragments Properties
- L L K Y H V V K -

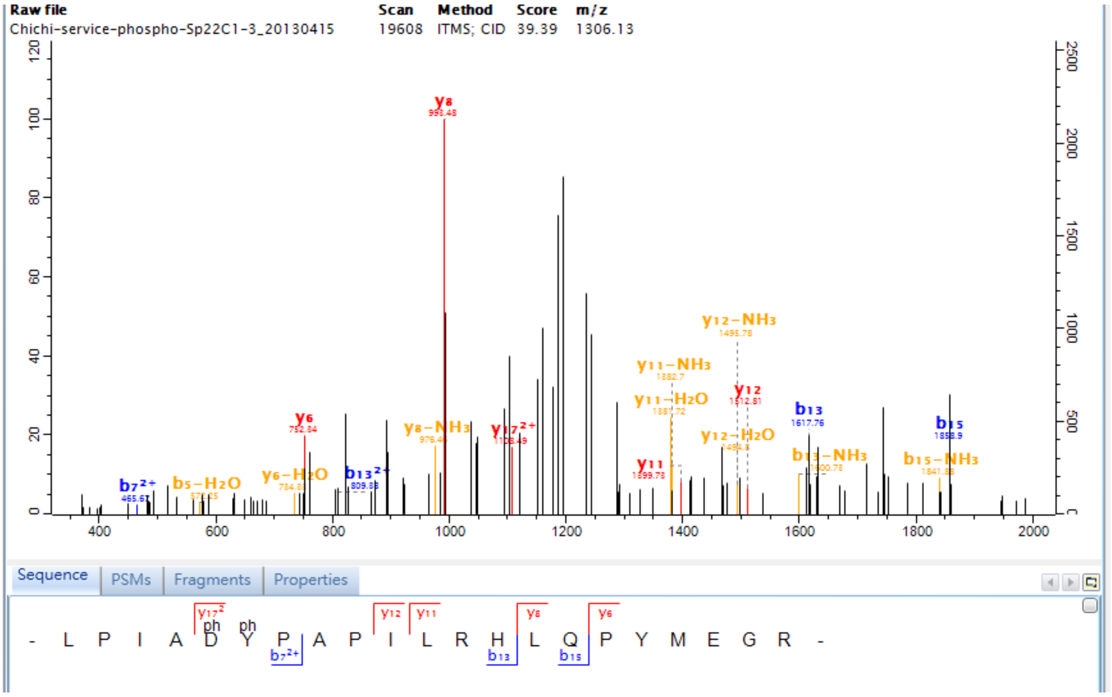
Raw file Scan Method Score m/z
Chichi-service-phospho-Sp22C1-3_20130415 19608 ITMS; CID 39.39 1306.13
y8
y6
b7 2+
b5-H2O
y6-H2O
b13 2+
y8-NH3
y17 2+
y11-NH3
y11-H2O
y11
y12-NH3
y12
y12-H2O
b13
b13-NH3
b15
b15-NH3
Sequence PSMs Fragments Properties
- L P I A D Y P A P I L R H L Q P Y M E G R -

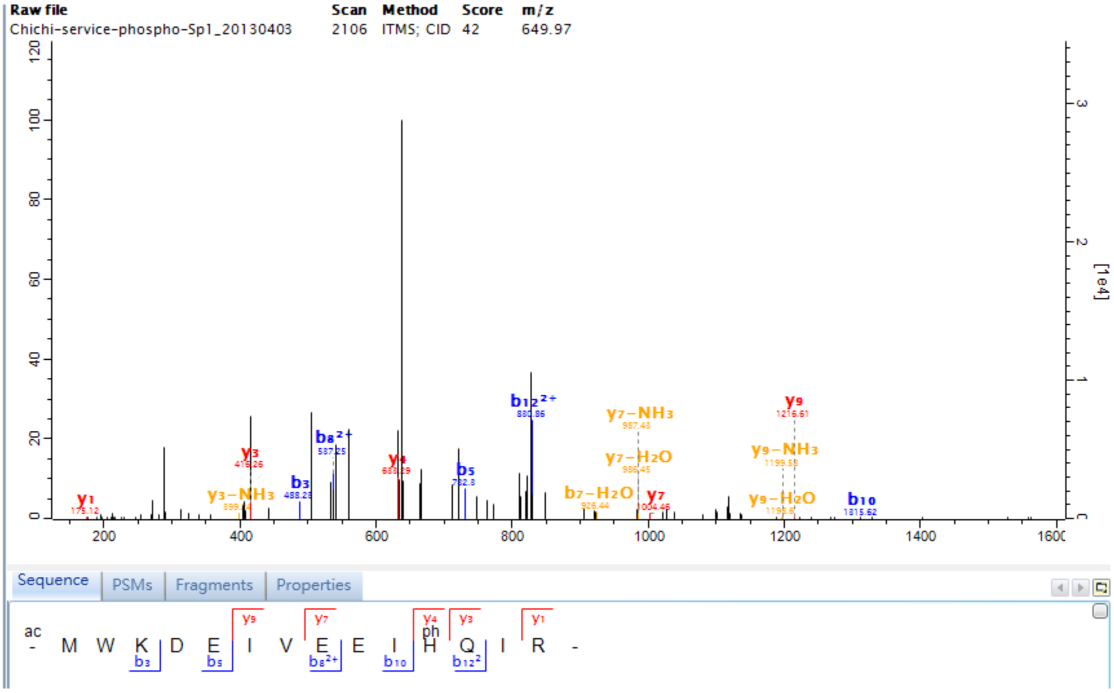

Raw file
Chichi-service-phospho-Sp1_20130403
Scan
2106
Method
ITMS; CID
Score
42
m/z
649.97
Sequence
PSMs
Fragments
Properties
ac - M W K D E I V E E I H Q I R -

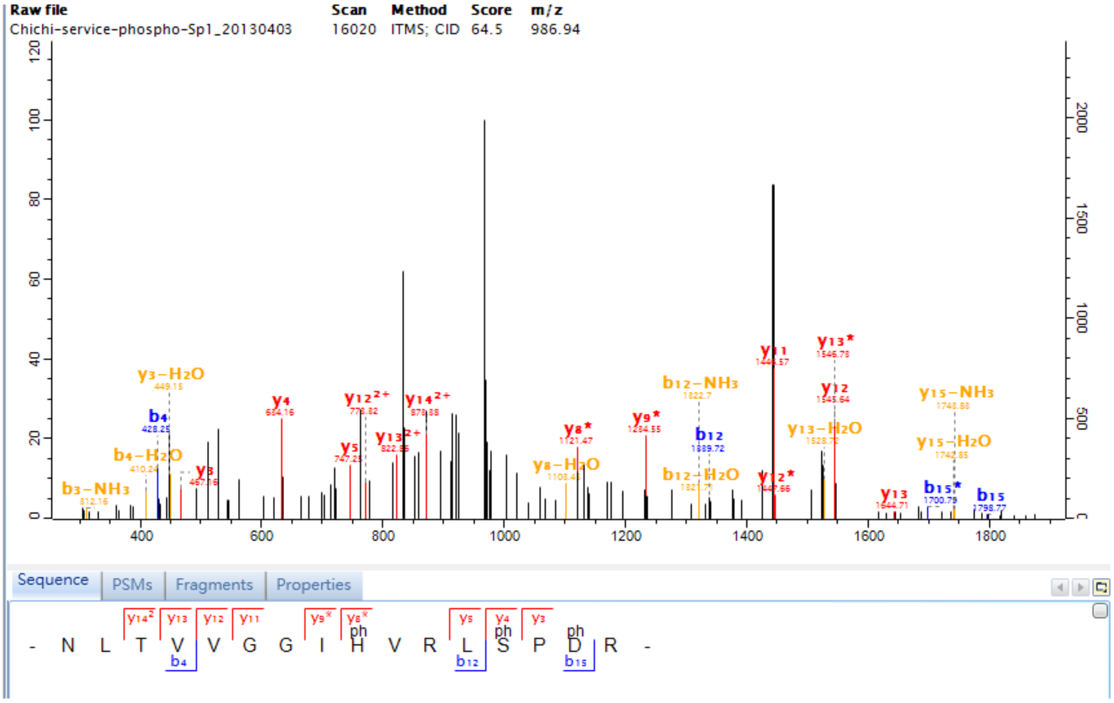

Raw file
Chichi-service-phospho-Sp1_20130403
Scan
16020
Method
ITMS; CID
Score
64.5
m/z
986.94
Sequence
PSMs
Fragments
Properties
- N L T V V G G I H V R L S P D R -

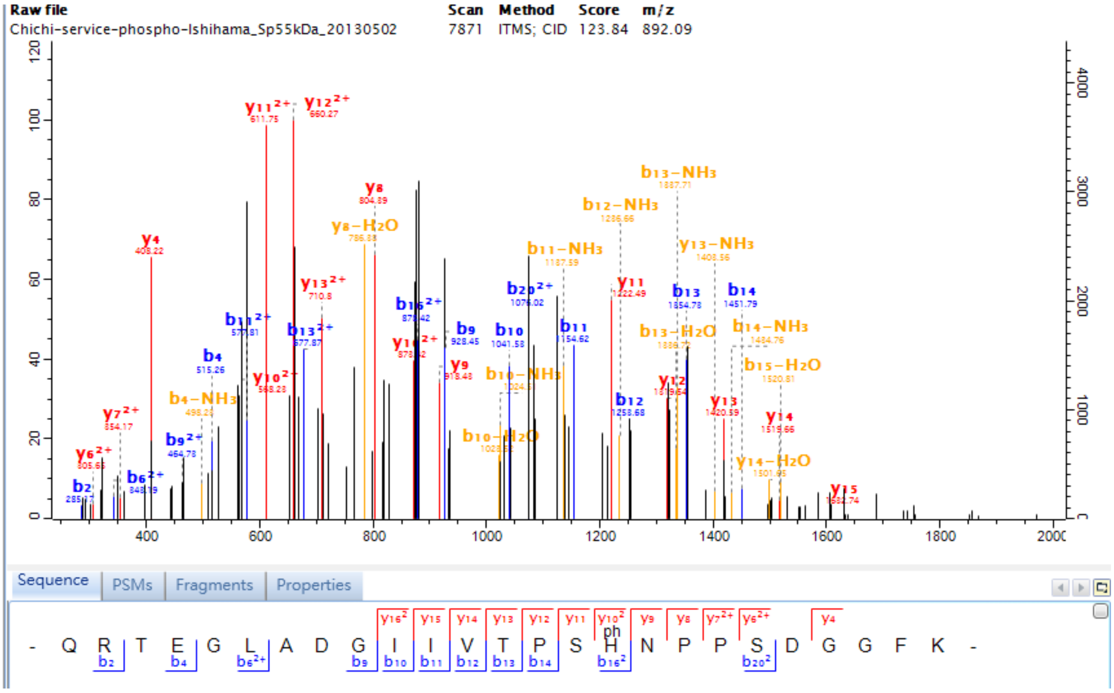
Raw file
Chichi-service-phospho-Ishihama_Sp55kDa_20130502
Scan Method Score m/z
7871 ITMS; CID 123.84 892.09
Sequence PSMs Fragments Properties
- Q R T E G L A D G I I V T P S H N P P S D G G F K -

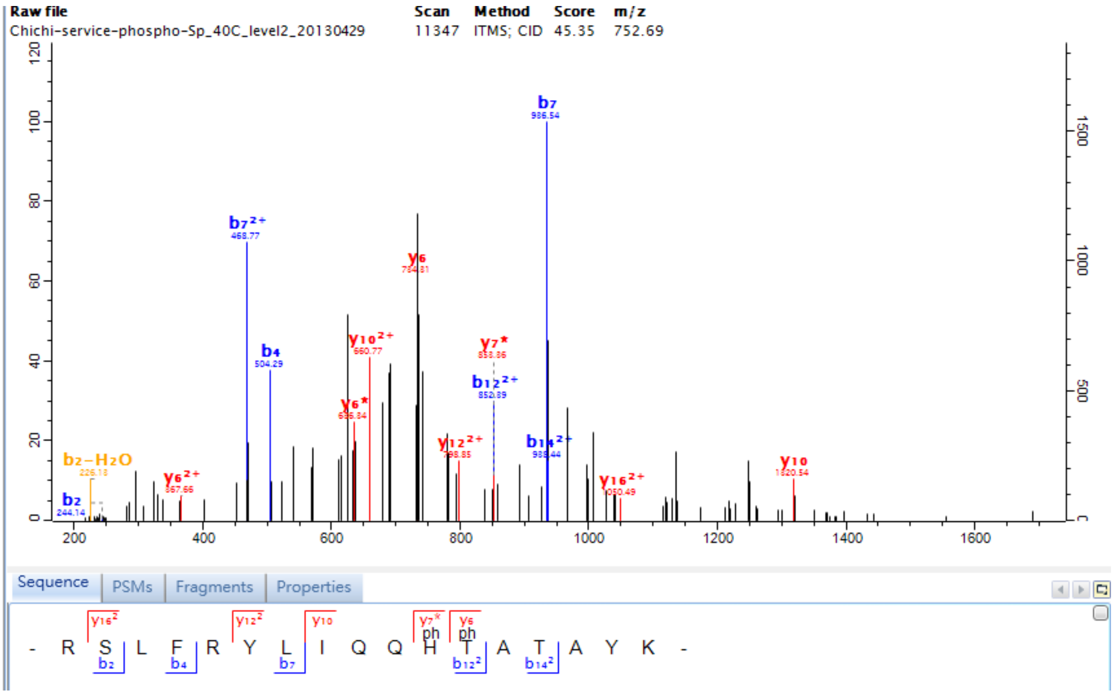
Raw file
Chichi-service-phospho-Sp_40C_level2_20130429
Scan Method Score m/z
11347 ITMS; CID 45.35 752.69
Sequence PSMs Fragments Properties
- R S L F R Y L I Q Q H T A T A Y K -

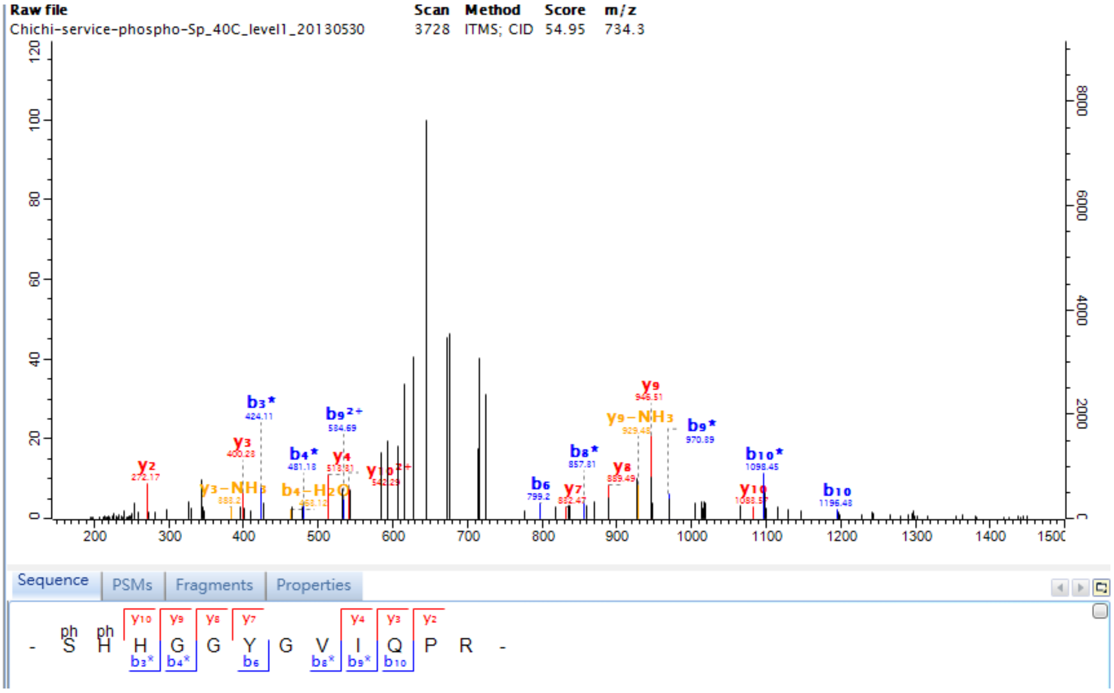
Raw file
Chichi-service-phospho-Sp_40C_level1_20130530
Scan Method Score m/z
3728 ITMS; CID 54.95 734.3
Sequence PSMs Fragments Properties
- S H H G G Y G V I Q P R -

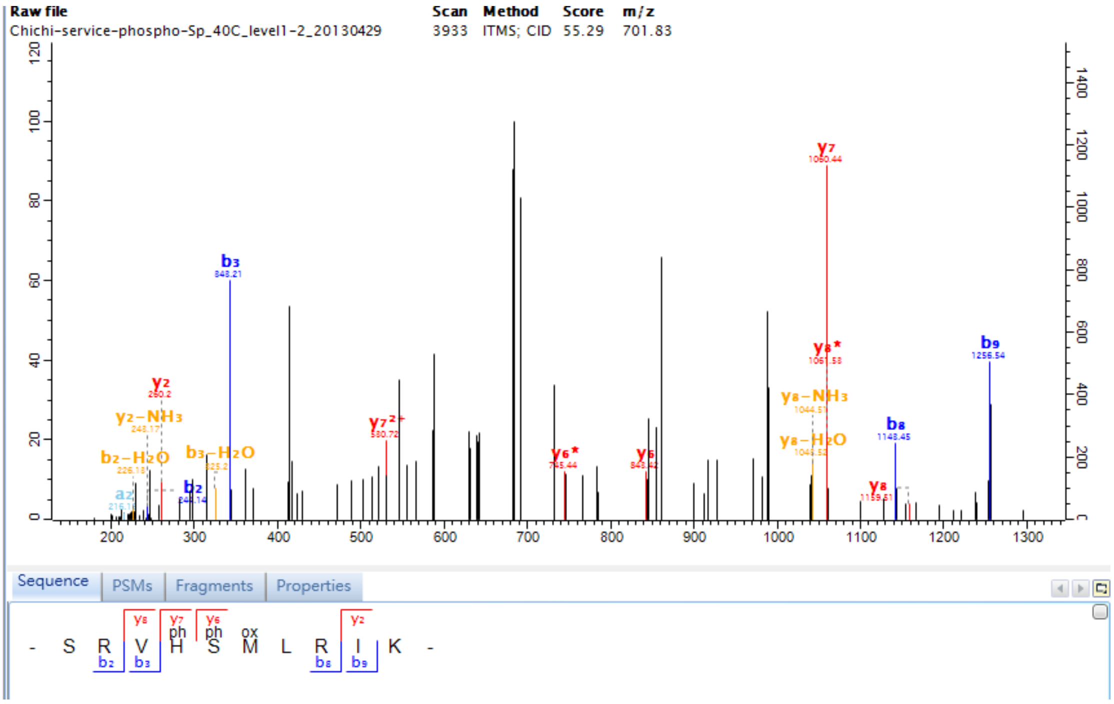
Raw file
Chichi-service-phospho-Sp_40C_level1-2_20130429
Scan Method Score m/z
3933 ITMS; CID 55.29 701.83
Sequence PSMs Fragments Properties
- S R V H S M L R I K -

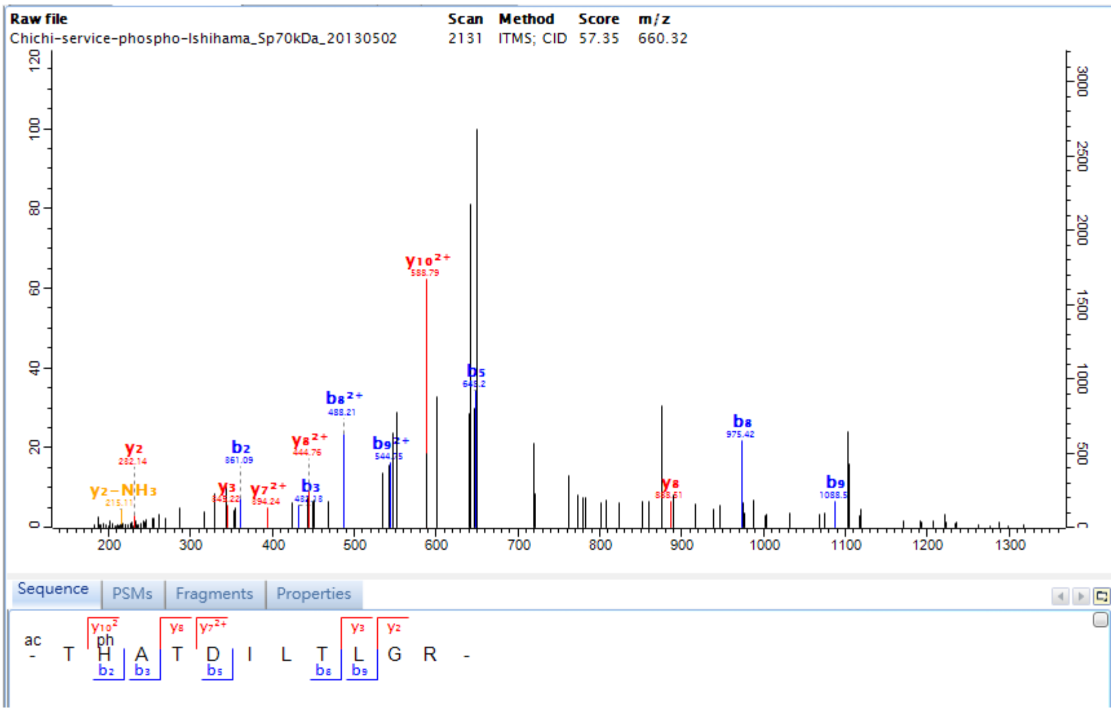
Raw file
Chichi-service-phospho-Ishihama_Sp70kDa_20130502
Scan Method Score m/z
2131 ITMS; CID 57.35 660.32
y10 2+
y2-NH3
y2
b2
y3
y7 2+
y8 2+
b3
b8 2+
b9 2+
b5
y8
b8
b9
Sequence PSMs Fragments Properties
ac - T H A T D I L T L G R -

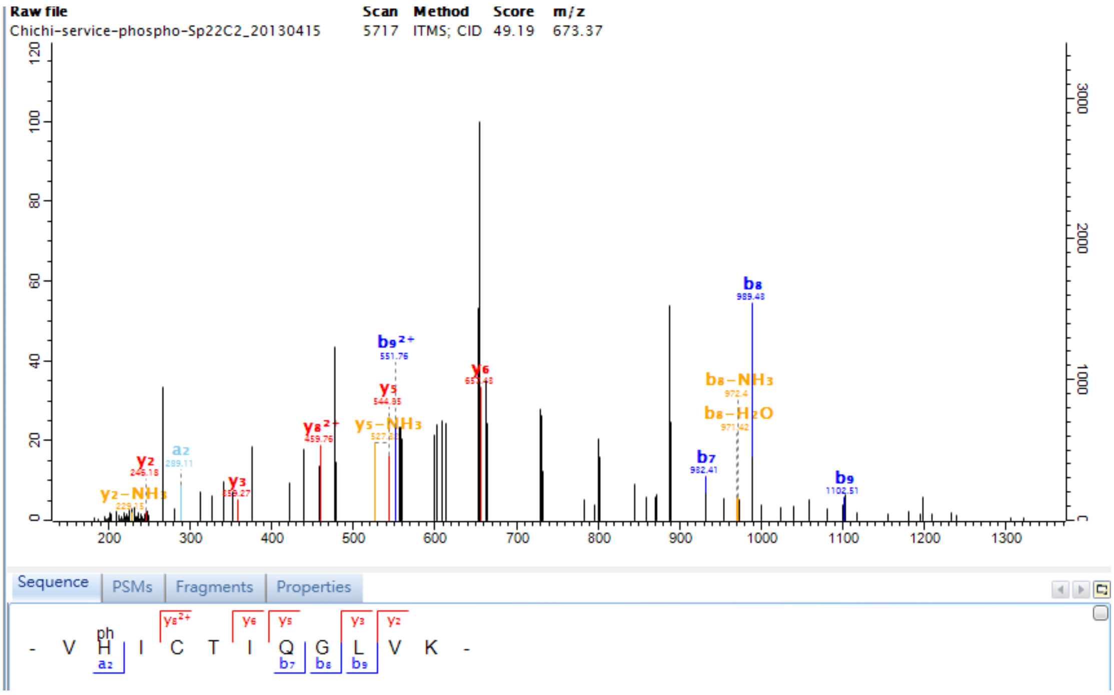
Raw file
Chichi-service-phospho-Sp22C2_20130415
Scan Method Score m/z
5717 ITMS; CID 49.19 673.37
y2-NH3
y2
a2
y3
y8 2+
y5-NH3
y5
b9 2+
y6
b7
b8-H2O
b8-NH3
b8
b9
Sequence PSMs Fragments Properties
- V H I C T I Q G L V K -

Acinetobacter baumannii SK17 (pathogenic bacterium)

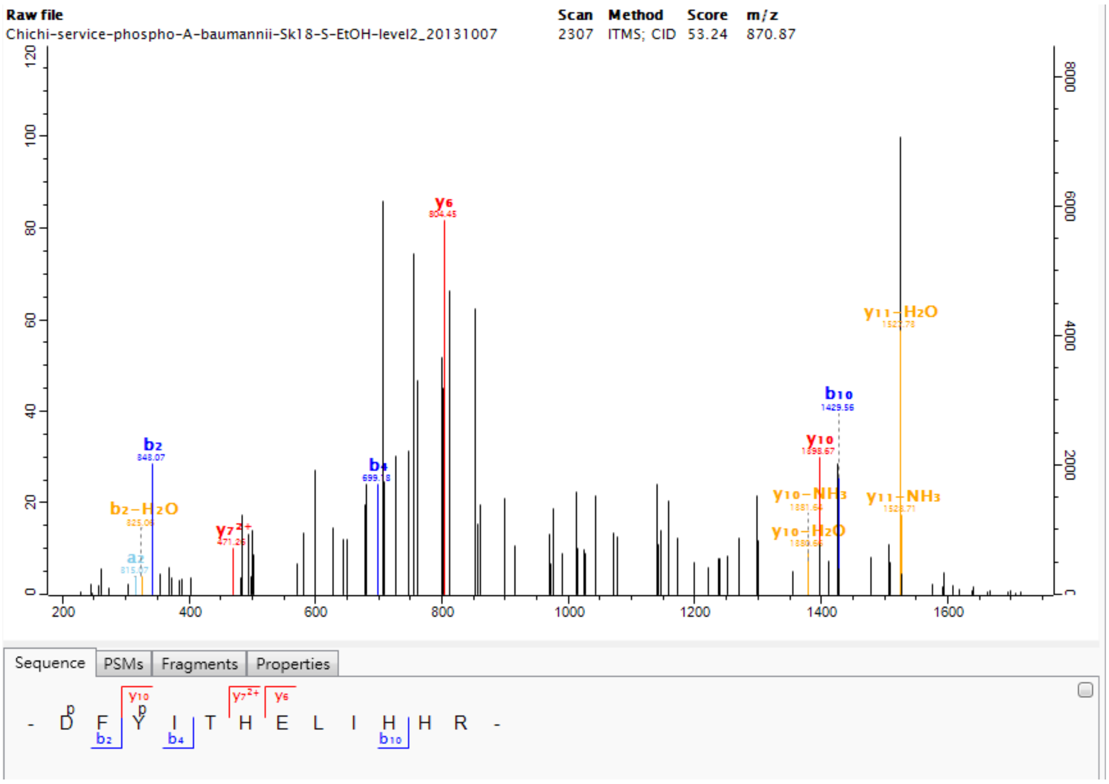

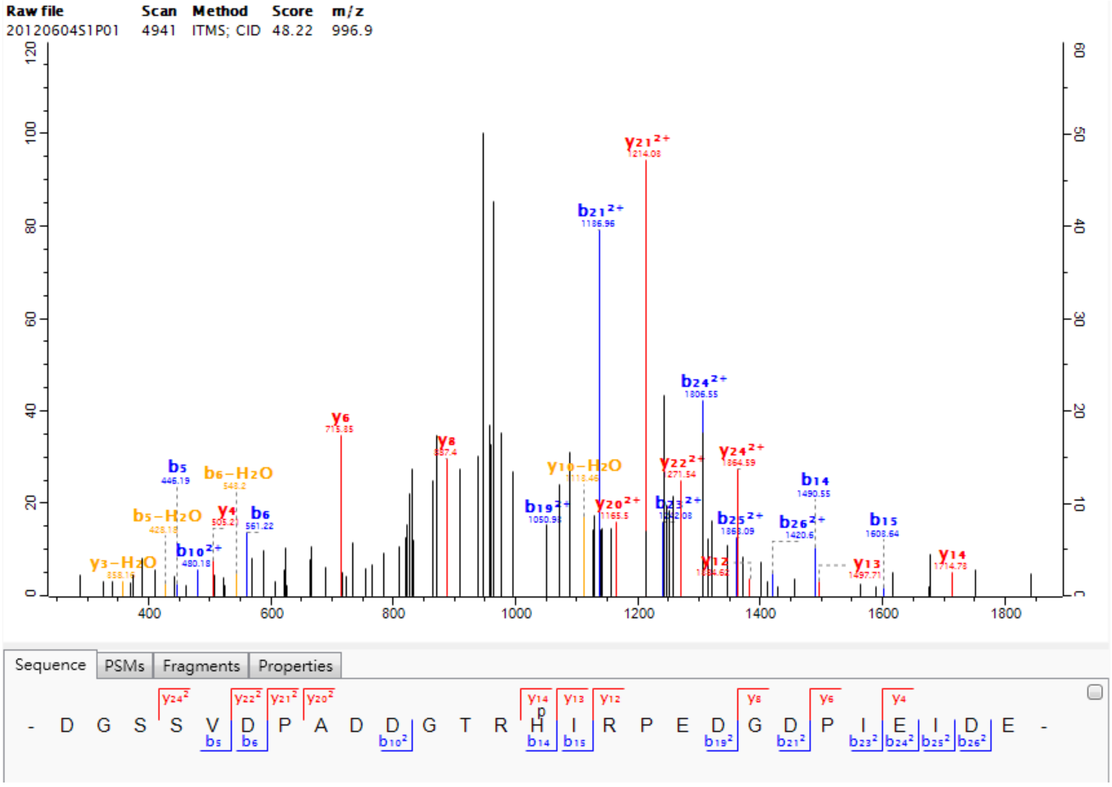

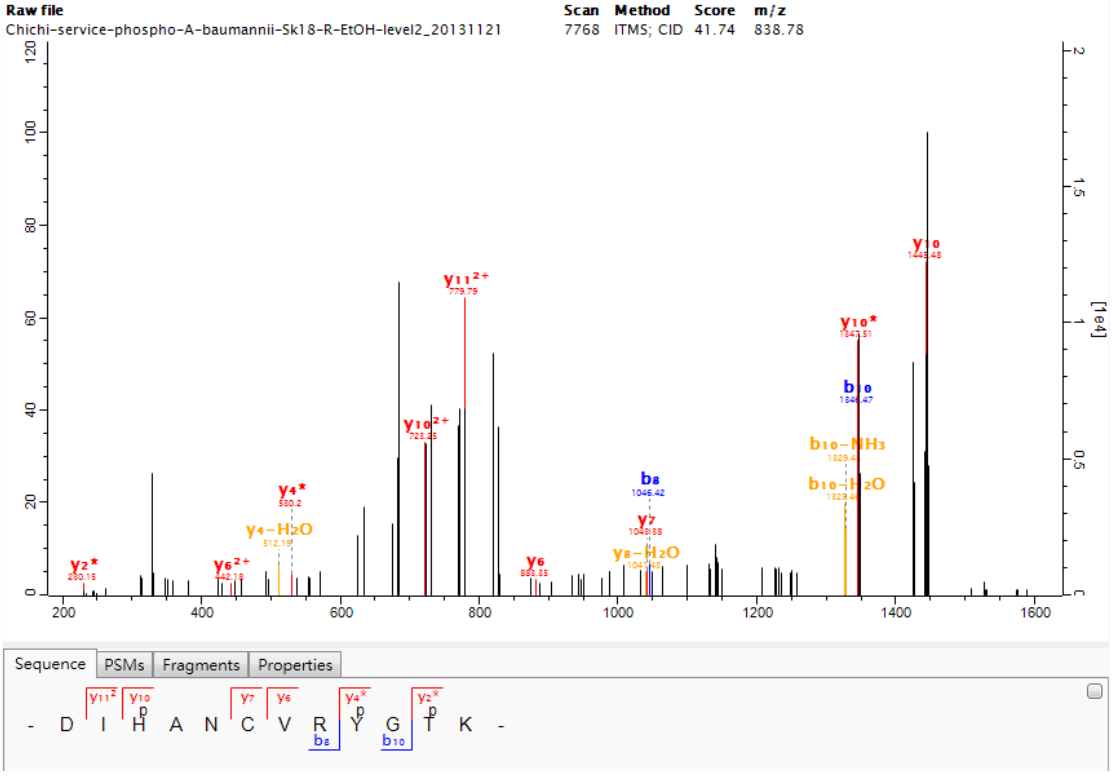
Raw file
Chichi-service-phospho-A-baumannii-Sk18-R-EtOH-level2_20131121
Scan Method Score m/z
7768 ITMS; CID 41.74 838.78
[1e4]
Sequence PSMs Fragments Properties
- D I H A N C V R Y G T K -

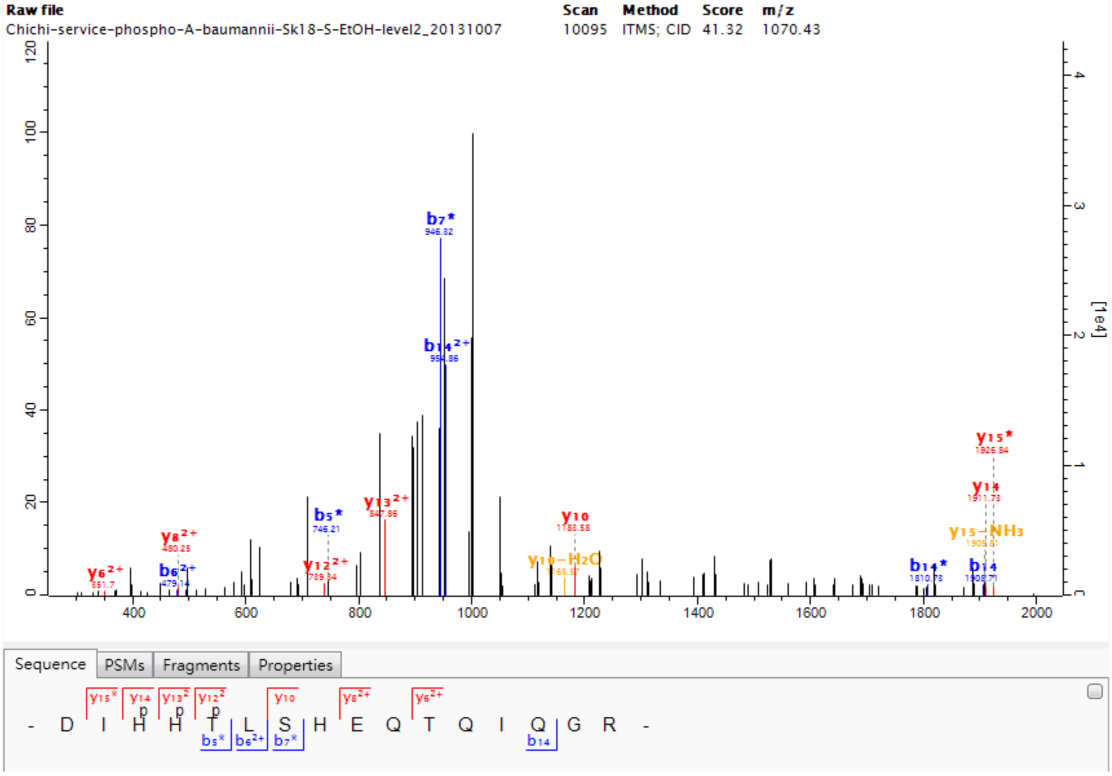
Raw file
Chichi-service-phospho-A-baumannii-Sk18-S-EtOH-level2_20131007
Scan Method Score m/z
10095 ITMS; CID 41.32 1070.43
[1e4]
Sequence PSMs Fragments Properties
- D I H H T L S H E Q T Q I Q G R -

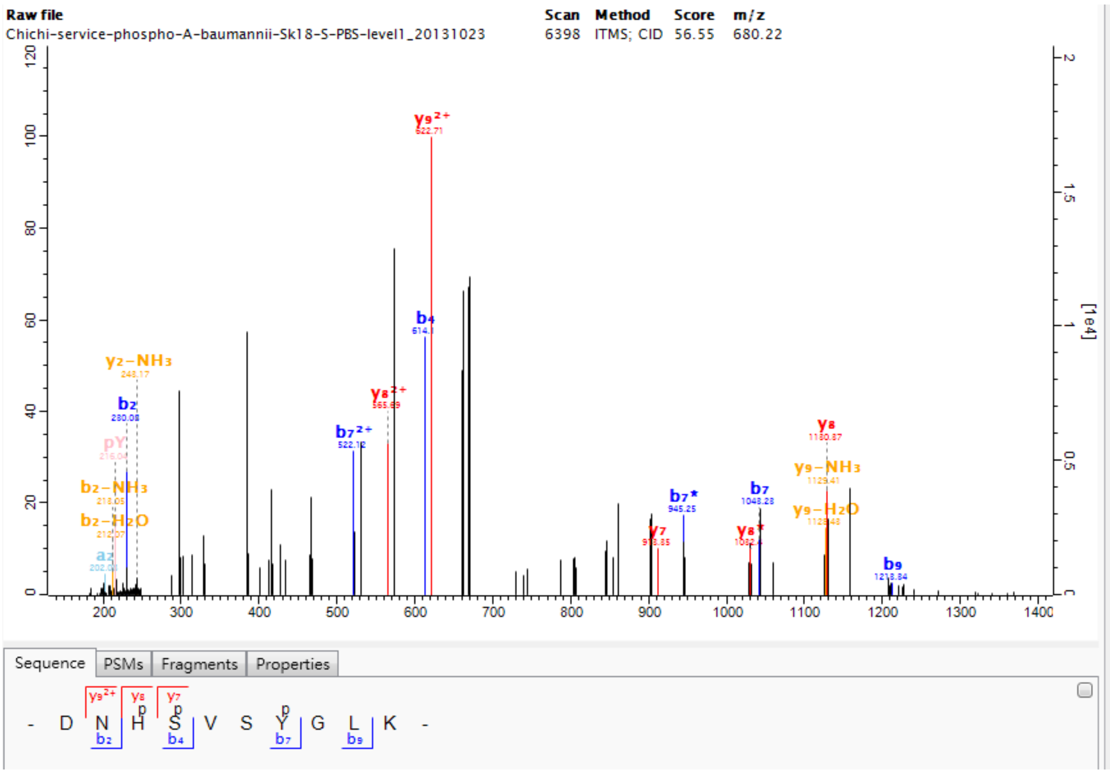
Raw file
Chichi-service-phospho-A-baumannii-Sk18-S-PBS-level1_20131023
Scan Method Score m/z
6398 ITMS; CID 56.55 680.22
[1e4]
Sequence
PSMs
Fragments
Properties
- D N H S V S Y G L K -

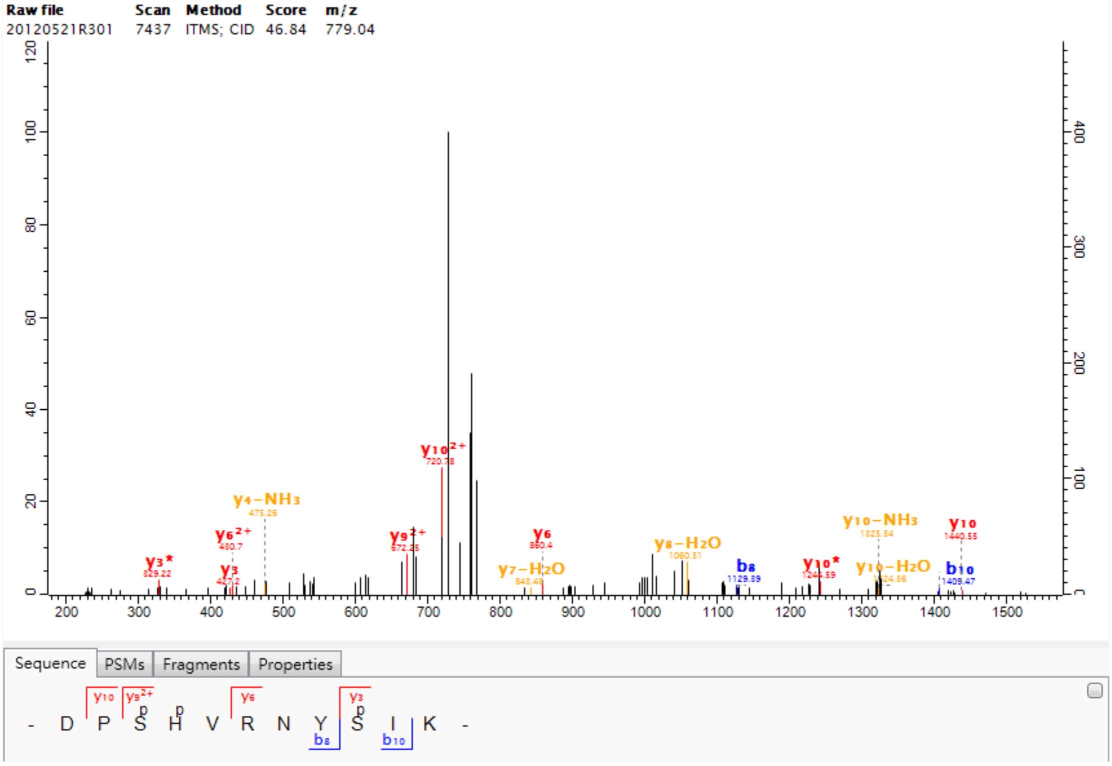
Raw file
20120521R301
Scan Method Score m/z
7437 ITMS; CID 46.84 779.04
Sequence
PSMs
Fragments
Properties
- D P S H V R N Y S I K -

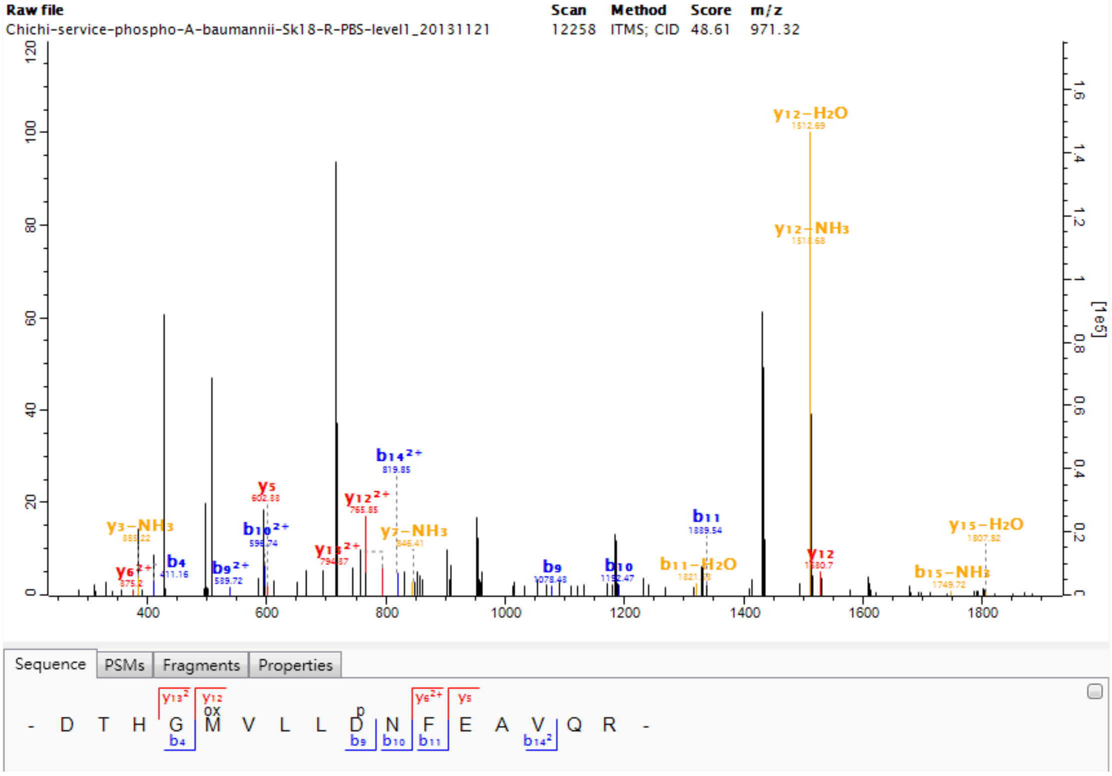
Raw file
Chichi-service-phospho-A-baumannii-Sk18-R-PBS-level1_20131121
Scan Method Score m/z
12258 ITMS; CID 48.61 971.32
[1e5]
Sequence
PSMs
Fragments
Properties
- D T H G M V L L D N F E A V Q R -

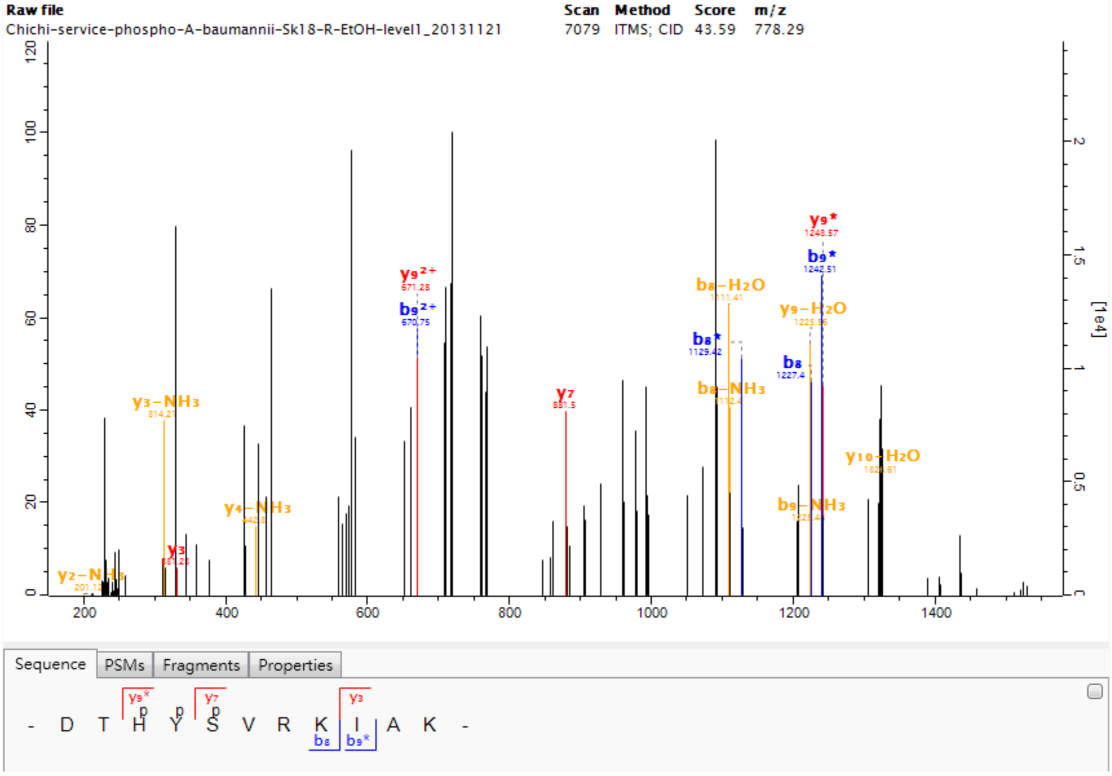
Raw file
Chichi-service-phospho-A-baumannii-Sk18-R-EtOH-level1_20131121
Scan Method Score m/z
7079 ITMS; CID 43.59 778.29
[1e4]
Sequence
PSMs
Fragments
Properties
- D T H Y S V R K I A K -

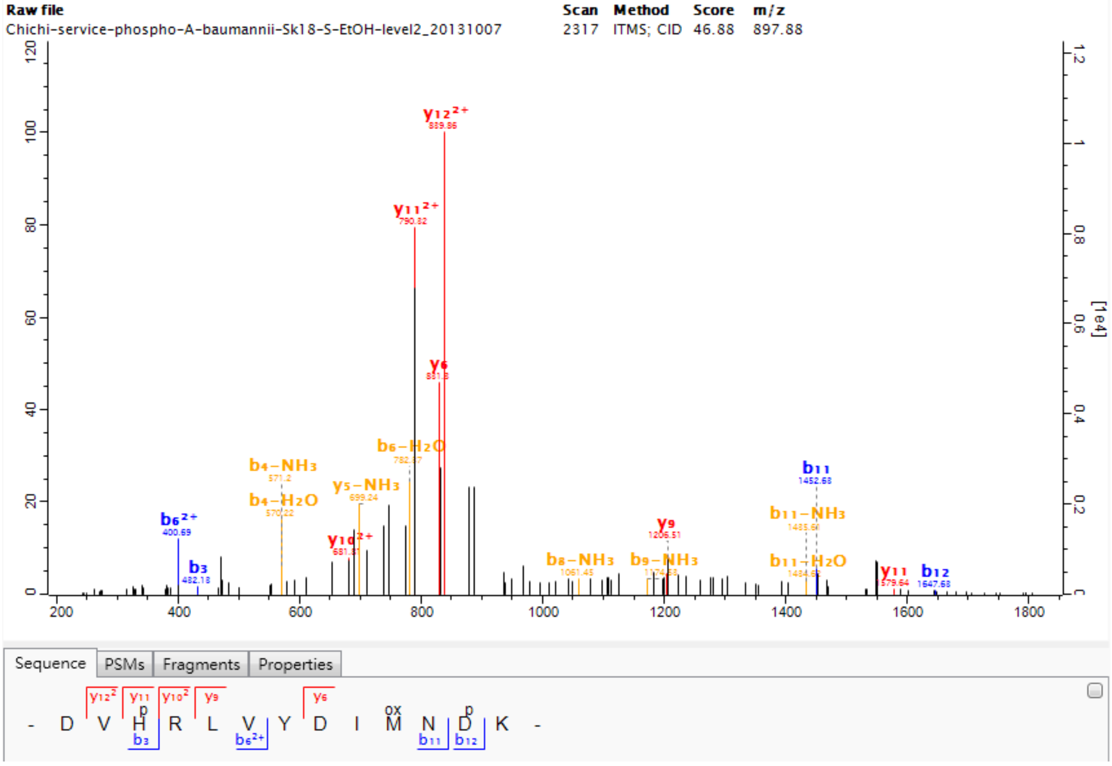
Raw file
Chichi-service-phospho-A-baumannii-Sk18-S-EtOH-level2_20131007
Scan 2317
Method ITMS; CID
Score 46.88
m/z 897.88
y12²⁺
y11²⁺
y6
b6-H2O
b4-NH3
y5-NH3
b4-H2O
b6²⁺
y10²⁺
b3
b11
b11-NH3
b8-NH3
b9-NH3
y9
b11-H2O
y11
b12
[1e4]
Sequence
PSMs
Fragments
Properties
- D V H R L V Y D I M N D K -

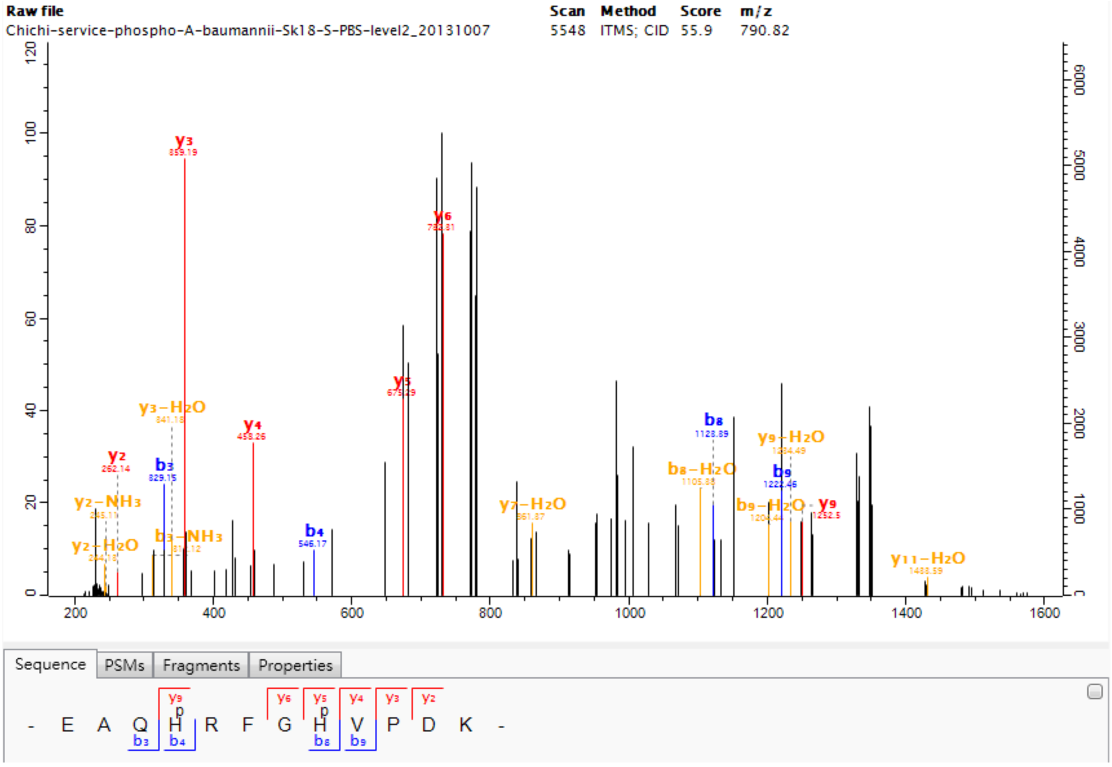
Raw file
Chichi-service-phospho-A-baumannii-Sk18-S-PBS-level2_20131007
Scan 5548
Method ITMS; CID
Score 55.9
m/z 790.82
y3
y6
y5
y3-H2O
y4
y2
b3
y2-NH3
b3-NH3
y2-H2O
b4
y7-H2O
b8
y9-H2O
b8-H2O
b9
b9-H2O
y9
y11-H2O
Sequence
PSMs
Fragments
Properties
- E A Q H R F G H V P D K -

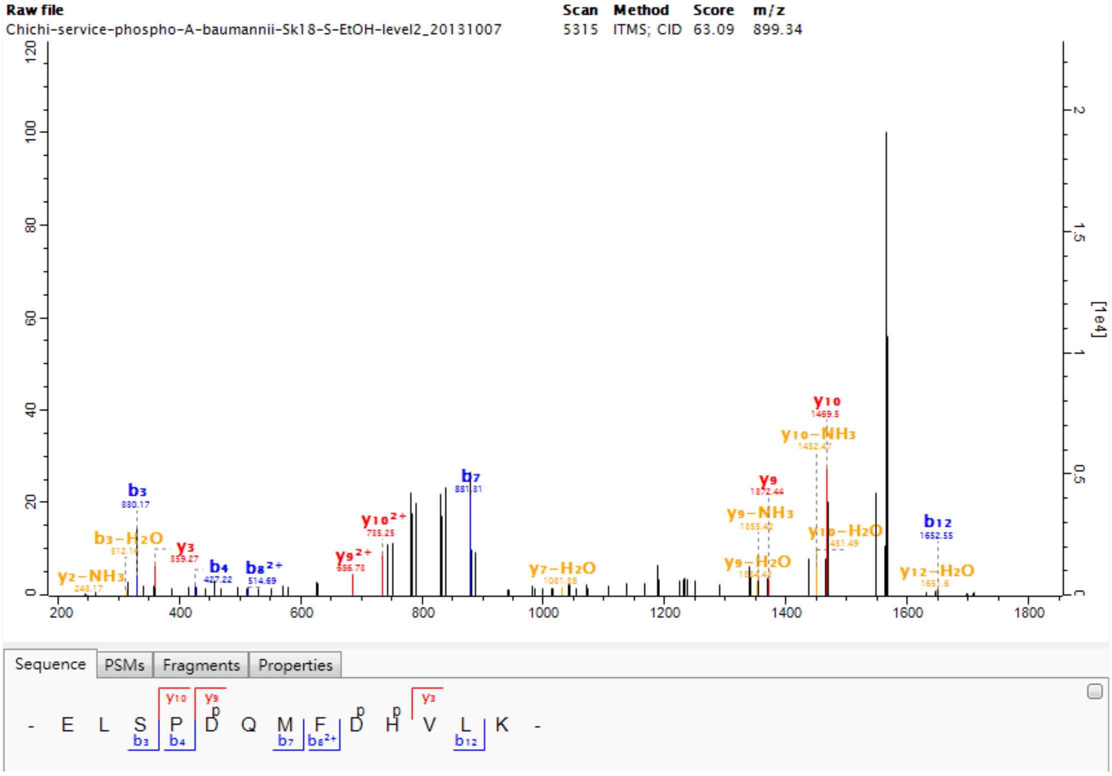
Raw file
Chichi-service-phospho-A-baumannii-Sk18-S-EtOH-level2_20131007
Scan 5315
Method ITMS; CID
Score 63.09
m/z 899.34
[1e4]
y10
y10−NH3
y9
y9−NH3
y9−H2O
y10−H2O
b12
y12−H2O
b7
b3
b3−H2O
y3
b4
b8 2+
y2−NH3
y10 2+
y9 2+
y7−H2O
Sequence
PSMs
Fragments
Properties
- E L S P D Q M F D H V L K -

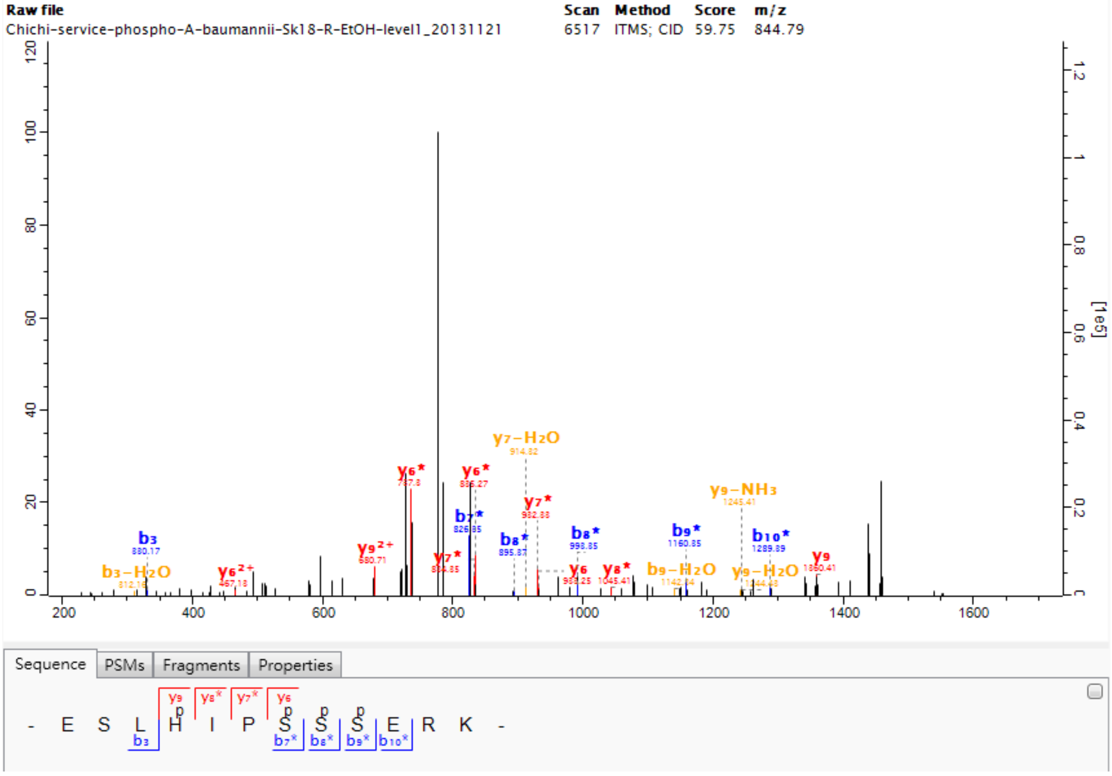
Raw file
Chichi-service-phospho-A-baumannii-Sk18-R-EtOH-level1_20131121
Scan 6517
Method ITMS; CID
Score 59.75
m/z 844.79
[1e5]
y7−H2O
y6*
y9−NH3
y7*
b7*
b8*
b9*
b10*
y9
b3
b3−H2O
y6 2+
y9 2+
y6
y8*
b9−H2O
y9−H2O
Sequence
PSMs
Fragments
Properties
- E S L H I P S S S E R K -

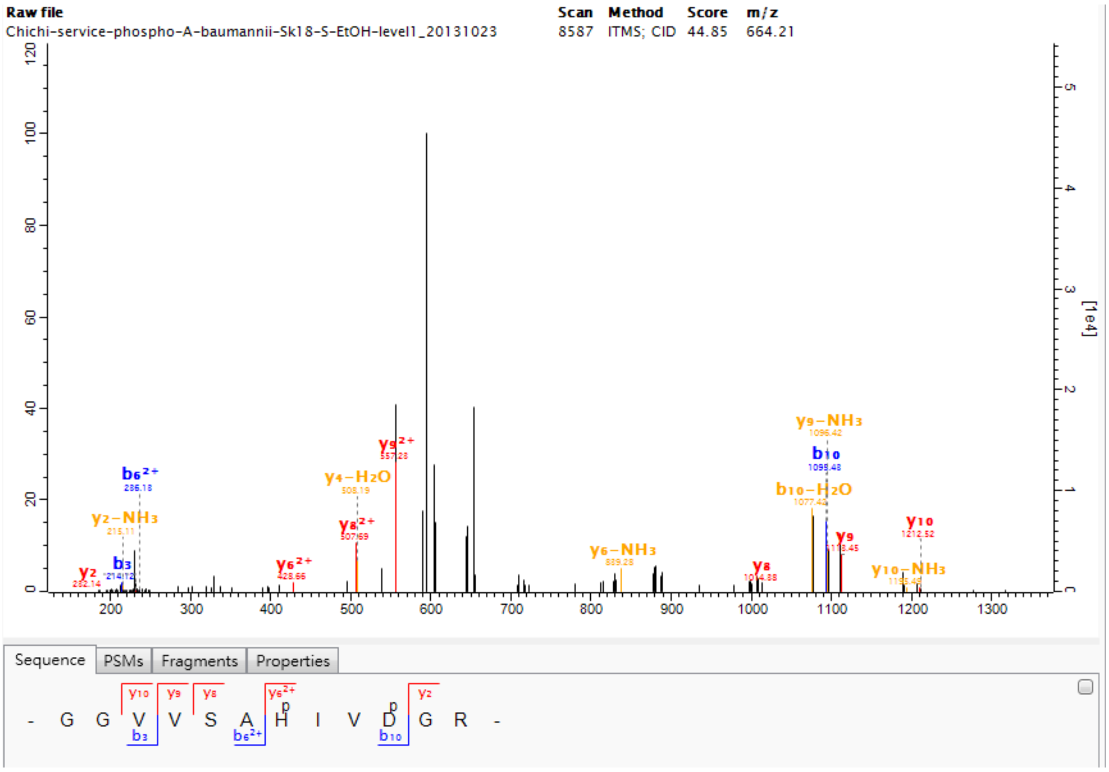
Raw file
Chichi-service-phospho-A-baumannii-Sk18-S-EtOH-level1_20131023
Scan Method Score m/z
8587 ITMS; CID 44.85 664.21
[1e4]
Sequence PSMs Fragments Properties
- G G V V S A H I V D G R -

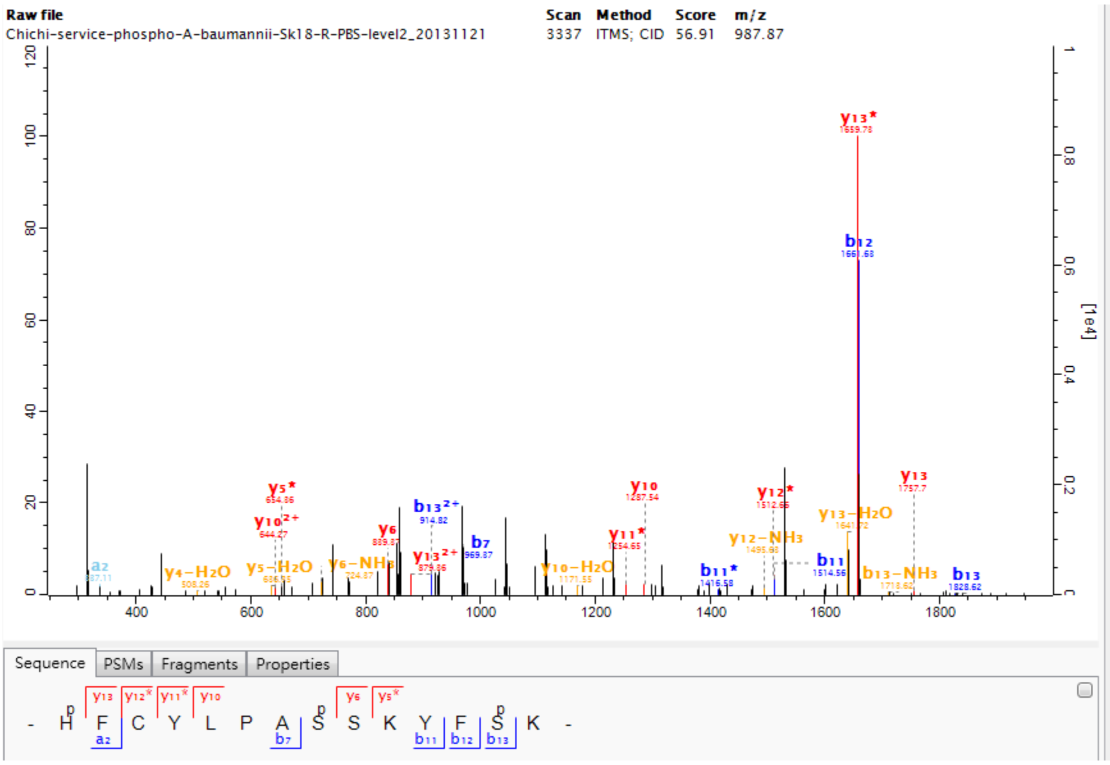
Raw file
Chichi-service-phospho-A-baumannii-Sk18-R-PBS-level2_20131121
Scan Method Score m/z
3337 ITMS; CID 56.91 987.87
[1e4]
Sequence PSMs Fragments Properties
- H F C Y L P A S S K Y F S K -

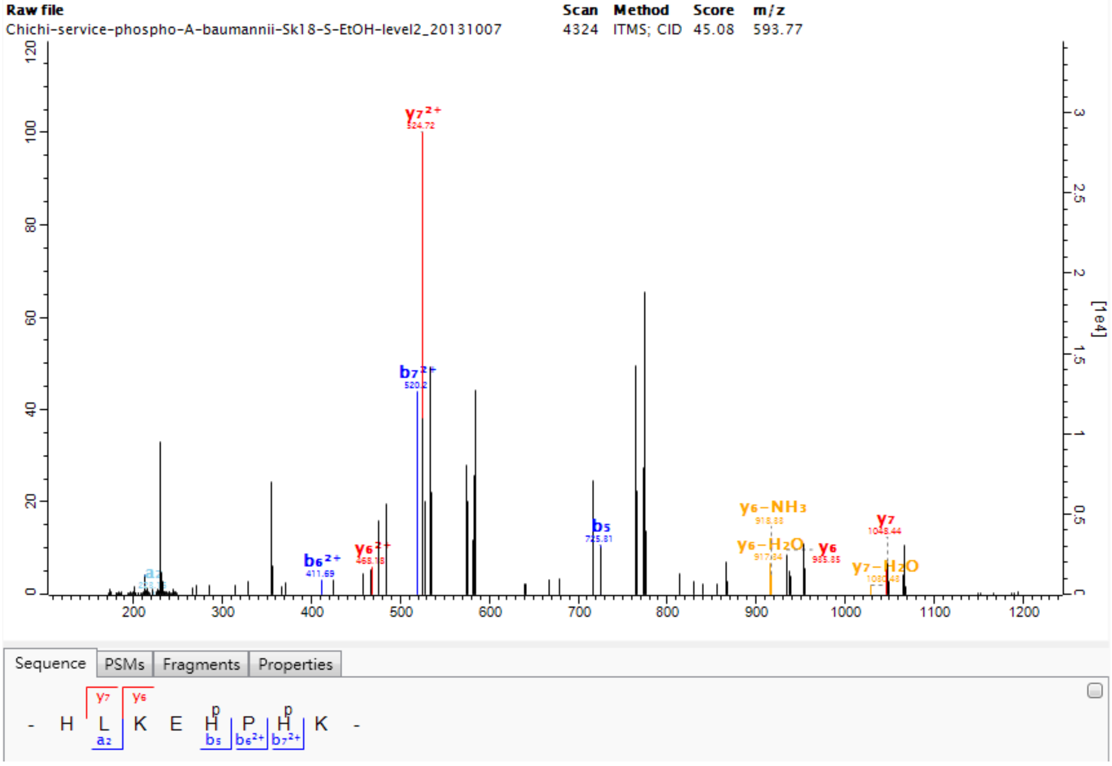
Raw file
Chichi-service-phospho-A-baumannii-Sk18-S-EtOH-level2_20131007
Scan Method Score m/z
4324 ITMS; CID 45.08 593.77
y7 2+
524.72
b7 2+
520.2
b6 2+
411.69
y6 2+
468.18
b5
725.81
y6-NH3
918.88
y6-H2O
917.84
y6
935.85
y7
1048.44
y7-H2O
[1e4]
Sequence PSMs Fragments Properties
- H L K E H P H K -

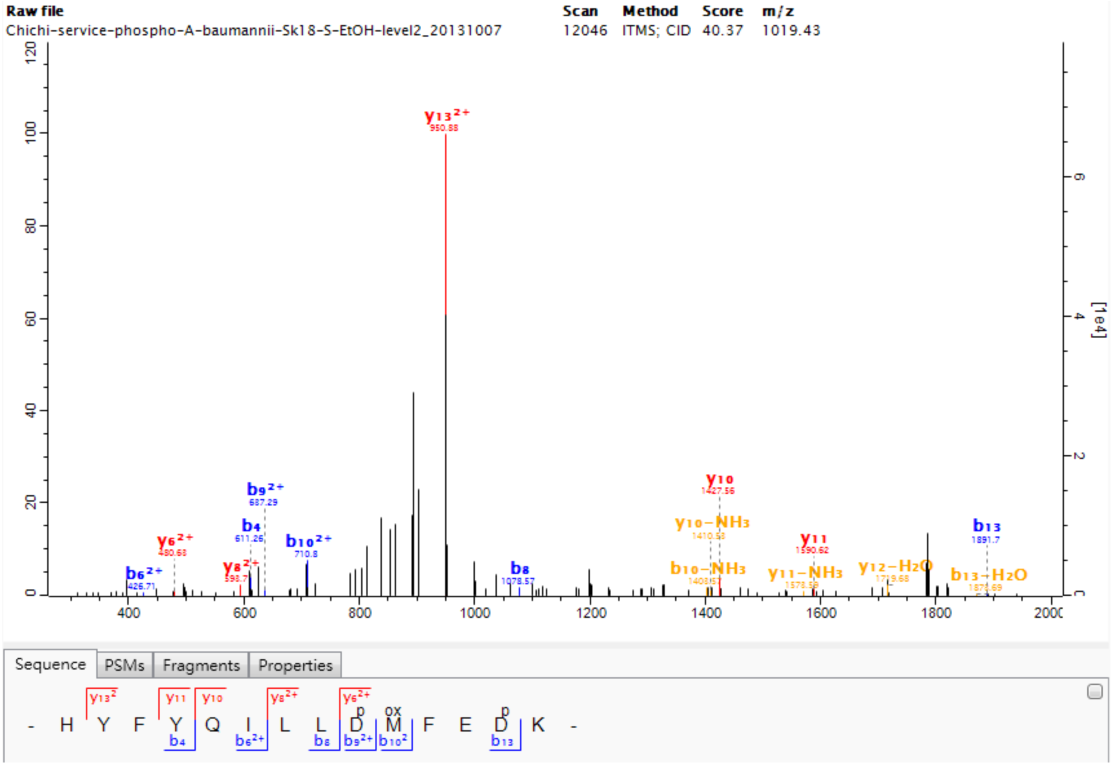
Raw file
Chichi-service-phospho-A-baumannii-Sk18-S-EtOH-level2_20131007
Scan Method Score m/z
12046 ITMS; CID 40.37 1019.43
y13 2+
950.88
b9 2+
687.29
b4
611.26
b10 2+
710.8
y6 2+
480.68
y8 2+
598.7
b6 2+
425.71
b8
1078.57
y10
1427.56
y10-NH3
1410.53
b10-NH3
y11
1590.62
y11-NH3
y12-H2O
1719.68
b13
1891.7
b13-H2O
[1e4]
Sequence PSMs Fragments Properties
- H Y F Y Q I L L D M F E D K -

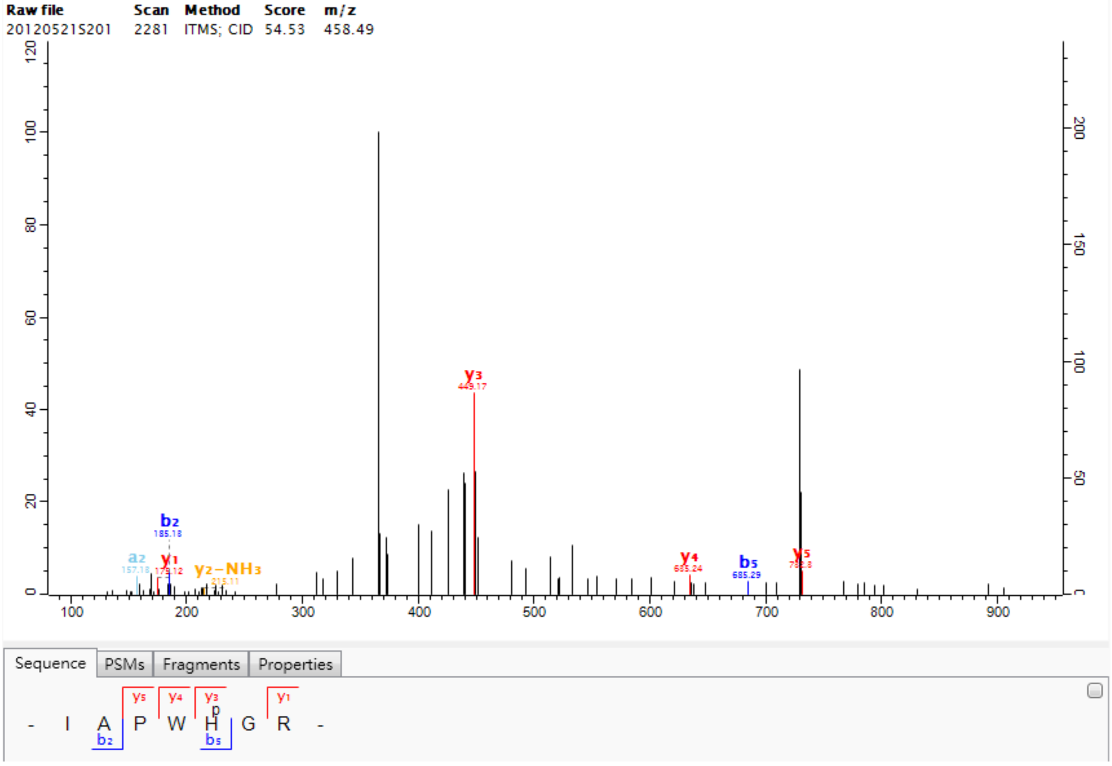
Raw file
20120521S201
Scan
2281
Method
ITMS; CID
Score
54.53
m/z
458.49
y3
449.17
b2
185.18
a2
y1
y2-NH3
y4
b5
685.29
y5
Sequence
PSMs
Fragments
Properties
- I A P W H G R -

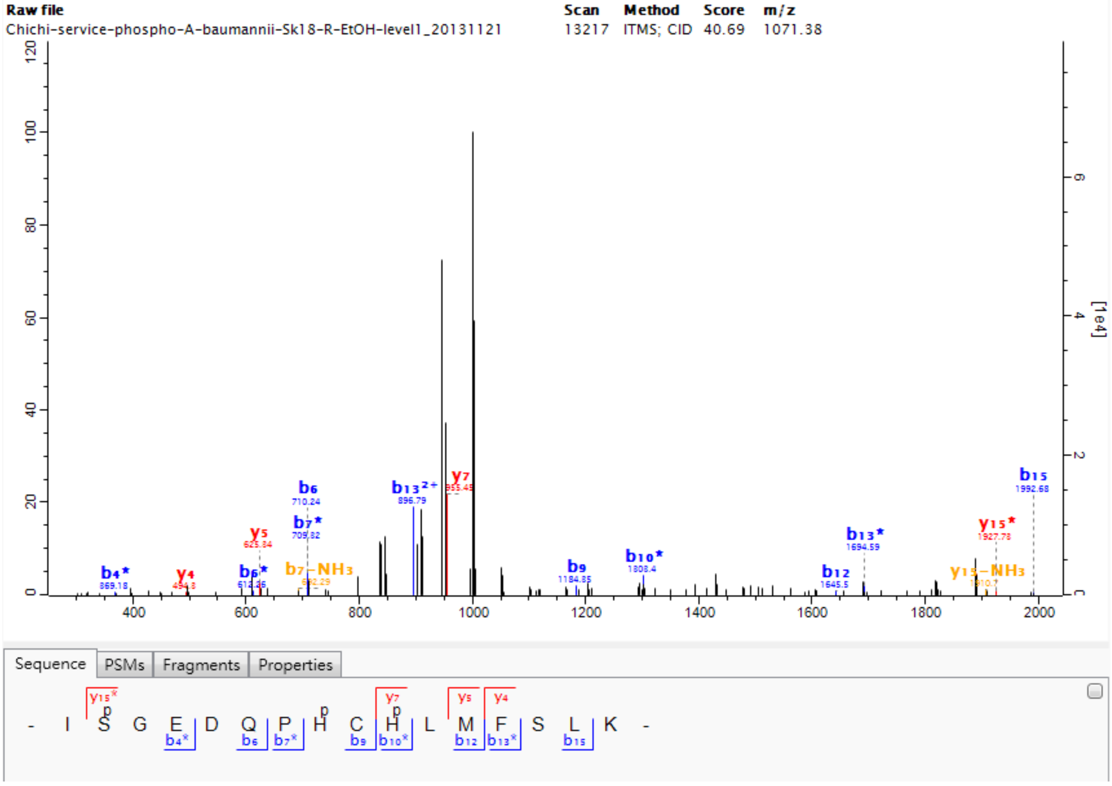
Raw file
Chichi-service-phospho-A-baumannii-Sk18-R-EtOH-level1_20131121
Scan
13217
Method
ITMS; CID
Score
40.69
m/z
1071.38
[1e4]
b4*
y4
y5
b6
710.24
b6*
b7*
b7-NH3
b13 2+
896.79
y7
b9
b10*
b12
b13*
b15
1992.68
y15*
y15-NH3
Sequence
PSMs
Fragments
Properties
- I S G E D Q P H C H L M F S L K -

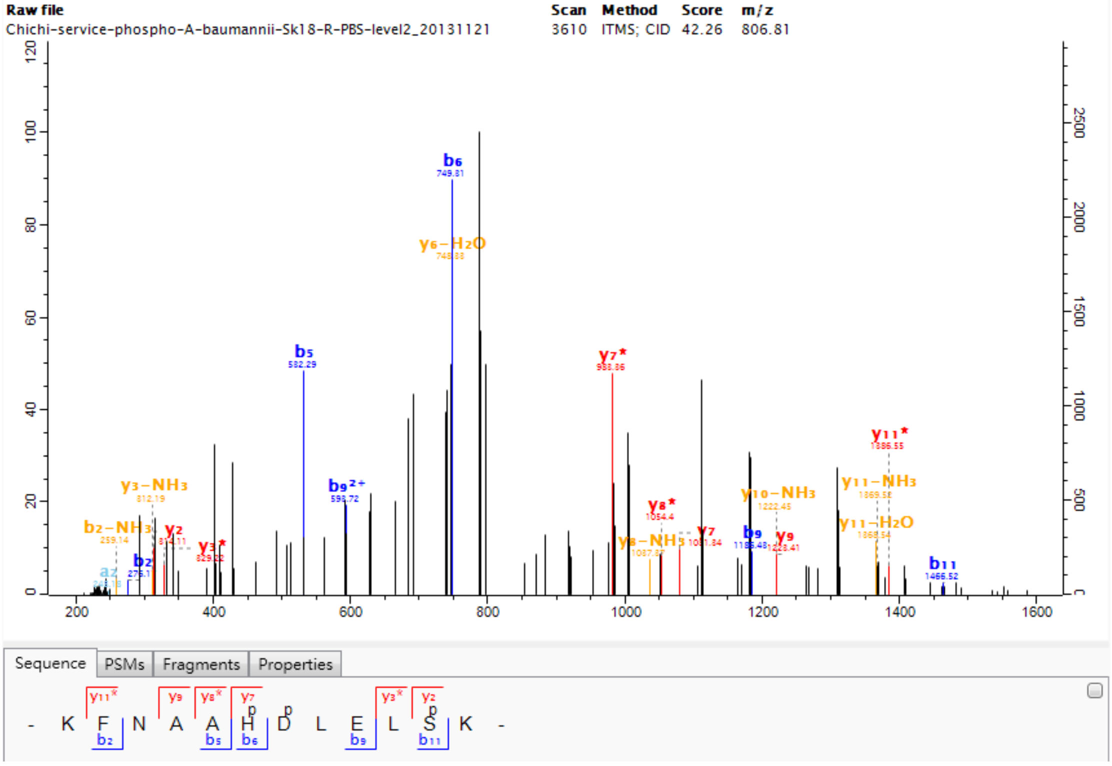
Raw file
Chichi-service-phospho-A-baumannii-Sk18-R-PBS-level2_20131121
Scan Method Score m/z
3610 ITMS; CID 42.26 806.81
Sequence
PSMs
Fragments
Properties
- K F N A A H D L E L S K -

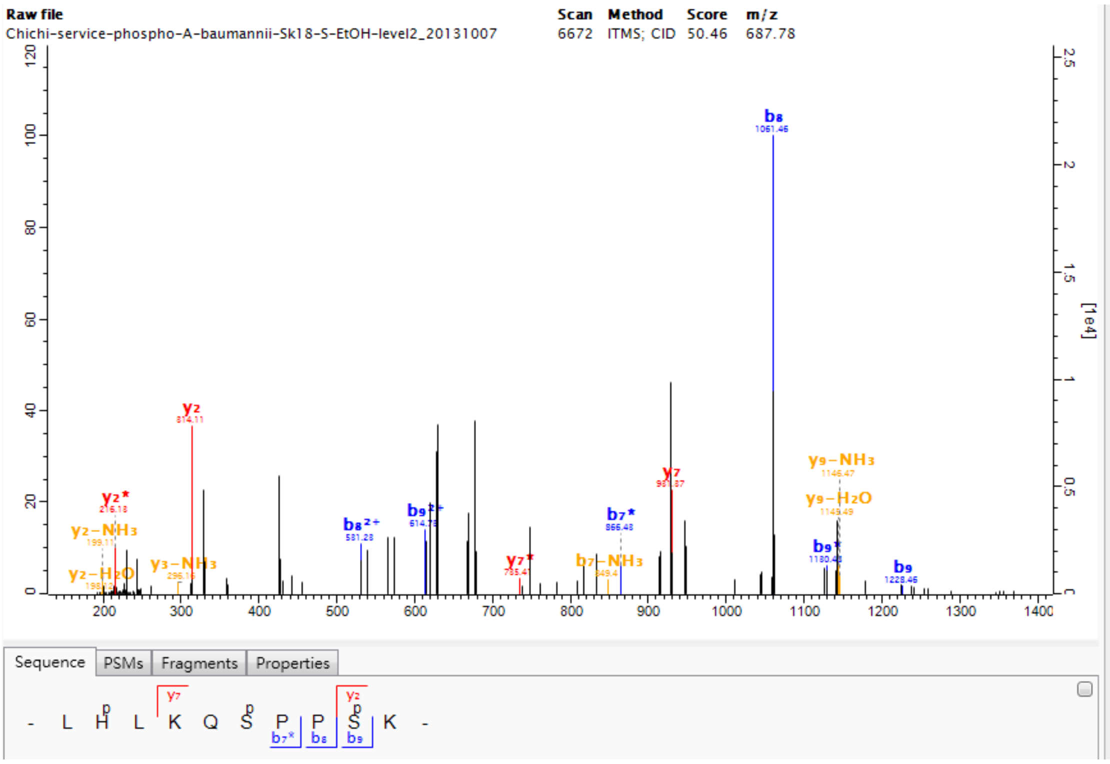
Raw file
Chichi-service-phospho-A-baumannii-Sk18-S-EtOH-level2_20131007
Scan Method Score m/z
6672 ITMS; CID 50.46 687.78
Sequence
PSMs
Fragments
Properties
- L H L K Q S P P S K -

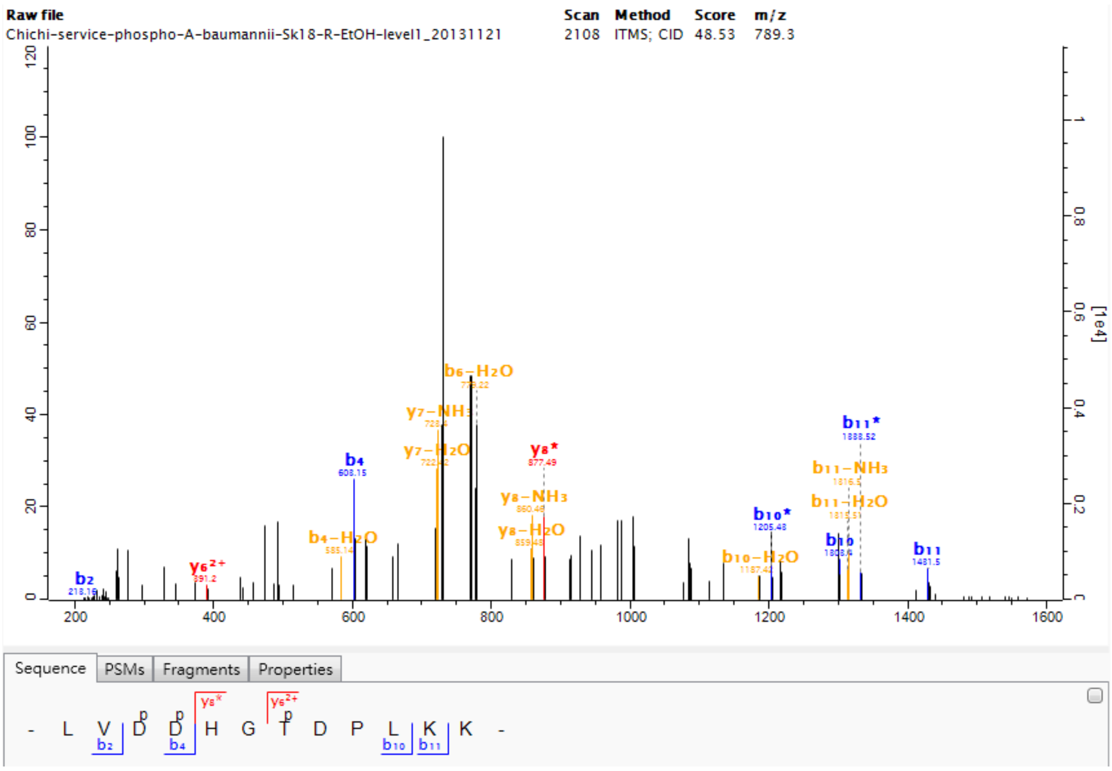
Raw file
Chichi-service-phospho-A-baumannii-Sk18-R-EtOH-level1_20131121
Scan Method Score m/z
2108 ITMS; CID 48.53 789.3
[1e4]
b2
y6 2+
b4
b4-H2O
y7-NH3
y7-H2O
b6-H2O
y8*
y8-NH3
y8-H2O
b10-H2O
b10*
b10
b11-NH3
b11-H2O
b11*
b11
Sequence PSMs Fragments Properties
- L V D D H G T D P L K K -

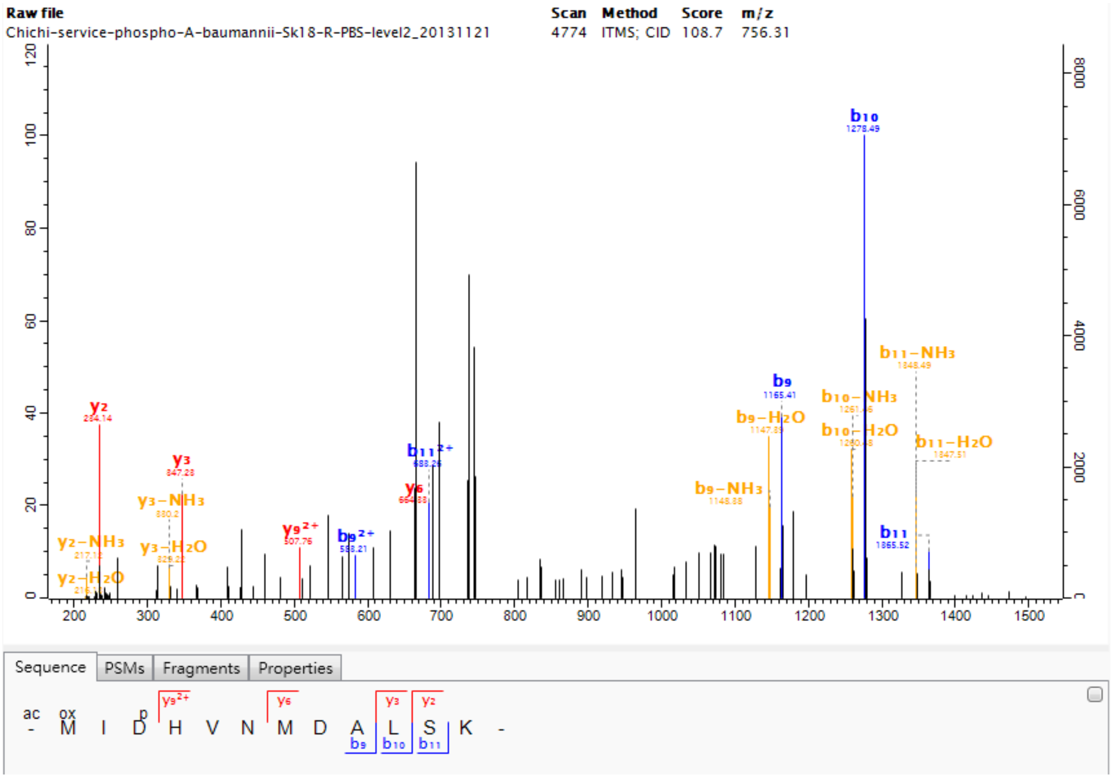
Raw file
Chichi-service-phospho-A-baumannii-Sk18-R-PBS-level2_20131121
Scan Method Score m/z
4774 ITMS; CID 108.7 756.31
y2
y2-NH3
y2-H2O
y3
y3-NH3
y3-H2O
y9 2+
b9 2+
b11 2+
y6
b9-NH3
b9-H2O
b9
b10-NH3
b10-H2O
b10
b11-NH3
b11-H2O
b11
Sequence PSMs Fragments Properties
ac ox
- M I D H V N M D A L S K -

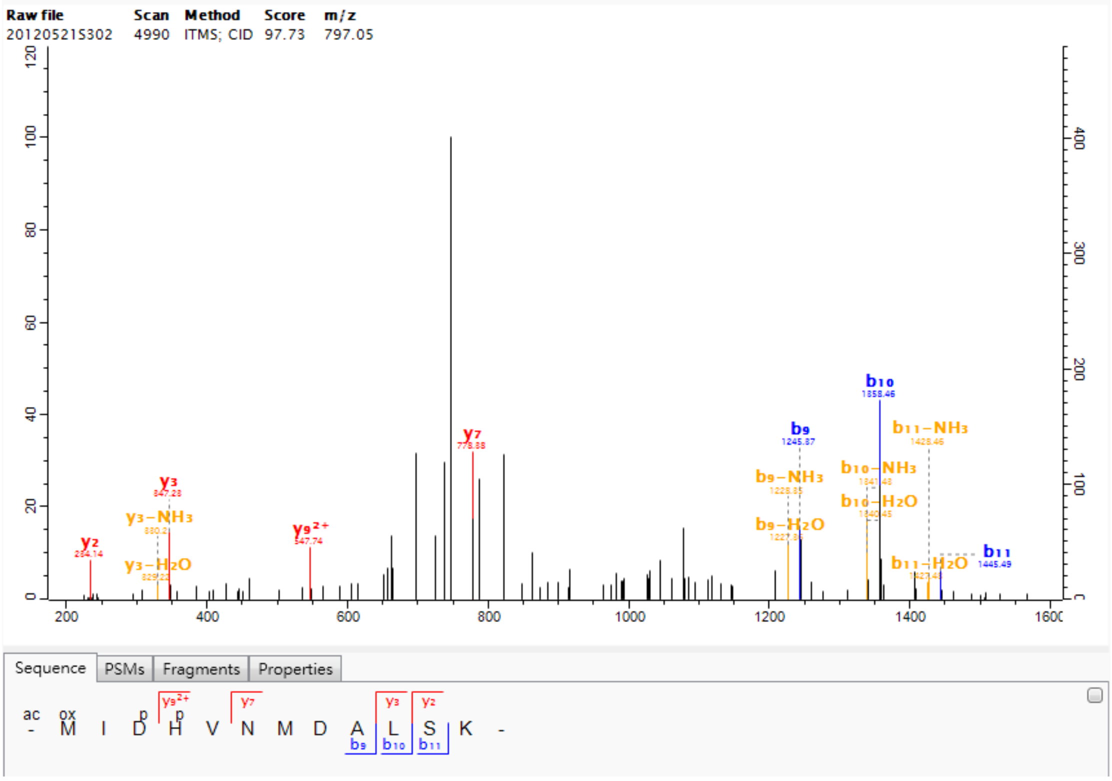
Raw file Scan Method Score m/z
20120521S302 4990 ITMS; CID 97.73 797.05
y2
y3
y3−NH3
y3−H2O
y9 2+
y7
b9
b9−NH3
b9−H2O
b10
b10−NH3
b10−H2O
b11−NH3
b11−H2O
b11
Sequence PSMs Fragments Properties
ac ox p p
- M I D H V N M D A L S K -

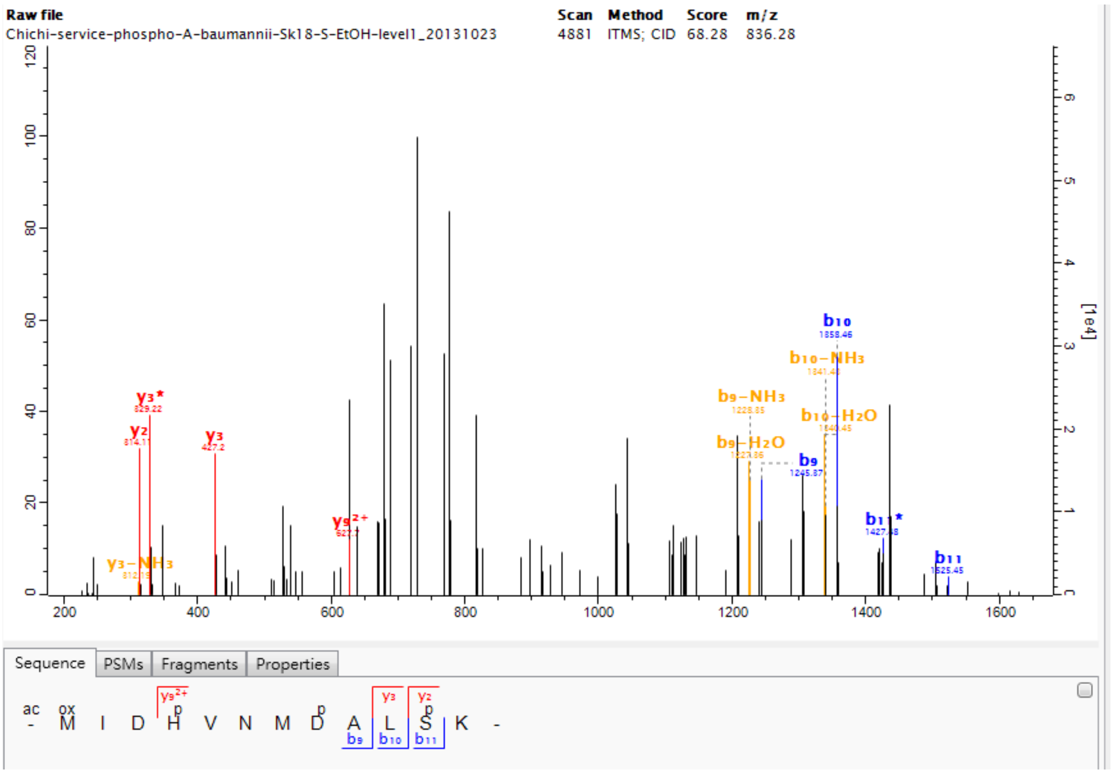
Raw file Scan Method Score m/z
Chichi-service-phospho-A-baumannii-Sk18-S-EtOH-level1_20131023 4881 ITMS; CID 68.28 836.28
[1e4]
y3*
y2
y3
y3−NH3
y9 2+
b9−NH3
b9−H2O
b9
b10
b10−NH3
b10−H2O
b11*
b11
Sequence PSMs Fragments Properties
ac ox p p p
- M I D H V N M D A L S K -

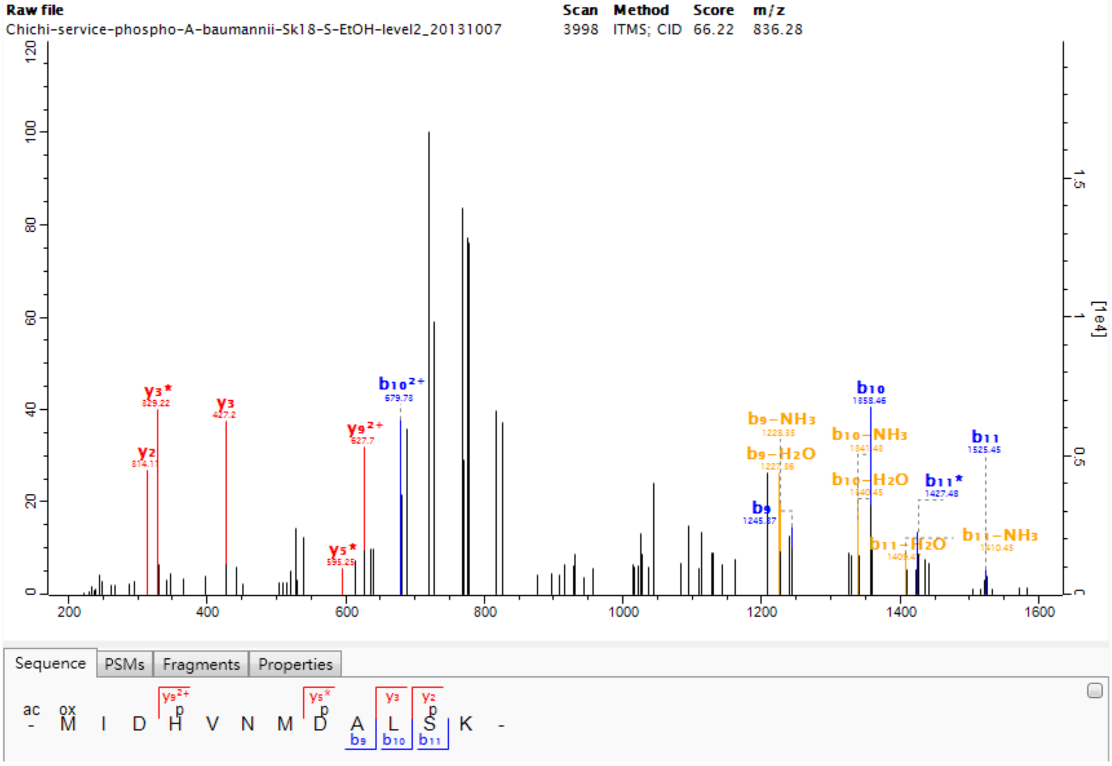
Raw file
Chichi-service-phospho-A-baumannii-Sk18-S-EtOH-level2_20131007
Scan Method Score m/z
3998 ITMS; CID 66.22 836.28
[1e4]
Sequence PSMs Fragments Properties
ac - M I D H V N M D A L S K -

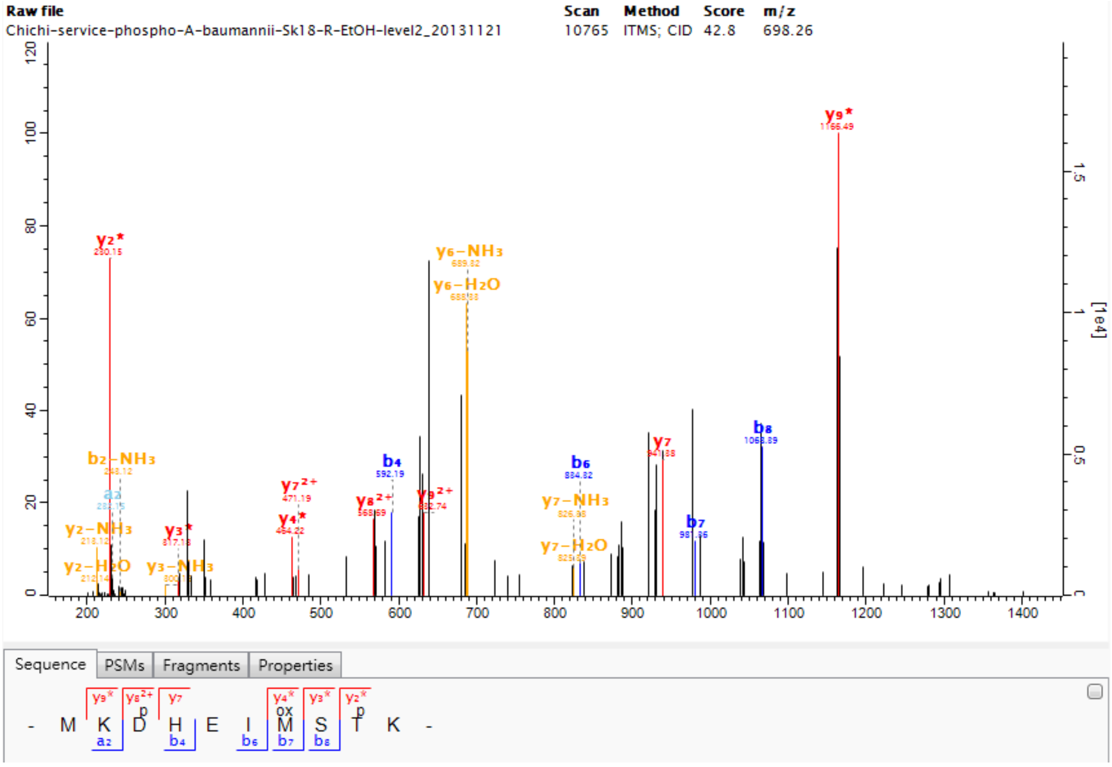
Raw file
Chichi-service-phospho-A-baumannii-Sk18-R-EtOH-level2_20131121
Scan Method Score m/z
10765 ITMS; CID 42.8 698.26
[1e4]
Sequence PSMs Fragments Properties
- M K D H E I M S T K -

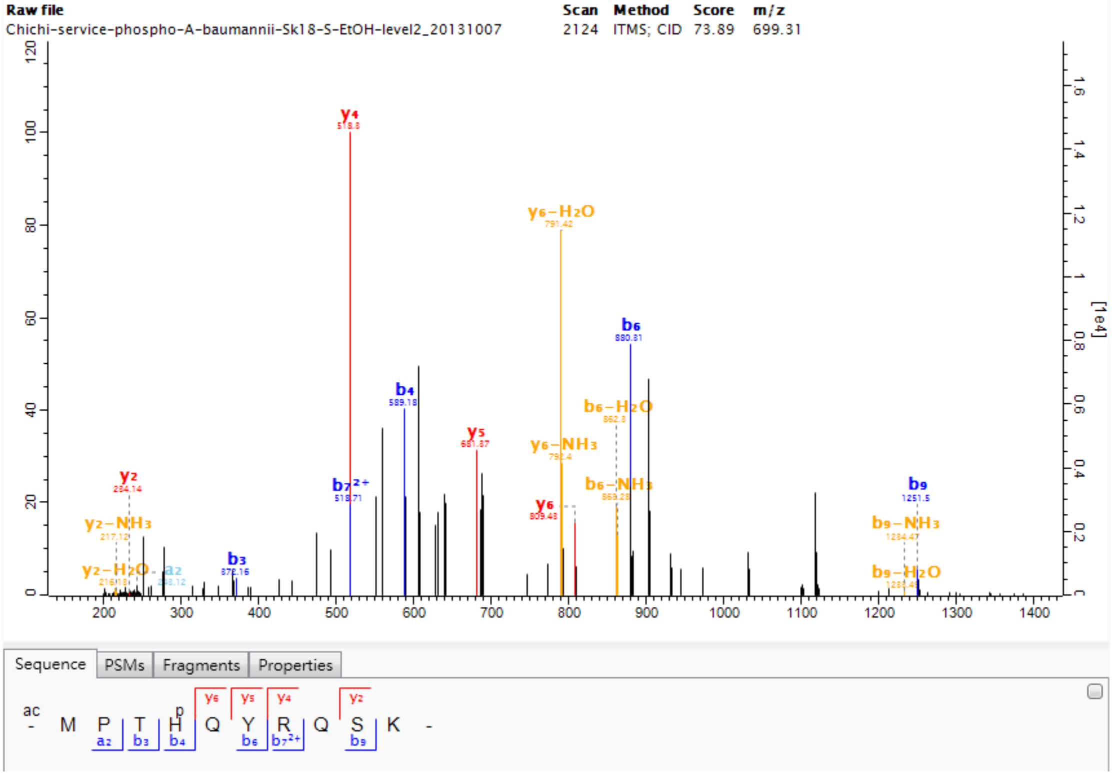
Raw file
Chichi-service-phospho-A-baumannii-Sk18-S-EtOH-level2_20131007
Scan Method Score m/z
2124 ITMS; CID 73.89 699.31
[1e4]
Sequence PSMs Fragments Properties
ac - M P T H Q Y R Q S K -

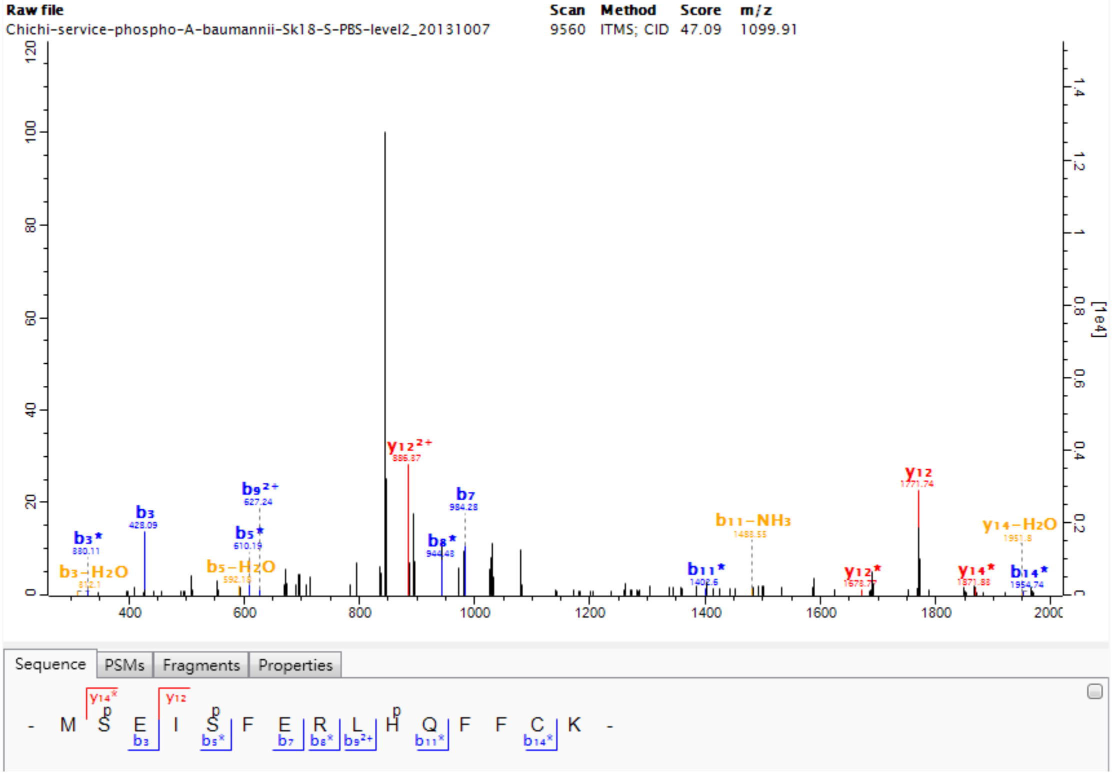
Raw file
Chichi-service-phospho-A-baumannii-Sk18-S-PBS-level2_20131007
Scan Method Score m/z
9560 ITMS; CID 47.09 1099.91
[1e4]
Sequence PSMs Fragments Properties
- M S E I S F E R L H Q F F C K -

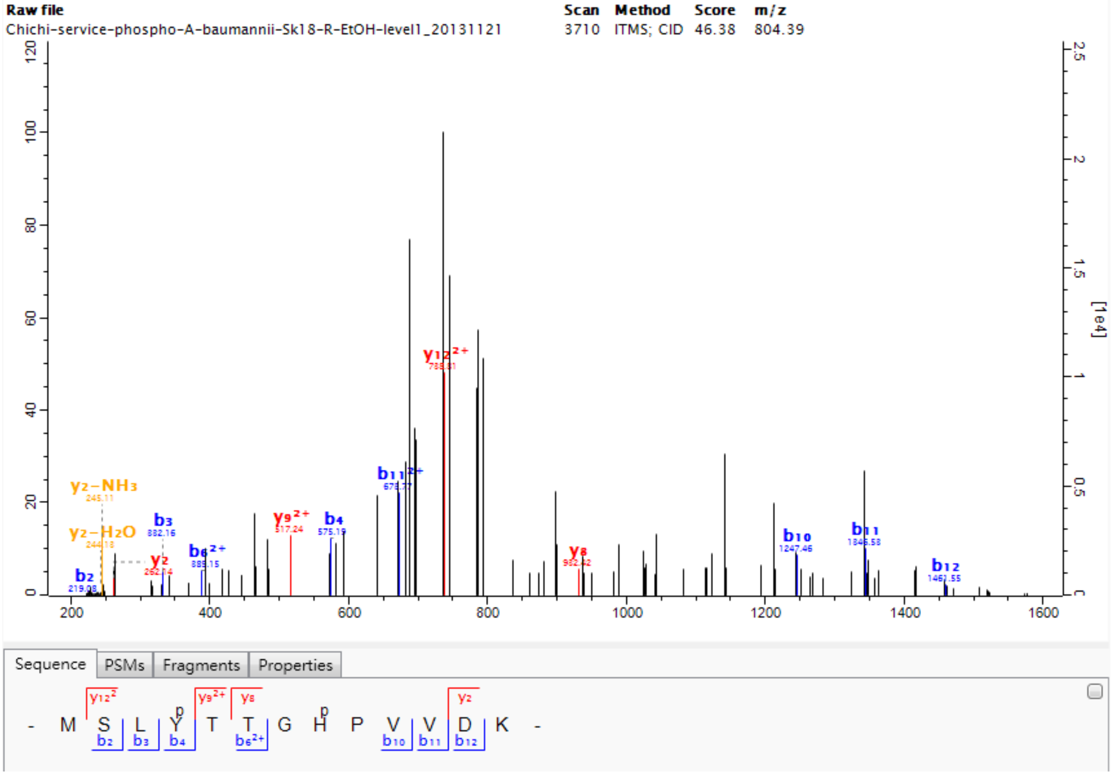
Raw file
Chichi-service-phospho-A-baumannii-Sk18-R-EtOH-level1_20131121
Scan
3710
Method
ITMS; CID
Score
46.38
m/z
804.39
[1e4]
Sequence
PSMs
Fragments
Properties
- M S L Y T T G H P V V D K -

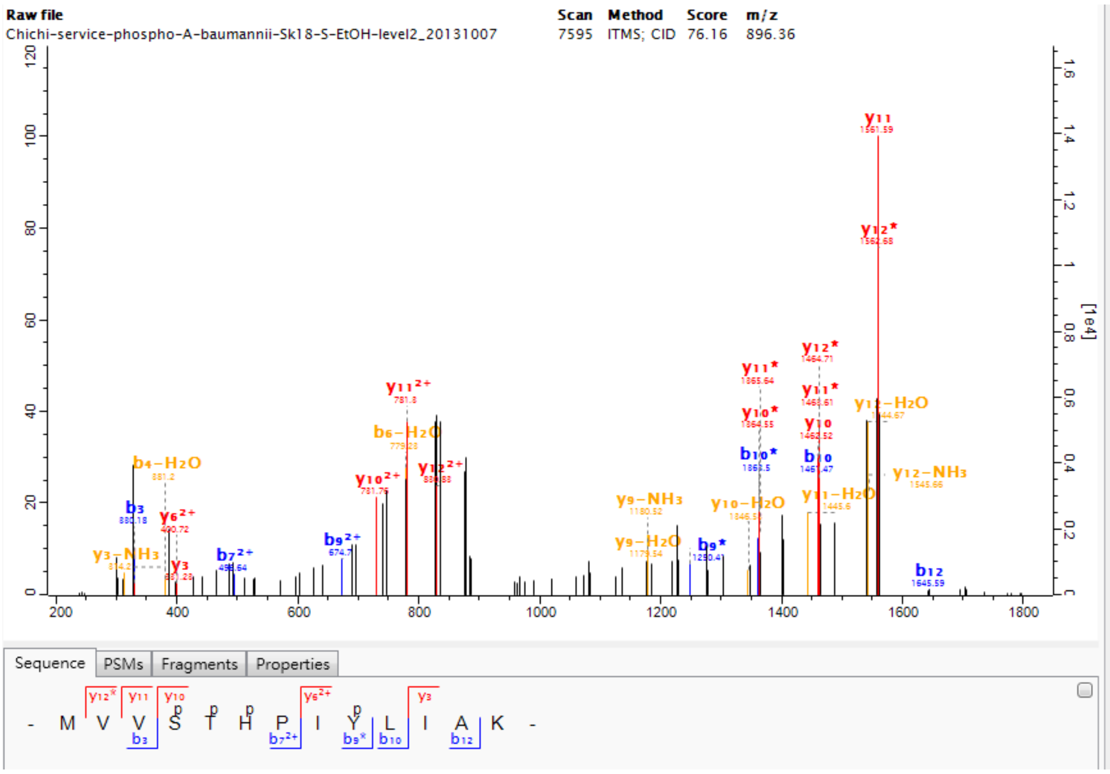
Raw file
Chichi-service-phospho-A-baumannii-Sk18-S-EtOH-level2_20131007
Scan
7595
Method
ITMS; CID
Score
76.16
m/z
896.36
[1e4]
Sequence
PSMs
Fragments
Properties
- M V V S T H P I Y L I A K -

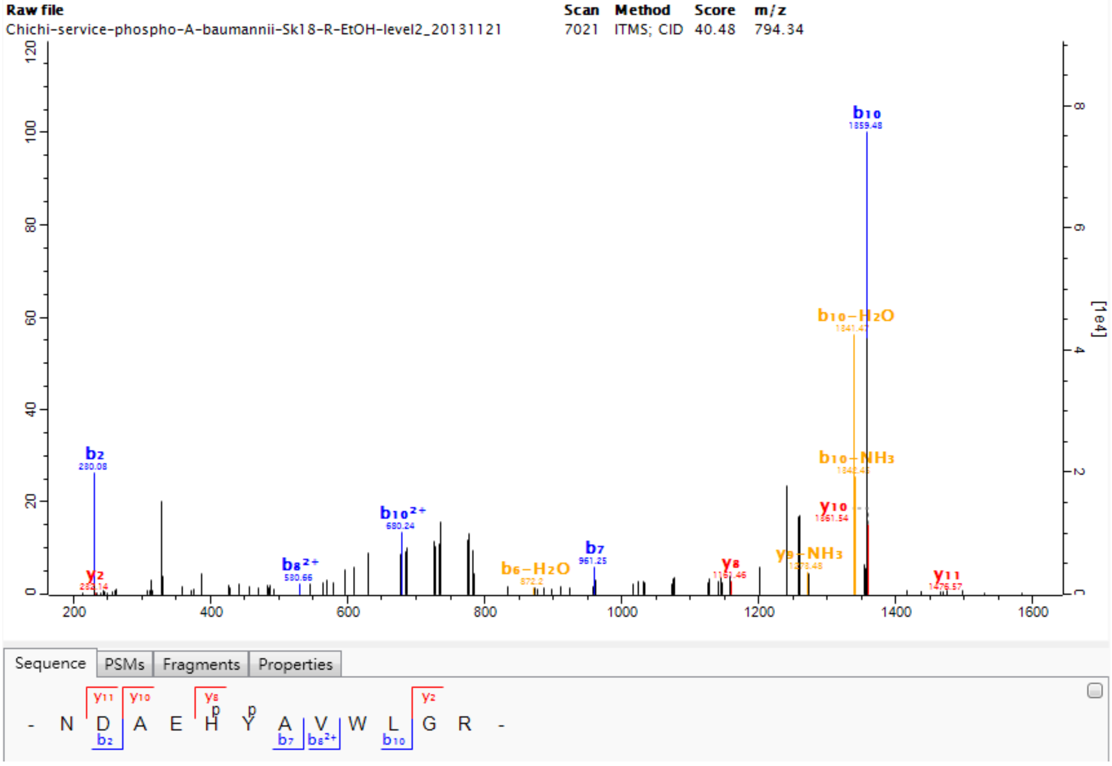
Raw file
Chichi-service-phospho-A-baumannii-Sk18-R-EtOH-level2_20131121
Scan Method Score m/z
7021 ITMS; CID 40.48 794.34
b10
1359.48
b10-H2O
1341.47
b10-NH3
y10
y9-NH3
y8
y11
b2
230.08
y2
b10 2+
680.24
b8 2+
580.66
b6-H2O
872.2
b7
961.25
[1e4]
Sequence PSMs Fragments Properties
- N D A E H Y A V W L G R -

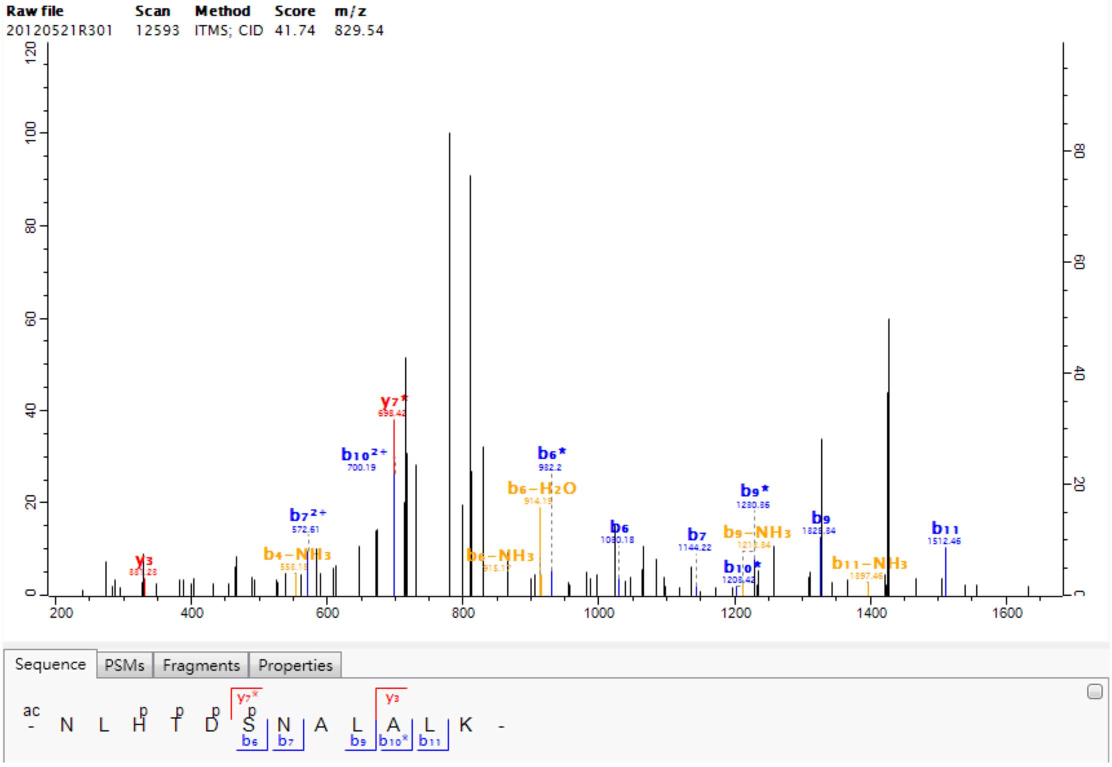
Raw file
20120521R301
Scan Method Score m/z
12593 ITMS; CID 41.74 829.54
y7*
698.42
b10 2+
700.19
b7 2+
572.61
y3
b4-NH3
b6*
982.2
b6-H2O
914.19
b6-NH3
b6
1030.18
b7
1144.22
b9*
1280.86
b9-NH3
b9
b10*
b11
1512.46
b11-NH3
1397.46
Sequence PSMs Fragments Properties
ac
- N L H T D S N A L A L K -

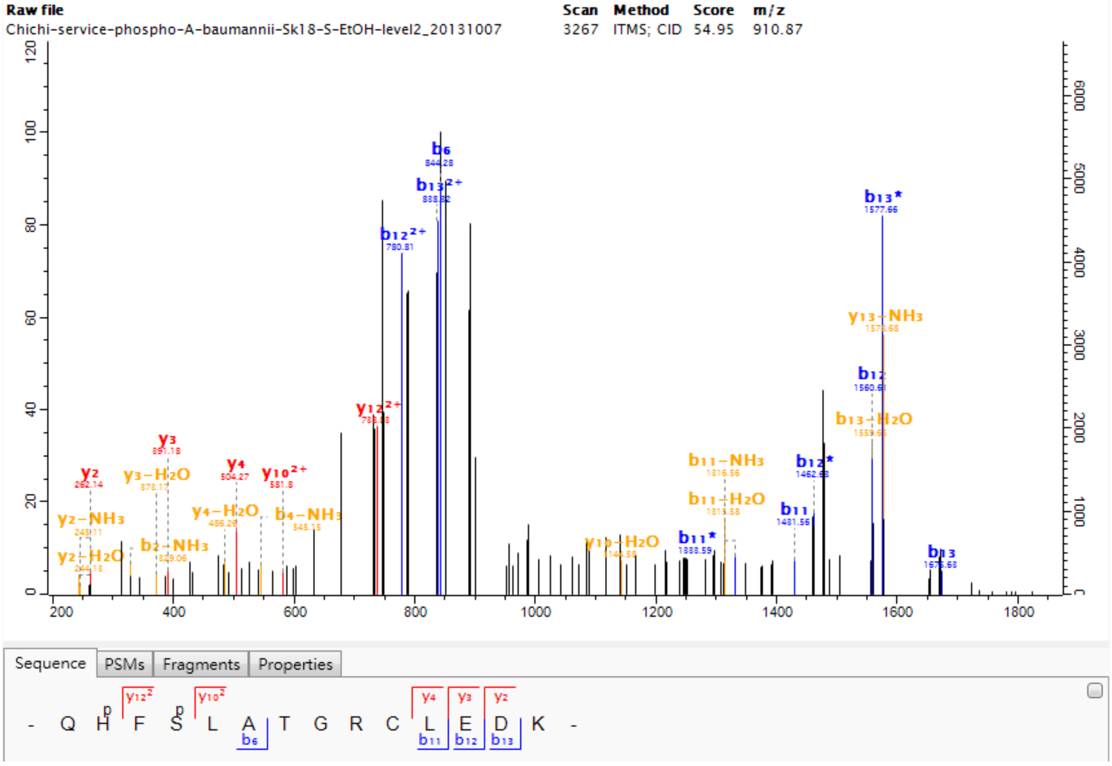
Raw file
Chichi-service-phospho-A-baumannii-Sk18-S-EtOH-level2_20131007
Scan Method Score m/z
3267 ITMS; CID 54.95 910.87
Sequence PSMs Fragments Properties
- Q H F S L A T G R C L E D K -

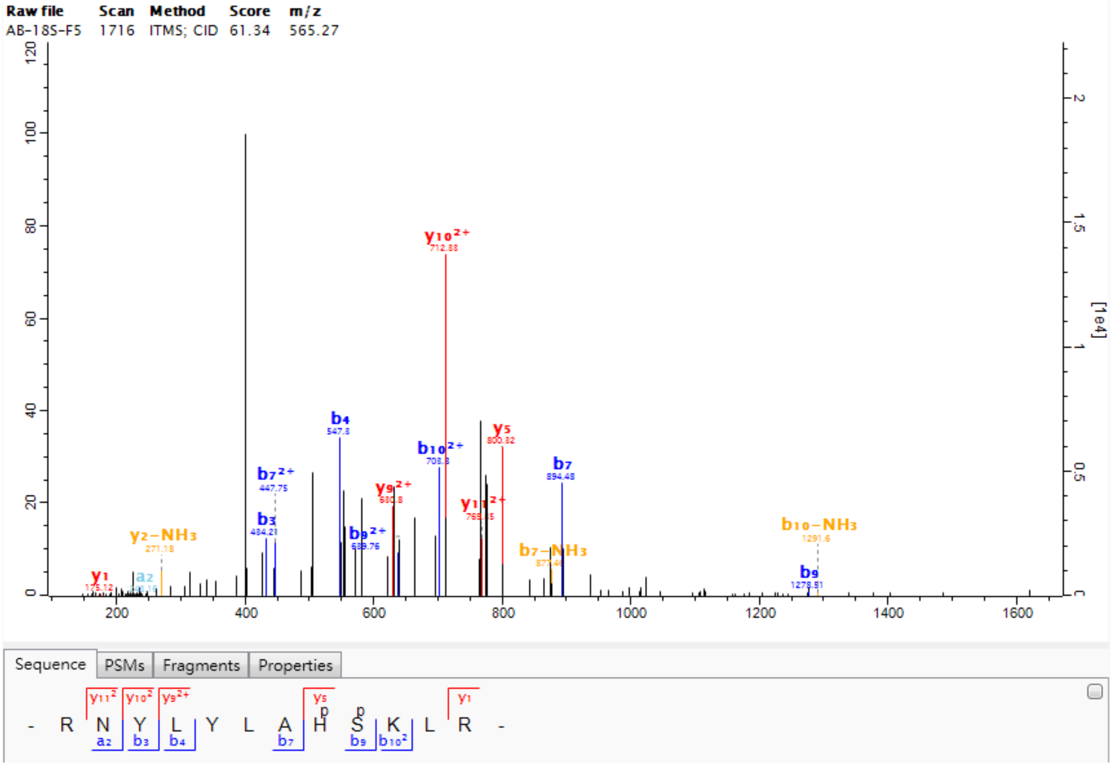
Raw file Scan Method Score m/z
AB-18S-F5 1716 ITMS; CID 61.34 565.27
Sequence PSMs Fragments Properties
- R N Y L Y L A H S K L R -

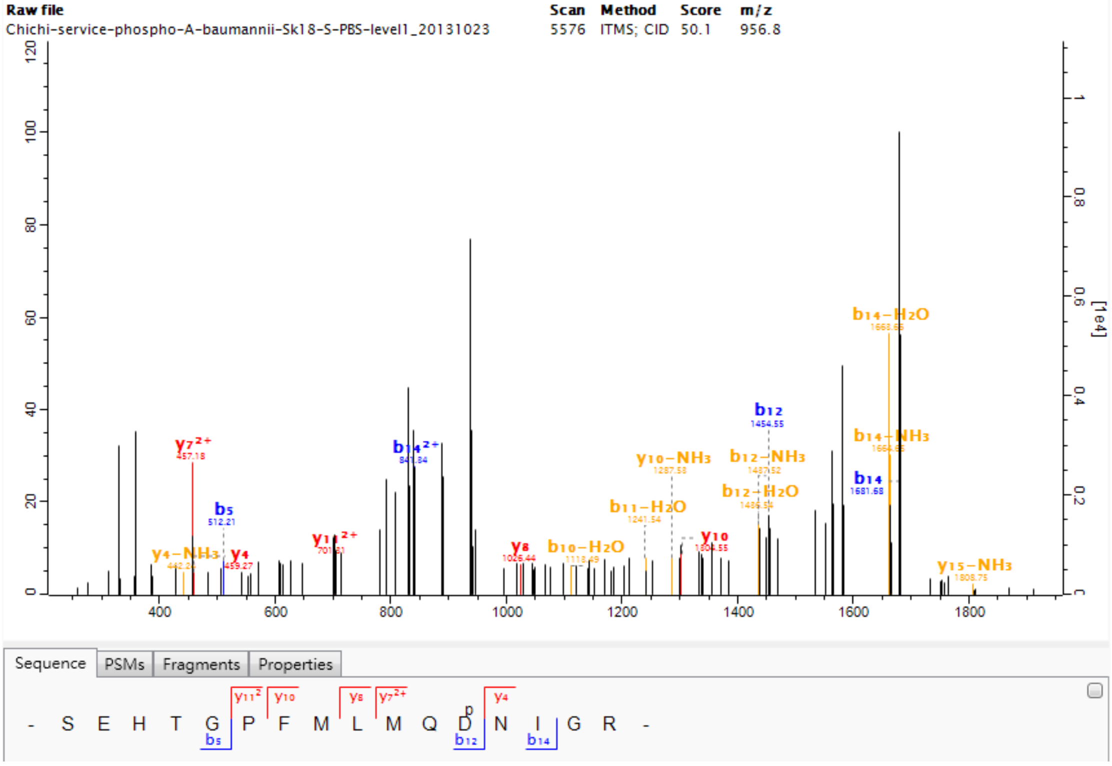
Raw file
Chichi-service-phospho-A-baumannii-Sk18-S-PBS-level1_20131023
Scan 5576
Method ITMS; CID
Score 50.1
m/z 956.8
[1e4]
Sequence
PSMs
Fragments
Properties
- S E H T G P F M L M Q D N I G R -

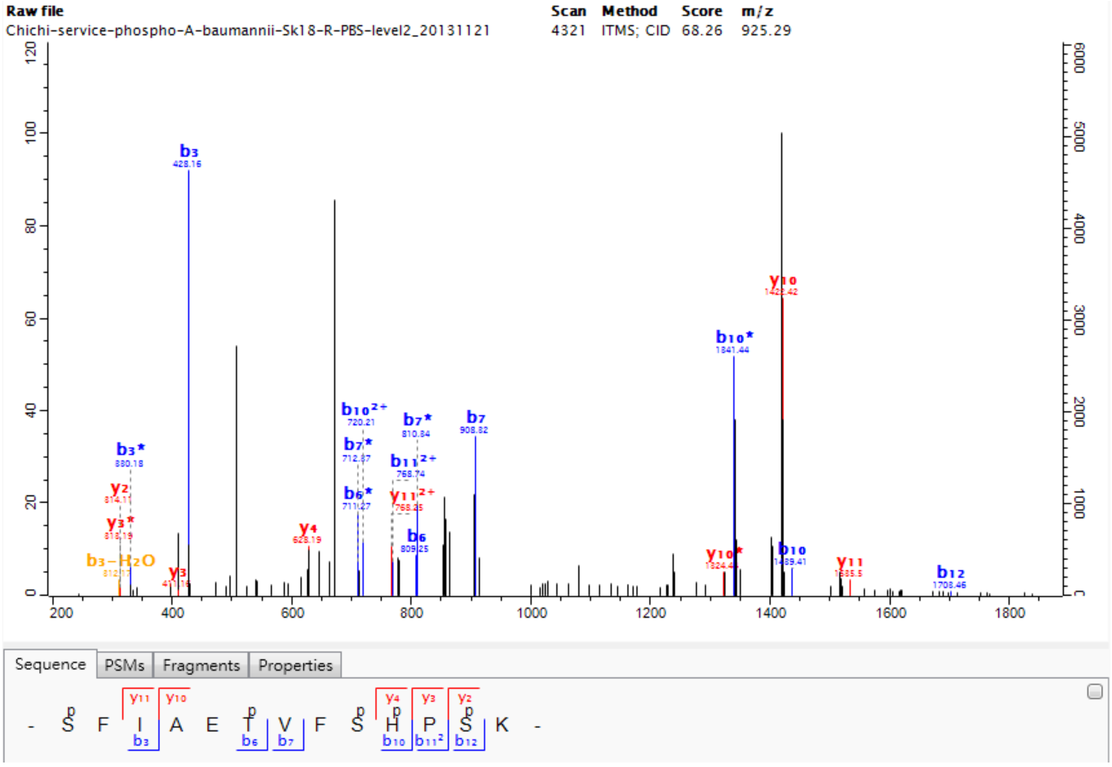
Raw file
Chichi-service-phospho-A-baumannii-Sk18-R-PBS-level2_20131121
Scan 4321
Method ITMS; CID
Score 68.26
m/z 925.29
Sequence
PSMs
Fragments
Properties
- S F I A E T V F S H P S K -

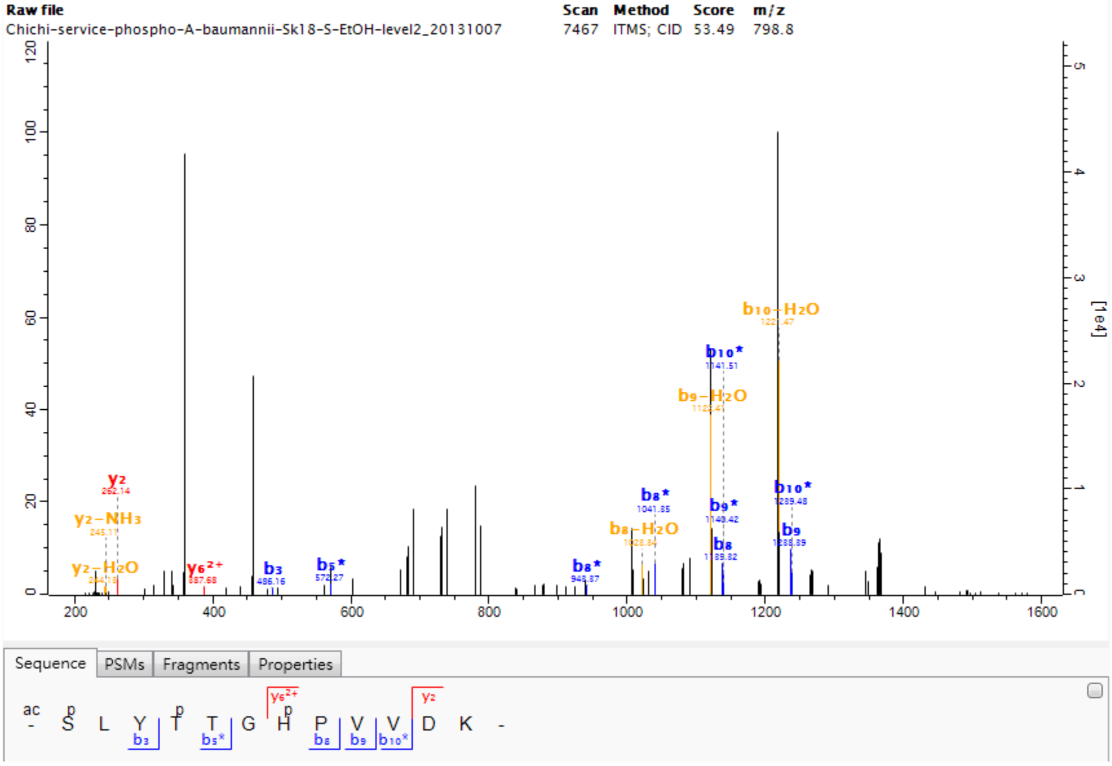
Raw file
Chichi-service-phospho-A-baumannii-Sk18-S-EtOH-level2_20131007
Scan Method Score m/z
7467 ITMS; CID 53.49 798.8
[1e4]
Sequence
PSMs
Fragments
Properties
ac - S L Y T T G H P V V D K -

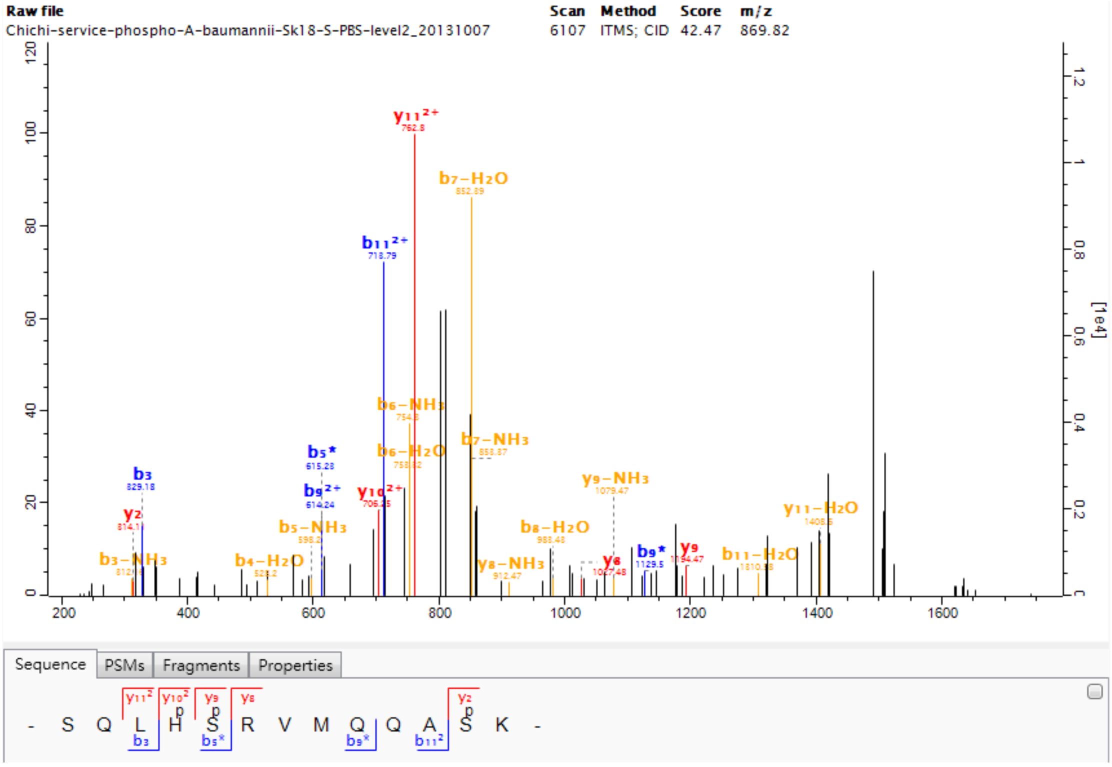
Raw file
Chichi-service-phospho-A-baumannii-Sk18-S-PBS-level2_20131007
Scan Method Score m/z
6107 ITMS; CID 42.47 869.82
[1e4]
Sequence
PSMs
Fragments
Properties
- S Q L H S R V M Q Q A S K -

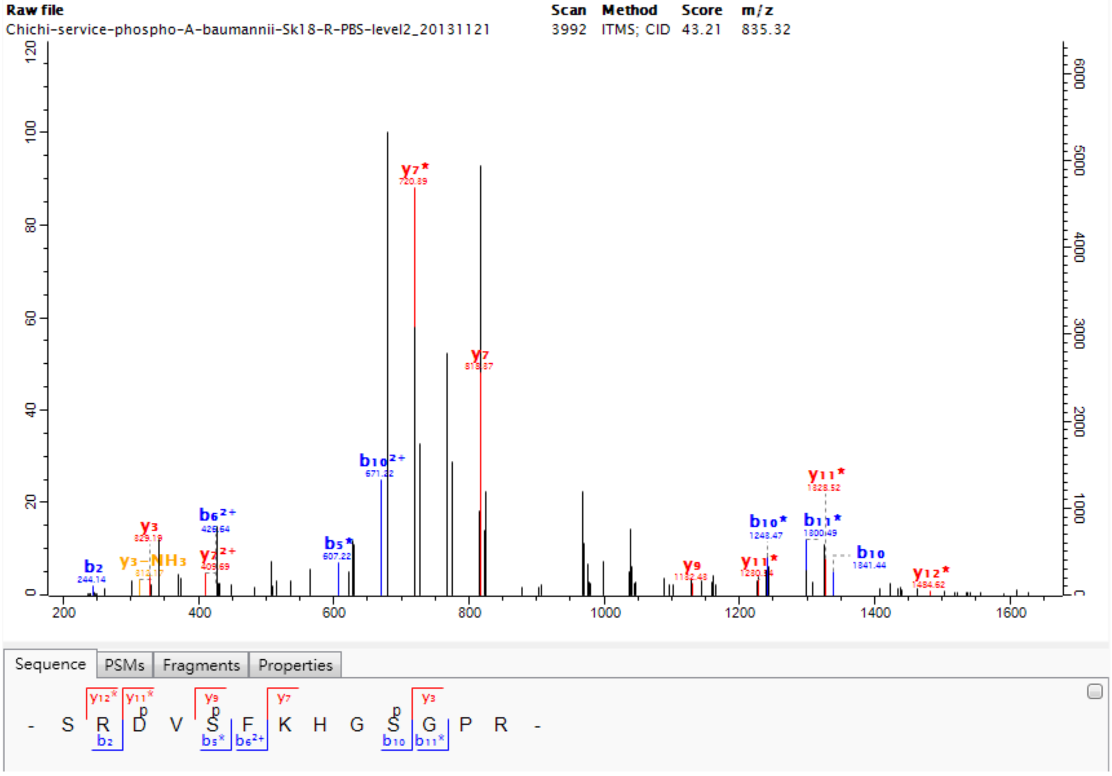
Raw file
Chichi-service-phospho-A-baumannii-Sk18-R-PBS-level2_20131121
Scan Method Score m/z
3992 ITMS; CID 43.21 835.32
Sequence
PSMs
Fragments
Properties
- S R D V S F K H G S G P R -

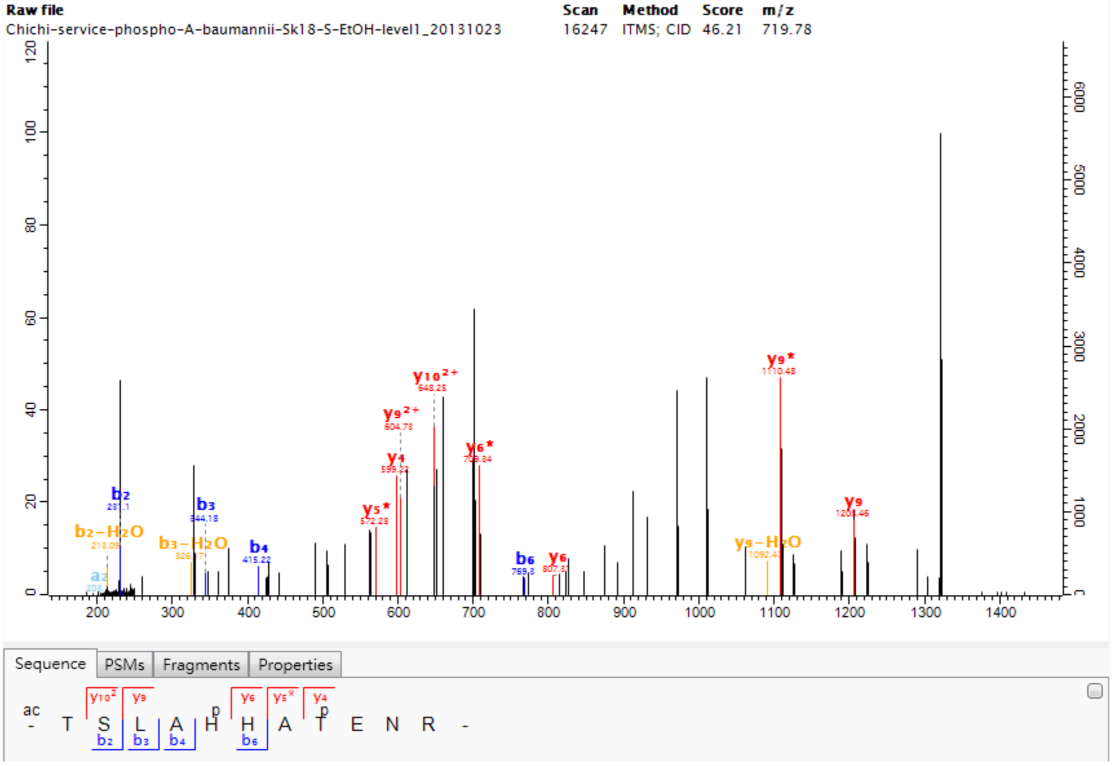
Raw file
Chichi-service-phospho-A-baumannii-Sk18-S-EtOH-level1_20131023
Scan Method Score m/z
16247 ITMS; CID 46.21 719.78
Sequence
PSMs
Fragments
Properties
ac - T S L A H H A T E N R -

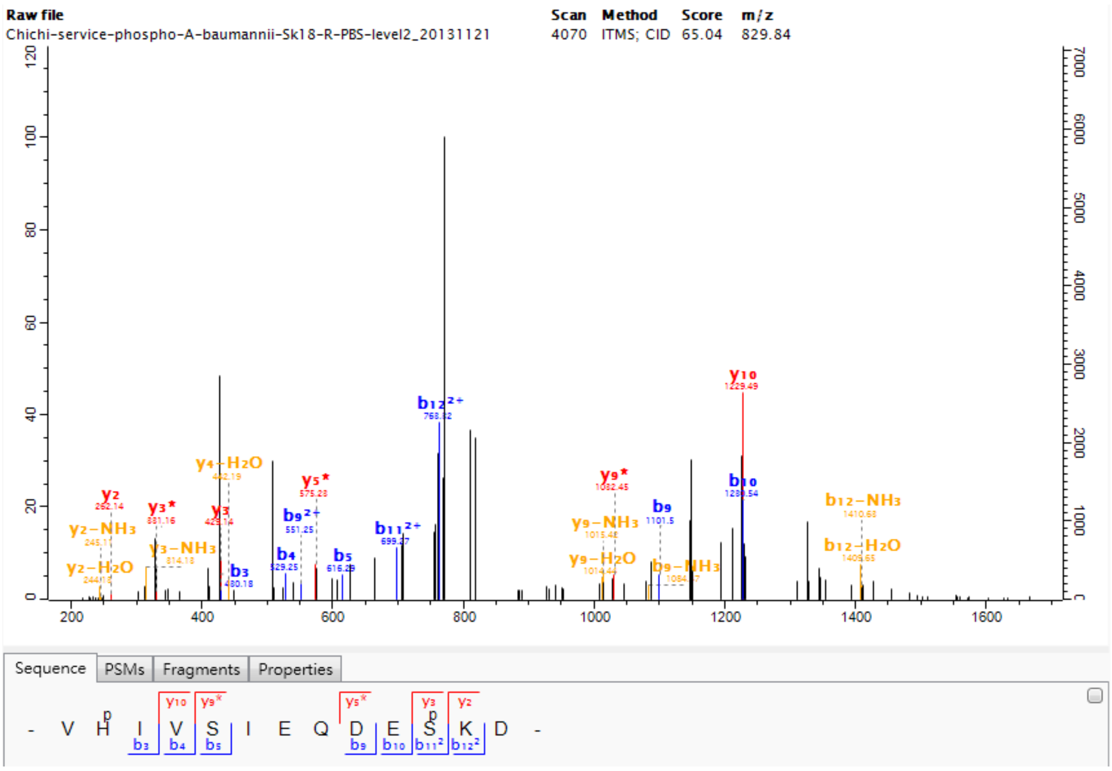
Raw file
Chichi-service-phospho-A-baumannii-Sk18-R-PBS-level2_20131121
Scan Method Score m/z
4070 ITMS; CID 65.04 829.84
Sequence
PSMs
Fragments
Properties
- V H I V S I E Q D E S K D -

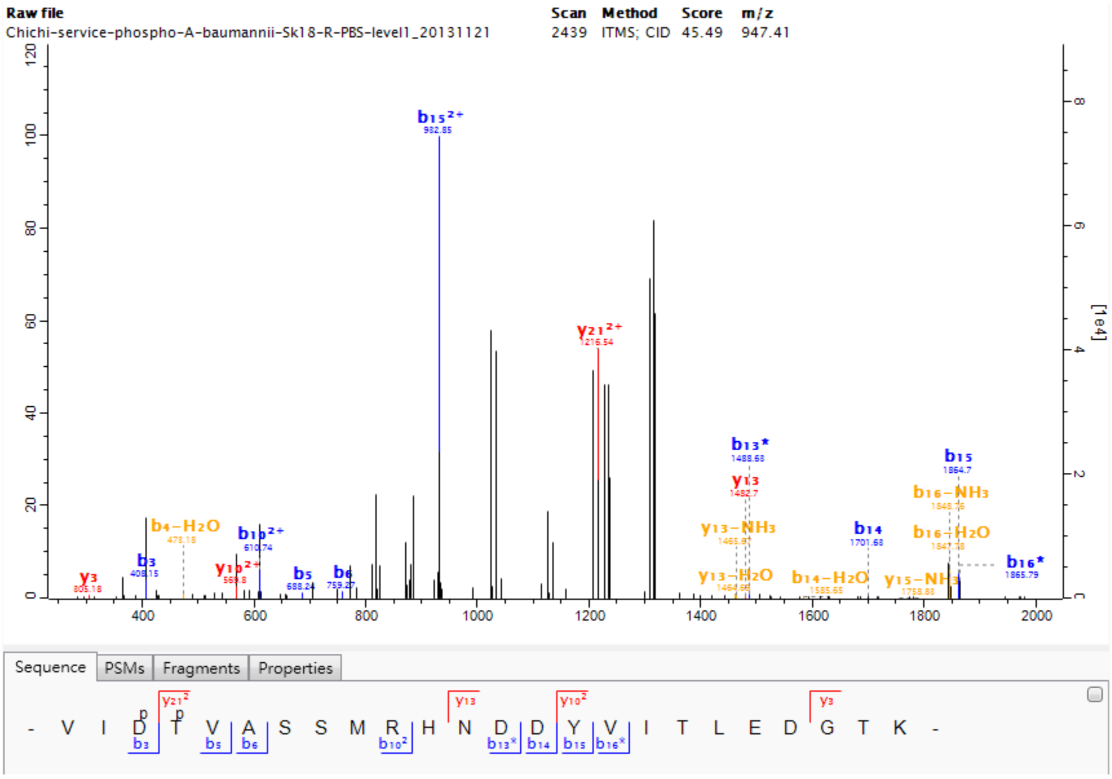
Raw file
Chichi-service-phospho-A-baumannii-Sk18-R-PBS-level1_20131121
Scan Method Score m/z
2439 ITMS; CID 45.49 947.41
Sequence
PSMs
Fragments
Properties
- V I D T V A S S M R H N D D Y V I T L E D G T K -

Helicobacter pylori (pathogenic bacterium)

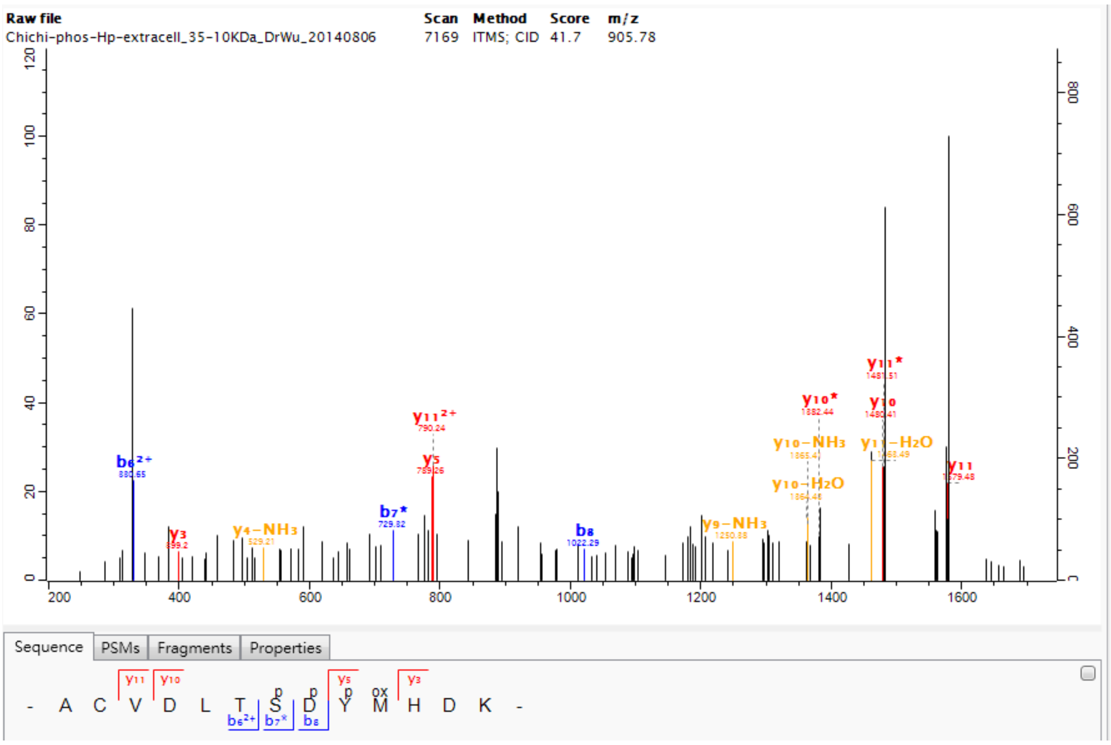

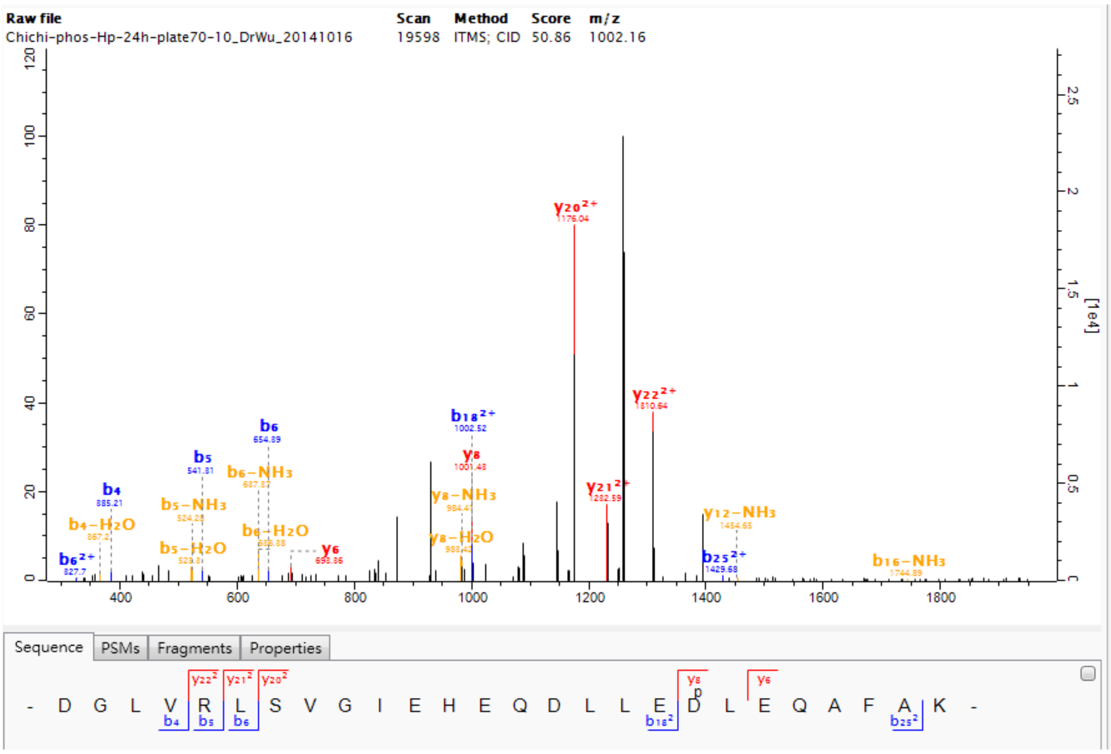

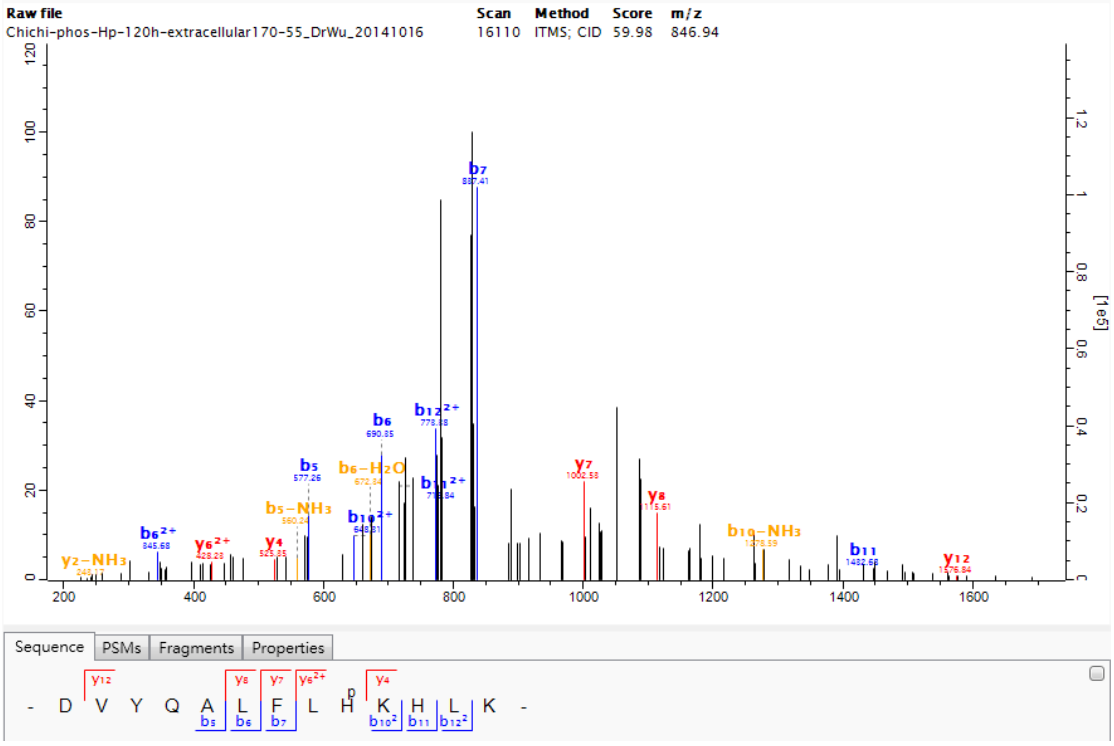
Raw file
Chichi-phos-Hp-120h-extracellular170-55_DrWu_20141016
Scan Method Score m/z
16110 ITMS; CID 59.98 846.94
[1e5]
Sequence PSMs Fragments Properties
- D V Y Q A L F L H K H L K -

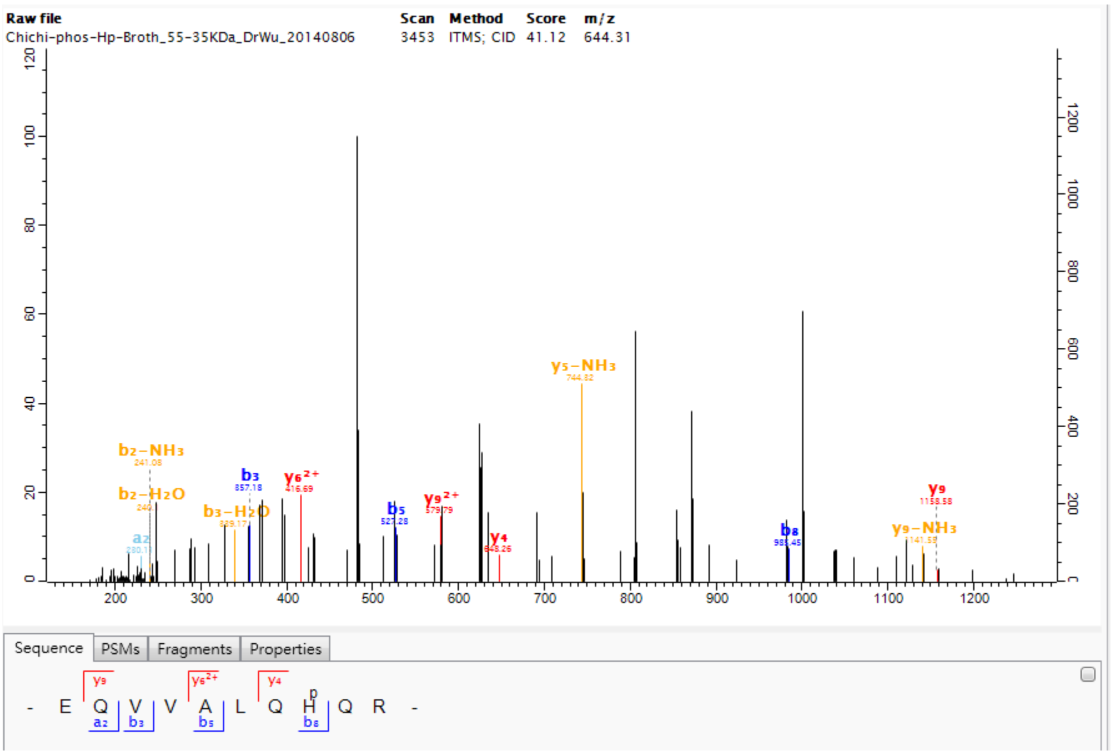
Raw file
Chichi-phos-Hp-Broth_55-35KDa_DrWu_20140806
Scan Method Score m/z
3453 ITMS; CID 41.12 644.31
Sequence PSMs Fragments Properties
- E Q V V A L Q H Q R -

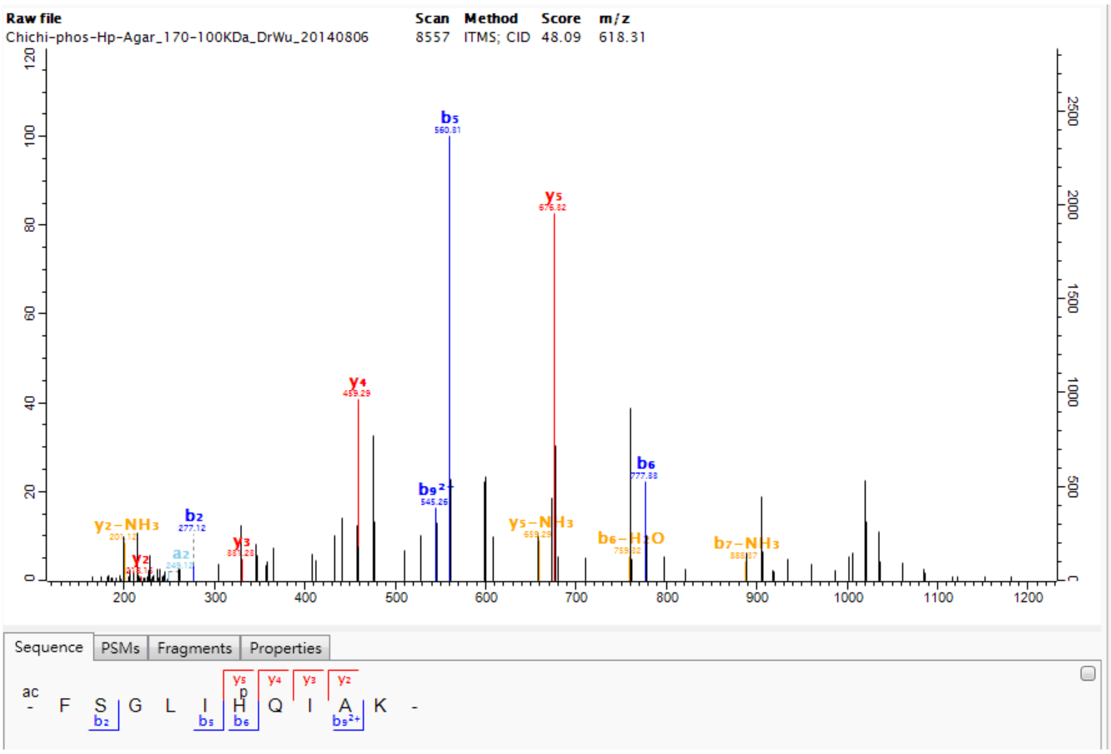
Raw file
Chichi-phos-Hp-Agar_170-100KDa_DrWu_20140806
Scan Method Score m/z
8557 ITMS; CID 48.09 618.31
y2-NH3
b2
a2
y2
y3
y4
b9²+
b5
y5-NH3
y5
b6-H2O
b6
b7-NH3
Sequence PSMs Fragments Properties
ac - F S G L I H Q I A K -

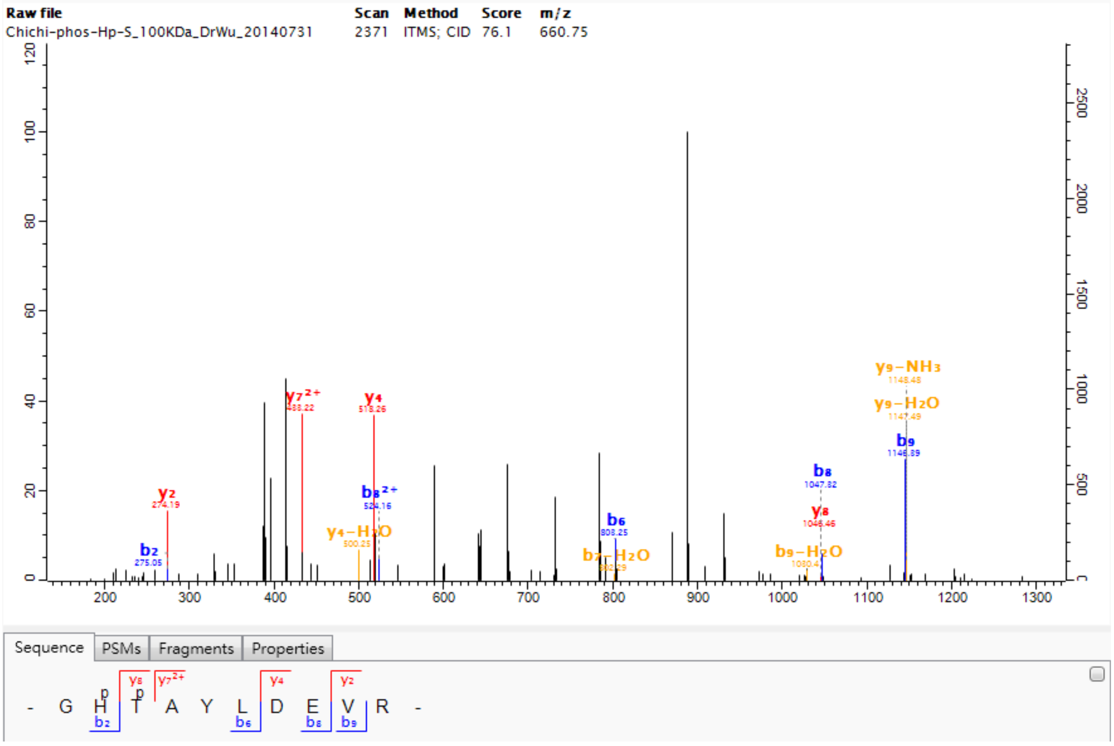
Raw file
Chichi-phos-Hp-S_100KDa_DrWu_20140731
Scan Method Score m/z
2371 ITMS; CID 76.1 660.75
b2
y2
y7²+
y4-H2O
y4
b8²+
b7-H2O
b6
b9-H2O
y8
b8
b9
y9-H2O
y9-NH3
Sequence PSMs Fragments Properties
- G H T A Y L D E V R -

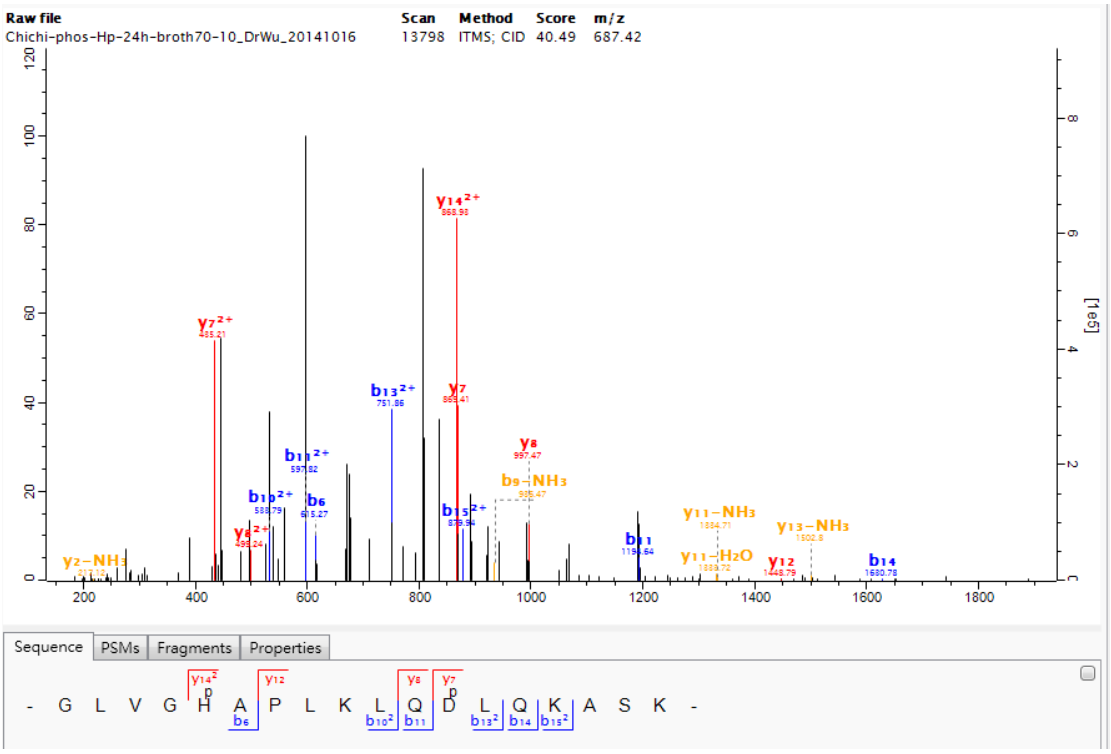
Raw file
Chichi-phos-Hp-24h-broth70-10_DrWu_20141016
Scan Method Score m/z
13798 ITMS; CID 40.49 687.42
y14 2+
y7 2+
b13 2+
y7
b11 2+
y8
b9-NH3
b10 2+
b6
b15 2+
y6 2+
b11
y11-NH3
y13-NH3
y11-H2O
y12
b14
y2-NH3
[1e5]
Sequence PSMs Fragments Properties
- G L V G H A P L K L Q D L Q K A S K -

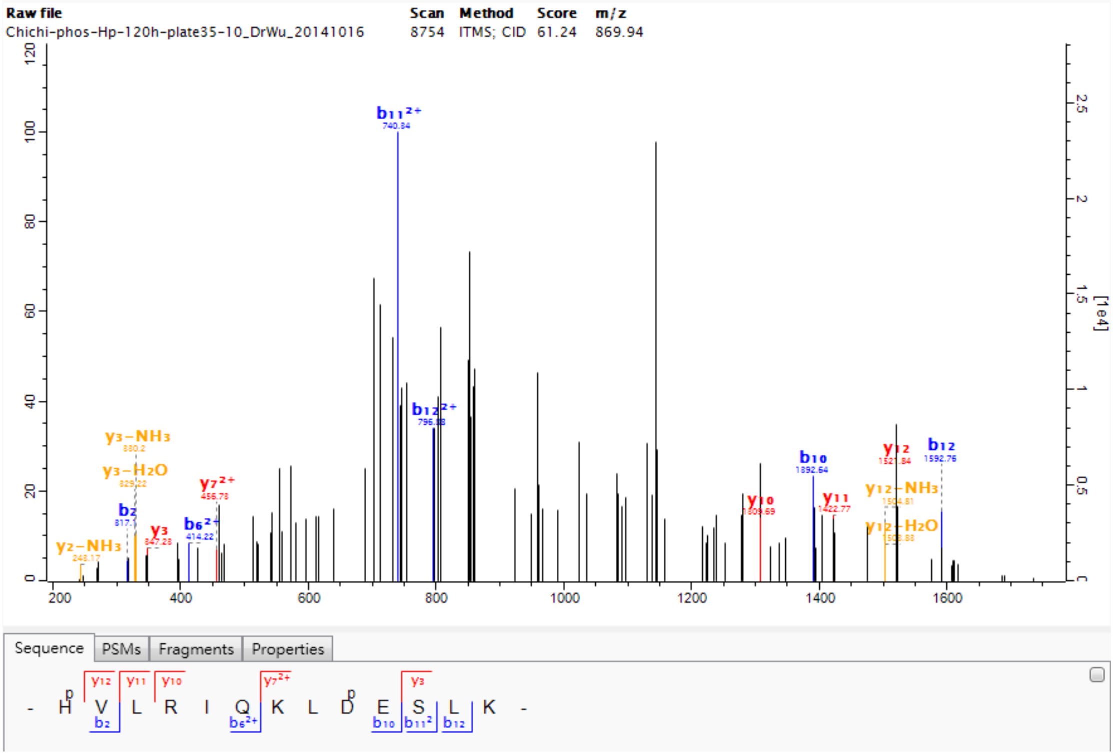
Raw file
Chichi-phos-Hp-120h-plate35-10_DrWu_20141016
Scan Method Score m/z
8754 ITMS; CID 61.24 869.94
b11 2+
b12 2+
y3-NH3
y3-H2O
y7 2+
b2
y3
b6 2+
y2-NH3
b10
y12
b12
y10
y11
y12-NH3
y12-H2O
[1e4]
Sequence PSMs Fragments Properties
- H V L R I Q K L D E S L K -

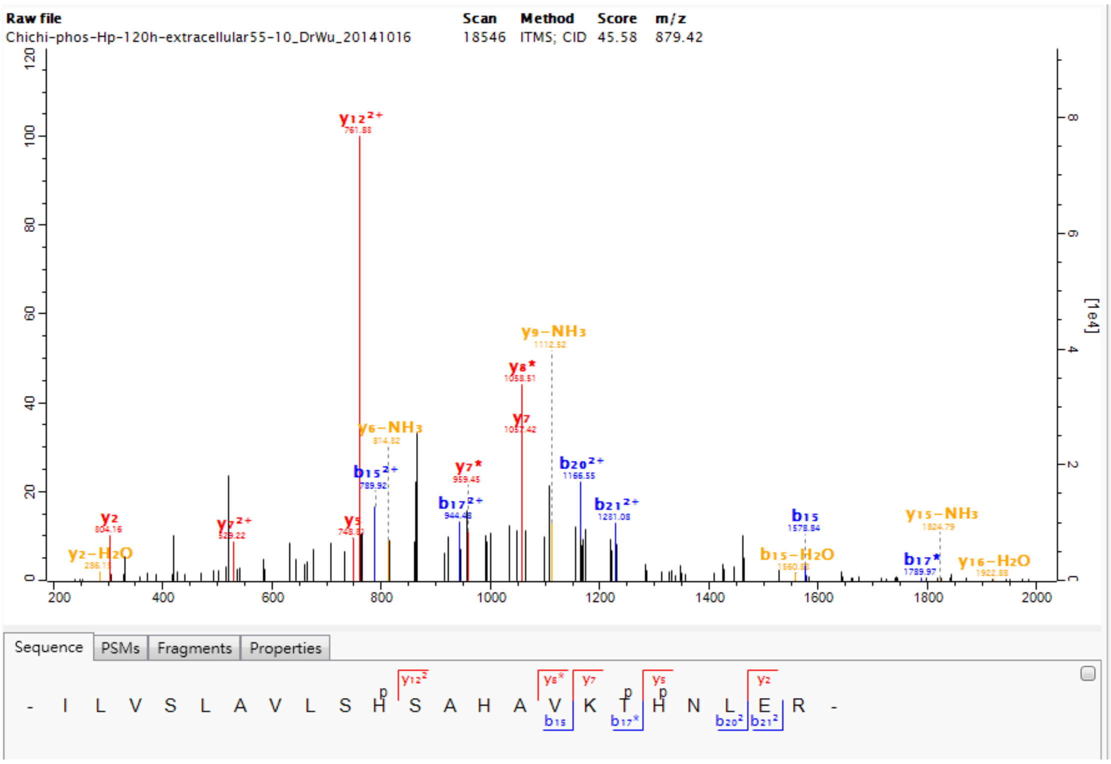
Raw file
Chichi-phos-Hp-120h-extracellular55-10_DrWu_20141016
Scan
18546
Method
ITMS; CID
Score
45.58
m/z
879.42
[1e4]
Sequence
PSMs
Fragments
Properties
- I L V S L A V L S H S A H A V K T H N L E R -

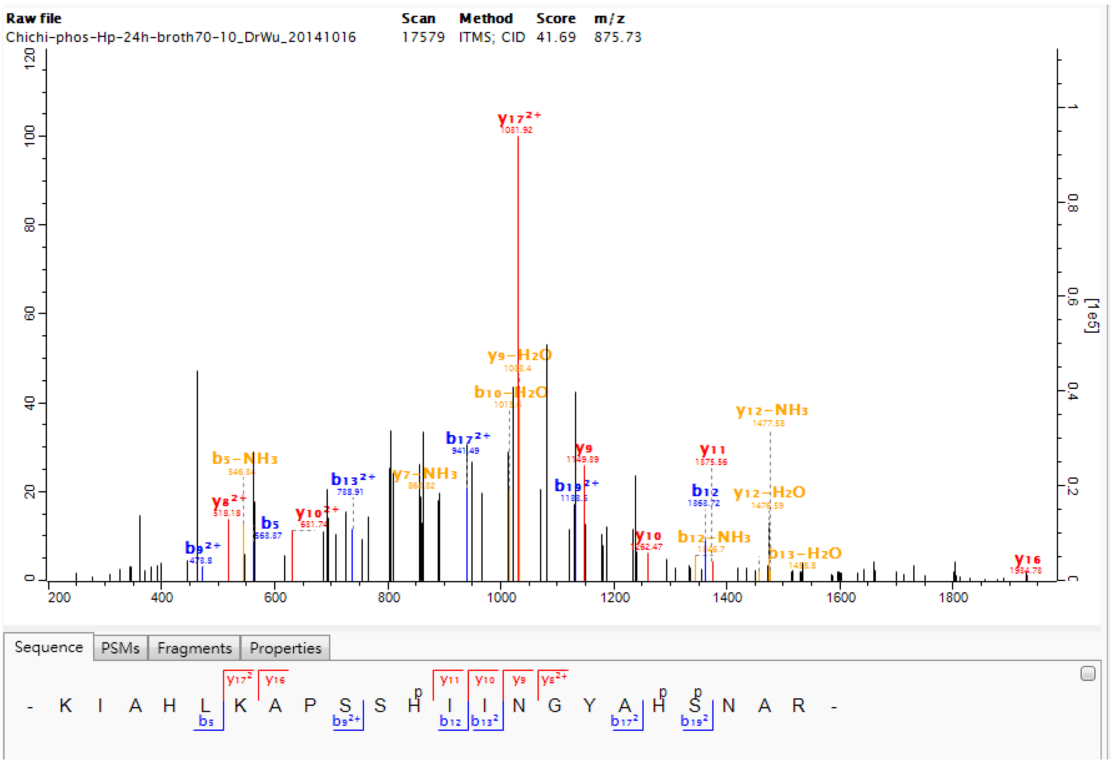
Raw file
Chichi-phos-Hp-24h-broth70-10_DrWu_20141016
Scan
17579
Method
ITMS; CID
Score
41.69
m/z
875.73
[1e5]
Sequence
PSMs
Fragments
Properties
- K I A H L K A P S S H I I N G Y A H S N A R -

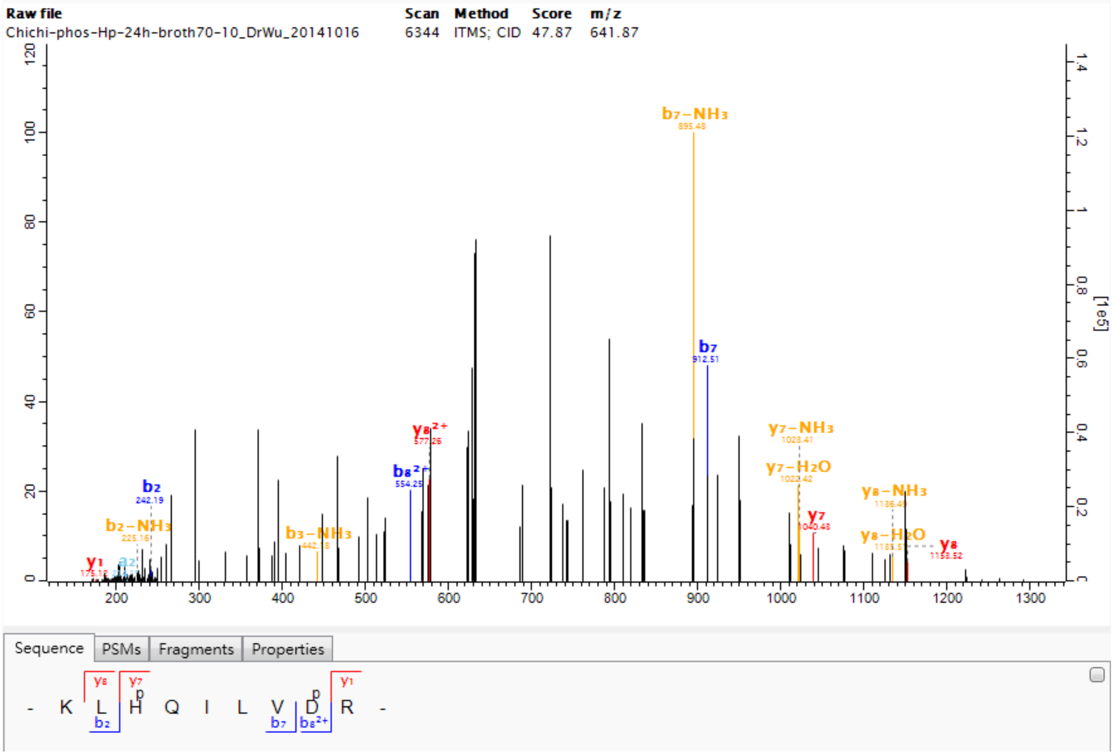
Raw file
Chichi-phos-Hp-24h-broth70-10_DrWu_20141016
Scan 6344
Method ITMS; CID
Score 47.87
m/z 641.87
[1e5]
b7-NH3
b7
y7-NH3
y7-H2O
y7
y8-NH3
y8-H2O
y8
y8 2+
b8 2+
b2
b2-NH3
b3-NH3
y1
Sequence
PSMs
Fragments
Properties
- K L H Q I L V D R -

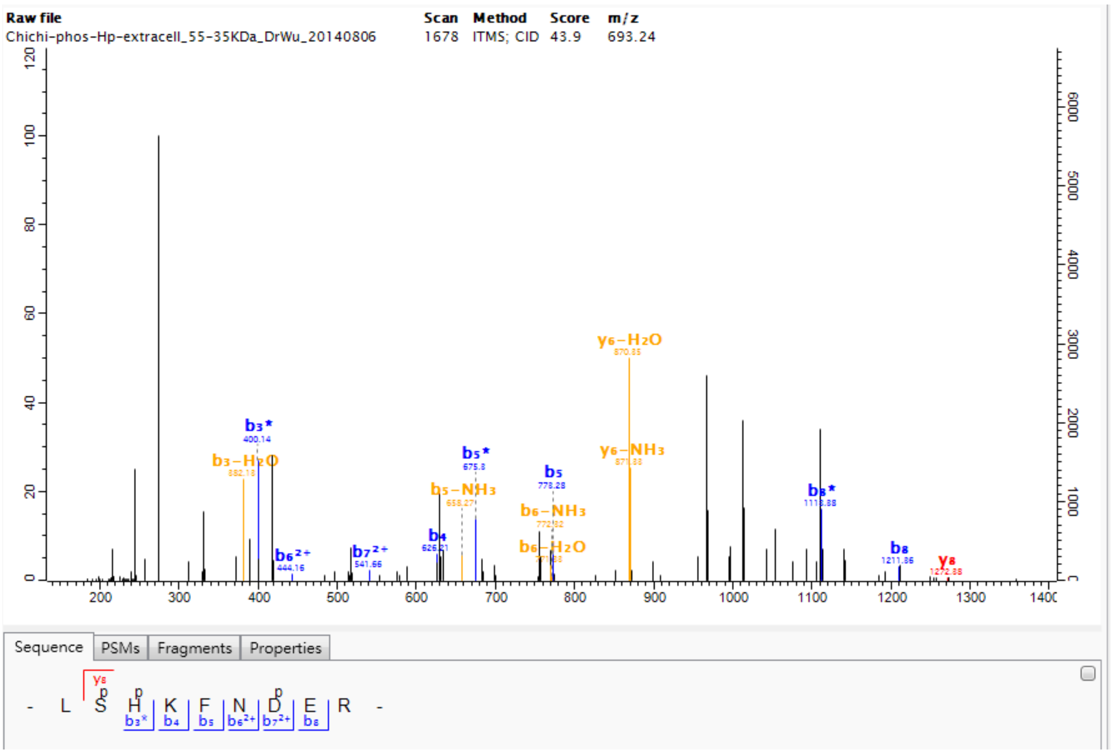
Raw file
Chichi-phos-Hp-extracell_55-35KDa_DrWu_20140806
Scan 1678
Method ITMS; CID
Score 43.9
m/z 693.24
y6-H2O
y6-NH3
b3*
b3-H2O
b5*
b5-NH3
b5
b6-NH3
b6-H2O
b6 2+
b7 2+
b4
b8*
b8
y8
Sequence
PSMs
Fragments
Properties
- L S H K F N D E R -

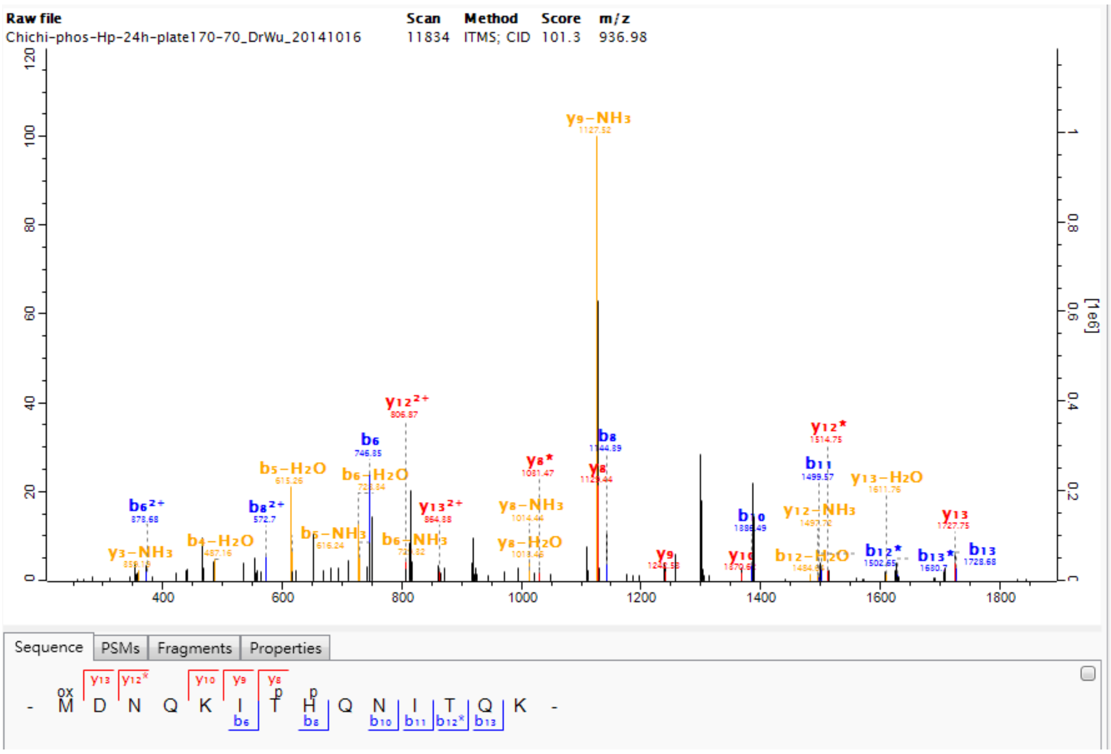
Raw file
Chichi-phos-Hp-24h-plate170-70_DrWu_20141016
Scan 11834
Method ITMS; CID
Score 101.3
m/z 936.98
y9-NH3
y12 2+
b6
y12*
b8
y8*
y8
b11
b5-H2O
b6-H2O
y13-H2O
b6 2+
b8 2+
y13 2+
y8-NH3
y12-NH3
b10
y13
b4-H2O
b5-NH3
b6-NH3
y8-H2O
y3-NH3
y9
y10
b12-H2O
b12*
b13*
b13
[1e6]
Sequence
PSMs
Fragments
Properties
- M D N Q K I T H Q N I T Q K -

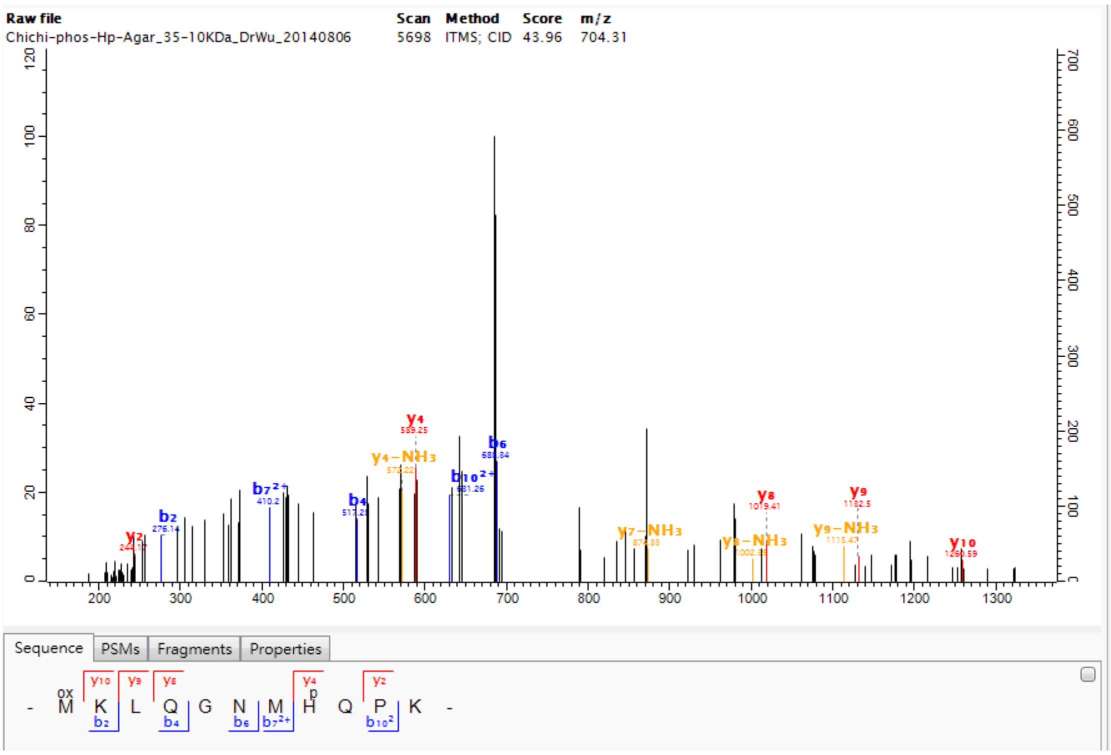
Raw file
Chichi-phos-Hp-Agar_35-10KDa_DrWu_20140806
Scan 5698
Method ITMS; CID
Score 43.96
m/z 704.31
y4
b6
y4-NH3
b10 2+
b7 2+
b4
b2
y2
y8
y9
y7-NH3
y8-NH3
y9-NH3
y10
Sequence
PSMs
Fragments
Properties
- M K L Q G N M H Q P K -

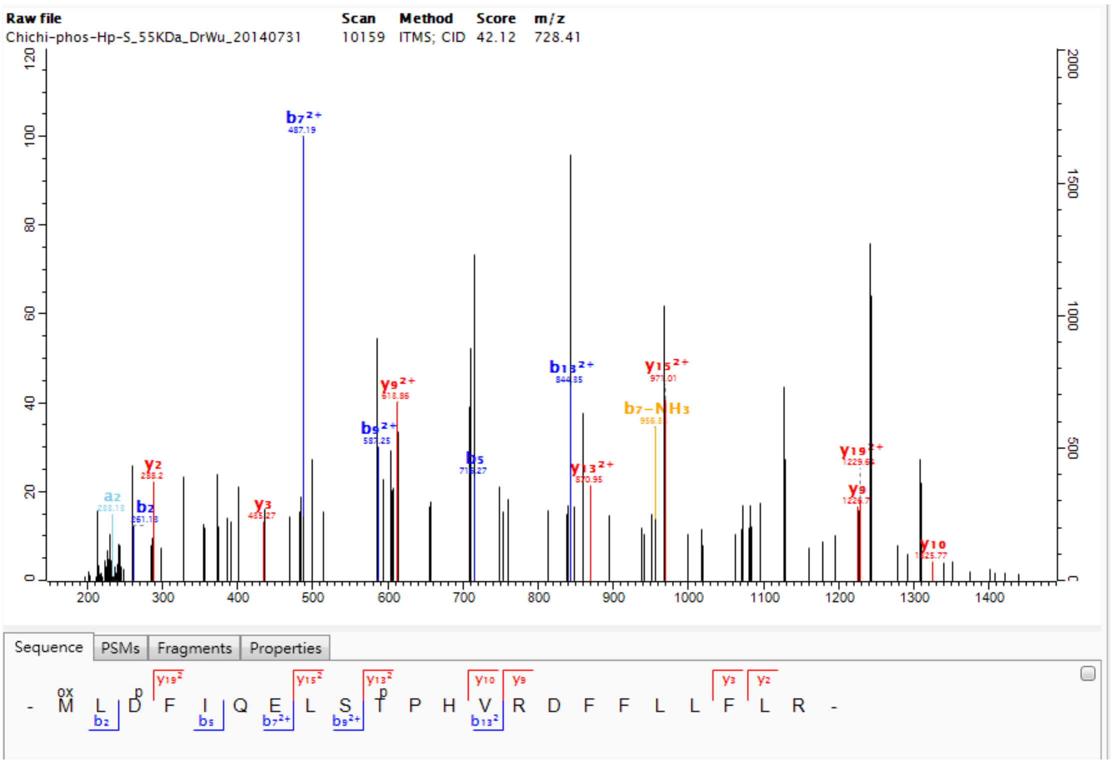
Raw file
Chichi-phos-Hp-S_55KDa_DrWu_20140731
Scan
10159
Method
ITMS; CID
Score
42.12
m/z
728.41
Sequence
PSMs
Fragments
Properties
- M L D F I Q E L S T P H V R D F F L L F L R -

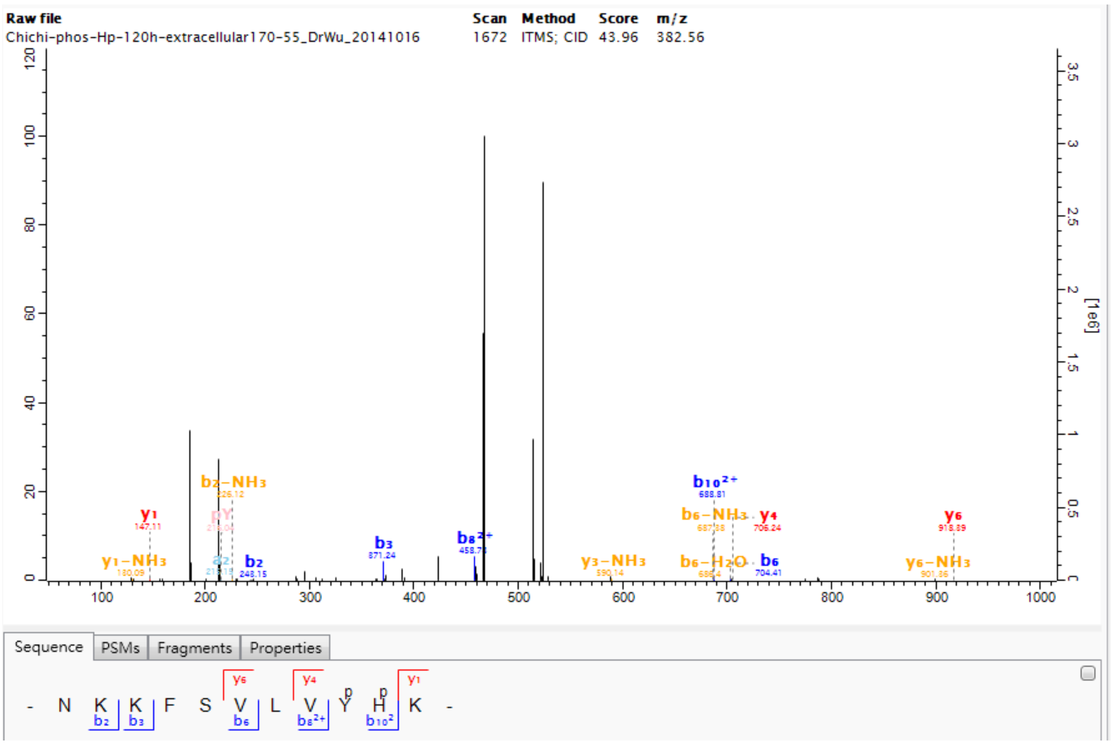
Raw file
Chichi-phos-Hp-120h-extracellular170-55_DrWu_20141016
Scan
1672
Method
ITMS; CID
Score
43.96
m/z
382.56
[1e6]
Sequence
PSMs
Fragments
Properties
- N K K F S V L V Y H K -

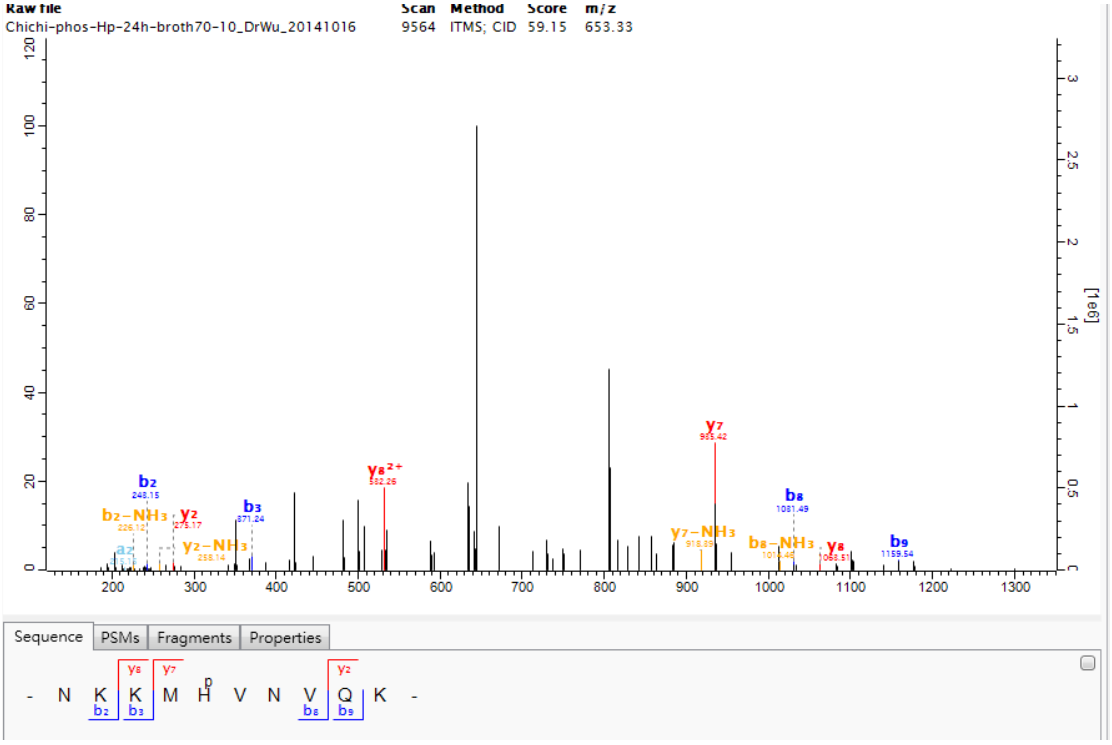
Raw file
Chichi-phos-Hp-24h-broth70-10_DrWu_20141016
Scan Method Score m/z
9564 ITMS; CID 59.15 653.33
[1e6]
Sequence PSMs Fragments Properties
- N K K M H V N V Q K -

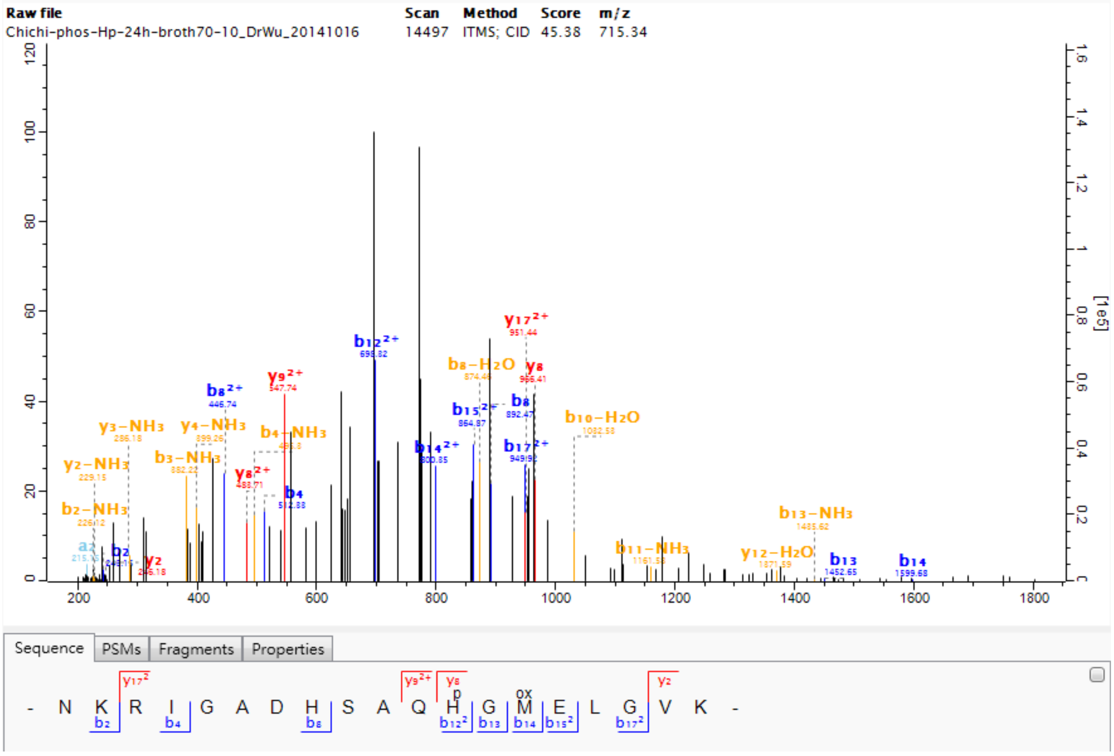
Raw file
Chichi-phos-Hp-24h-broth70-10_DrWu_20141016
Scan Method Score m/z
14497 ITMS; CID 45.38 715.34
[1e6]
Sequence PSMs Fragments Properties
- N K R I G A D H S A Q H G M E L G V K -

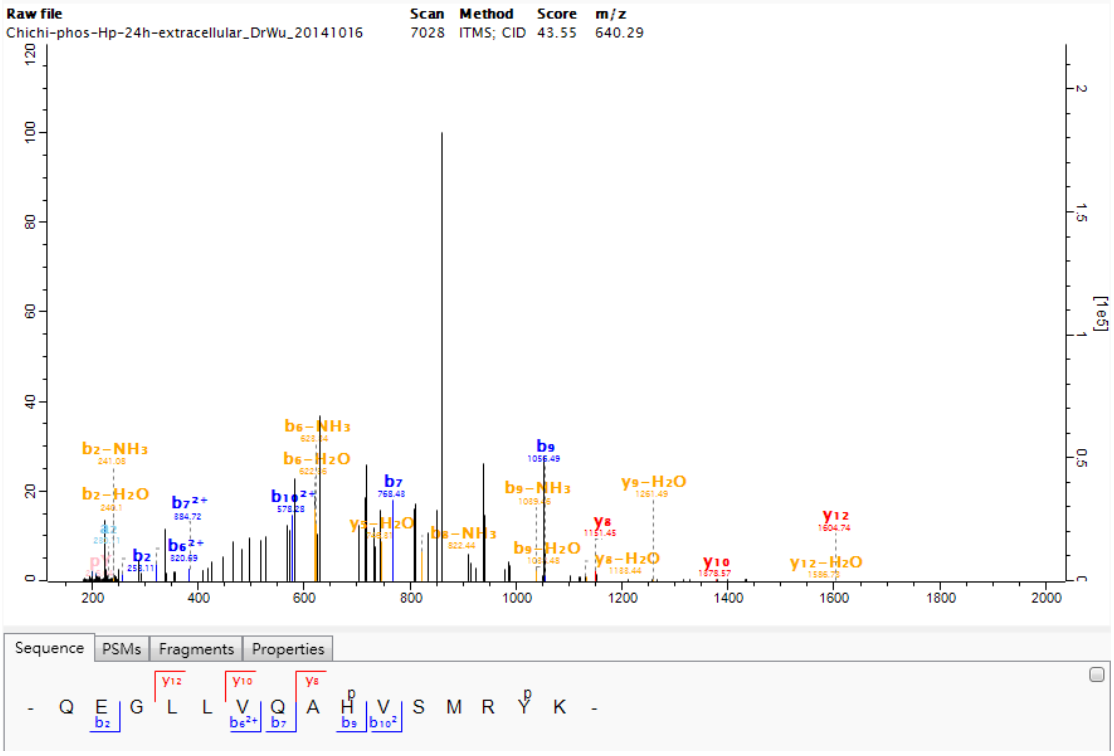
Raw file
Chichi-phos-Hp-24h-extracellular_DrWu_20141016
Scan Method Score m/z
7028 ITMS; CID 43.55 640.29
[1e5]
b2-NH3
b2-H2O
b7 2+
b6 2+
b2
b10 2+
b6-NH3
b6-H2O
b7
b9
b9-NH3
b8-NH3
b9-H2O
y9-H2O
y8
y8-H2O
y10
y12
y12-H2O
Sequence
PSMs
Fragments
Properties
- Q E G L L V Q A H V S M R Y K -

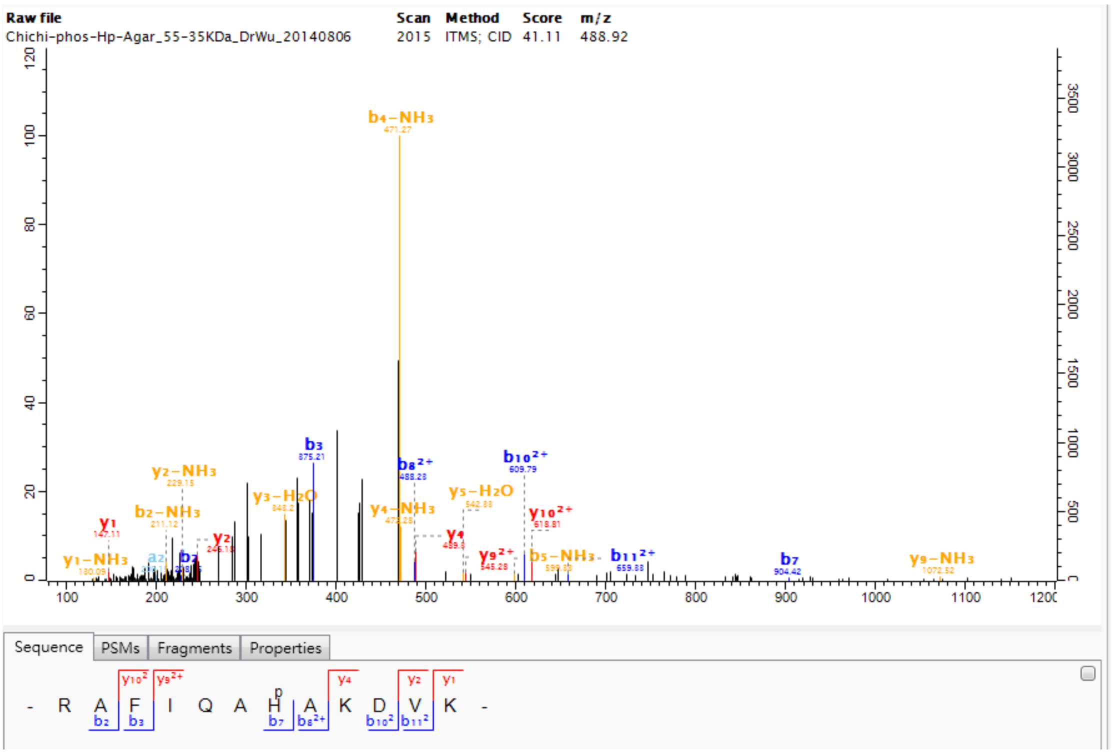
Raw file
Chichi-phos-Hp-Agar_55-35KDa_DrWu_20140806
Scan Method Score m/z
2015 ITMS; CID 41.11 488.92
b4-NH3
y2-NH3
b2-NH3
y1
y1-NH3
y3-H2O
b3
b8 2+
y4-NH3
y5-H2O
b10 2+
y10 2+
y9 2+
b5-NH3
b11 2+
b7
y9-NH3
Sequence
PSMs
Fragments
Properties
- R A F I Q A H A K D V K -

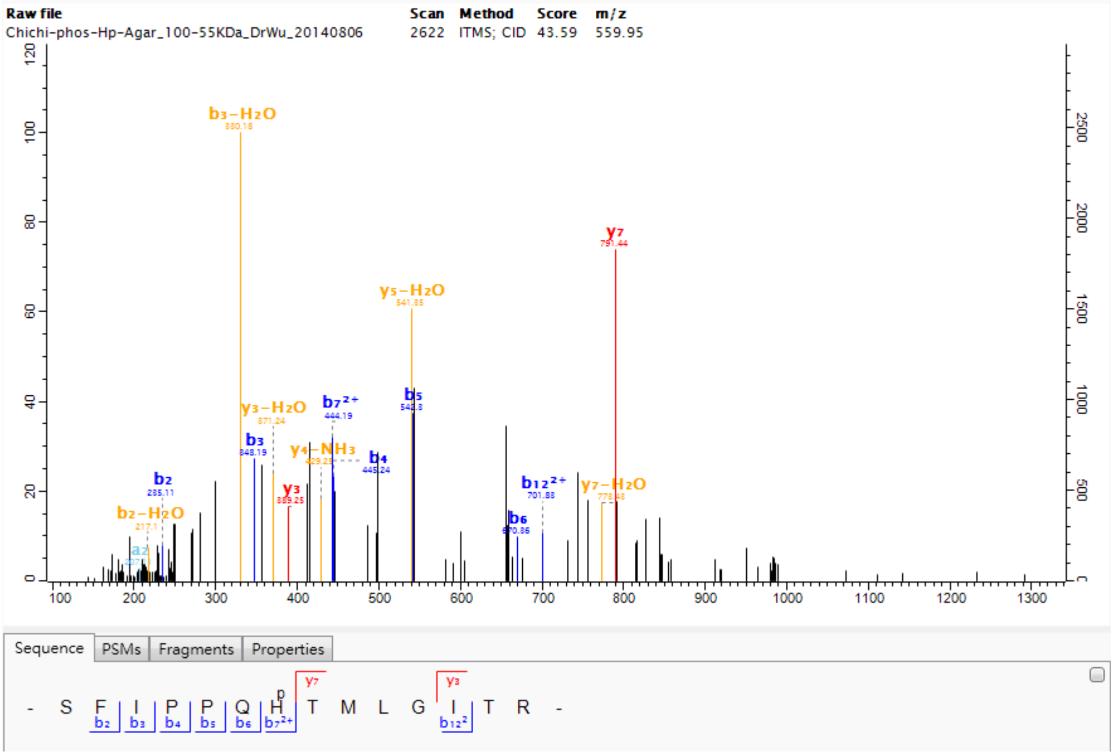
Raw file
Chichi-phos-Hp-Agar_100-55KDa_DrWu_20140806
Scan 2622
Method ITMS; CID
Score 43.59
m/z 559.95
b3-H2O
y7
y5-H2O
b5
b7 2+
y3-H2O
b3
y4-NH3
b4
b2
b12 2+
y7-H2O
y3
b2-H2O
b6
Sequence
PSMs
Fragments
Properties
- S F I P P Q H T M L G I T R -

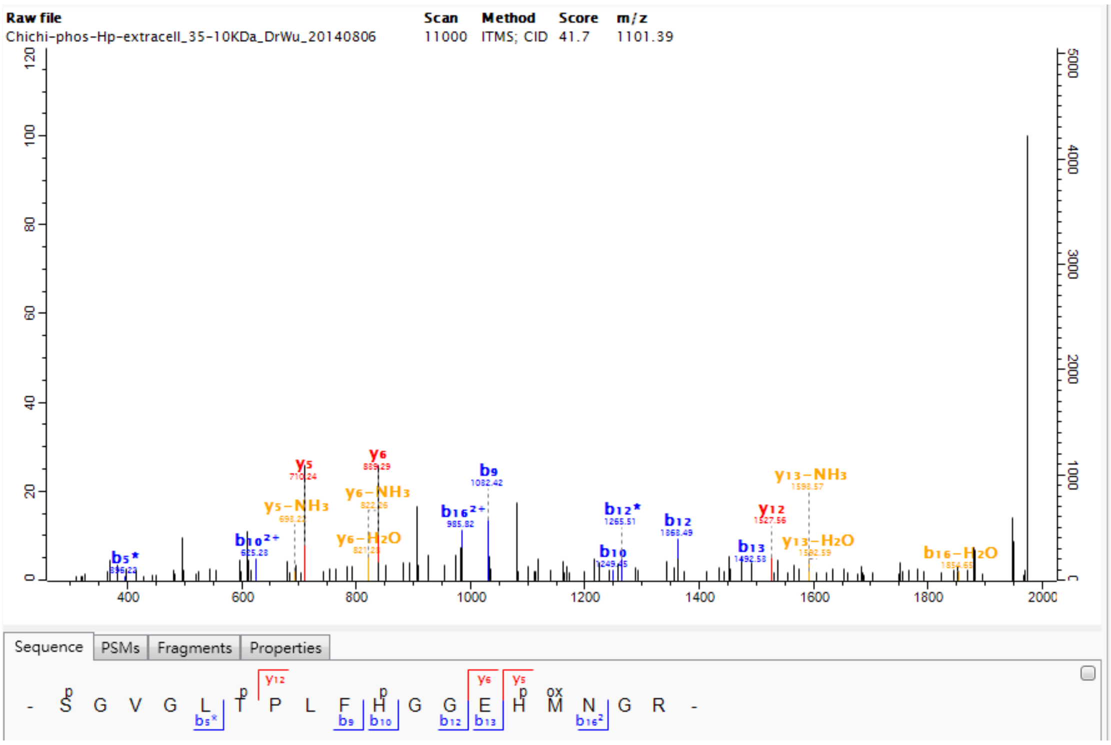
Raw file
Chichi-phos-Hp-extracell_35-10KDa_DrWu_20140806
Scan 11000
Method ITMS; CID
Score 41.7
m/z 1101.39
y5
y6
b9
y5-NH3
y6-NH3
b16 2+
b12*
b12
y12
y13-NH3
b10 2+
y6-H2O
b10
b13
y13-H2O
b16-H2O
b5*
Sequence
PSMs
Fragments
Properties
- S G V G L T P L F H G G E H M N G R -

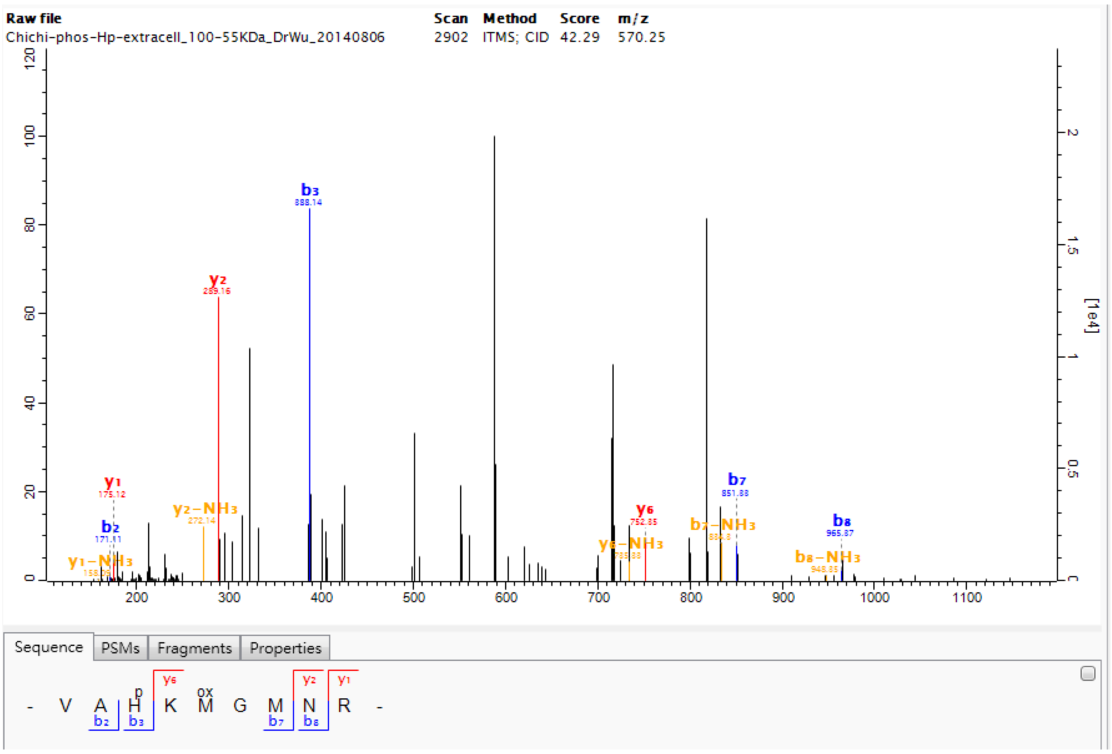
Raw file
Chichi-phos-Hp-extracell_100-55KDa_DrWu_20140806
Scan 2902
Method ITMS; CID
Score 42.29
m/z 570.25
[1e4]
b3
y2
y1
b2
y2-NH3
y1-NH3
b7
b8
y6
b7-NH3
y6-NH3
b8-NH3
Sequence
PSMs
Fragments
Properties
- V A H K M G M N R -

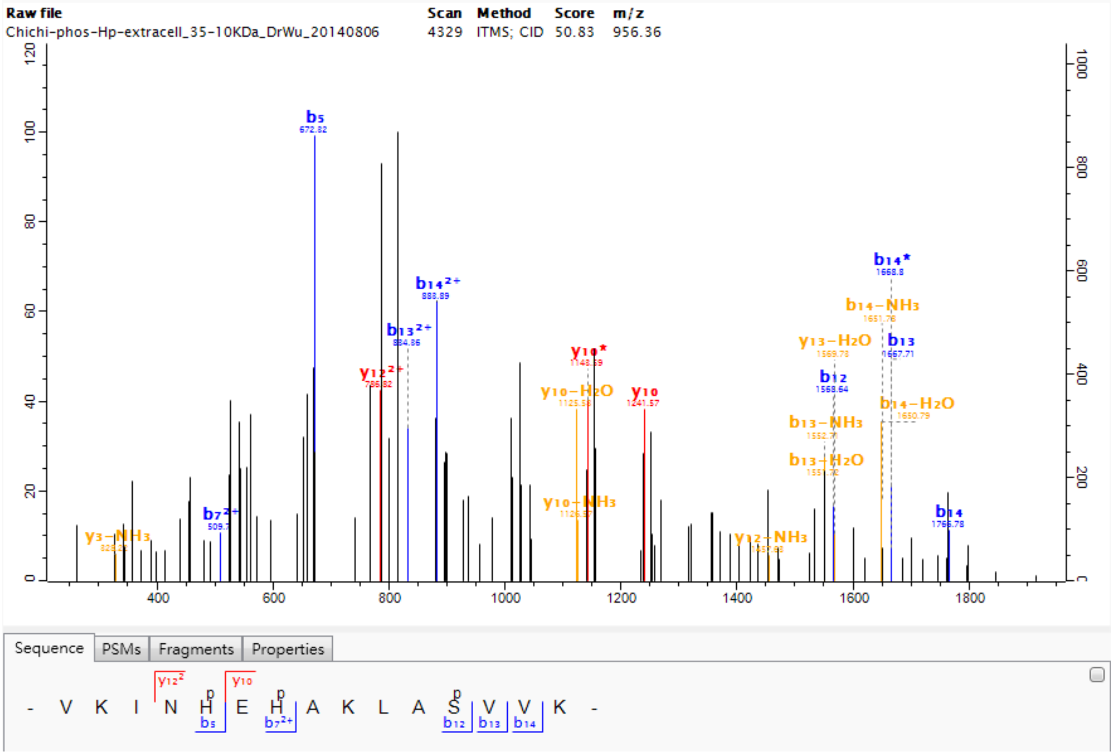
Raw file
Chichi-phos-Hp-extracell_35-10KDa_DrWu_20140806
Scan 4329
Method ITMS; CID
Score 50.83
m/z 956.36
b5
b14 2+
b13 2+
y12 2+
y10*
y10-H2O
y10
b14*
b14-NH3
y13-H2O
b13
b12
b13-NH3
b13-H2O
b14-H2O
b14
b7 2+
y3-NH3
y10-NH3
y12-NH3
Sequence
PSMs
Fragments
Properties
- V K I N H E H A K L A S V V K -

Klebsiella pneumonia NTUH-K2044 (pathogenic bacterium)

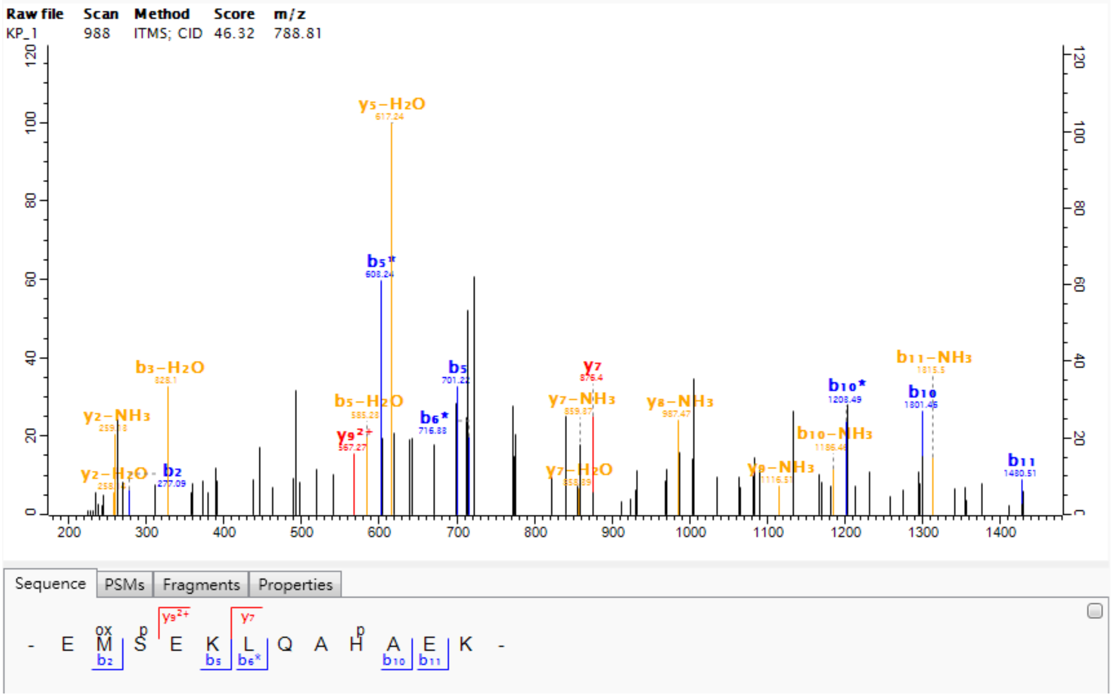

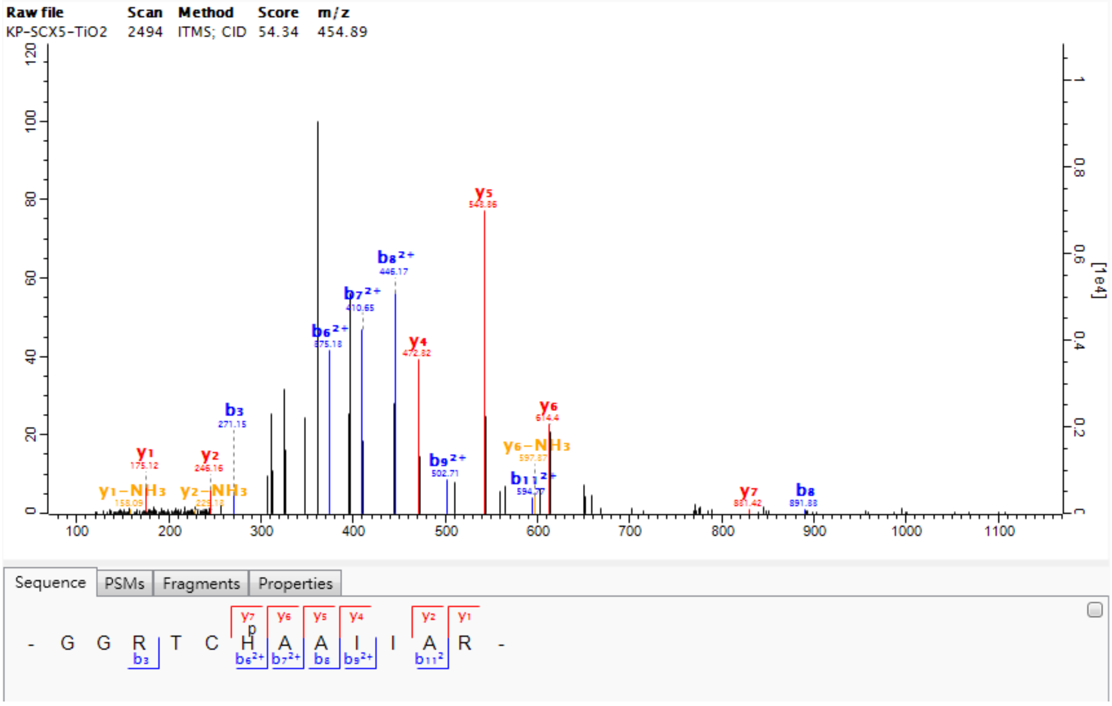

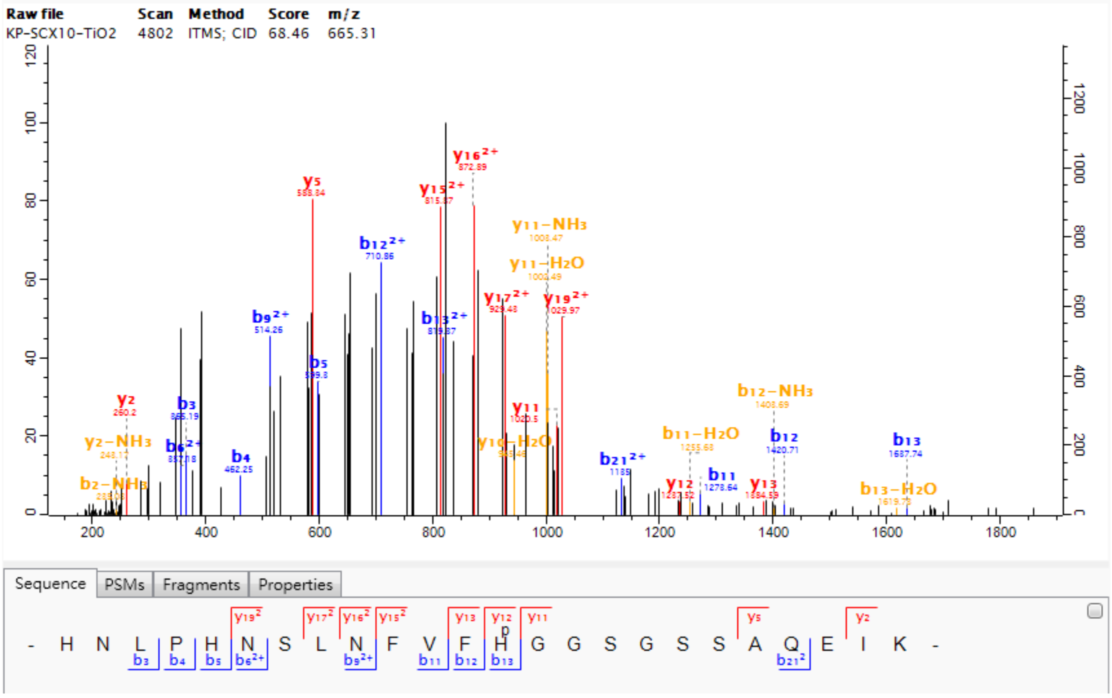
Raw file Scan Method Score m/z
KP-SCX10-TiO2 4802 ITMS; CID 68.46 665.31
y5
y15 2+
y16 2+
y11-NH3
y11-H2O
b12 2+
b9 2+
b13 2+
y17 2+
y19 2+
b5
y2
b3
y2-NH3
b6 2+
b4
b2-NH3
y11
y10-H2O
b21 2+
b11-H2O
b12-NH3
b12
b13
b11
y12
y13
b13-H2O
Sequence PSMs Fragments Properties
- H N L P H N S L N F V F H G G S G S S A Q E I K -

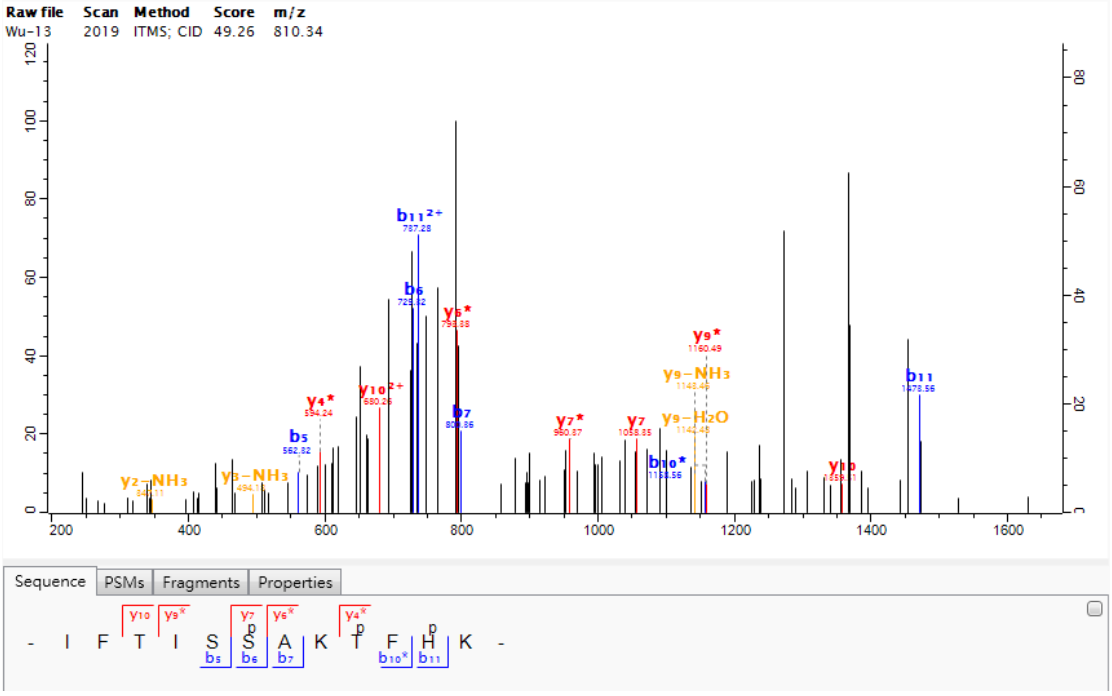
Raw file Scan Method Score m/z
Wu-13 2019 ITMS; CID 49.26 810.34
b11 2+
b6
y6*
y4*
y10 2+
b5
b7
y7*
y7
y9*
y9-NH3
y9-H2O
b10*
b11
y2-NH3
y3-NH3
Sequence PSMs Fragments Properties
- I F T I S S A K T F H K -

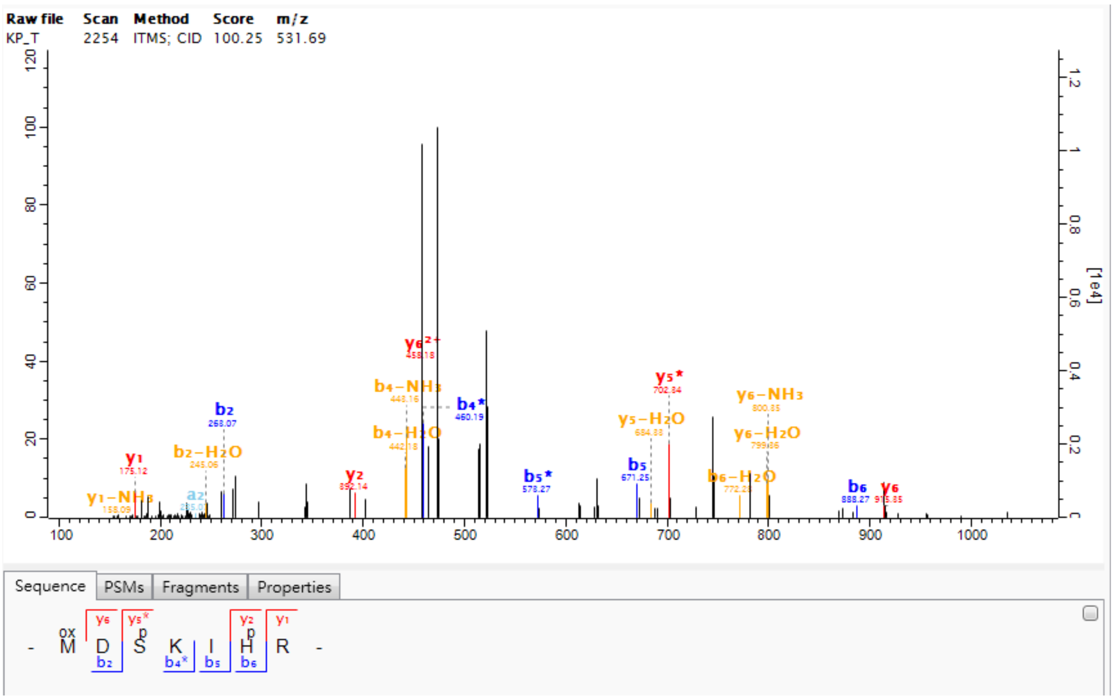
Raw file Scan Method Score m/z
KP_T 2254 ITMS; CID 100.25 531.69
Sequence
PSMs
Fragments
Properties
- M D S K I H R -

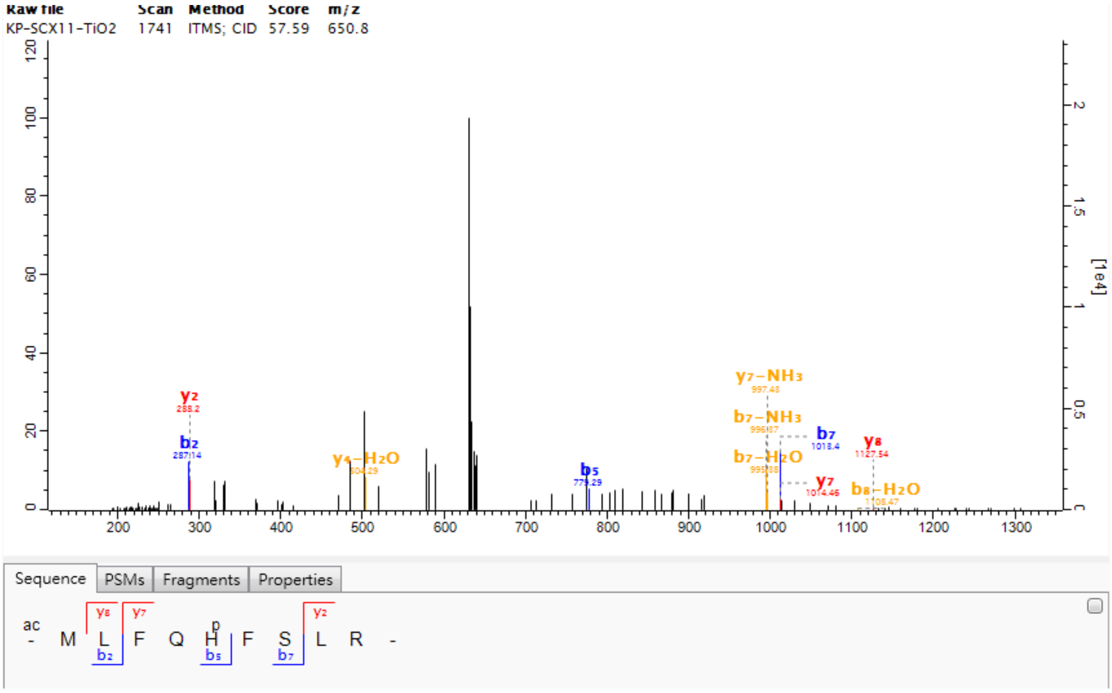
Raw file Scan Method Score m/z
KP-SCX11-TiO2 1741 ITMS; CID 57.59 650.8
Sequence
PSMs
Fragments
Properties
- M L F Q H F S L R -

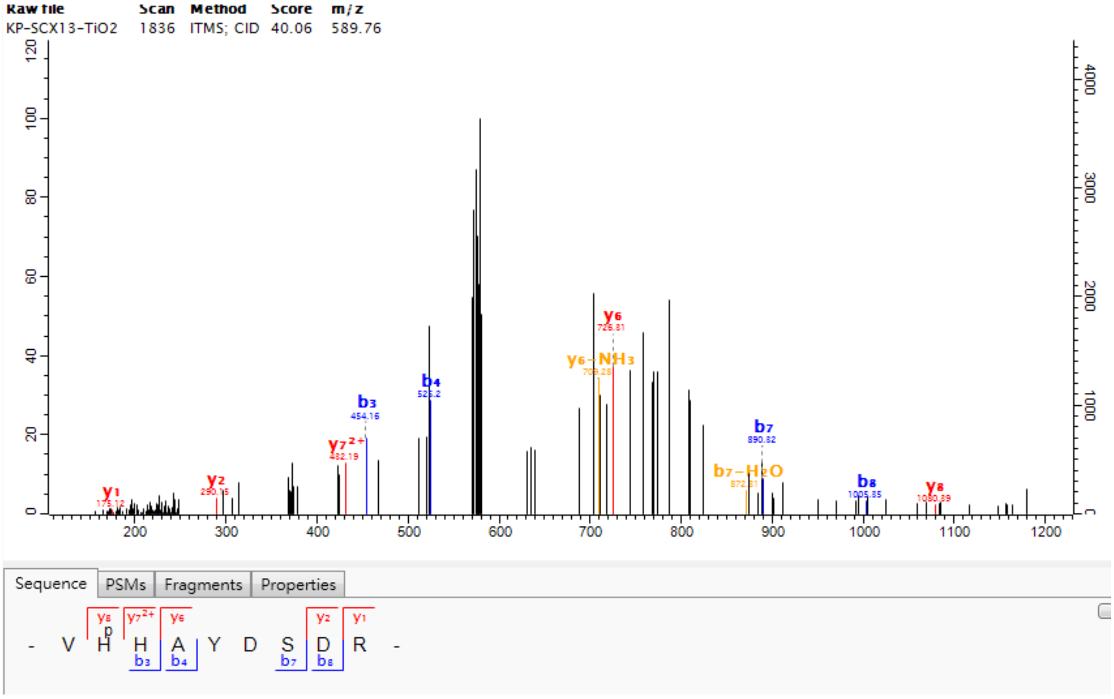

Raw file Scan Method Score m/z
KP-SCX13-TiO2 1836 ITMS; CID 40.06 589.76
y1
y2
y7 2+
b3
454.16
b4
y6-NH3
y6
726.31
b7
890.32
b7-H2O
b8
y8
Sequence
PSMs
Fragments
Properties
- V H H A Y D S D R -

Vibrio vulnificus (pathogenic bacterium)

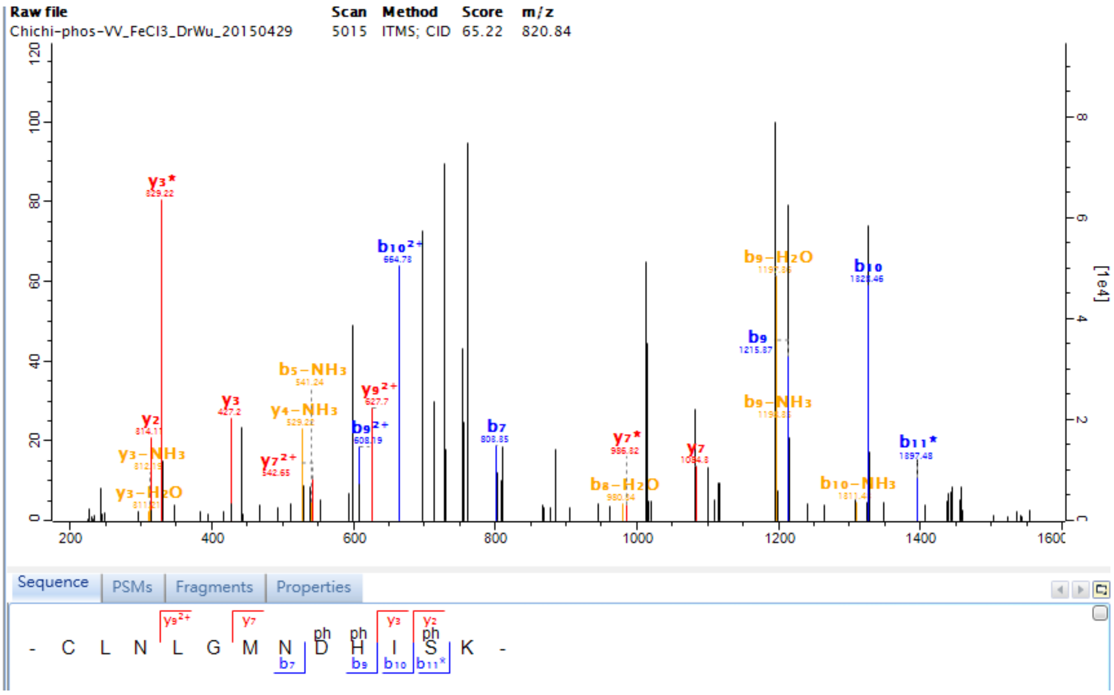

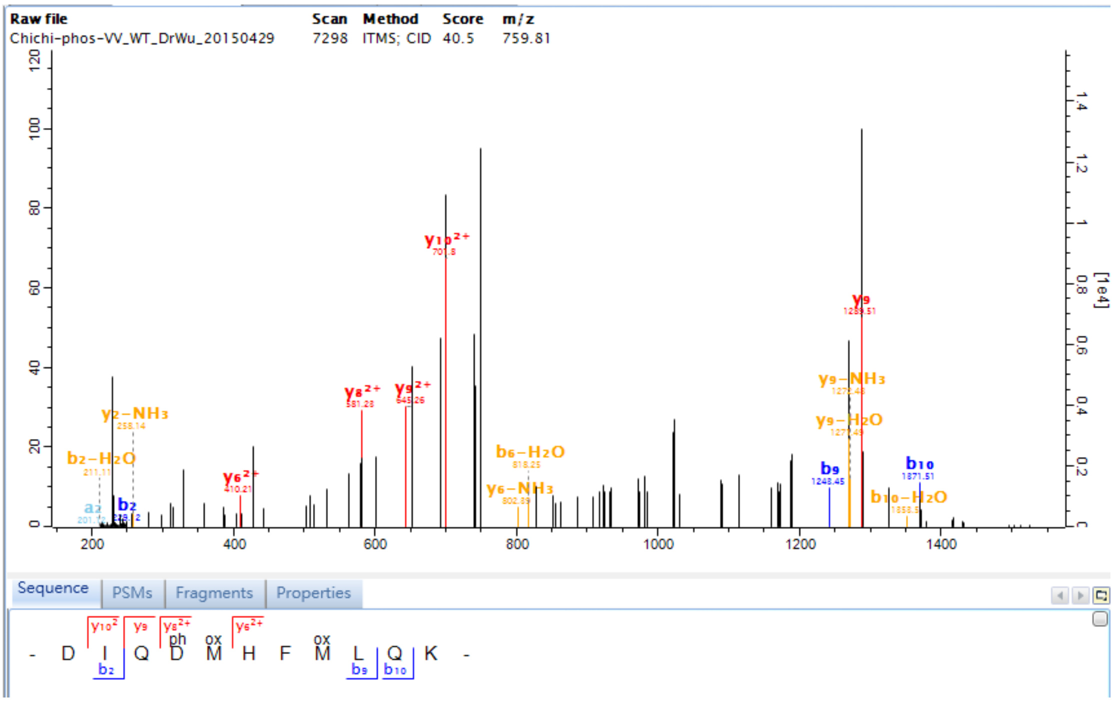

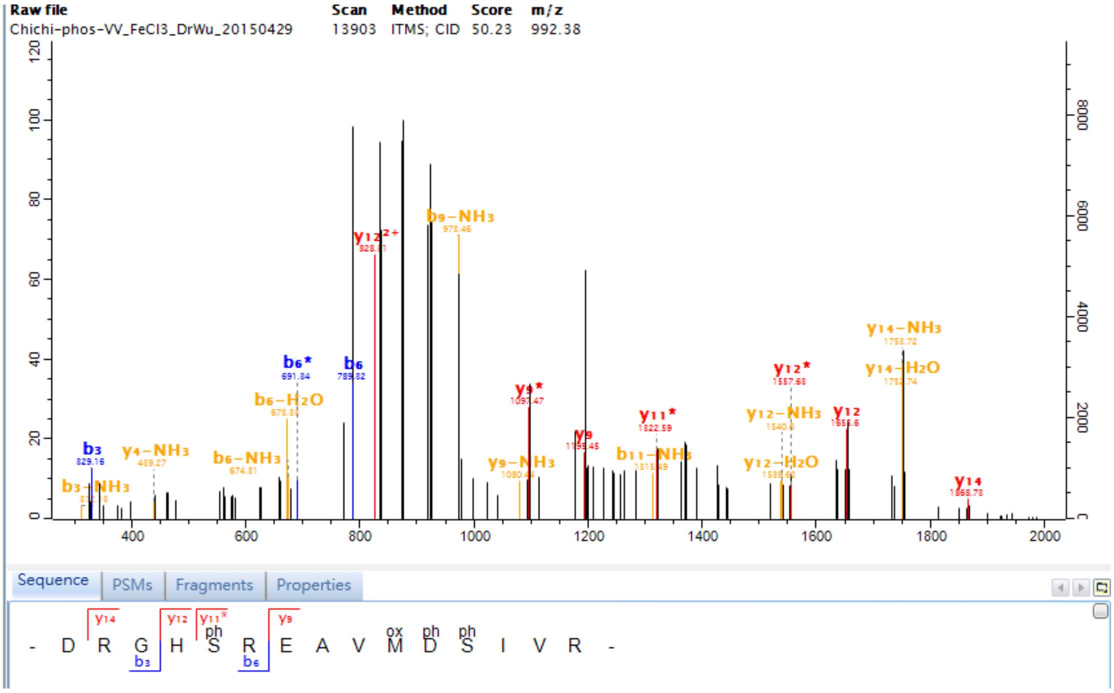
Raw file
Chichi-phos-VV_FeCl3_DrWu_20150429
Scan 13903
Method ITMS; CID
Score 50.23
m/z 992.38
b3
y4−NH3
b3−NH3
b6−NH3
b6−H2O
b6*
b6
y12 2+
b9−NH3
y9−NH3
y9
b11−NH3
y11*
y12−NH3
y12−H2O
y12*
y12
y14−NH3
y14−H2O
y14
Sequence
PSMs
Fragments
Properties
- D R G H S R E A V M D S I V R -

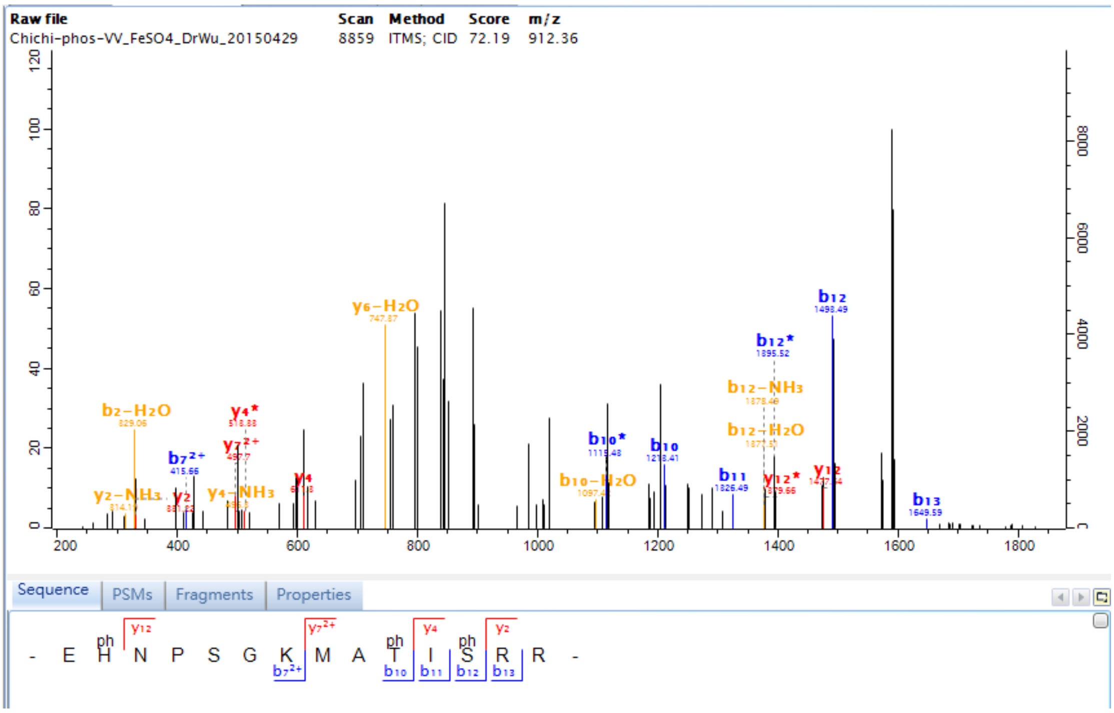
Raw file
Chichi-phos-VV_FeSO4_DrWu_20150429
Scan 8859
Method ITMS; CID
Score 72.19
m/z 912.36
b2−H2O
y2−NH3
y2
b7 2+
y4*
y7 2+
y4−NH3
y4
y6−H2O
b10*
b10−H2O
b10
b11
b12−NH3
b12−H2O
b12*
b12
y12*
y12
b13
Sequence
PSMs
Fragments
Properties
- E H N P S G K M A T I S R R -

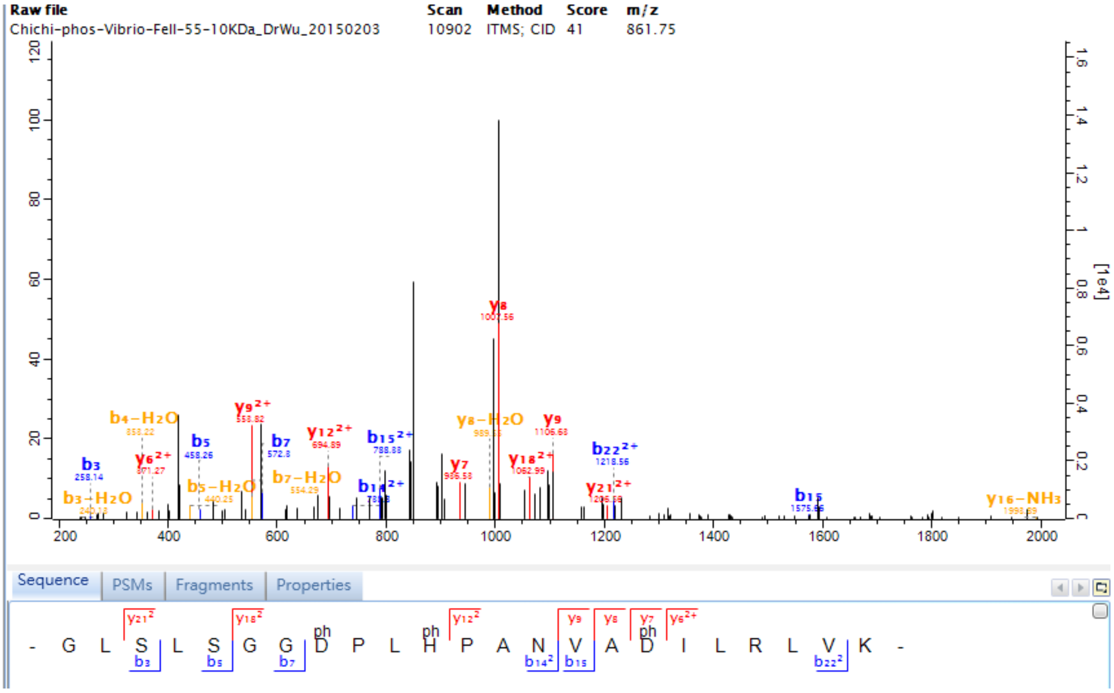
Raw file
Chichi-phos-Vibrio-FeII-55-10KDa_DrWu_20150203
Scan Method Score m/z
10902 ITMS; CID 41 861.75
[1e4]
Sequence PSMs Fragments Properties
- G L S L S G G D P L H P A N V A D I L R L V K -

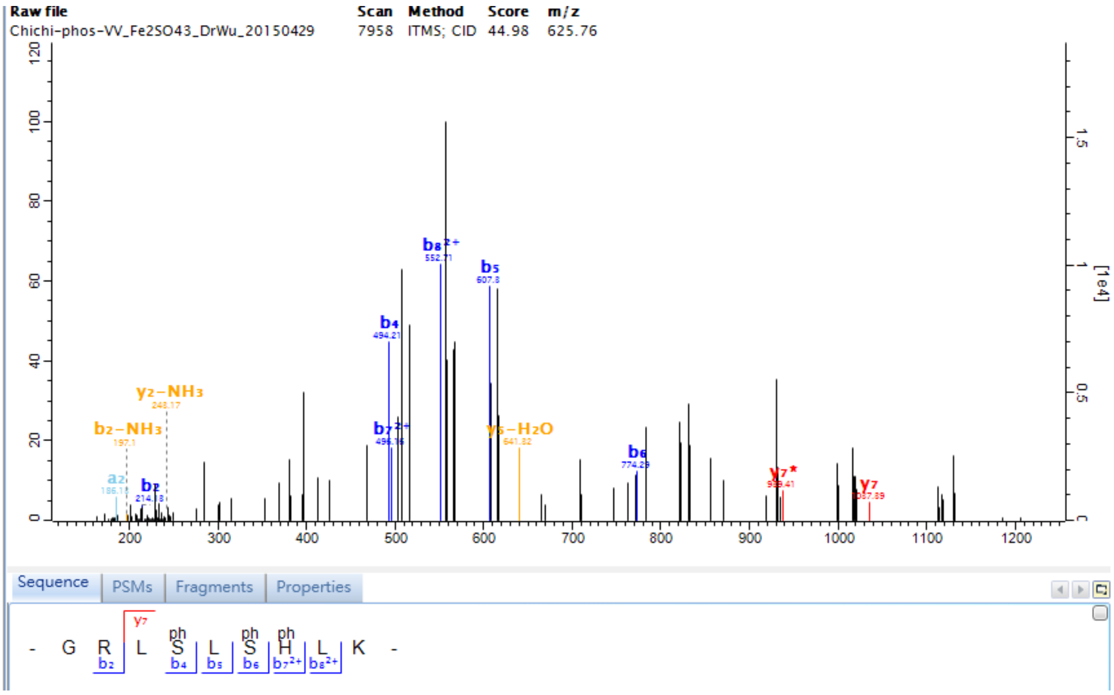
Raw file
Chichi-phos-VV_Fe2SO43_DrWu_20150429
Scan Method Score m/z
7958 ITMS; CID 44.98 625.76
[1e4]
Sequence PSMs Fragments Properties
- G R L S L S H L K -

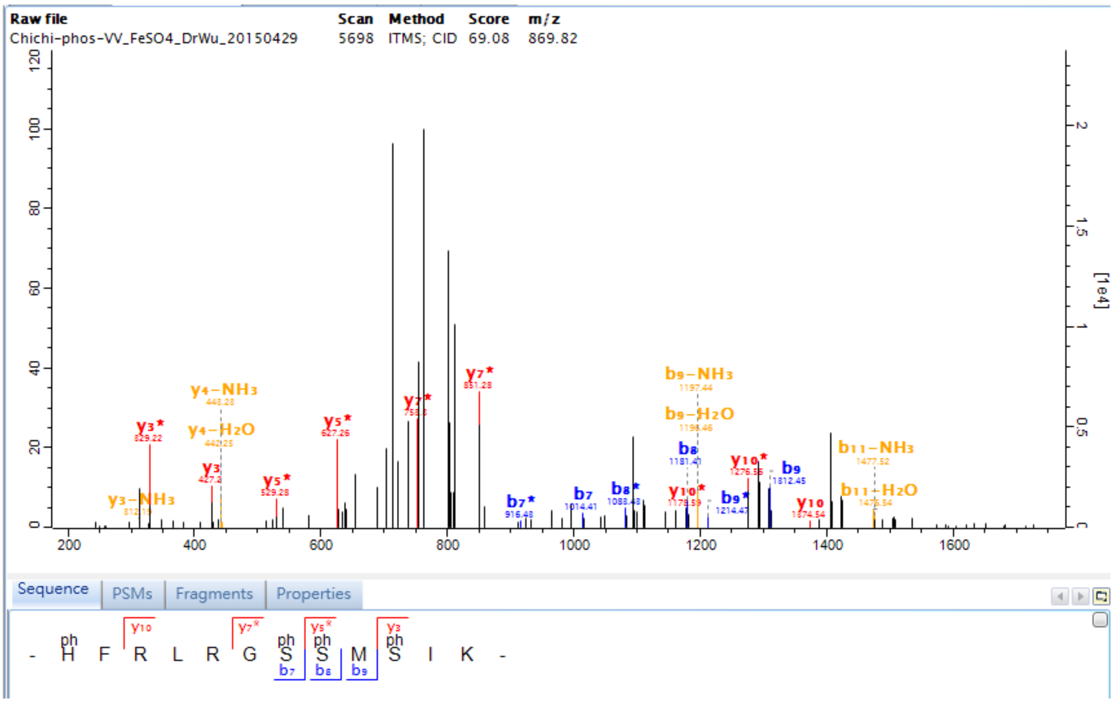
Raw file
Chichi-phos-VV_FeSO4_DrWu_20150429
Scan Method Score m/z
5698 ITMS; CID 69.08 869.82
[1e4]
Sequence
PSMs
Fragments
Properties
- H F R L R G S S M S I K -

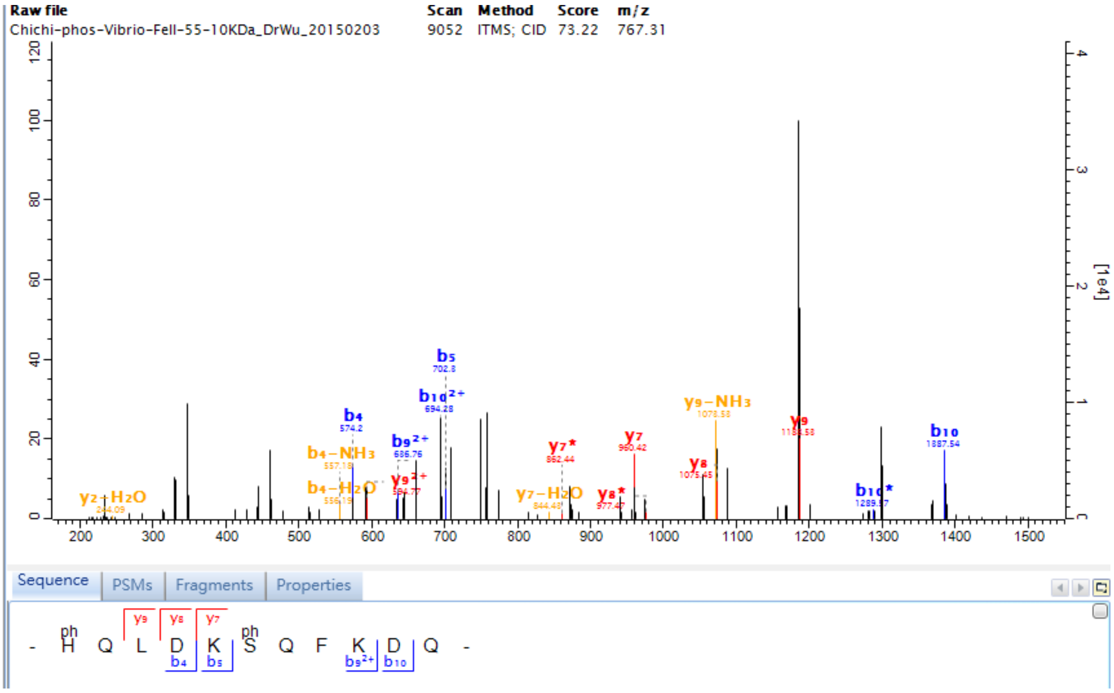
Raw file
Chichi-phos-Vibrio-FeII-55-10KDa_DrWu_20150203
Scan Method Score m/z
9052 ITMS; CID 73.22 767.31
[1e4]
Sequence
PSMs
Fragments
Properties
- H Q L D K S Q F K D Q -

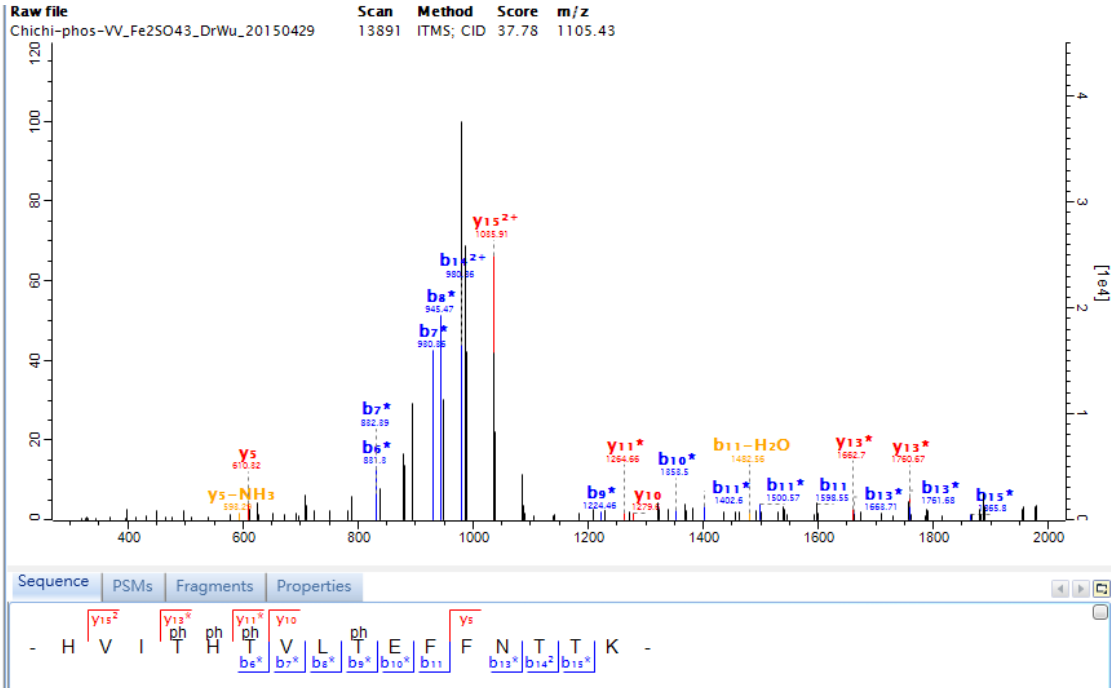
Raw file
Chichi-phos-VV_Fe2SO43_DrWu_20150429
Scan 13891
Method ITMS; CID
Score 37.78
m/z 1105.43
[1e4]
Sequence
PSMs
Fragments
Properties
- H V I T H T V L T E F F N T T K -

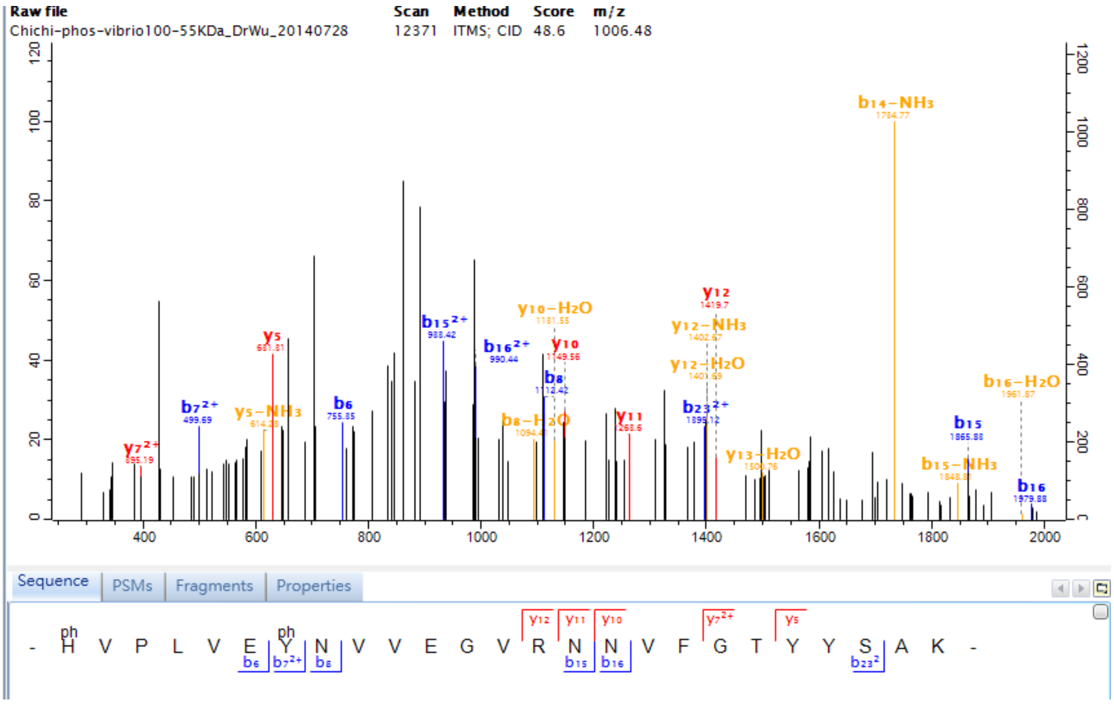
Raw file
Chichi-phos-vibrio100-55KDa_DrWu_20140728
Scan 12371
Method ITMS; CID
Score 48.6
m/z 1006.48
Sequence
PSMs
Fragments
Properties
- H V P L V E Y N V V E G V R N N V F G T Y Y S A K -

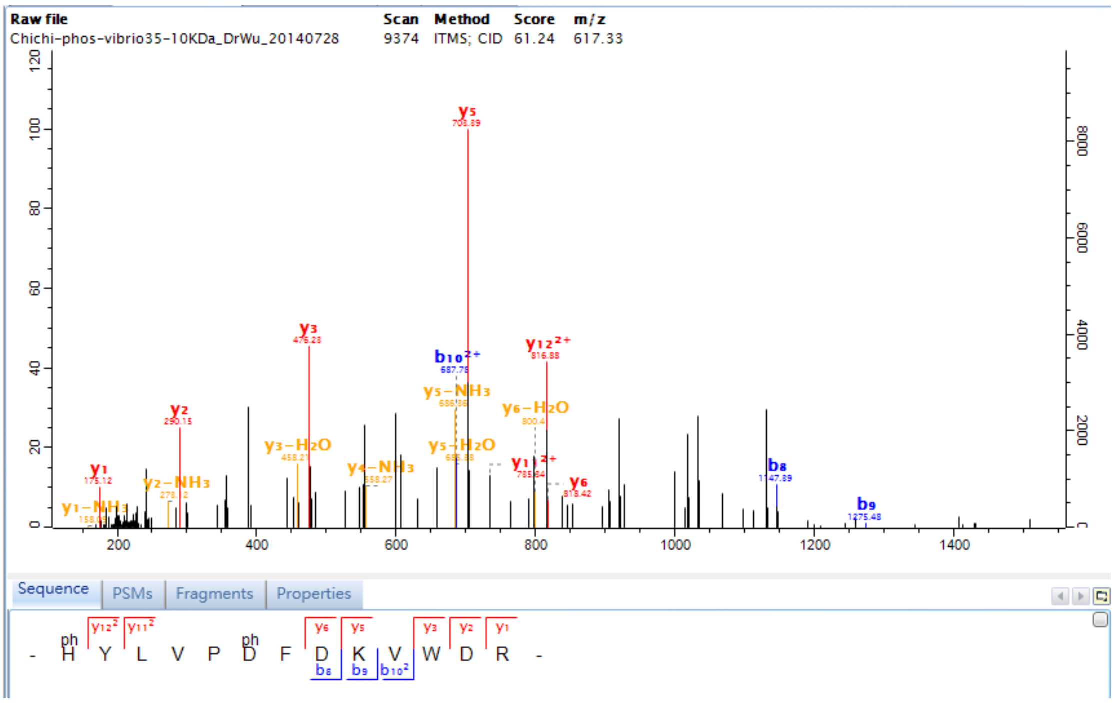
Raw file
Chichi-phos-vibrio35-10KDa_DrWu_20140728
Scan Method Score m/z
9374 ITMS; CID 61.24 617.33
y5
y3
b10 2+
y12 2+
y5-NH3
y6-H2O
y2
y3-H2O
y5-H2O
y4-NH3
y1
y2-NH3
y11 2+
y6
b8
b9
y1-NH3
Sequence
PSMs
Fragments
Properties
- H Y L V P D F D K V W D R -

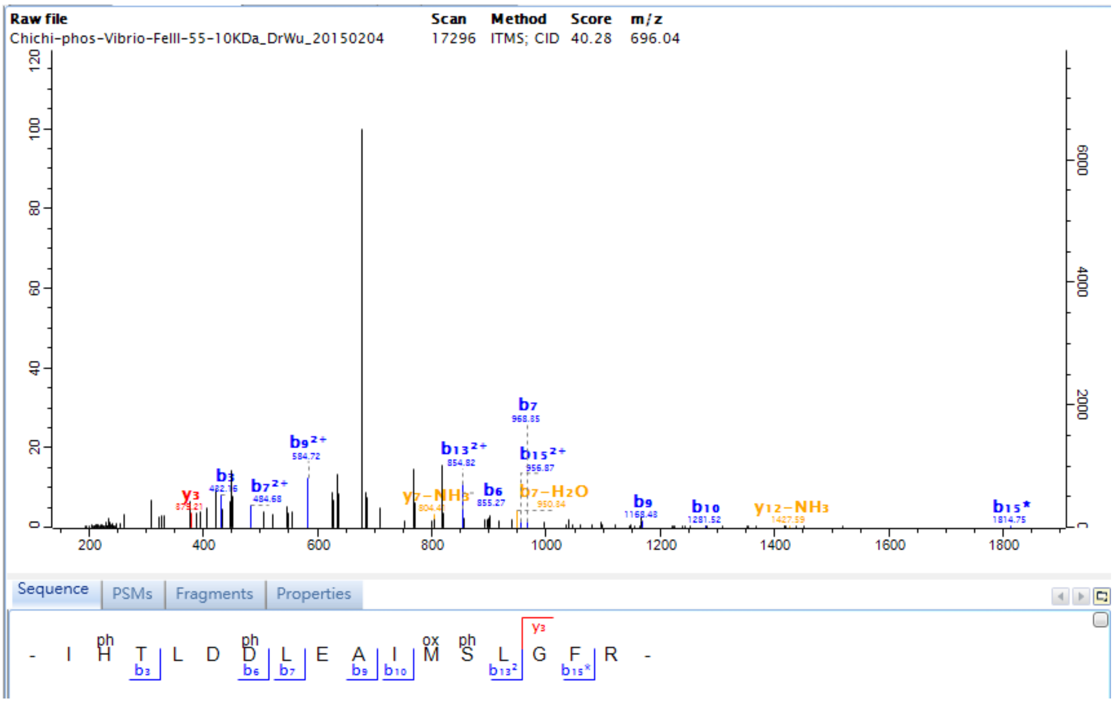
Raw file
Chichi-phos-Vibrio-FeIII-55-10KDa_DrWu_20150204
Scan Method Score m/z
17296 ITMS; CID 40.28 696.04
b7
b9 2+
b13 2+
b15 2+
b3
b7 2+
y3
y7-NH3
b6
b7-H2O
b9
b10
y12-NH3
b15*
Sequence
PSMs
Fragments
Properties
- I H T L D D L E A I M S L G F R -

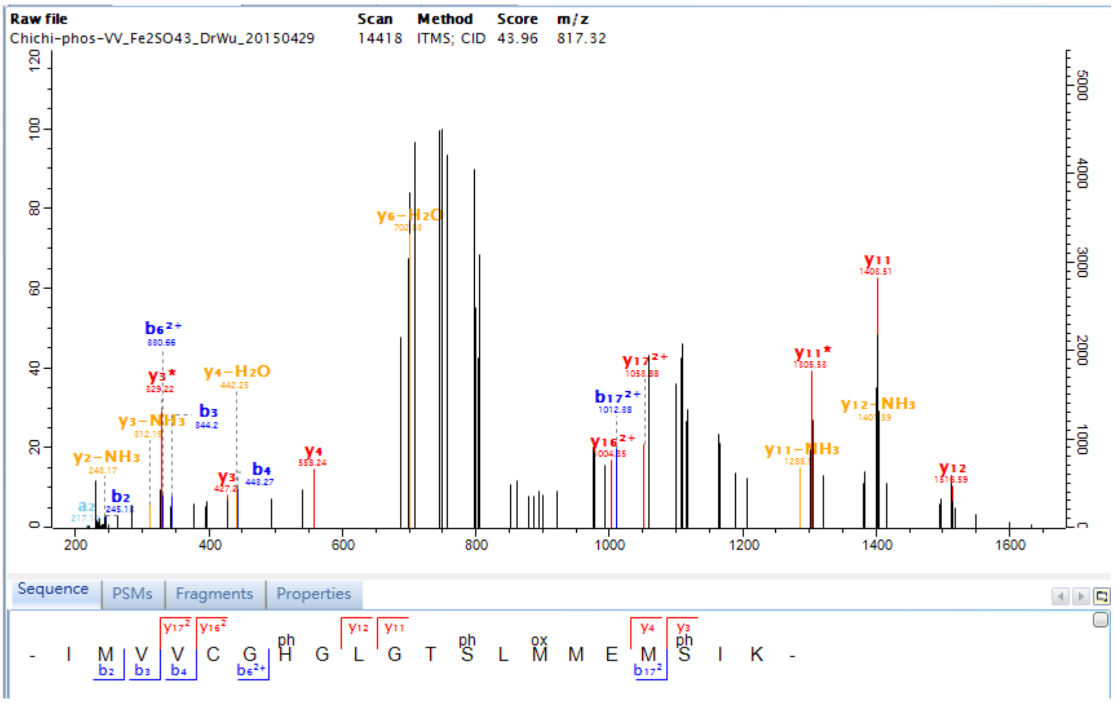
Raw file
Chichi-phos-VV_Fe2SO43_DrWu_20150429
Scan Method Score m/z
14418 ITMS; CID 43.96 817.32
Sequence PSMs Fragments Properties
- I M V V C G H G L G T S L M M E M S I K -

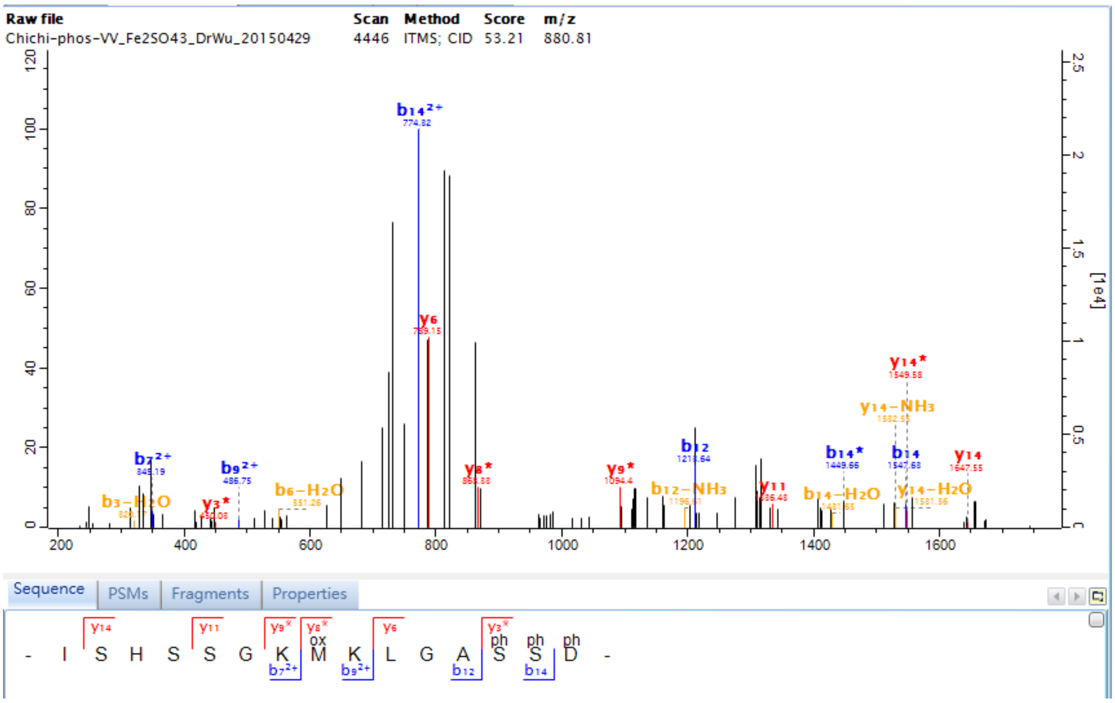
Raw file
Chichi-phos-VV_Fe2SO43_DrWu_20150429
Scan Method Score m/z
4446 ITMS; CID 53.21 880.81
Sequence PSMs Fragments Properties
- I S H S S G K M K L G A S S D -

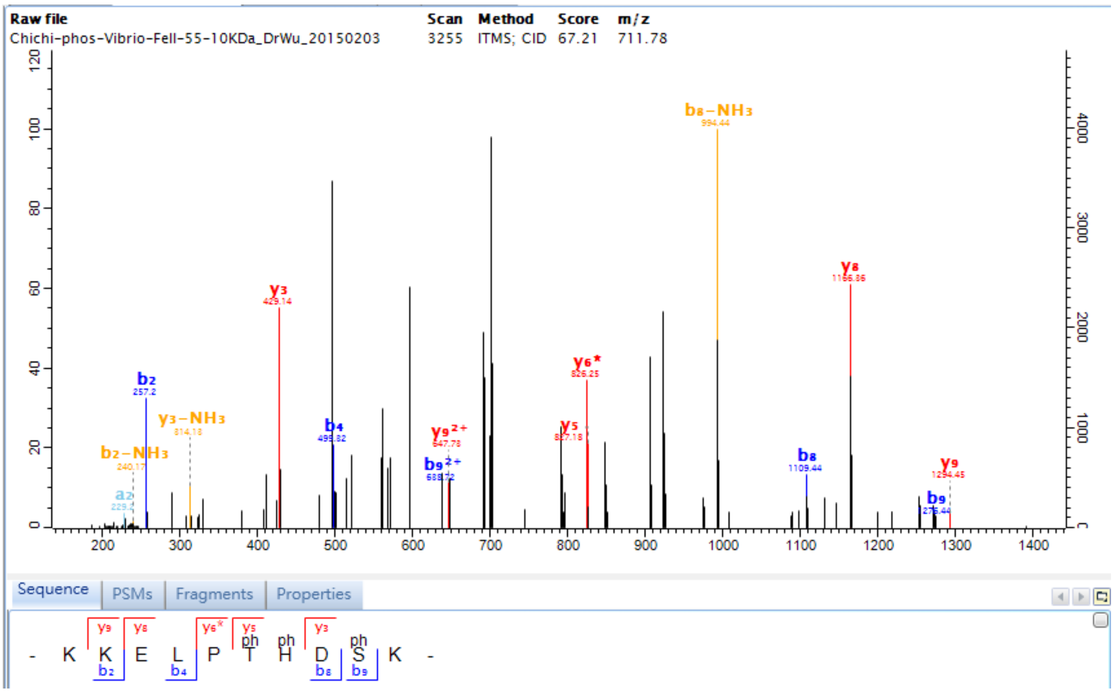
Raw file
Chichi-phos-Vibrio-FeII-55-10KDa_DrWu_20150203
Scan Method Score m/z
3255 ITMS; CID 67.21 711.78
Sequence
PSMs
Fragments
Properties
- K K E L P T H D S K -

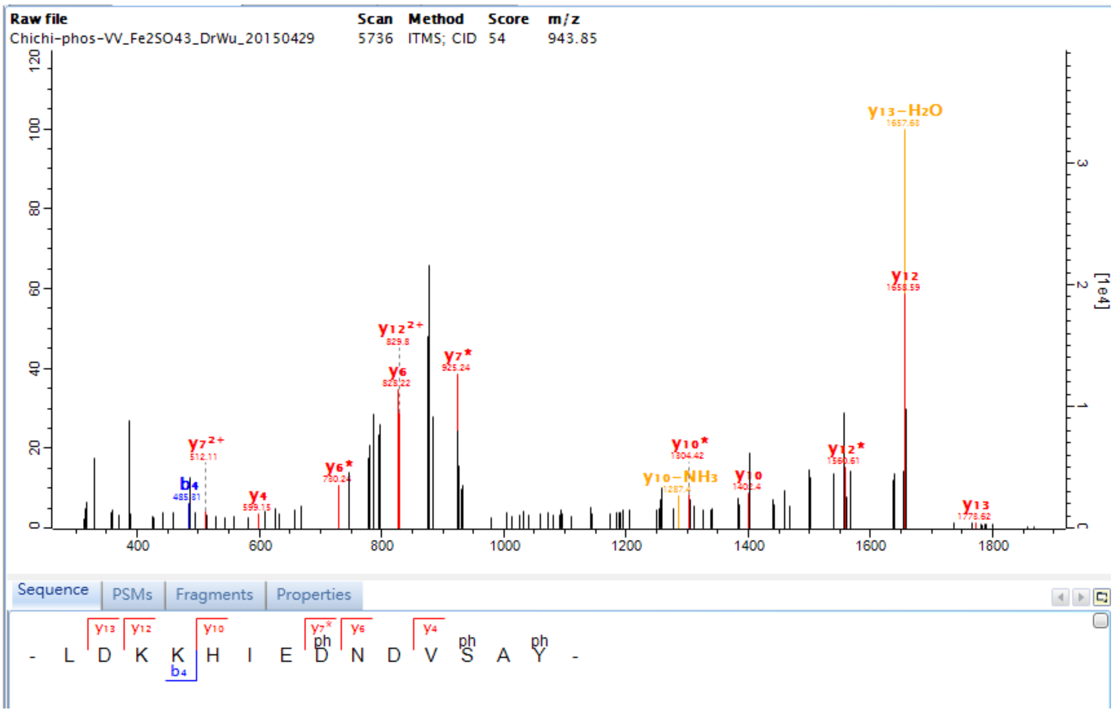
Raw file
Chichi-phos-VV_Fe2SO43_DrWu_20150429
Scan Method Score m/z
5736 ITMS; CID 54 943.85
Sequence
PSMs
Fragments
Properties
- L D K K H I E D N D V S A Y -

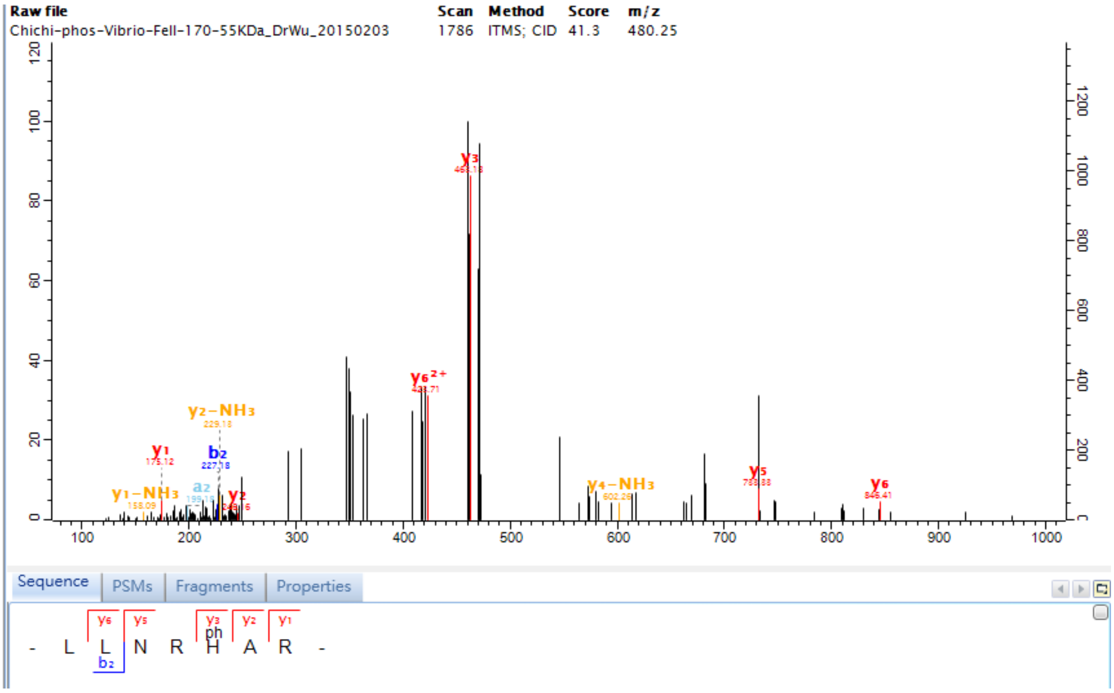
Raw file
Chichi-phos-Vibrio-FeII-170-55KDa_DrWu_20150203
Scan 1786
Method ITMS; CID
Score 41.3
m/z 480.25
Sequence
PSMs
Fragments
Properties
- L L N R H A R -

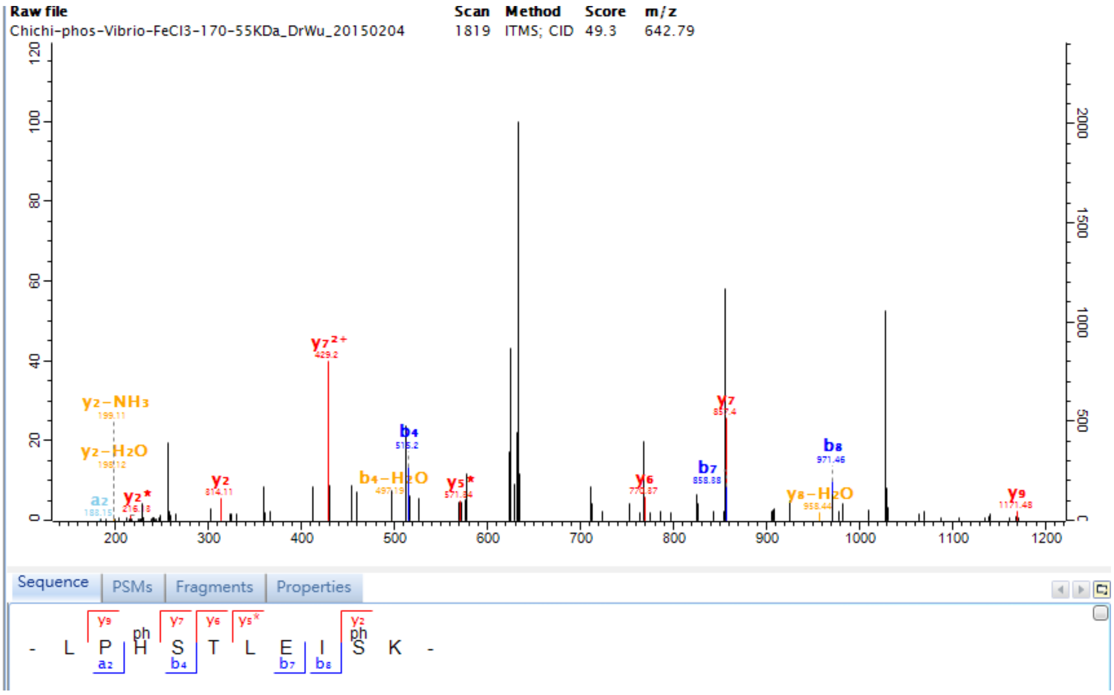
Raw file
Chichi-phos-Vibrio-FeCl3-170-55KDa_DrWu_20150204
Scan 1819
Method ITMS; CID
Score 49.3
m/z 642.79
Sequence
PSMs
Fragments
Properties
- L P H S T L E I S K -

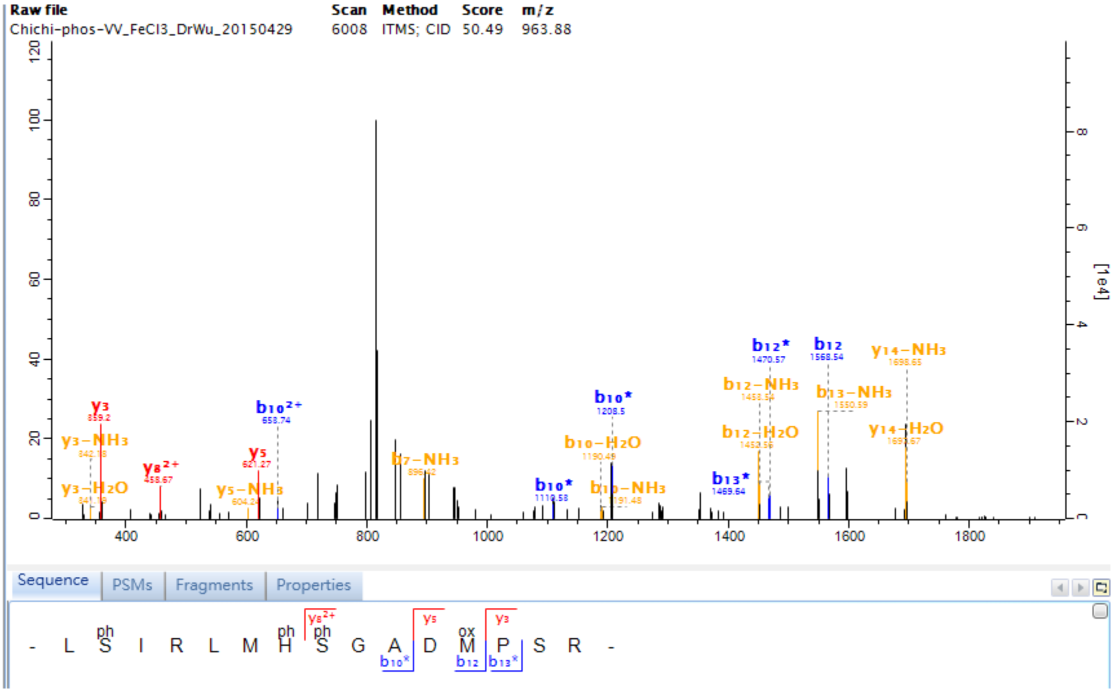
Raw file
Chichi-phos-VV_FeCl3_DrWu_20150429
Scan 6008
Method ITMS; CID
Score 50.49
m/z 963.88
[1e4]
y3
y3-NH3
y3-H2O
y8 2+
y5
y5-NH3
b10 2+
b7-NH3
b10*
b10-H2O
b10-NH3
b13*
b12*
b12-NH3
b12-H2O
b12
b13-NH3
y14-NH3
y14-H2O
Sequence
PSMs
Fragments
Properties
- L S I R L M H S G A D M P S R -

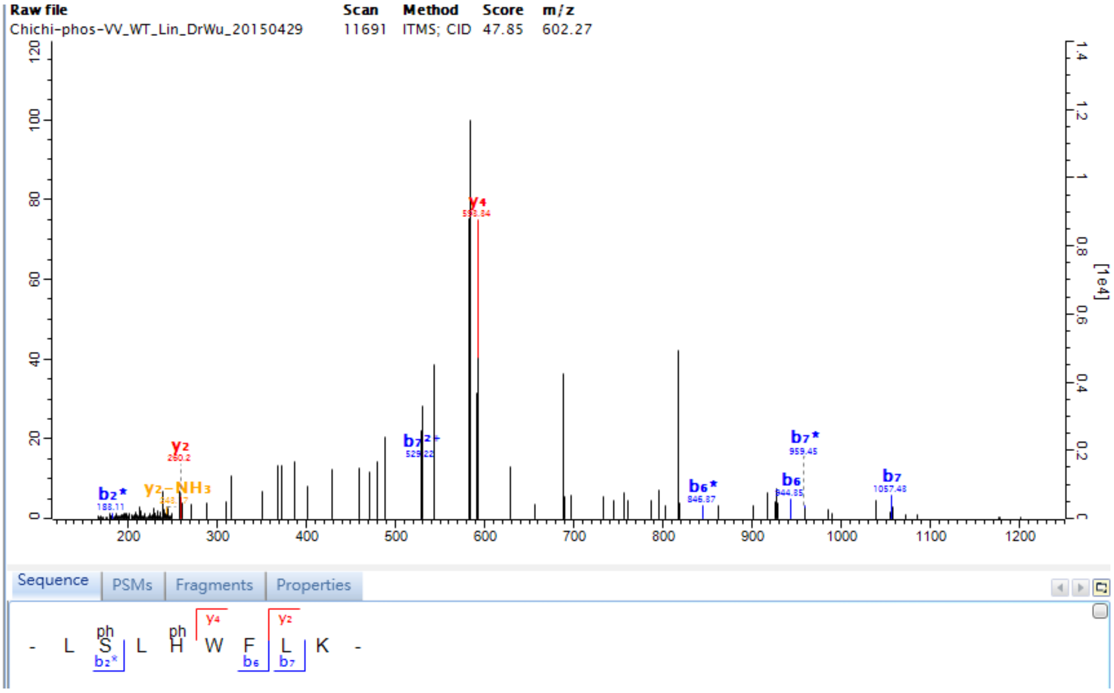
Raw file
Chichi-phos-VV_WT_Lin_DrWu_20150429
Scan 11691
Method ITMS; CID
Score 47.85
m/z 602.27
[1e4]
y4
y2
y2-NH3
b2*
b7 2+
b6*
b6
b7*
b7
Sequence
PSMs
Fragments
Properties
- L S L H W F L K -

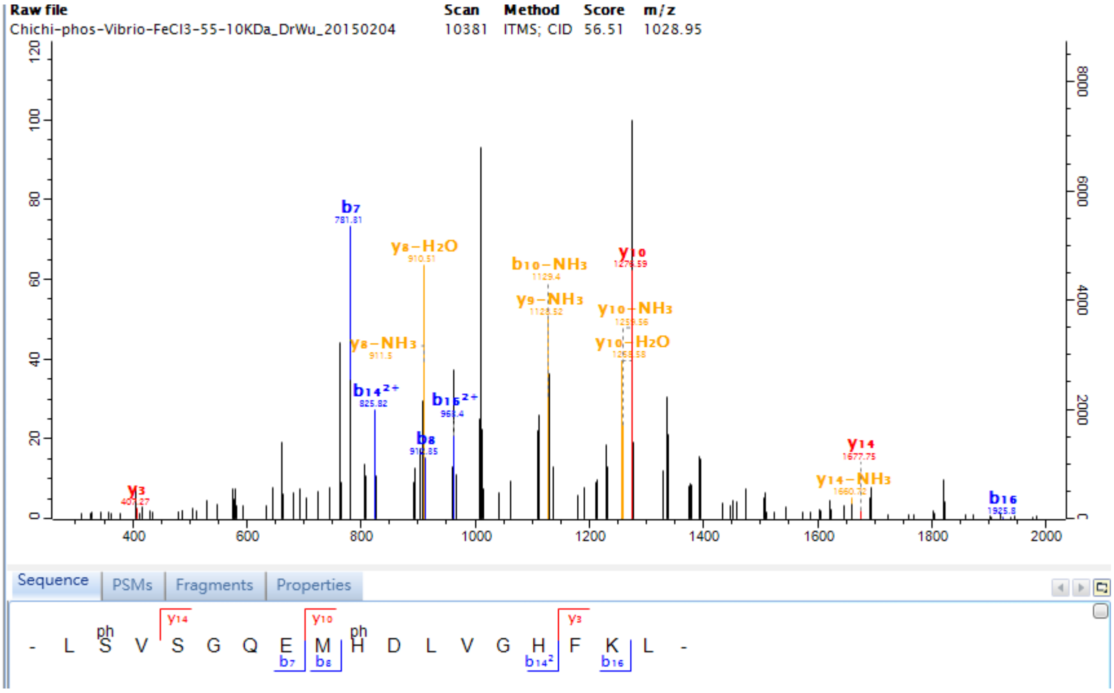
Raw file
Chichi-phos-Vibrio-FeCl3-55-10KDa_DrWu_20150204
Scan
10381
Method
ITMS; CID
Score
56.51
m/z
1028.95
b7
y8-H2O
b10-NH3
y10
y9-NH3
y10-NH3
y8-NH3
y10-H2O
b14 2+
b15 2+
b8
y14
y14-NH3
y3
b16
Sequence
PSMs
Fragments
Properties
- L S V S G Q E M H D L V G H F K L -

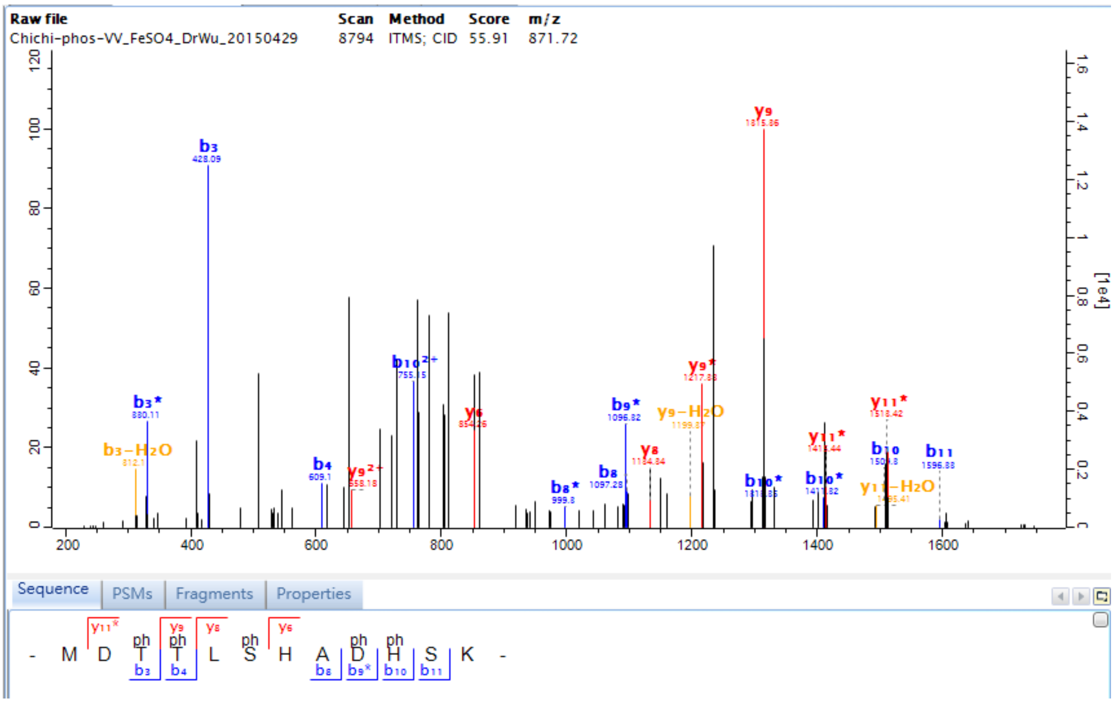
Raw file
Chichi-phos-VV_FeSO4_DrWu_20150429
Scan
8794
Method
ITMS; CID
Score
55.91
m/z
871.72
y9
b3
b10 2+
y9*
b3*
y6
b9*
y9-H2O
b3-H2O
b4
y9 2+
y8
b8
b8*
y11*
y11*
b10
b11
b10*
b10*
y11-H2O
[1e4]
Sequence
PSMs
Fragments
Properties
- M D T T L S H A D H S K -

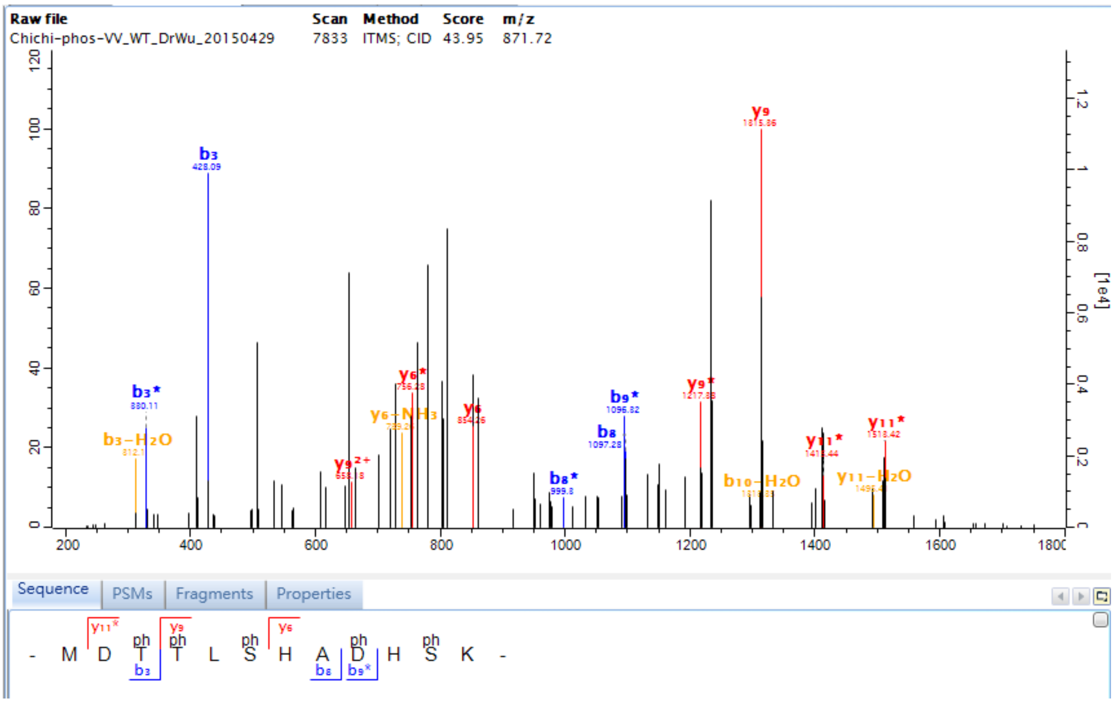
Raw file
Chichi-phos-VV_WT_DrWu_20150429
Scan Method Score m/z
7833 ITMS; CID 43.95 871.72
y9
b3
b3*
b3-H2O
y6*
y6-NH3
y6
y9 2+
b9*
b8
b8*
y9*
b10-H2O
y11*
y11-H2O
[1e4]
Sequence
PSMs
Fragments
Properties
- M D T T L S H A D H S K -

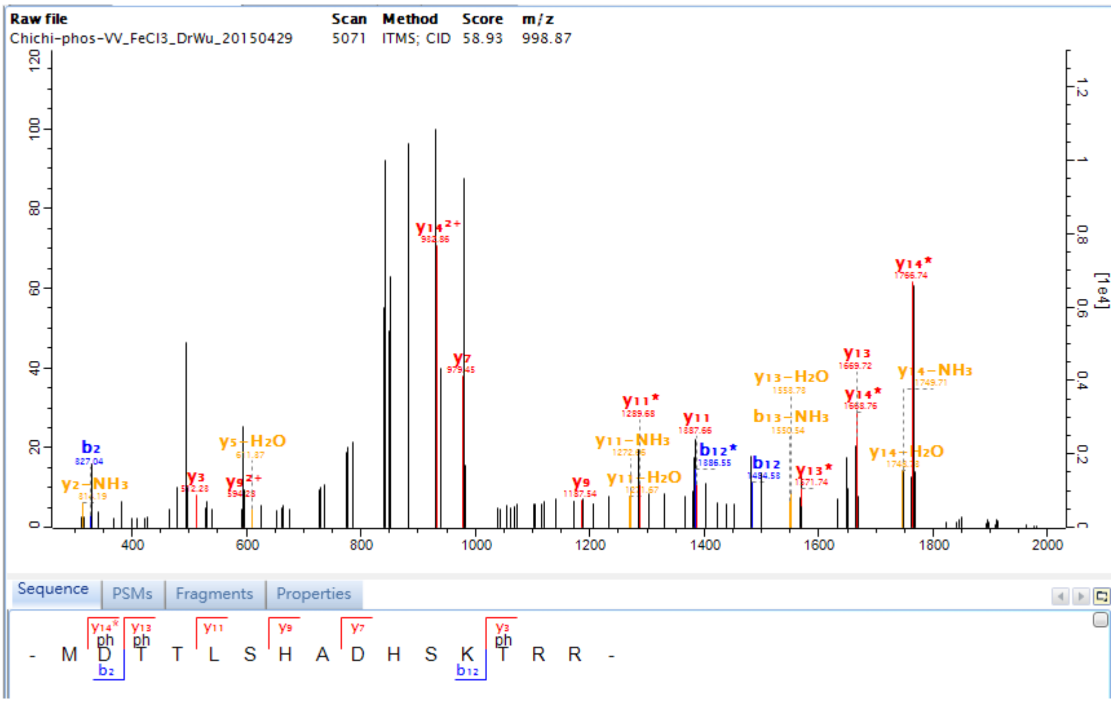
Raw file
Chichi-phos-VV_FeCl3_DrWu_20150429
Scan Method Score m/z
5071 ITMS; CID 58.93 998.87
y14 2+
y7
y14*
y13
y13-H2O
y14-NH3
b13-NH3
y11*
y11
y11-NH3
y11-H2O
b12*
b12
y13*
y14-H2O
y9
b2
y2-NH3
y3
y5-H2O
y9 2+
[1e4]
Sequence
PSMs
Fragments
Properties
- M D T T L S H A D H S K T R R -

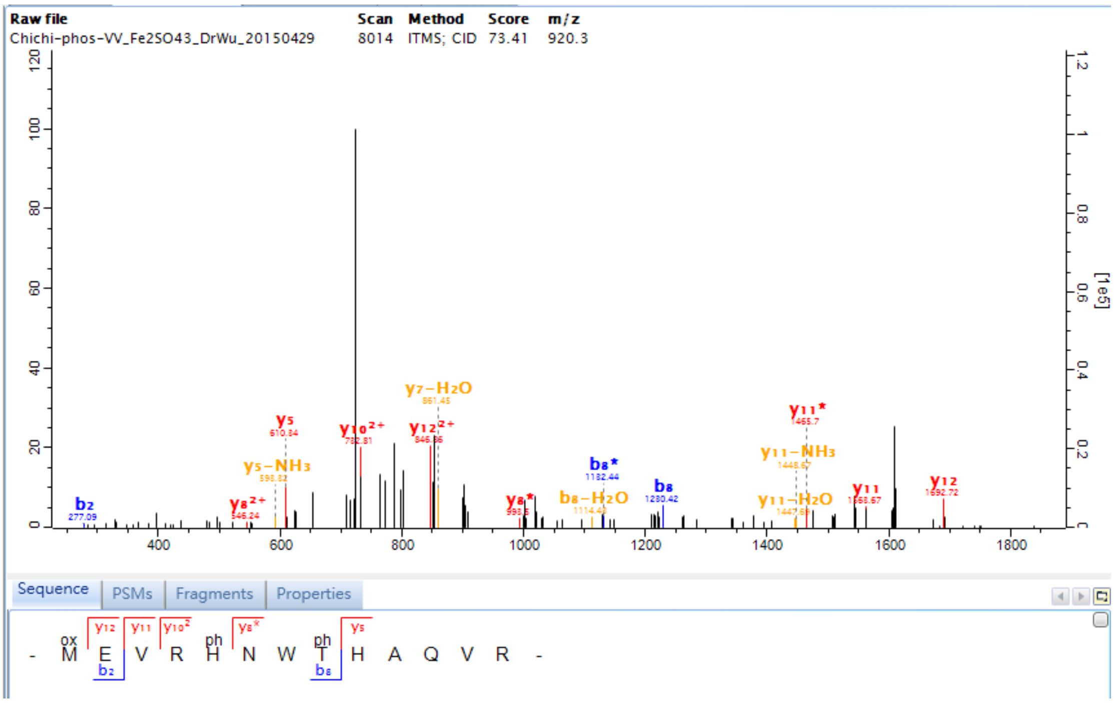
Raw file
Chichi-phos-VV_Fe2SO43_DrWu_20150429
Scan Method Score m/z
8014 ITMS; CID 73.41 920.3
Sequence PSMs Fragments Properties
- M E V R H N W T H A Q V R -

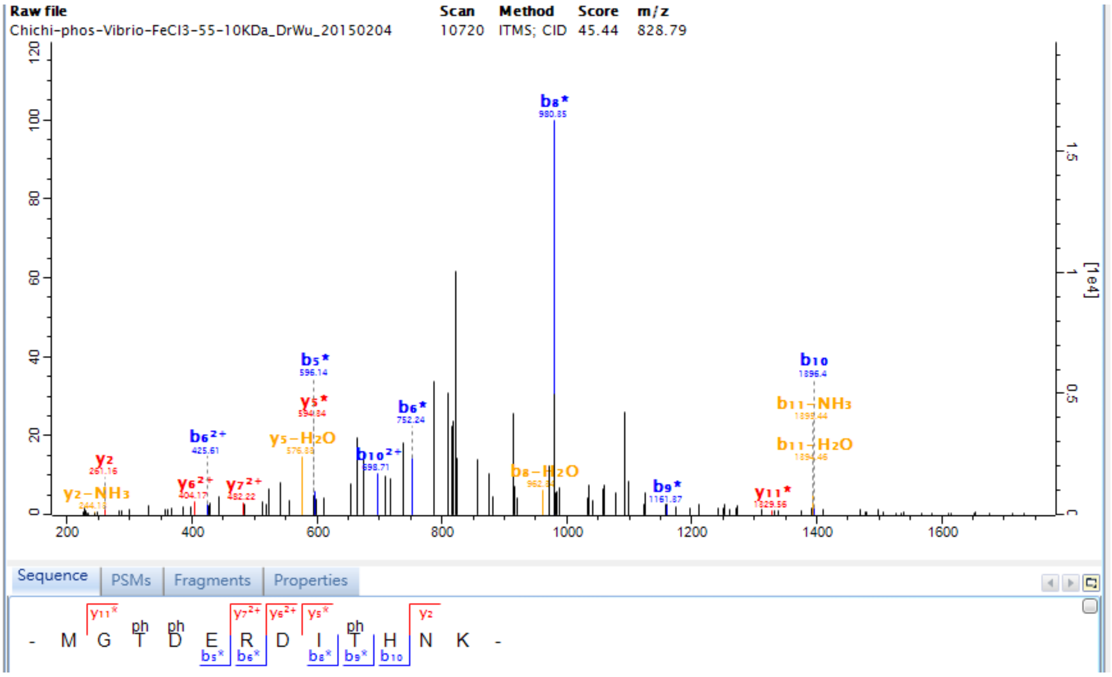
Raw file
Chichi-phos-Vibrio-FeCl3-55-10KDa_DrWu_20150204
Scan Method Score m/z
10720 ITMS; CID 45.44 828.79
Sequence PSMs Fragments Properties
- M G T D E R D I T H N K -

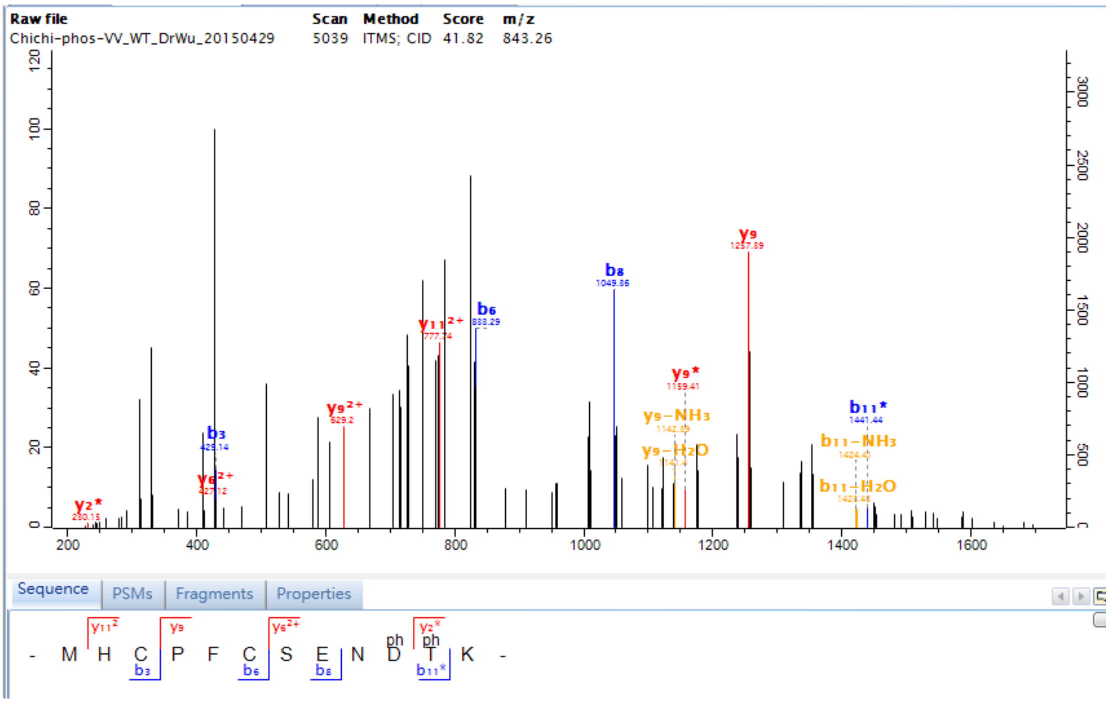
Raw file
Chichi-phos-VV_WT_DrWu_20150429
Scan
5039
Method
ITMS; CID
Score
41.82
m/z
843.26
Sequence
PSMs
Fragments
Properties
- M H C P F C S E N D T K -

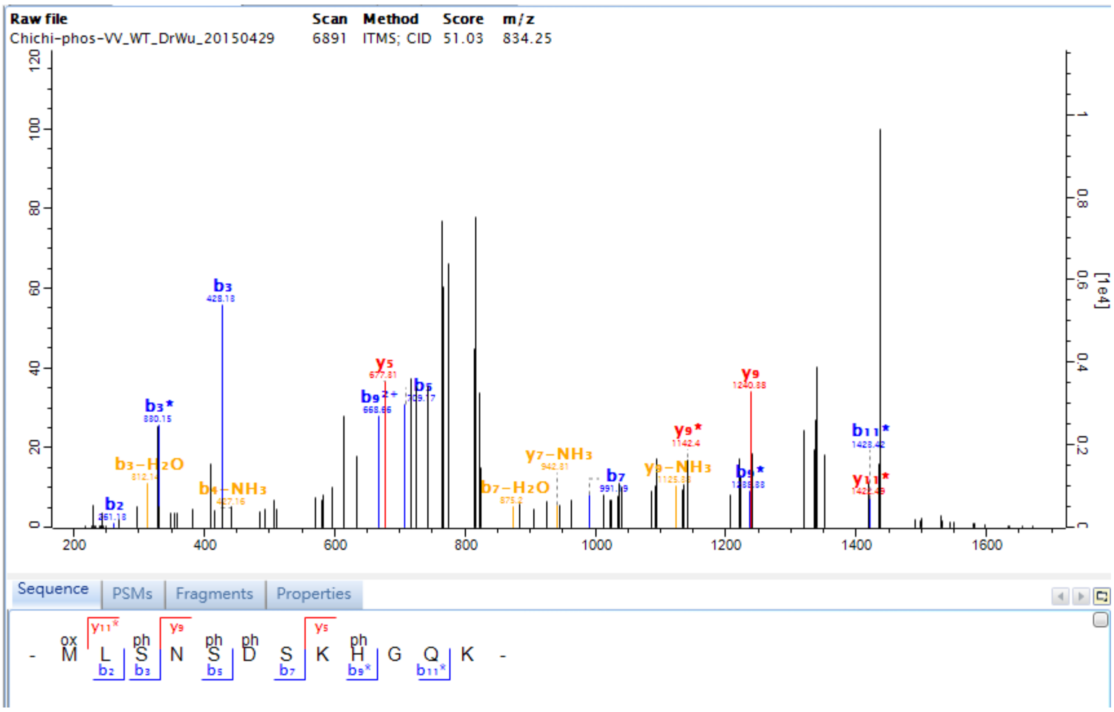
Raw file
Chichi-phos-VV_WT_DrWu_20150429
Scan
6891
Method
ITMS; CID
Score
51.03
m/z
834.25
Sequence
PSMs
Fragments
Properties
- M L S N S D S K H G Q K -

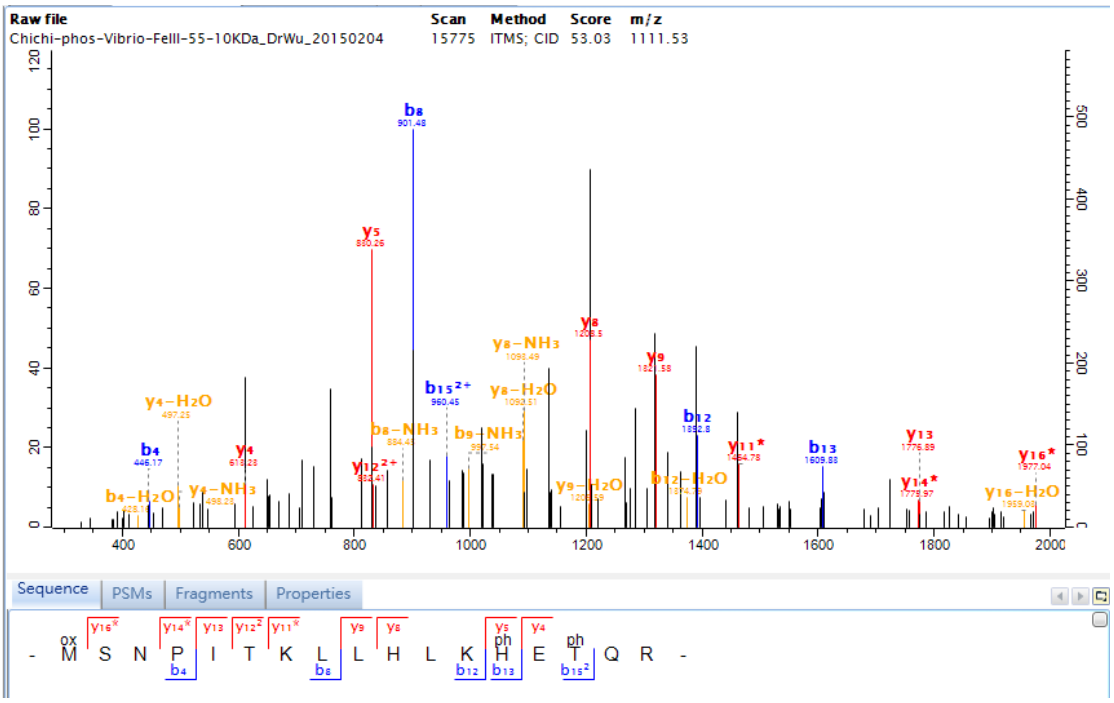
Raw file
Chichi-phos-Vibrio-FeIII-55-10KDa_DrWu_20150204
Scan 15775
Method ITMS; CID
Score 53.03
m/z 1111.53
Sequence
PSMs
Fragments
Properties
- M S N P I T K L L H L K H E T Q R -

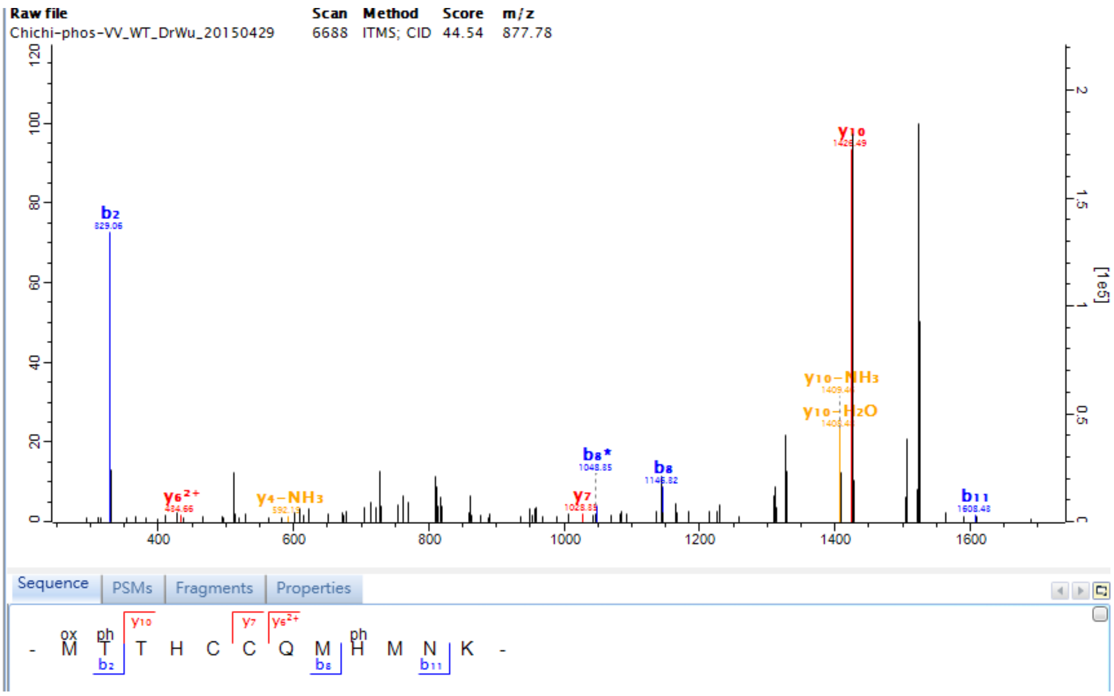
Raw file
Chichi-phos-VV_WT_DrWu_20150429
Scan 6688
Method ITMS; CID
Score 44.54
m/z 877.78
[1e5]
Sequence
PSMs
Fragments
Properties
- M T T H C C Q M H M N K -

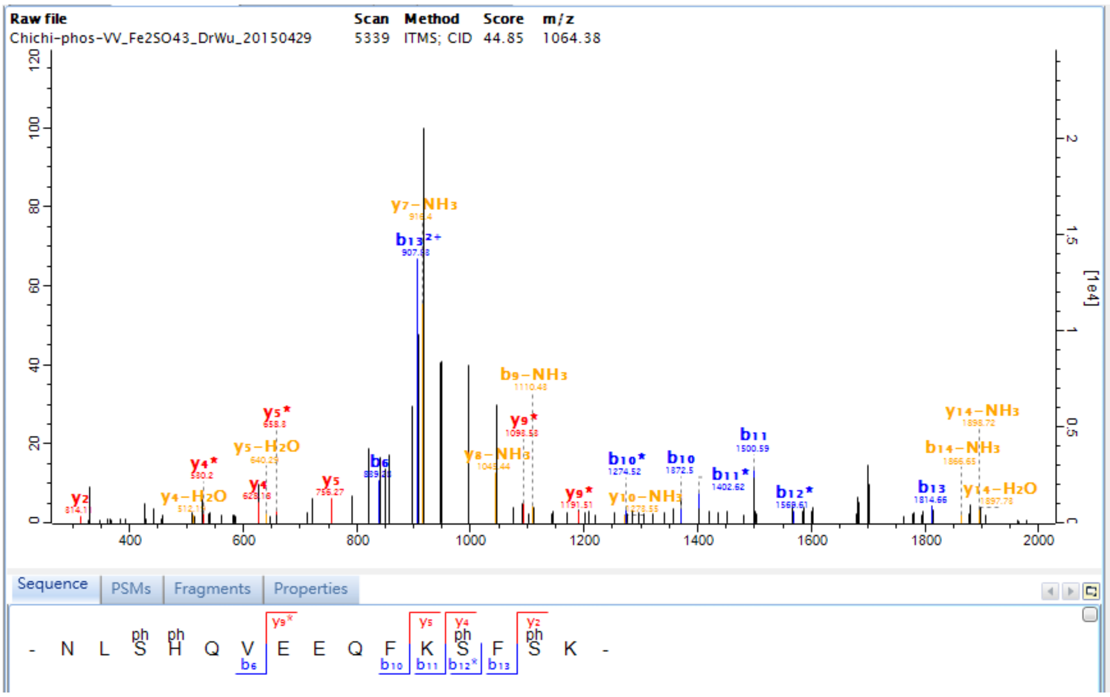
Raw file
Chichi-phos-VV_Fe2SO43_DrWu_20150429
Scan
5339
Method
ITMS; CID
Score
44.85
m/z
1064.38
Sequence
PSMs
Fragments
Properties
- N L S H Q V E E Q F K S F S K -

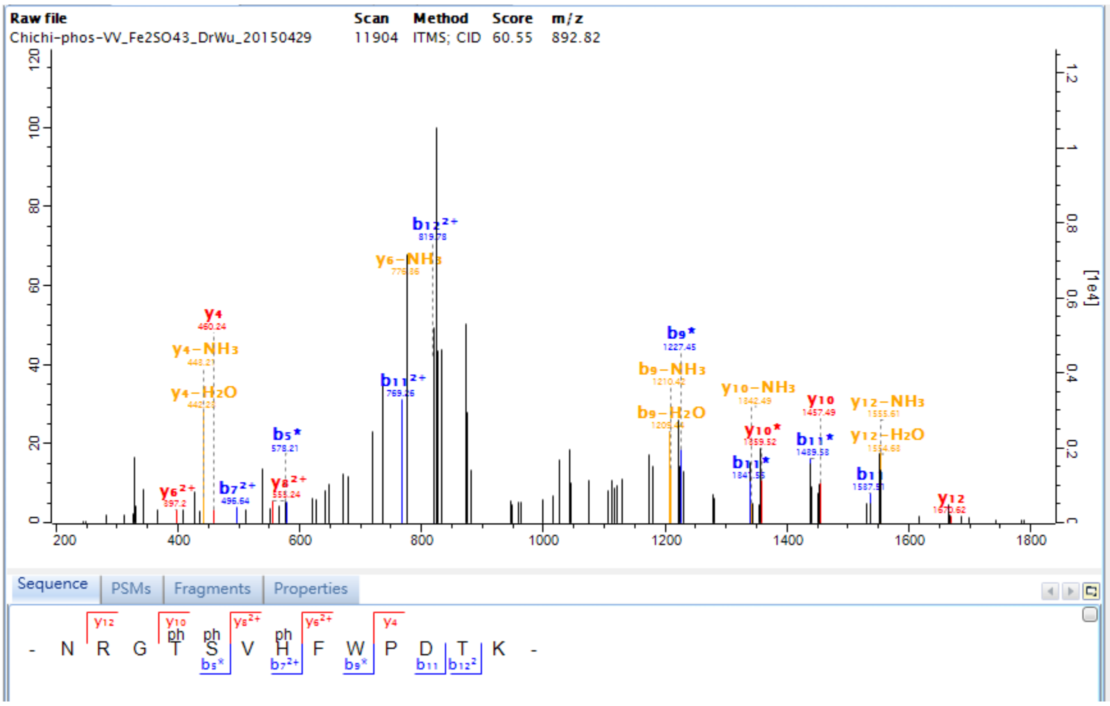
Raw file
Chichi-phos-VV_Fe2SO43_DrWu_20150429
Scan
11904
Method
ITMS; CID
Score
60.55
m/z
892.82
Sequence
PSMs
Fragments
Properties
- N R G T S V H F W P D T K -

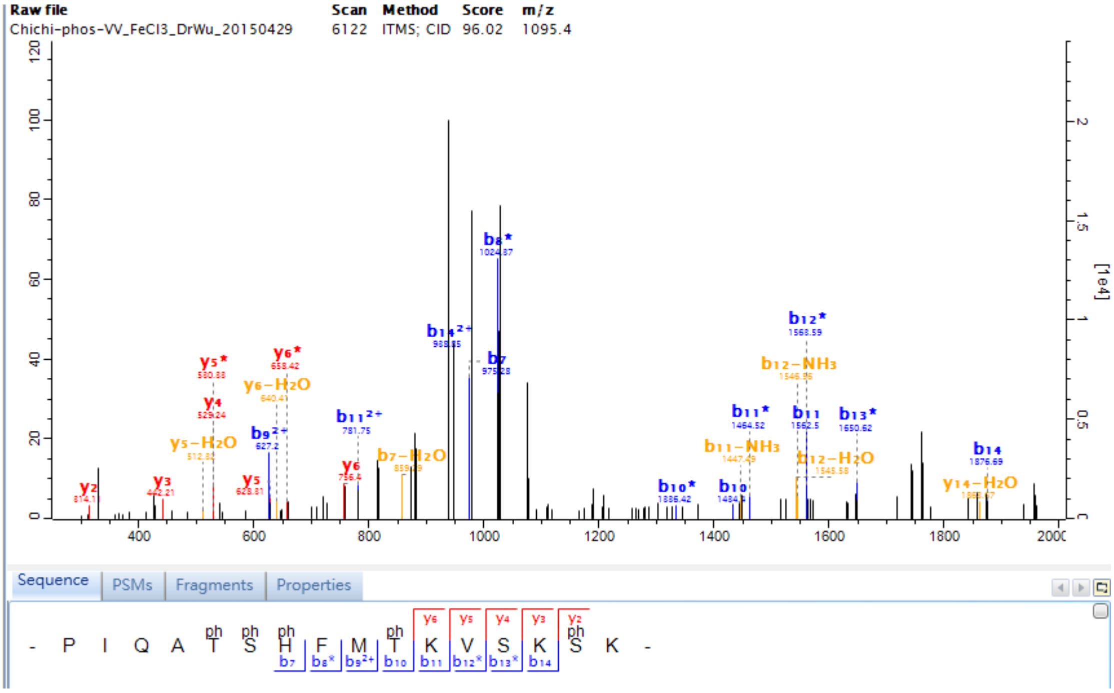
Raw file
Chichi-phos-VV_FeCl3_DrWu_20150429
Scan Method Score m/z
6122 ITMS; CID 96.02 1095.4
Sequence
PSMs
Fragments
Properties
- P I Q A T S H F M T K V S K S K -

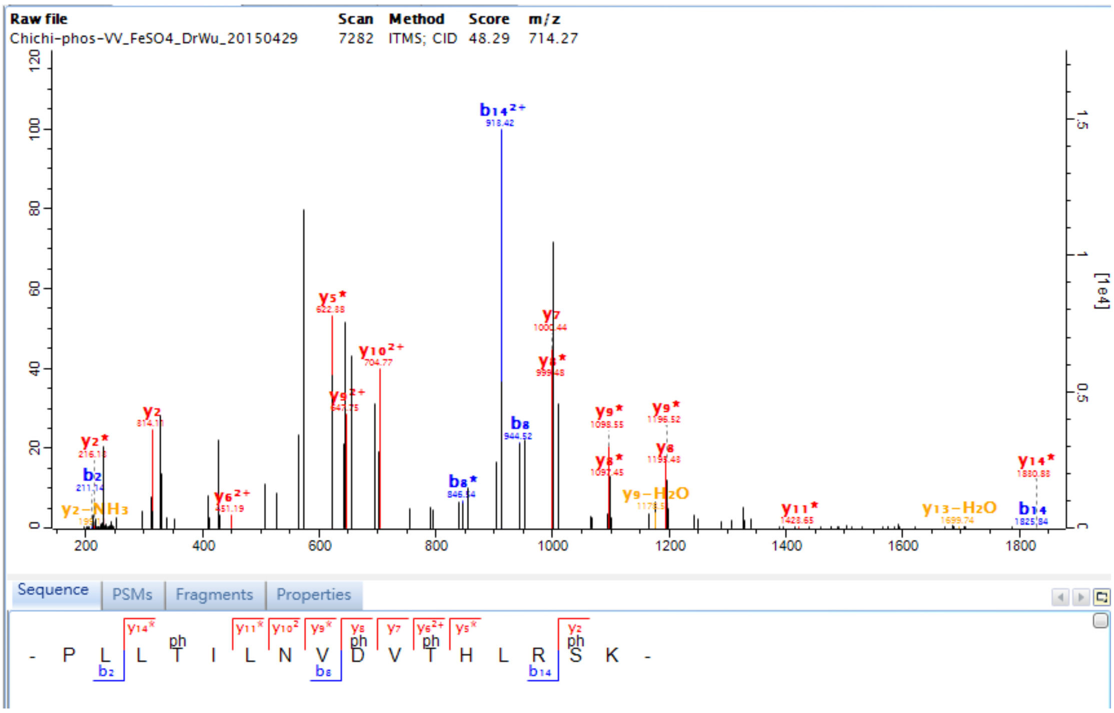
Raw file
Chichi-phos-VV_FeSO4_DrWu_20150429
Scan Method Score m/z
7282 ITMS; CID 48.29 714.27
Sequence
PSMs
Fragments
Properties
- P L L T I L N V D V T H L R S K -

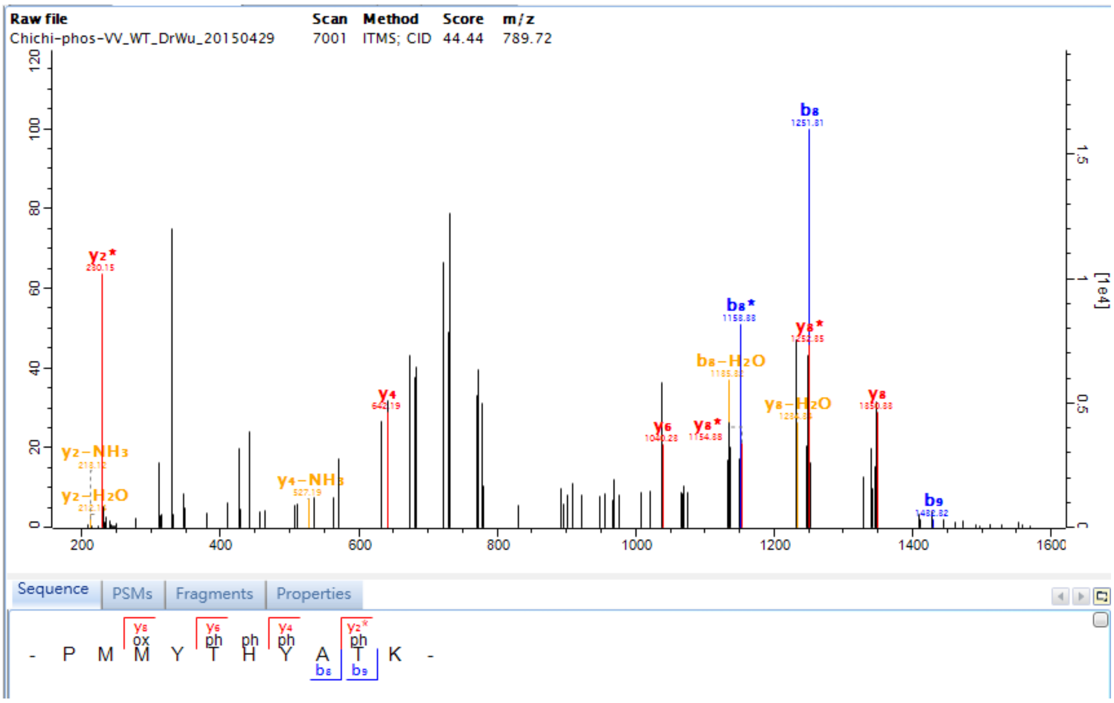
Raw file
Chichi-phos-VV_WT_DrWu_20150429
Scan Method Score m/z
7001 ITMS; CID 44.44 789.72
Sequence PSMs Fragments Properties
- P M M Y T H Y A T K -
ox ph ph ph ph

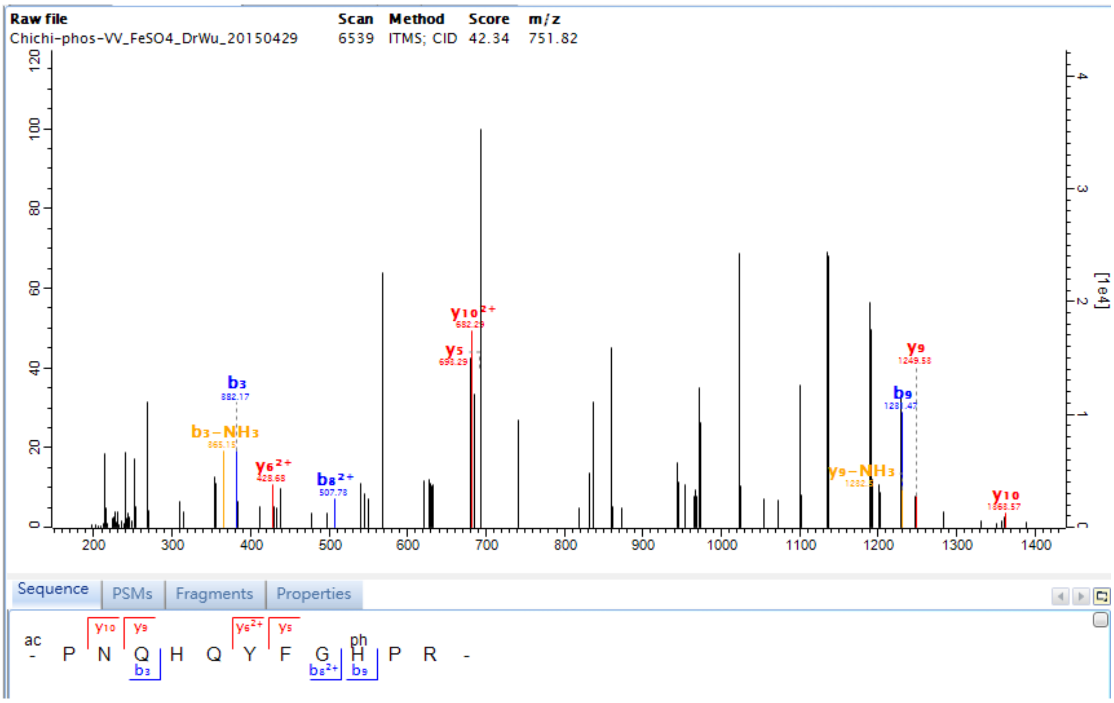
Raw file
Chichi-phos-VV_FeSO4_DrWu_20150429
Scan Method Score m/z
6539 ITMS; CID 42.34 751.82
Sequence PSMs Fragments Properties
ac - P N Q H Q Y F G H P R -
ph

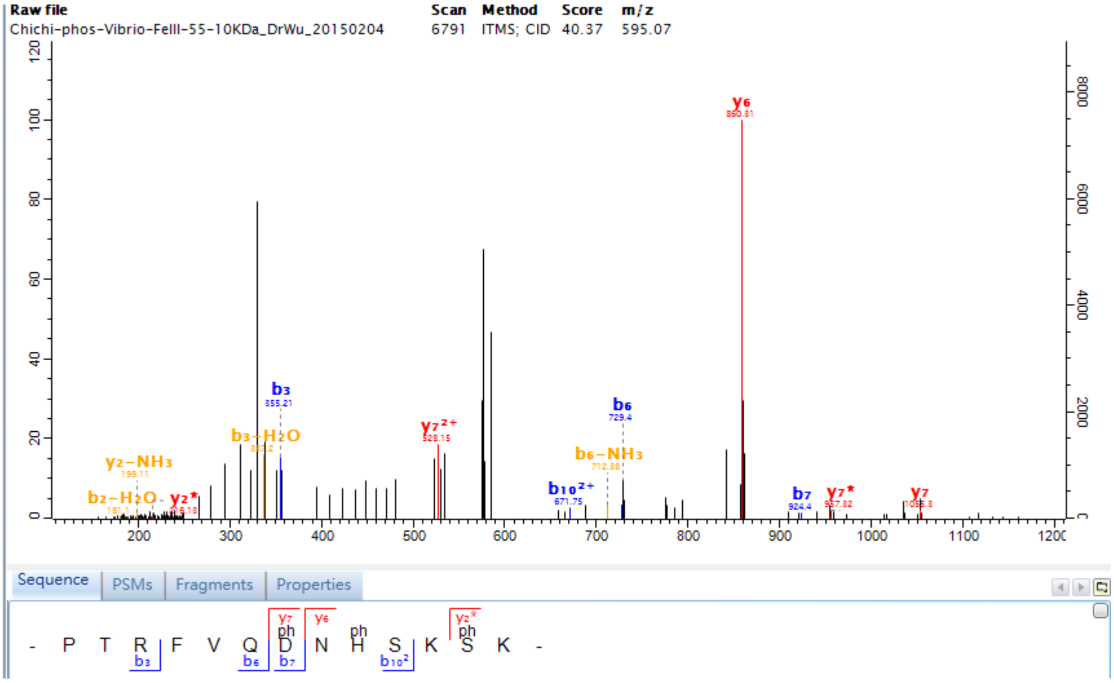
Raw file
Chichi-phos-Vibrio-FeIII-55-10KDa_DrWu_20150204
Scan Method Score m/z
6791 ITMS; CID 40.37 595.07
y6
b3
b3-H2O
y7 2+
b6
b6-NH3
b10 2+
y2-NH3
b2-H2O
y2*
b7
y7*
y7
Sequence PSMs Fragments Properties
- P T R F V Q D N H S K S K -

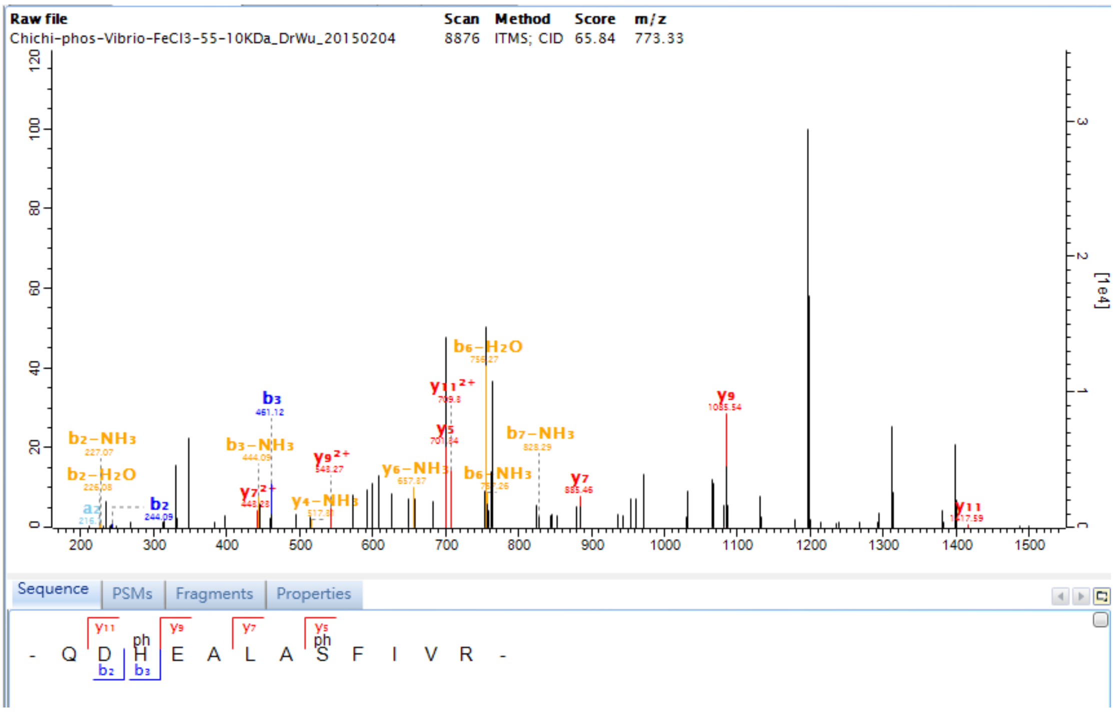
Raw file
Chichi-phos-Vibrio-FeCl3-55-10KDa_DrWu_20150204
Scan Method Score m/z
8876 ITMS; CID 65.84 773.33
[1e4]
b6-H2O
y11 2+
y5
b3
y9
b7-NH3
b2-NH3
b3-NH3
y9 2+
y6-NH3
b6-NH3
y7
b2-H2O
y7 2+
y4-NH3
b2
y11
Sequence PSMs Fragments Properties
- Q D H E A L A S F I V R -

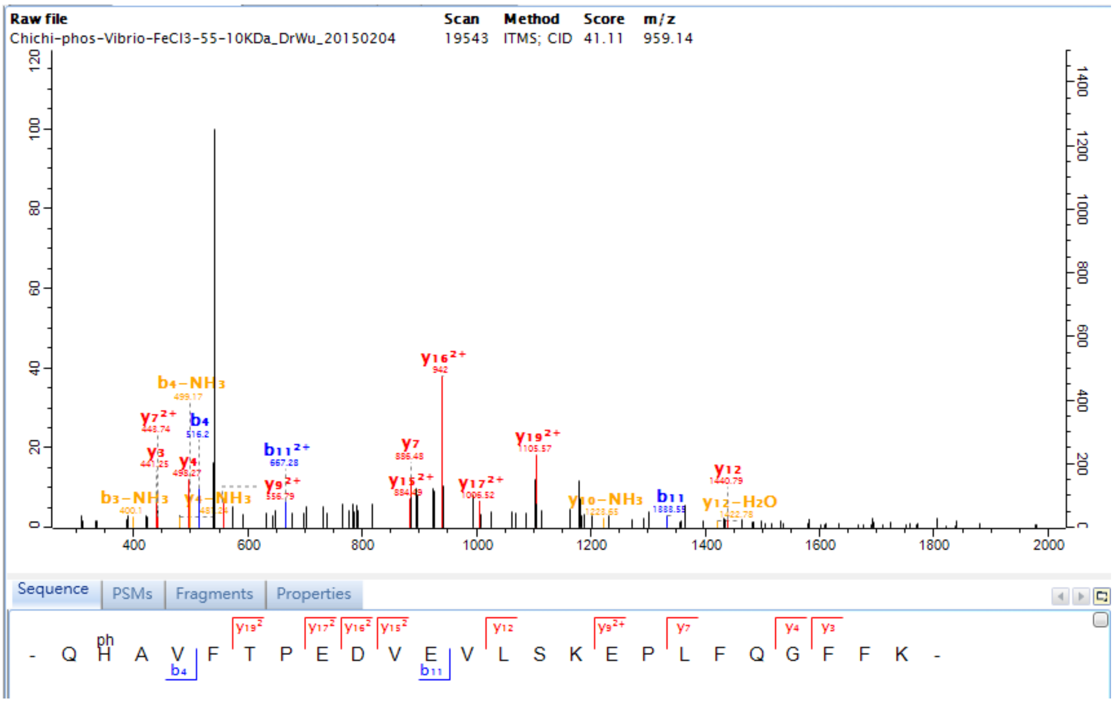

Raw file
Chichi-phos-Vibrio-FeCl3-55-10KDa_DrWu_20150204
Scan 19543
Method ITMS; CID
Score 41.11
m/z 959.14
Sequence
PSMs
Fragments
Properties
- Q H A V F T P E D V E V L S K E P L F Q G F F K -

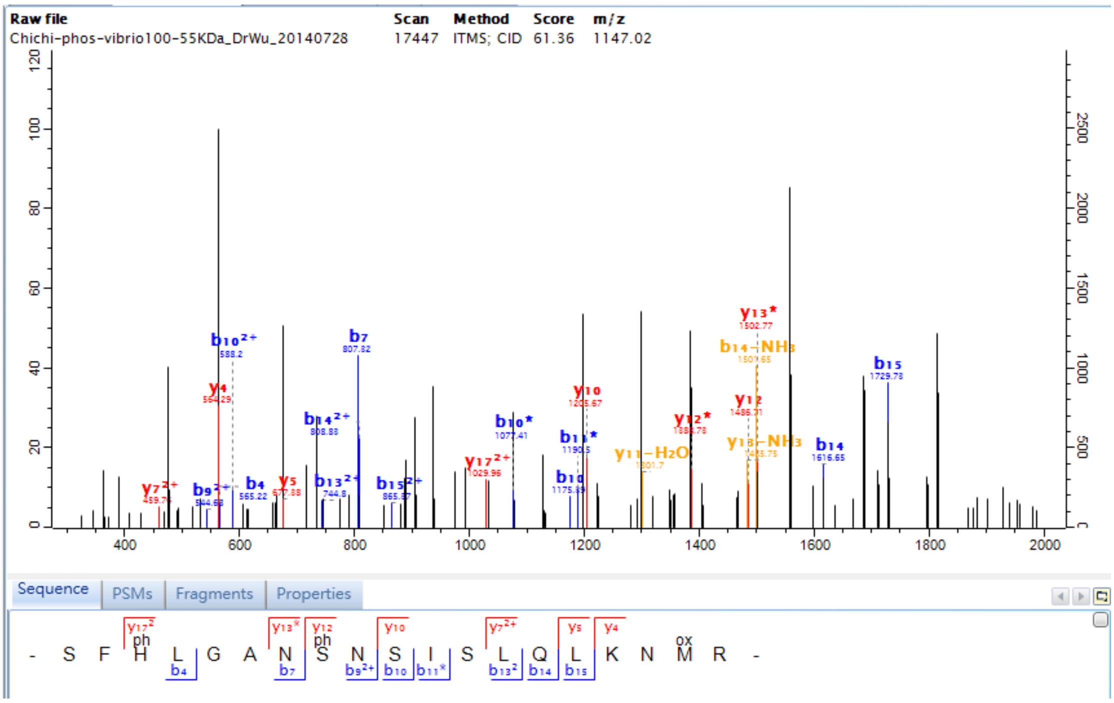

Raw file
Chichi-phos-vibrio100-55KDa_DrWu_20140728
Scan 17447
Method ITMS; CID
Score 61.36
m/z 1147.02
Sequence
PSMs
Fragments
Properties
- S F H L G A N S N S I S L Q L K N M R -

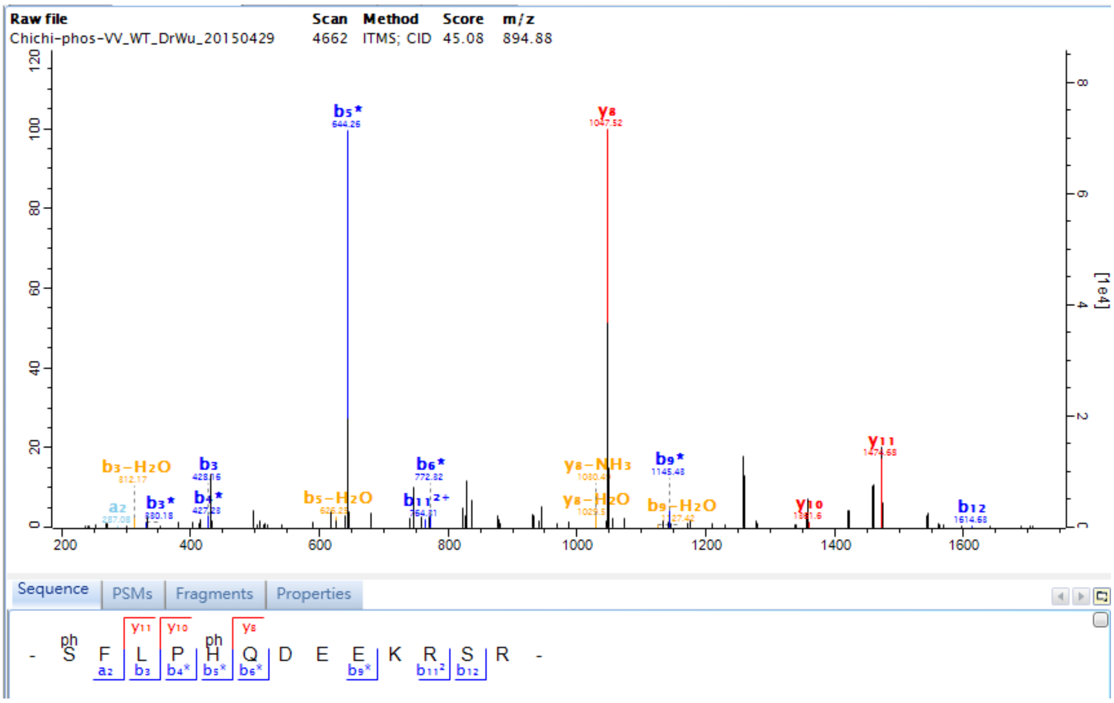
Raw file
Chichi-phos-VV_WT_DrWu_20150429
Scan Method Score m/z
4662 ITMS; CID 45.08 894.88
b5*
y8
b3-H2O
b3
a2
b3*
b4*
b5-H2O
b6*
b11 2+
y8-NH3
y8-H2O
b9*
b9-H2O
y10
y11
b12
[1e4]
Sequence PSMs Fragments Properties
- S F L P H Q D E E K R S R -

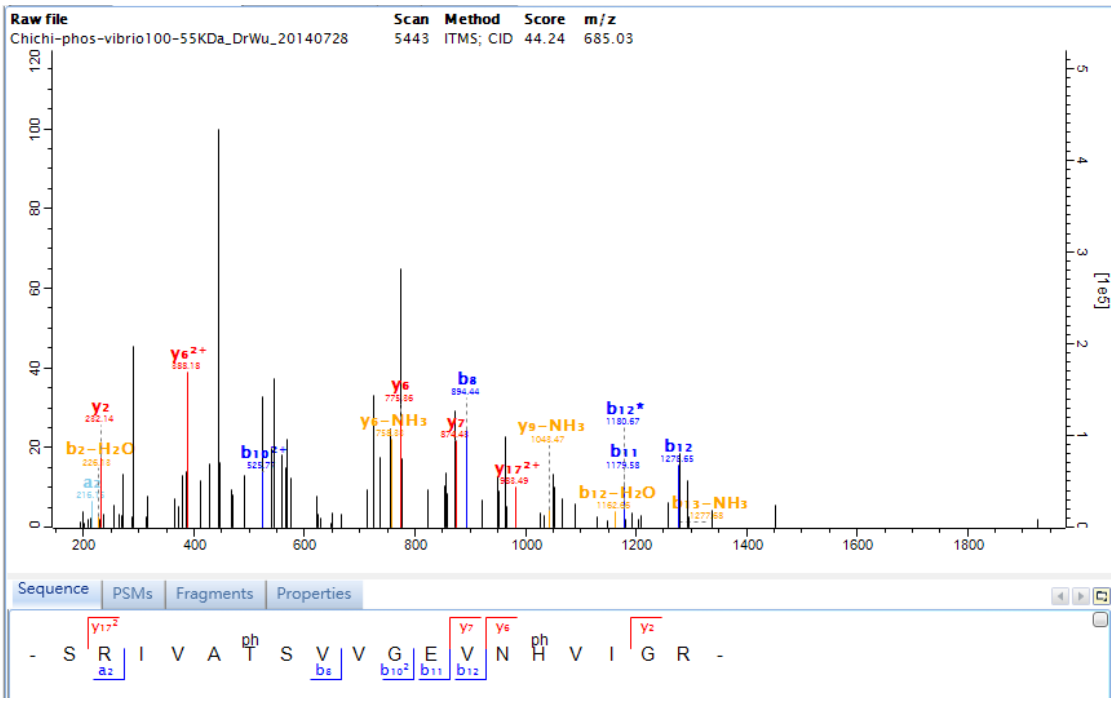
Raw file
Chichi-phos-vibrio100-55KDa_DrWu_20140728
Scan Method Score m/z
5443 ITMS; CID 44.24 685.03
y6 2+
y2
b2-H2O
a2
b10 2+
y6
y6-NH3
y7
b8
y9-NH3
y17 2+
b12*
b11
b12
b12-H2O
b13-NH3
[1e5]
Sequence PSMs Fragments Properties
- S R I V A T S V V G E V N H V I G R -

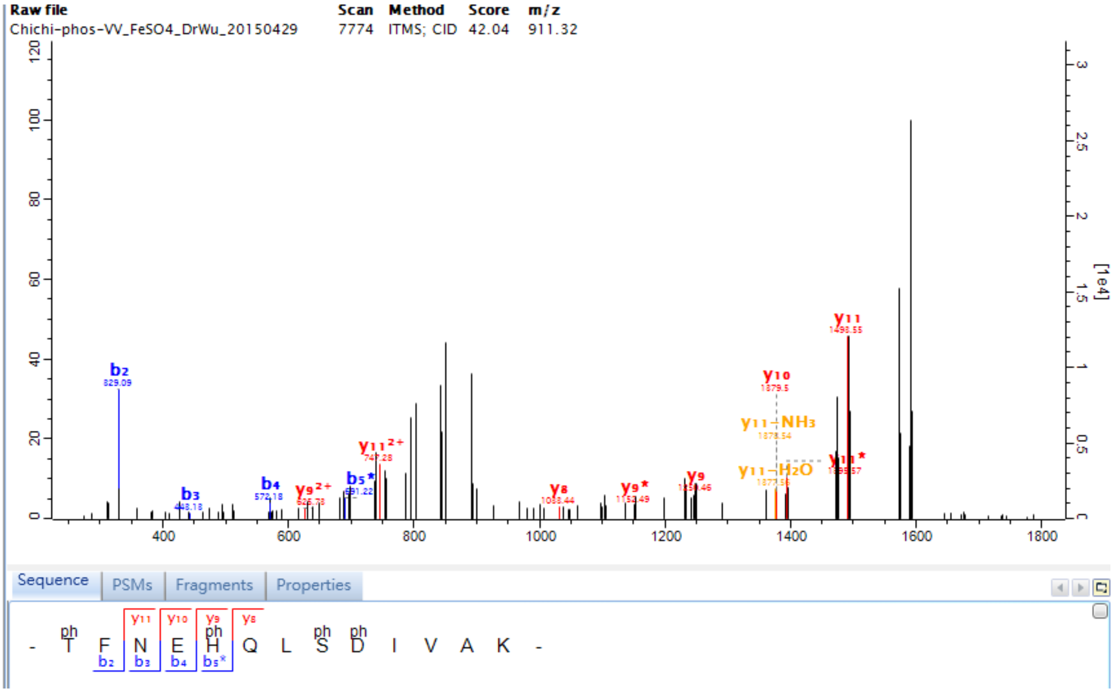
Raw file
Chichi-phos-VV_FeSO4_DrWu_20150429
Scan 7774
Method ITMS; CID
Score 42.04
m/z 911.32
Sequence
PSMs
Fragments
Properties
- T F N E H Q L S D I V A K -

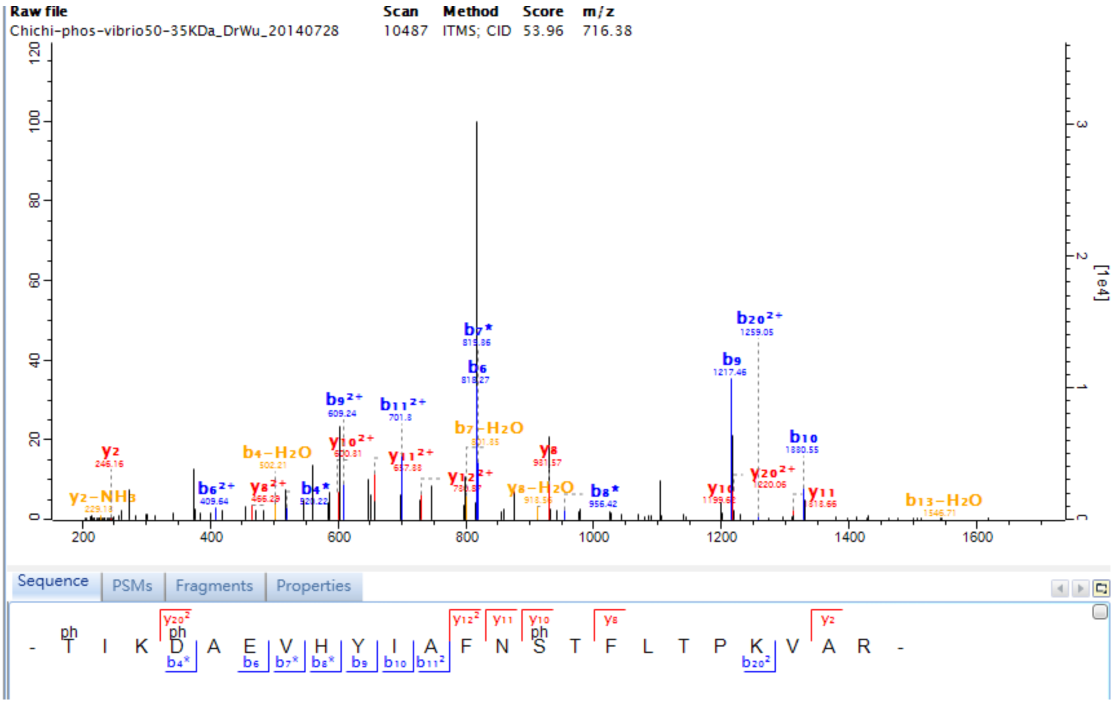
Raw file
Chichi-phos-vibrio50-35KDa_DrWu_20140728
Scan 10487
Method ITMS; CID
Score 53.96
m/z 716.38
Sequence
PSMs
Fragments
Properties
- T I K D A E V H Y I A F N S T F L T P K V A R -

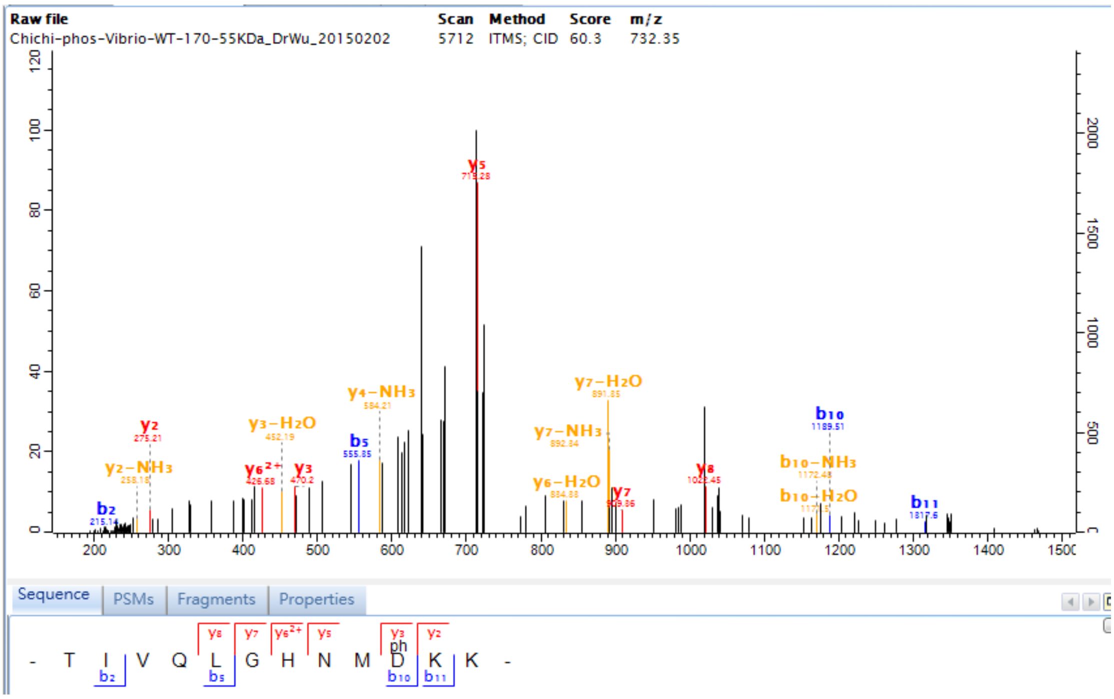
Raw file
Chichi-phos-Vibrio-WT-170-55KDa_DrWu_20150202
Scan Method Score m/z
5712 ITMS; CID 60.3 732.35
Sequence
PSMs
Fragments
Properties
- T I V Q L G H N M D K K -

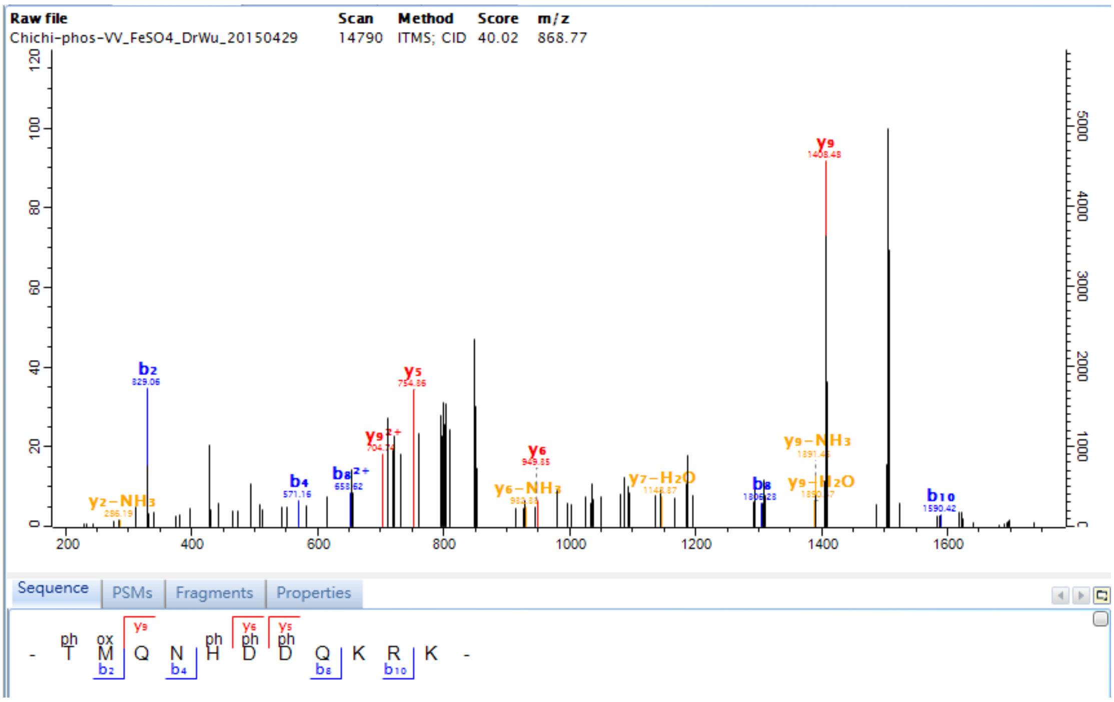
Raw file
Chichi-phos-VV_FeSO4_DrWu_20150429
Scan Method Score m/z
14790 ITMS; CID 40.02 868.77
Sequence
PSMs
Fragments
Properties
- T M Q N H D D Q K R K -

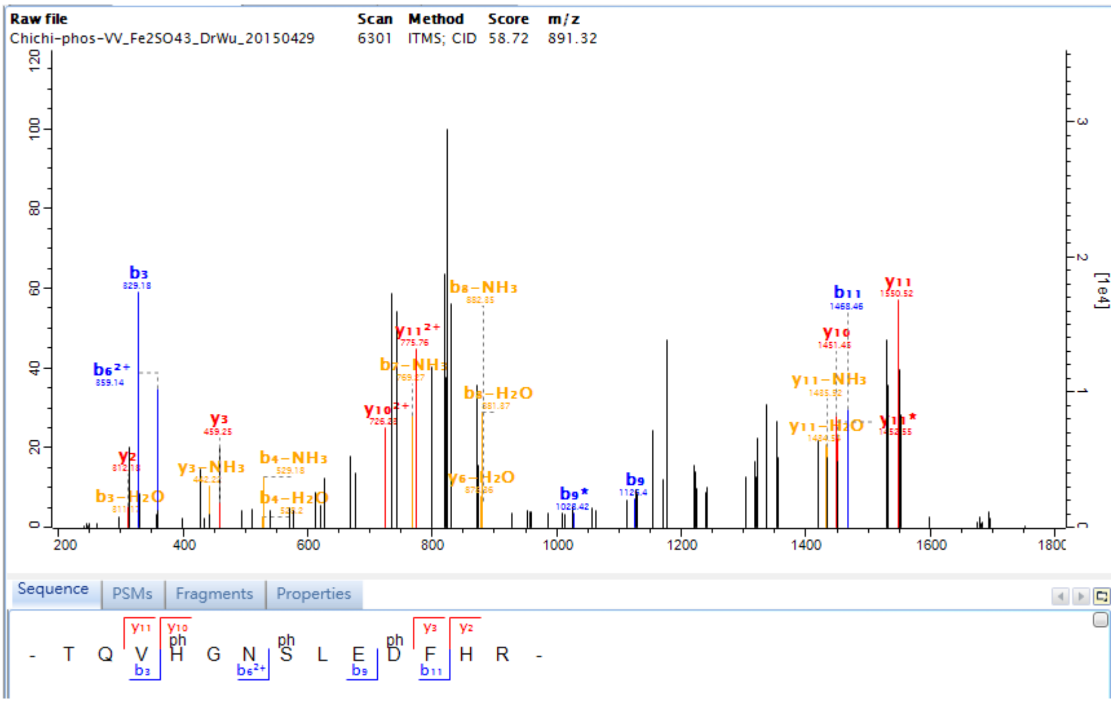
Raw file
Chichi-phos-VV_Fe2SO43_DrWu_20150429
Scan 6301
Method ITMS; CID
Score 58.72
m/z 891.32
Sequence
PSMs
Fragments
Properties
- T Q V H G N S L E D F H R -

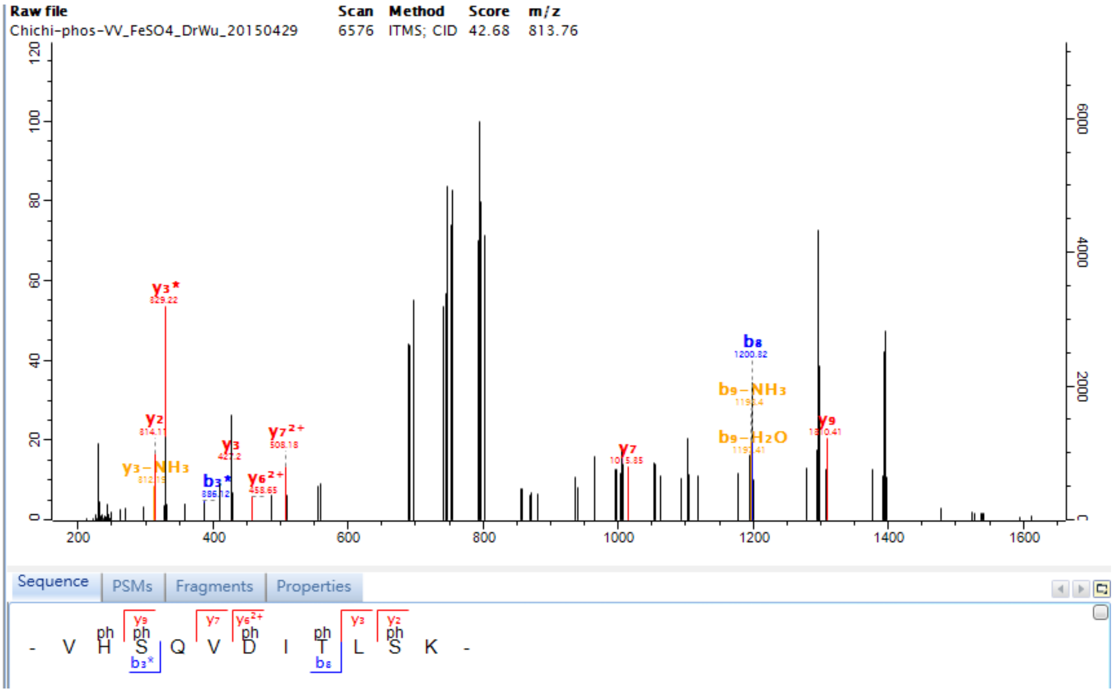
Raw file
Chichi-phos-VV_FeSO4_DrWu_20150429
Scan 6576
Method ITMS; CID
Score 42.68
m/z 813.76
Sequence
PSMs
Fragments
Properties
- V H S Q V D I T L S K -

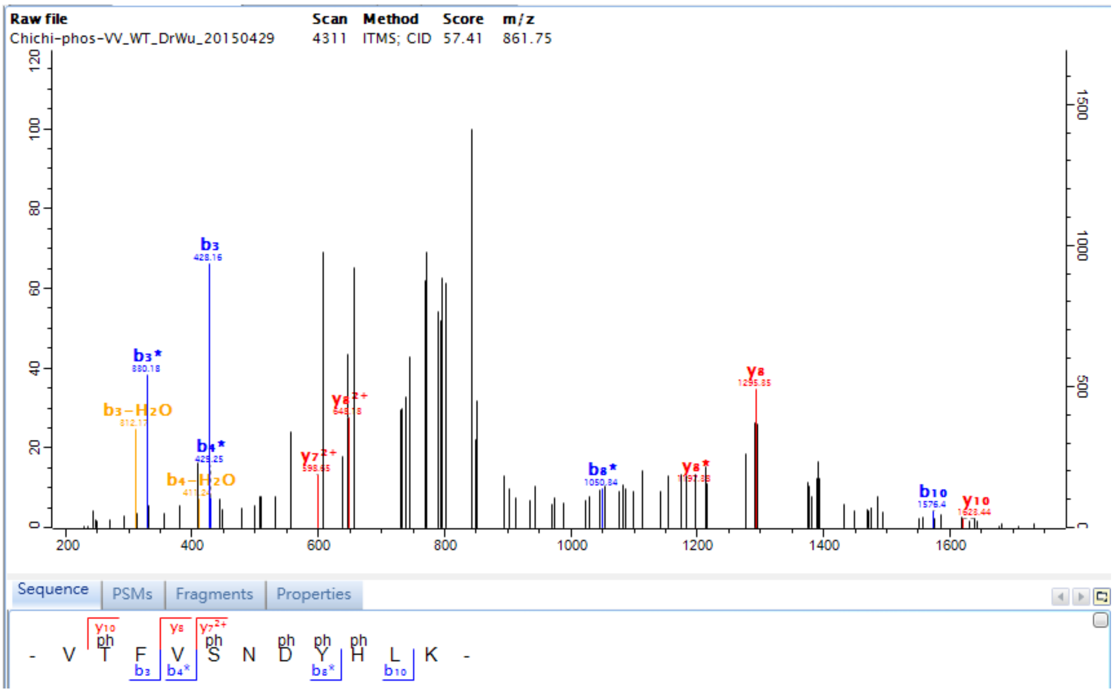

Raw file
Chichi-phos-VV_WT_DrWu_20150429
Scan
4311
Method
ITMS; CID
Score
57.41
m/z
861.75
Sequence
PSMs
Fragments
Properties
- V T F V S N D Y H L K -

Meiothermus taiwanensis WR220 (thermophilic bacterium)

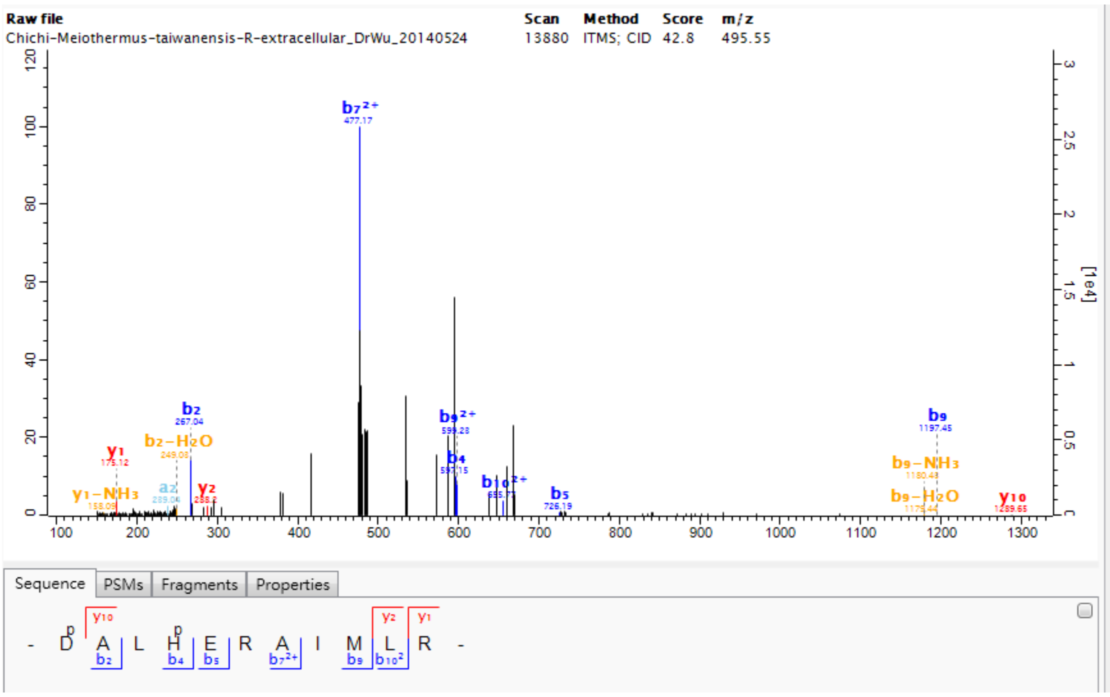

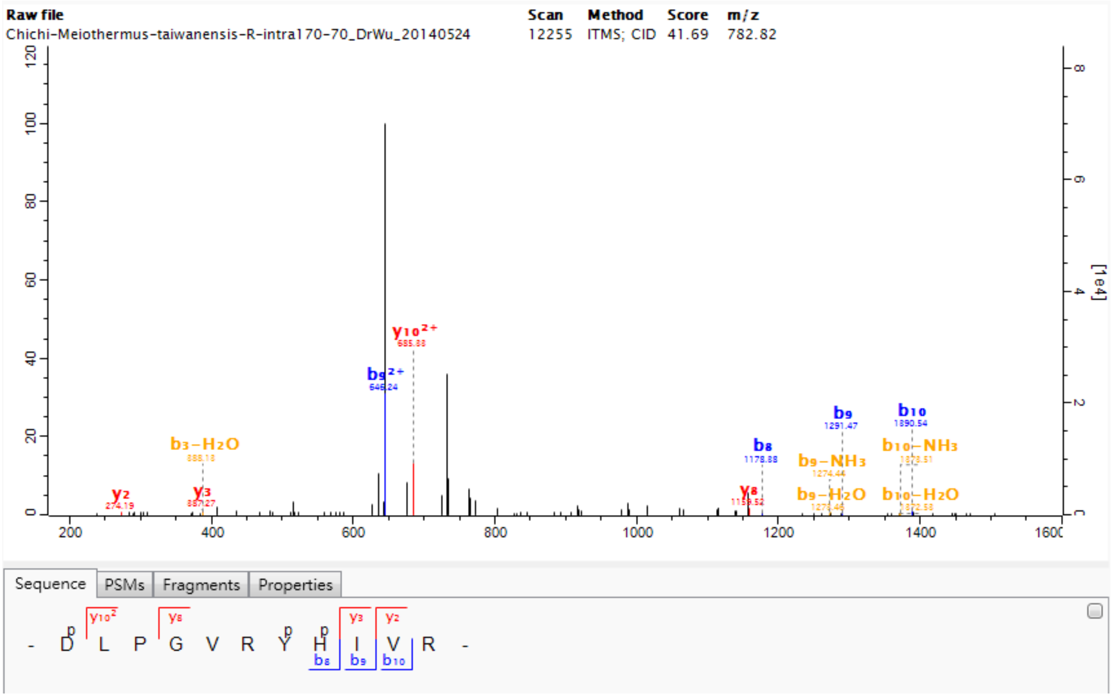

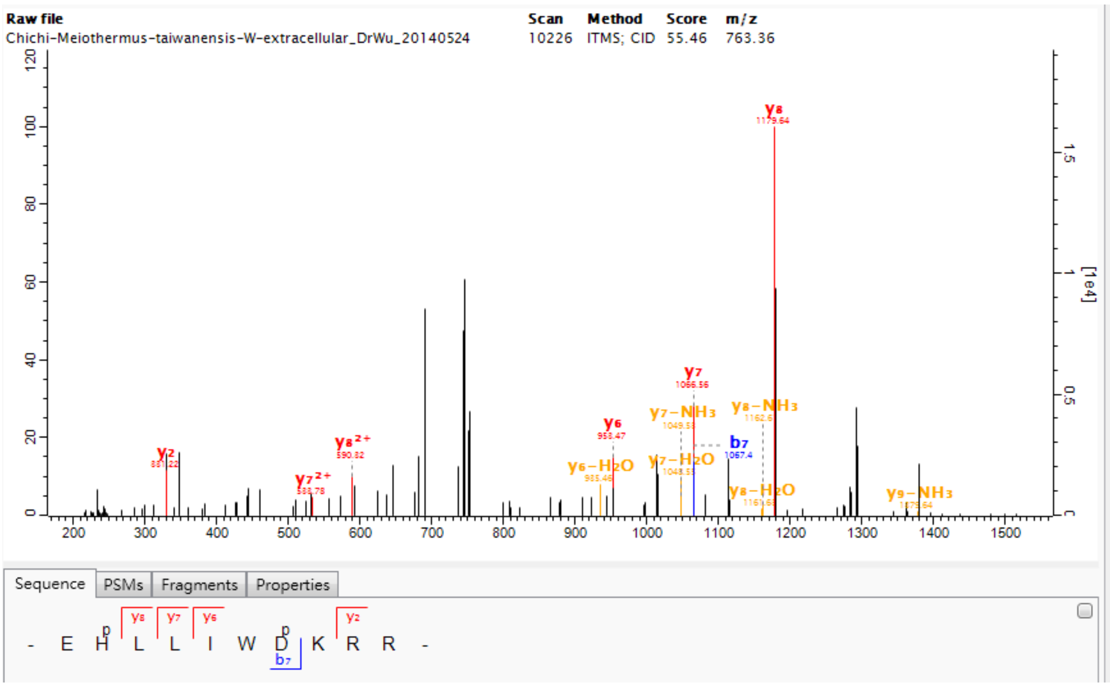
Raw file
Chichi-Meiothermus-taiwanensis-W-extracellular_DrWu_20140524
Scan Method Score m/z
10226 ITMS; CID 55.46 763.36
y8 1179.64
y7 1066.56
y6 953.47
y7-NH3
y8-NH3
b7 1067.4
y6-H2O
y7-H2O
y8-H2O
y9-NH3
y2
y8 2+ 590.82
y7 2+
[1e4]
Sequence PSMs Fragments Properties
- E H L L I W D K R R -

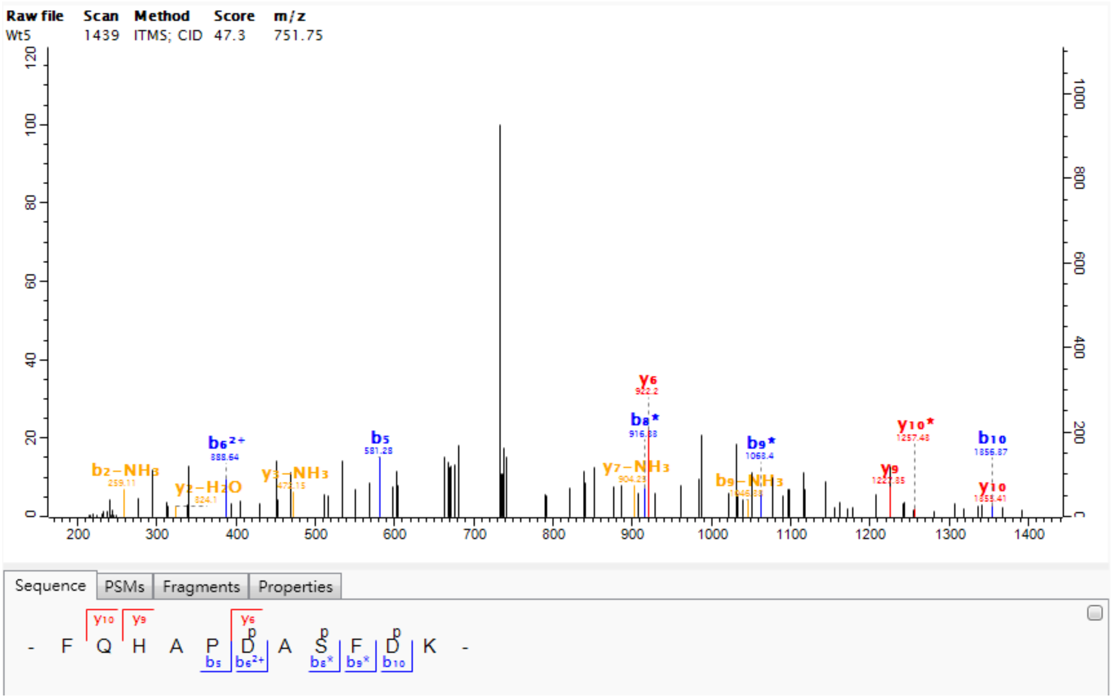
Raw file Scan Method Score m/z
Wt5 1439 ITMS; CID 47.3 751.75
y6 922.2
b8* 916.38
y7-NH3
b9* 1068.4
b9-NH3
y10* 1257.48
b10 1356.87
y9
y10
b6 2+ 388.64
b5 581.28
b2-NH3 259.11
y2-H2O
y3-NH3
Sequence PSMs Fragments Properties
- F Q H A P D A S F D K -

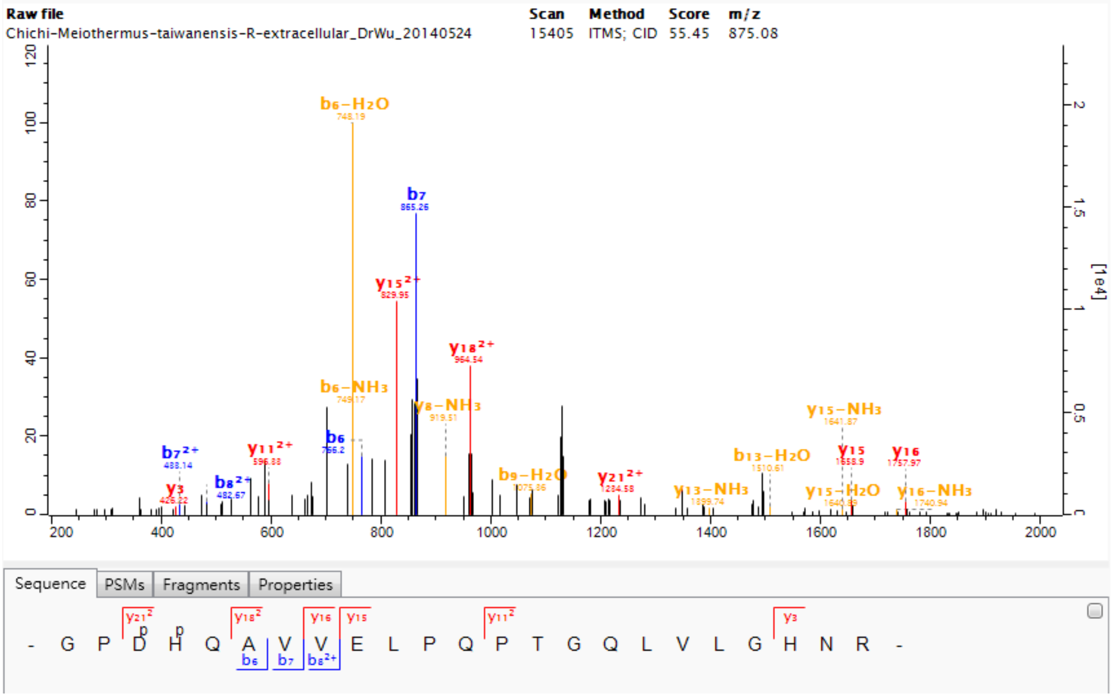
Raw file
Chichi-Meiothermus-taiwanensis-R-extracellular_DrWu_20140524
Scan Method Score m/z
15405 ITMS; CID 55.45 875.08
Sequence PSMs Fragments Properties
- G P D H Q A V V E L P Q P T G Q L V L G H N R -

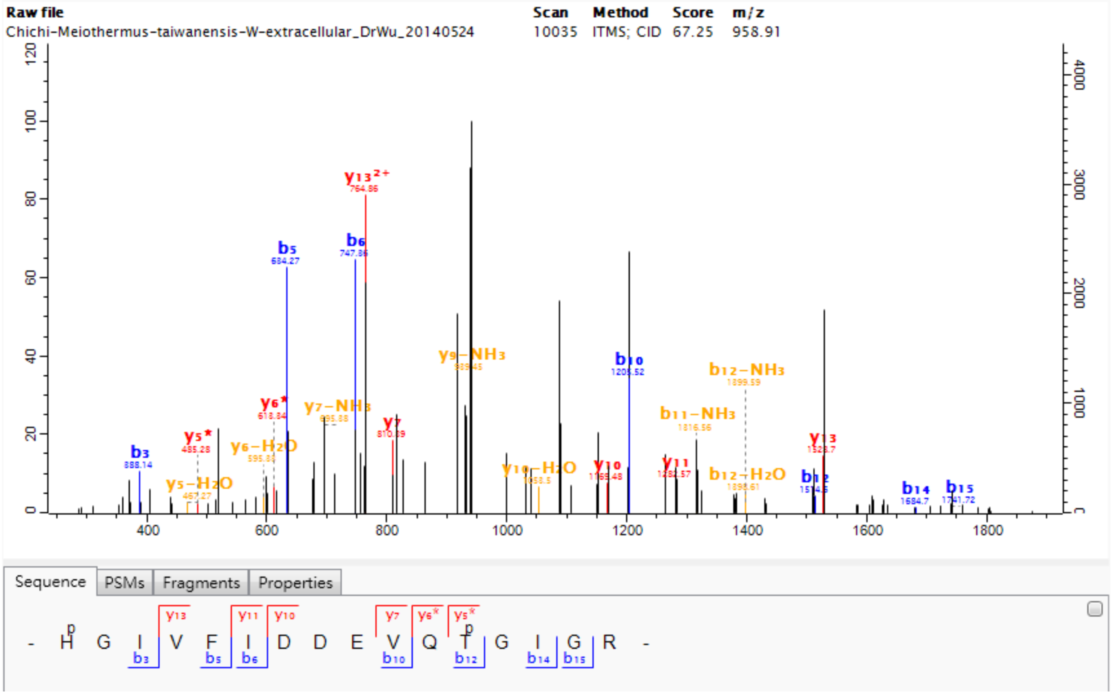
Raw file
Chichi-Meiothermus-taiwanensis-W-extracellular_DrWu_20140524
Scan Method Score m/z
10035 ITMS; CID 67.25 958.91
Sequence PSMs Fragments Properties
- H G I V F I D D E V Q T G I G R -

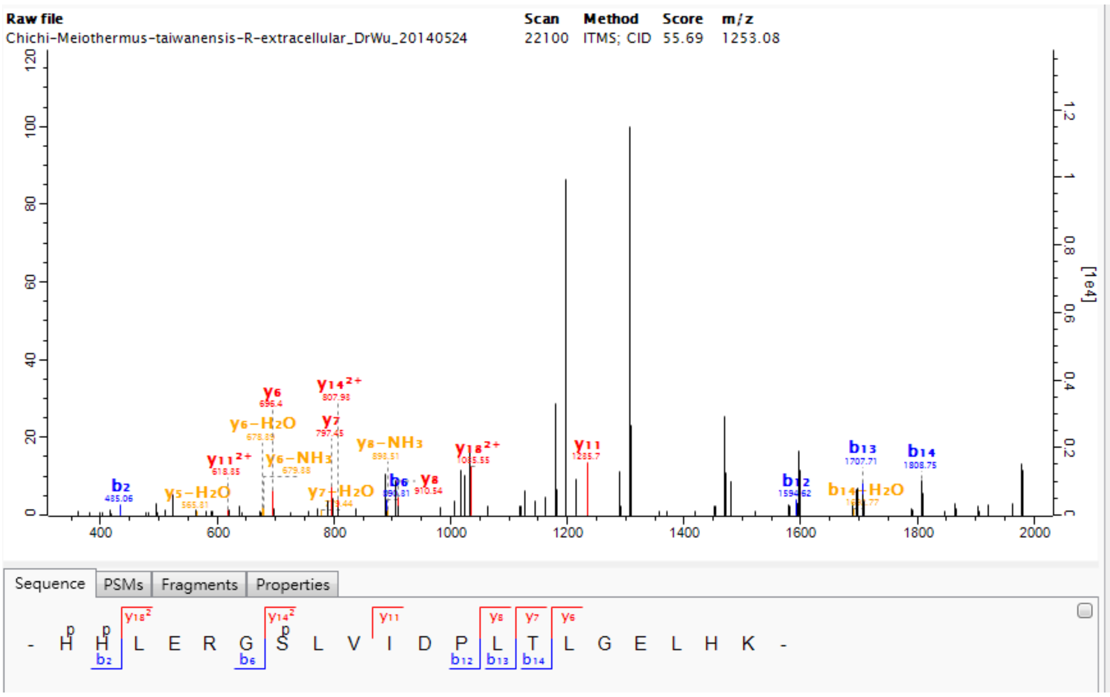
Raw file
Chichi-Meiothermus-taiwanensis-R-extracellular_DrWu_20140524
Scan 22100
Method ITMS; CID
Score 55.69
m/z 1253.08
Sequence
PSMs
Fragments
Properties
- H H L E R G S L V I D P L T L G E L H K -

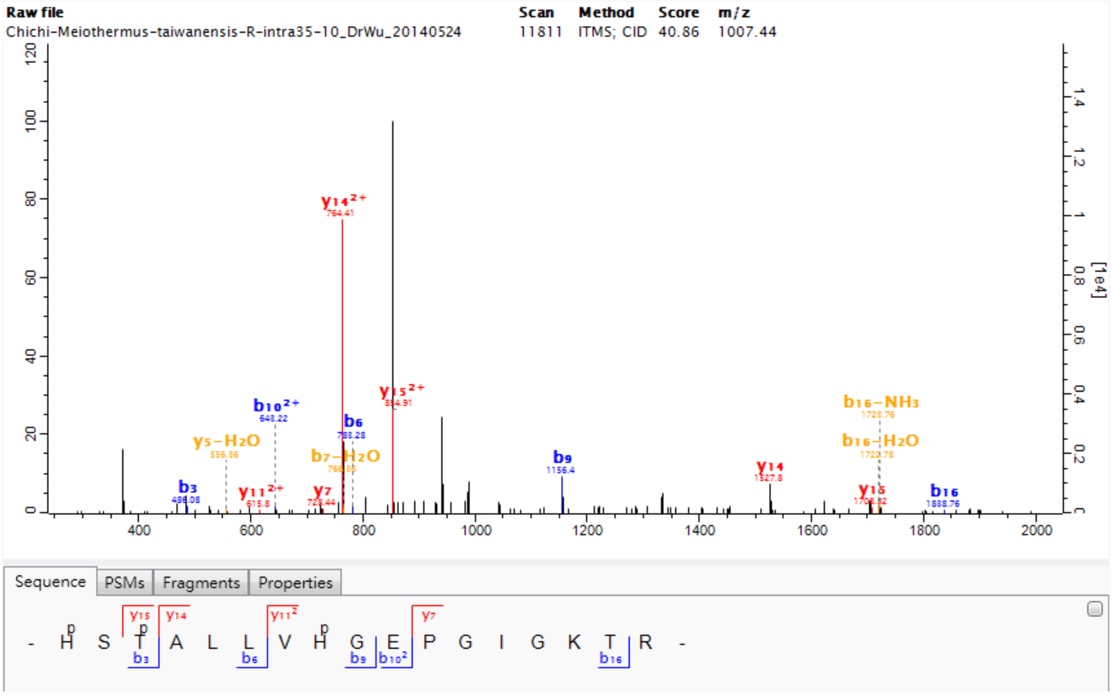
Raw file
Chichi-Meiothermus-taiwanensis-R-intra35-10_DrWu_20140524
Scan 11811
Method ITMS; CID
Score 40.86
m/z 1007.44
Sequence
PSMs
Fragments
Properties
- H S T A L L V H G E P G I G K T R -

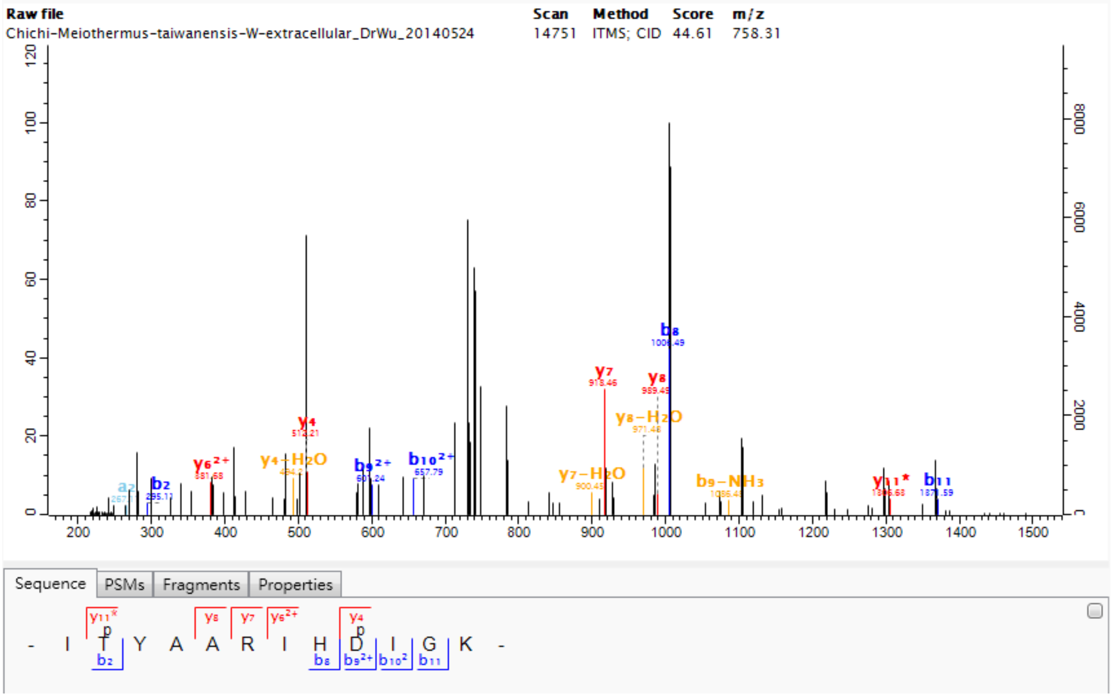
Raw file
Chichi-Meiothermus-taiwanensis-W-extracellular_DrWu_20140524
Scan
14751
Method
ITMS; CID
Score
44.61
m/z
758.31
Sequence
PSMs
Fragments
Properties
- I T Y A A R I H D I G K -

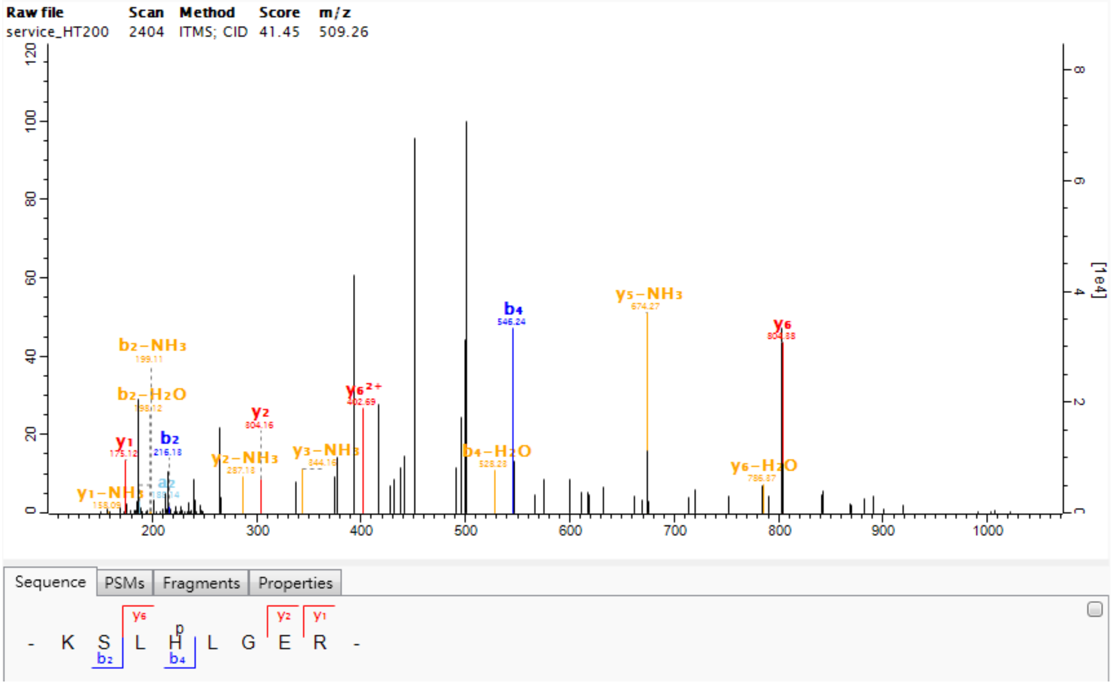
Raw file
service_HT200
Scan
2404
Method
ITMS; CID
Score
41.45
m/z
509.26
[1e4]
Sequence
PSMs
Fragments
Properties
- K S L H L G E R -

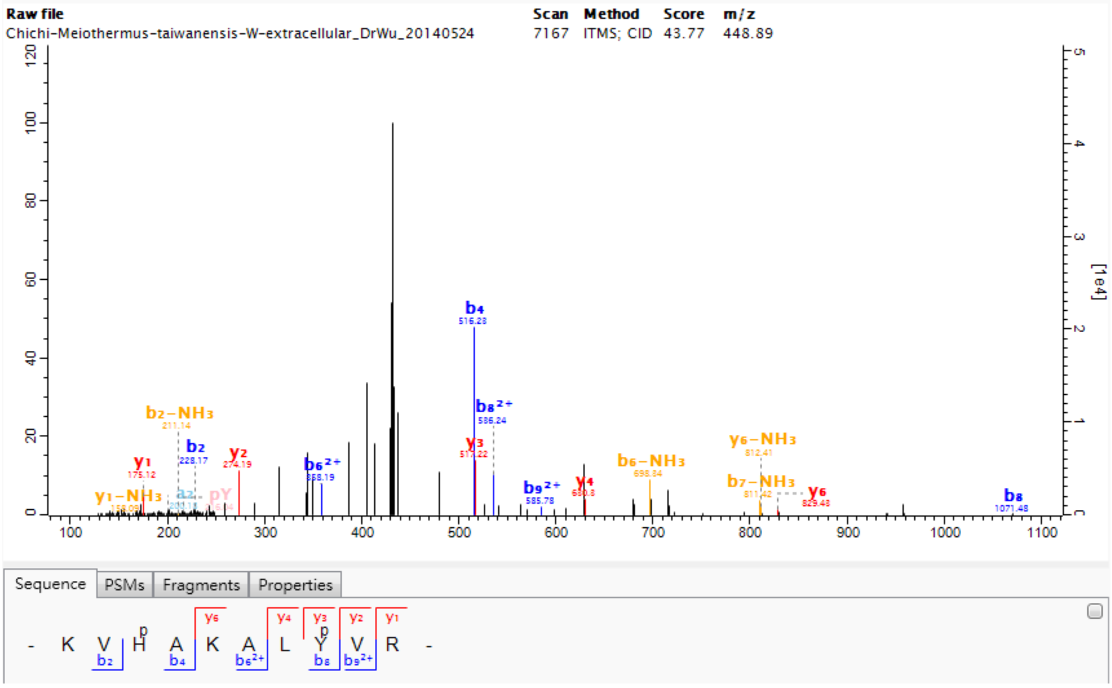

Raw file
Chichi-Meiothermus-taiwanensis-W-extracellular_DrWu_20140524
Scan Method Score m/z
7167 ITMS; CID 43.77 448.89
[1e4]
b4
b8 2+
b2-NH3
b2
y2
y1
y3
b6 2+
y6-NH3
b6-NH3
b7-NH3
y4
y6
b9 2+
b8
y1-NH3
Sequence
PSMs
Fragments
Properties
- K V H A K A L Y V R -

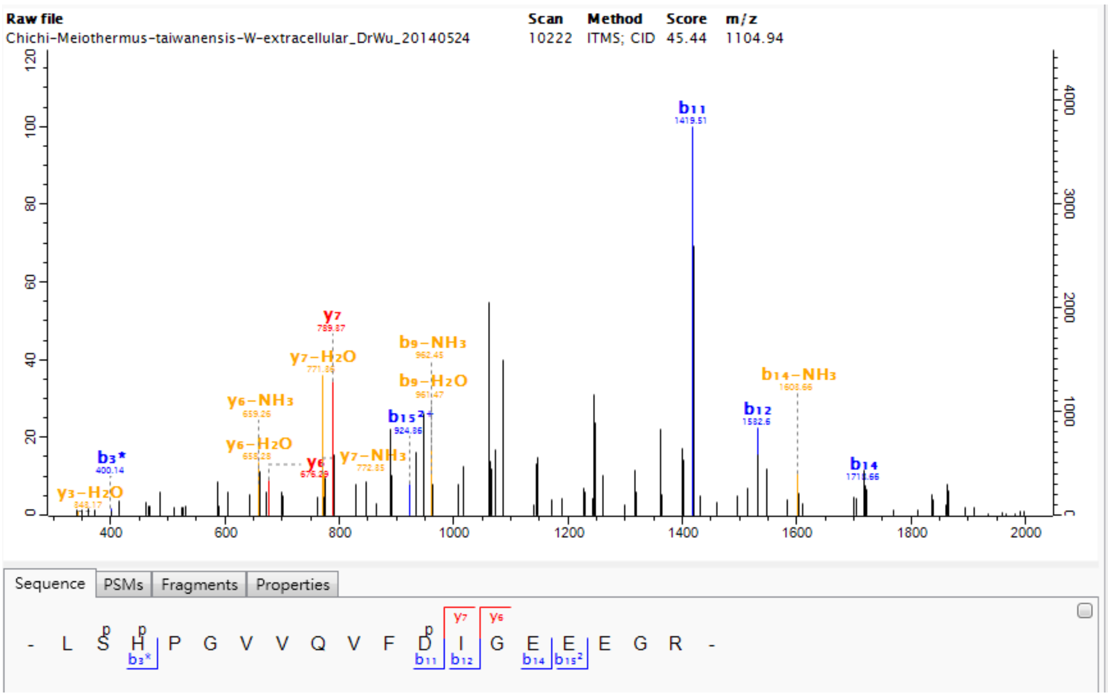

Raw file
Chichi-Meiothermus-taiwanensis-W-extracellular_DrWu_20140524
Scan Method Score m/z
10222 ITMS; CID 45.44 1104.94
b11
y7
y7-H2O
b9-NH3
b9-H2O
y6-NH3
y6-H2O
b15 2+
b14-NH3
b12
b3*
y3-H2O
y6
y7-NH3
b14
Sequence
PSMs
Fragments
Properties
- L S H P G V V Q V F D I G E E E G R -

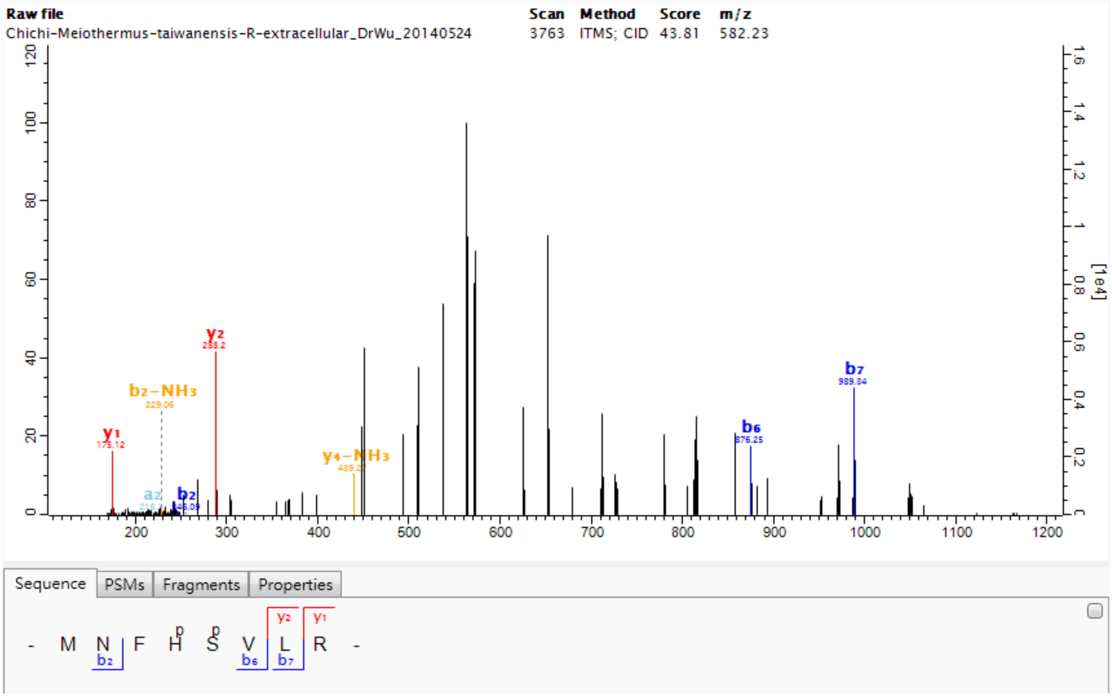
Raw file
Chichi-Meiothermus-taiwanensis-R-extracellular_DrWu_20140524
Scan Method Score m/z
3763 ITMS; CID 43.81 582.23
y1
y2
b2-NH3
a2
b2
y4-NH3
b6
b7
[1e4]
Sequence PSMs Fragments Properties
- M N F H S V L R -

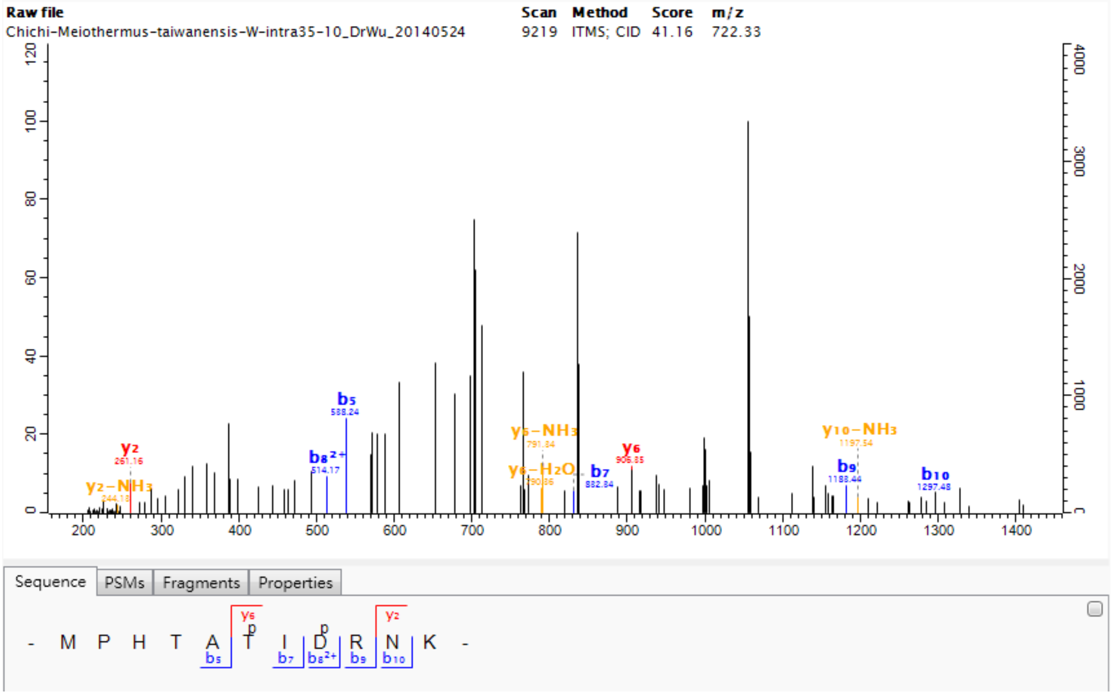
Raw file
Chichi-Meiothermus-taiwanensis-W-intra35-10_DrWu_20140524
Scan Method Score m/z
9219 ITMS; CID 41.16 722.33
y2
y2-NH3
b5
b8 2+
b7
y6
b9
b10
y10-NH3
Sequence PSMs Fragments Properties
- M P H T A T I D R N K -

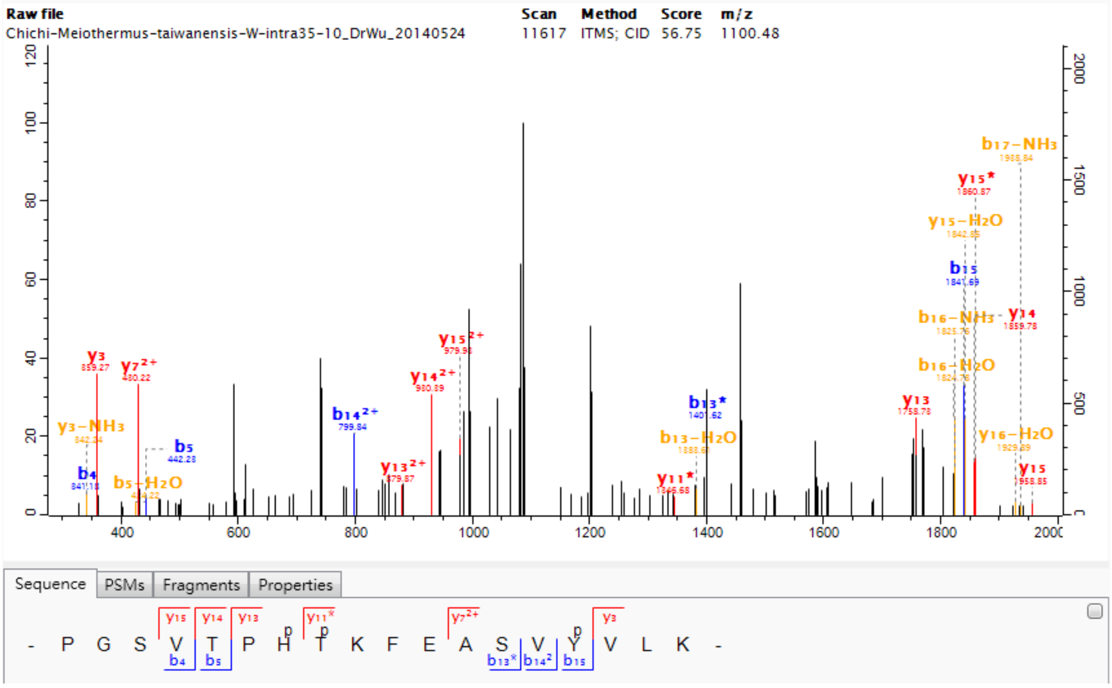
Raw file
Chichi-Meiothermus-taiwanensis-W-intra35-10_DrWu_20140524
Scan
11617
Method
ITMS; CID
Score
56.75
m/z
1100.48
Sequence
PSMs
Fragments
Properties
- P G S V T P H T K F E A S V Y V L K -

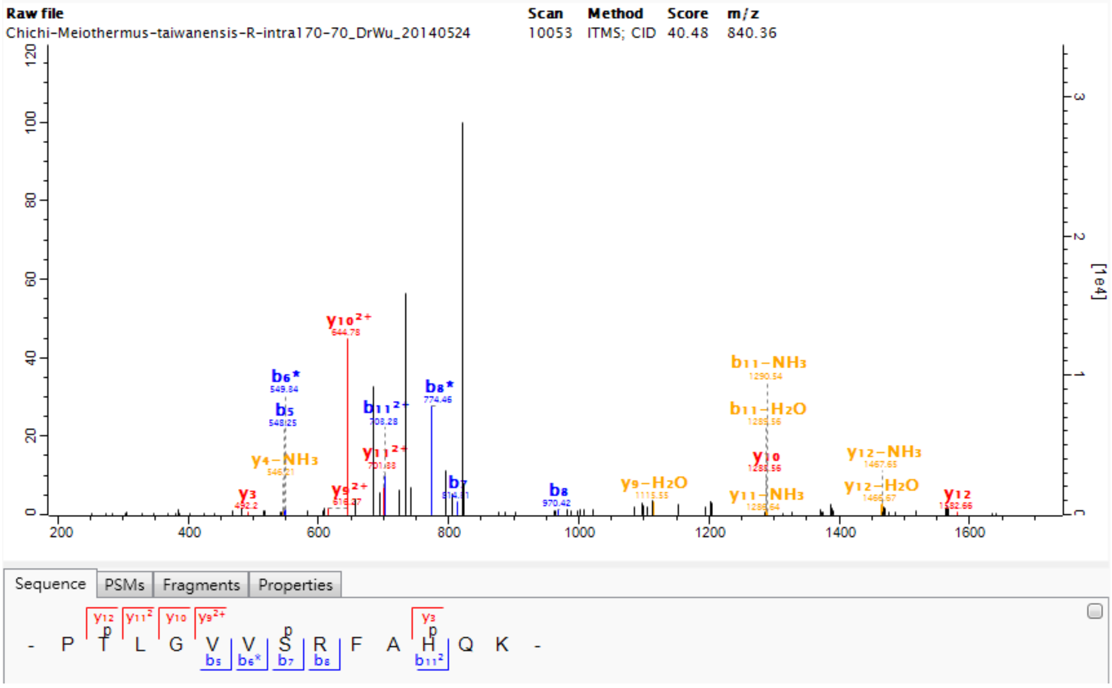
Raw file
Chichi-Meiothermus-taiwanensis-R-intra170-70_DrWu_20140524
Scan
10053
Method
ITMS; CID
Score
40.48
m/z
840.36
[1e4]
Sequence
PSMs
Fragments
Properties
- P T L G V V S R F A H Q K -

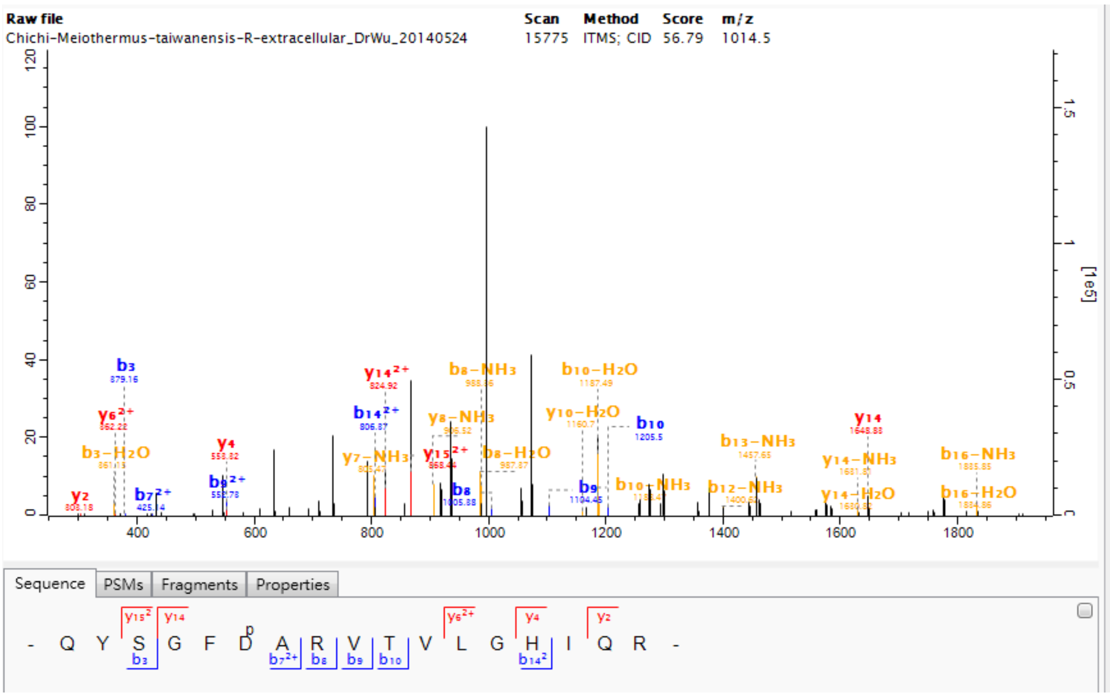
Raw file
Chichi-Meiothermus-taiwanensis-R-extracellular_DrWu_20140524
Scan
15775
Method
ITMS; CID
Score
56.79
m/z
1014.5
[1e5]
Sequence
PSMs
Fragments
Properties
- Q Y S G F D A R V T V L G H I Q R -

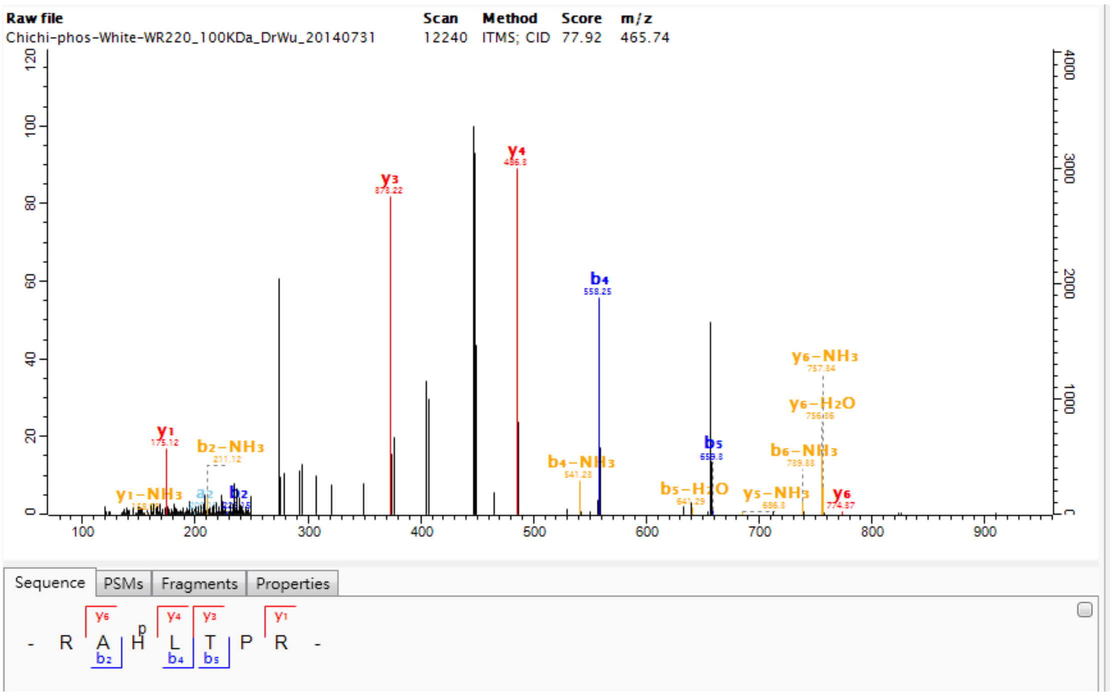
Raw file
Chichi-phos-White-WR220_100KDa_DrWu_20140731
Scan
12240
Method
ITMS; CID
Score
77.92
m/z
465.74
Sequence
PSMs
Fragments
Properties
- R A H L T P R -

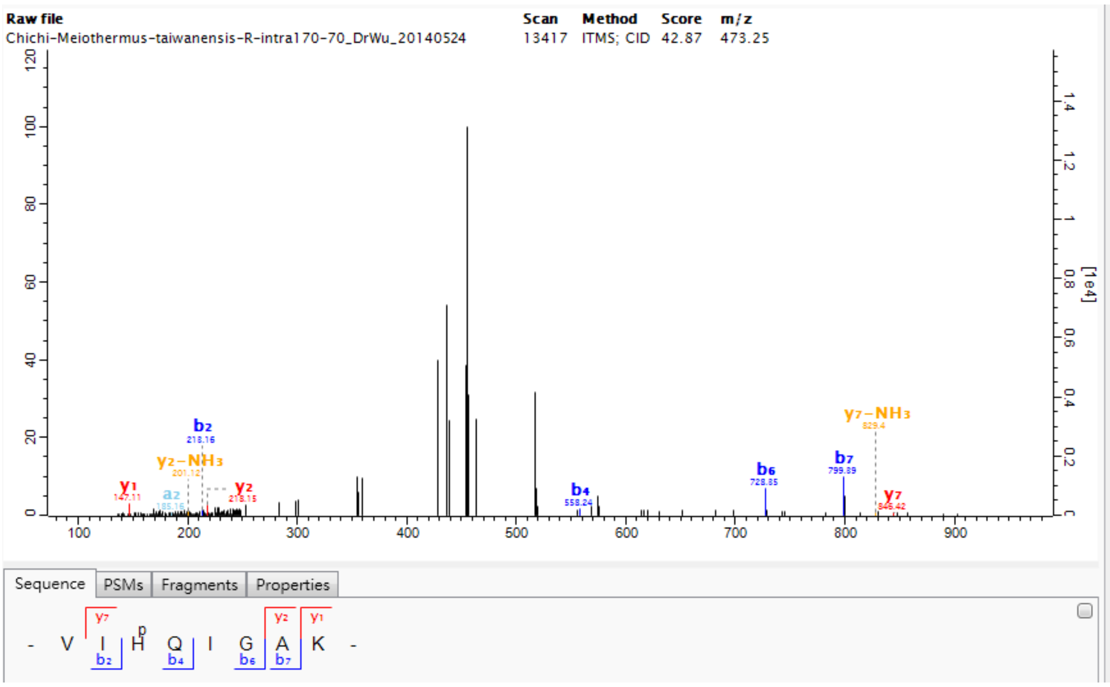
Raw file
Chichi-Meiothermus-taiwanensis-R-intra170-70_DrWu_20140524
Scan Method Score m/z
13417 ITMS; CID 42.87 473.25
b2
y2-NH3
y1
a2
y2
b4
b6
b7
y7-NH3
y7
[1e4]
Sequence PSMs Fragments Properties
- V I H Q I G A K -

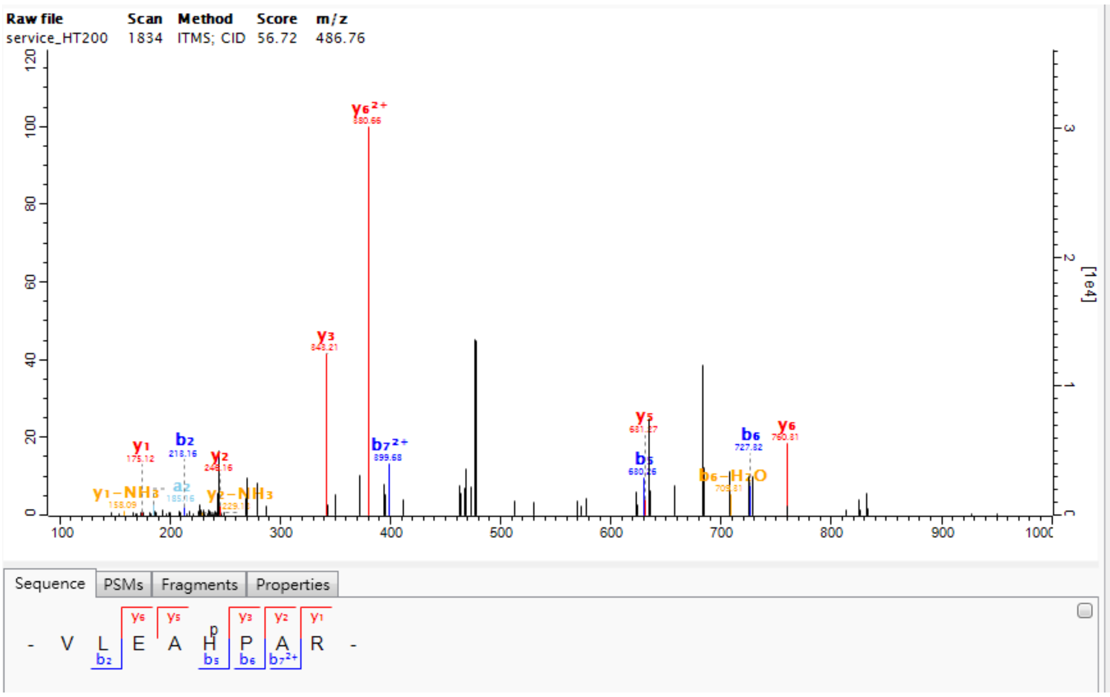
Raw file
service_HT200
Scan Method Score m/z
1834 ITMS; CID 56.72 486.76
y6 2+
y3
y1
b2
y2
a2
y1-NH3
y2-NH3
b7 2+
y5
b5
b6
y6
b6-H2O
[1e4]
Sequence PSMs Fragments Properties
- V L E A H P A R -

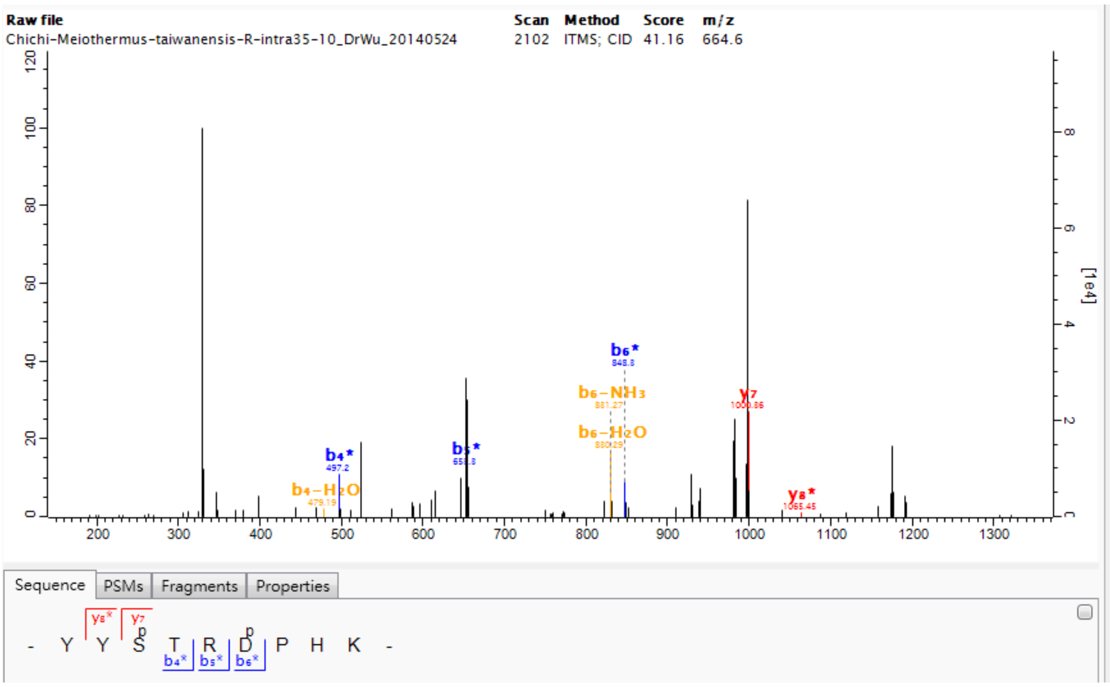

Raw file
Chichi-Meiothermus-taiwanensis-R-intra35-10_DrWu_20140524
Scan 2102
Method ITMS; CID
Score 41.16
m/z 664.6
b6*
b6-NH3
b6-H2O
b4*
b5*
b4-H2O
y7
y8*
[1e4]
Sequence
PSMs
Fragments
Properties
- Y Y S T R D P H K -

Thermus thermophiles HB27 (thermophilic bacterium)

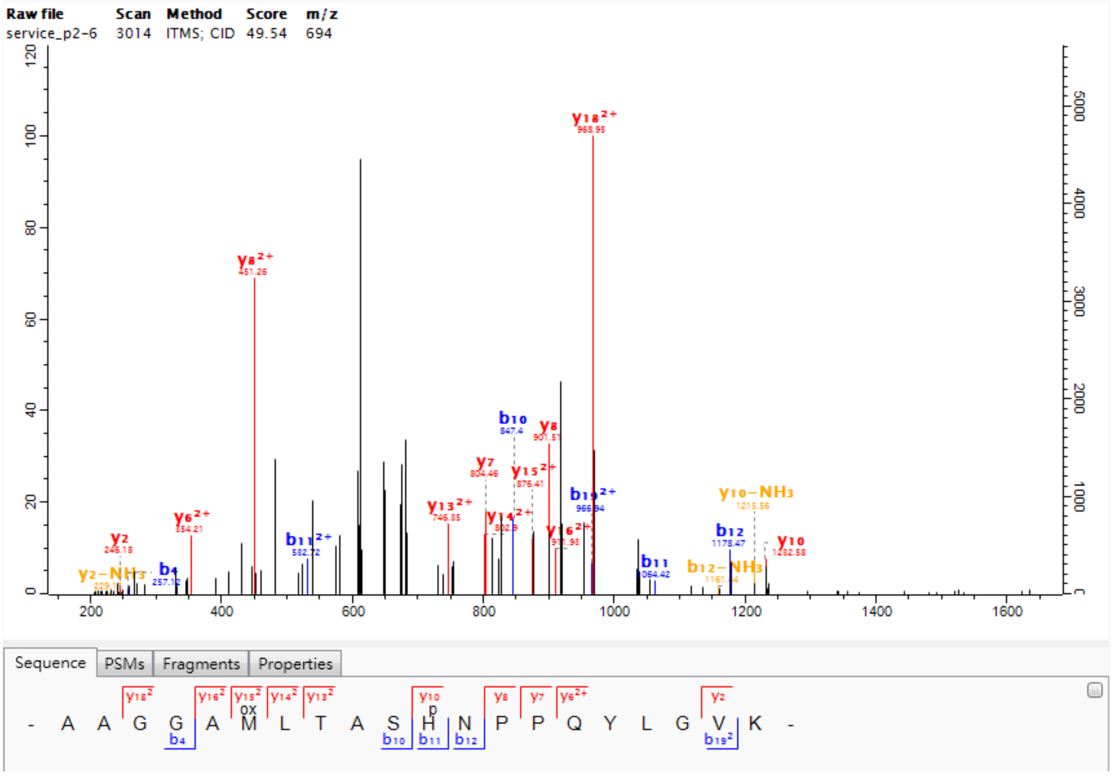

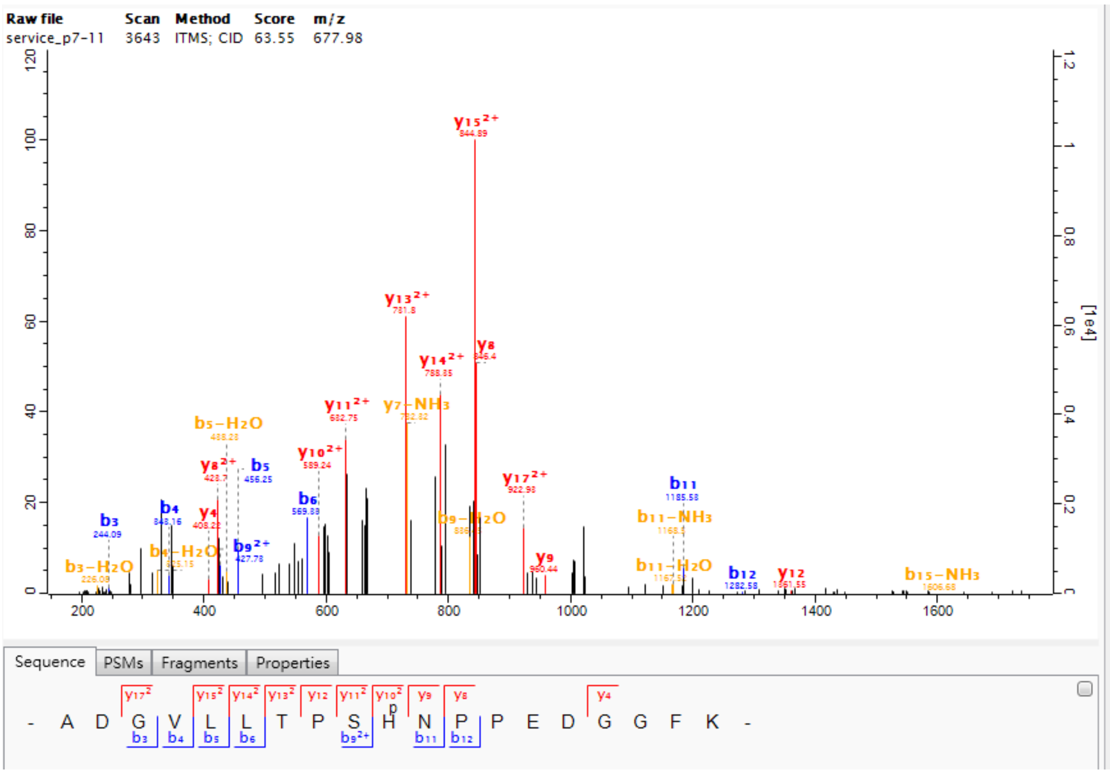

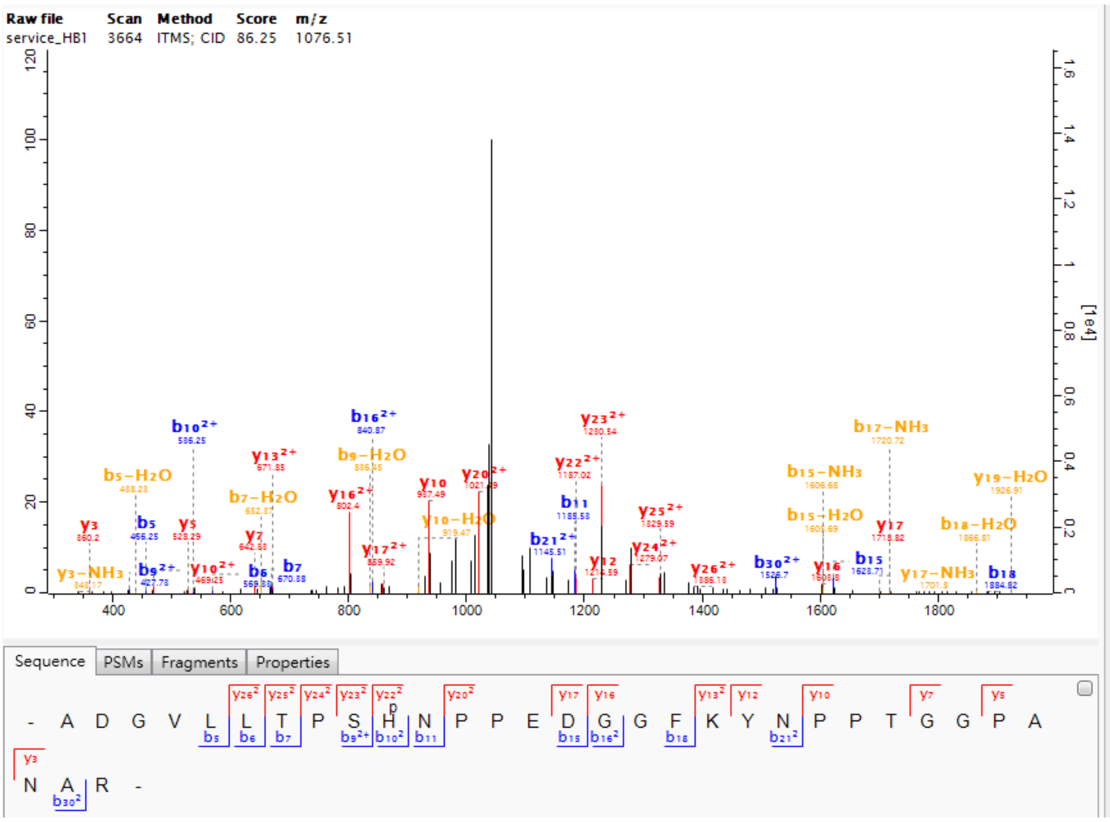
Raw file Scan Method Score m/z
service_HB1 3664 ITMS; CID 86.25 1076.51
Sequence PSMs Fragments Properties
- A D G V L L T P S H N P P E D G G F K Y N P P T G G P A
N A R -

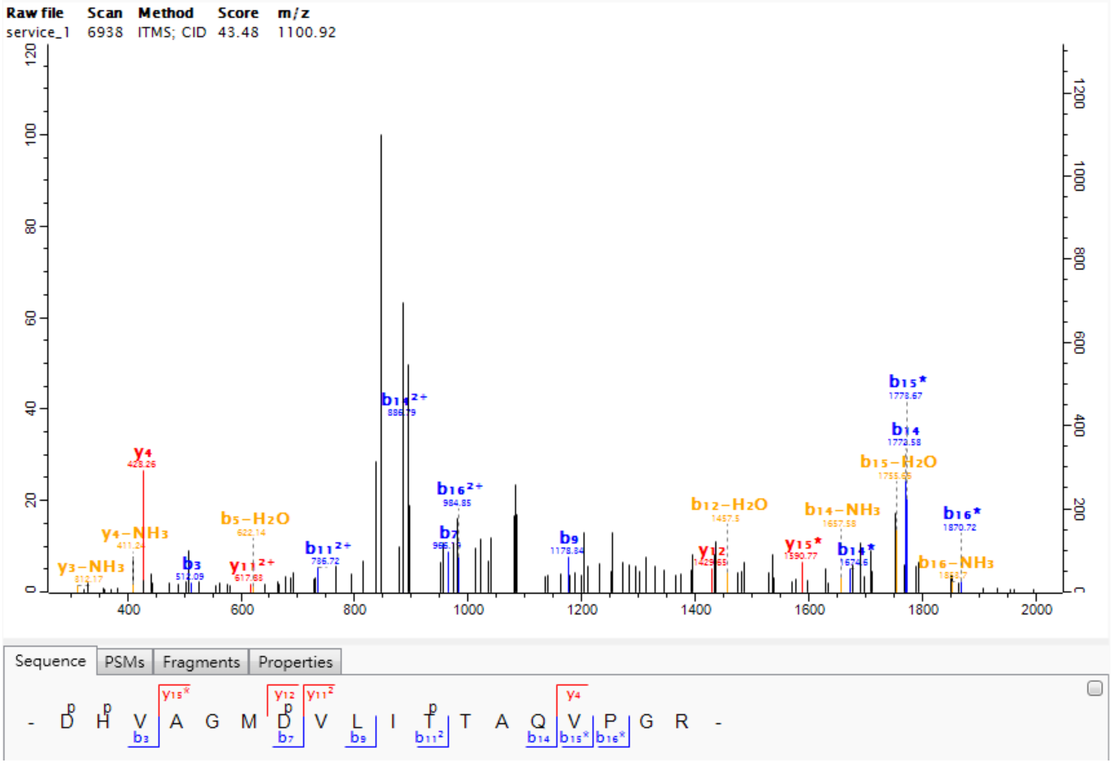
Raw file Scan Method Score m/z
service_1 6938 ITMS; CID 43.48 1100.92
Sequence PSMs Fragments Properties
- D H V A G M D V L I T T A Q V P G R -

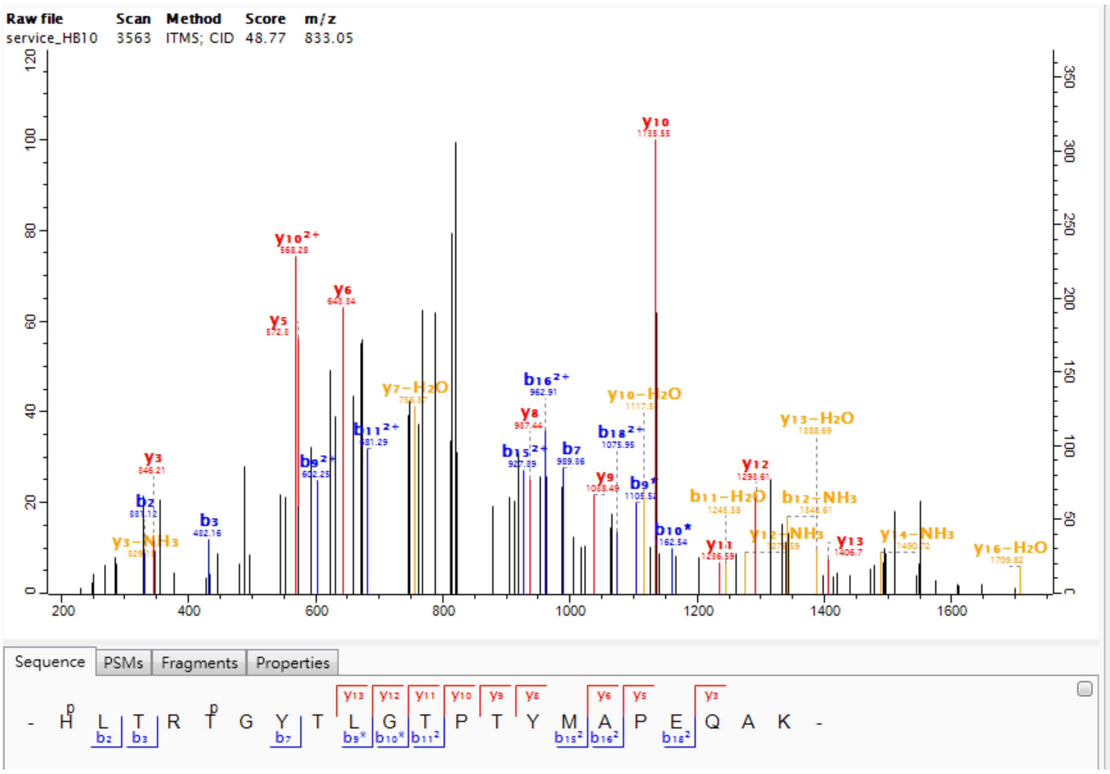
Raw file Scan Method Score m/z
service_HB10 3563 ITMS; CID 48.77 833.05
Sequence PSMs Fragments Properties

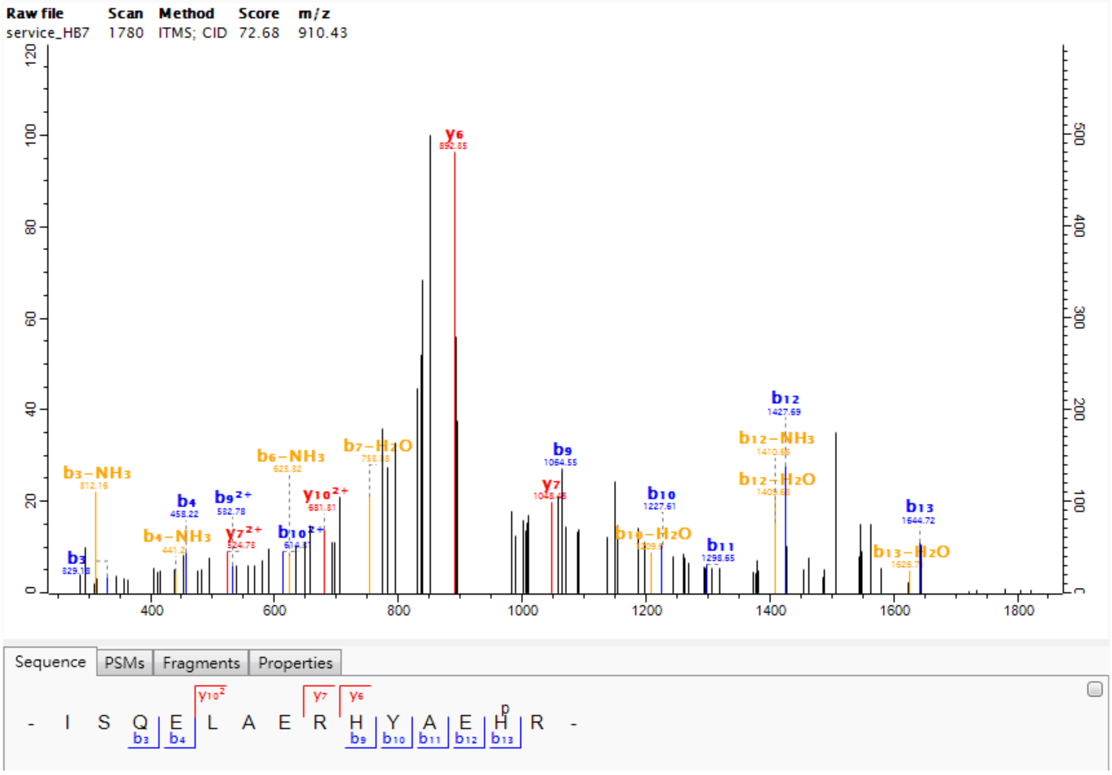
Raw file Scan Method Score m/z
service_HB7 1780 ITMS; CID 72.68 910.43
Sequence PSMs Fragments Properties

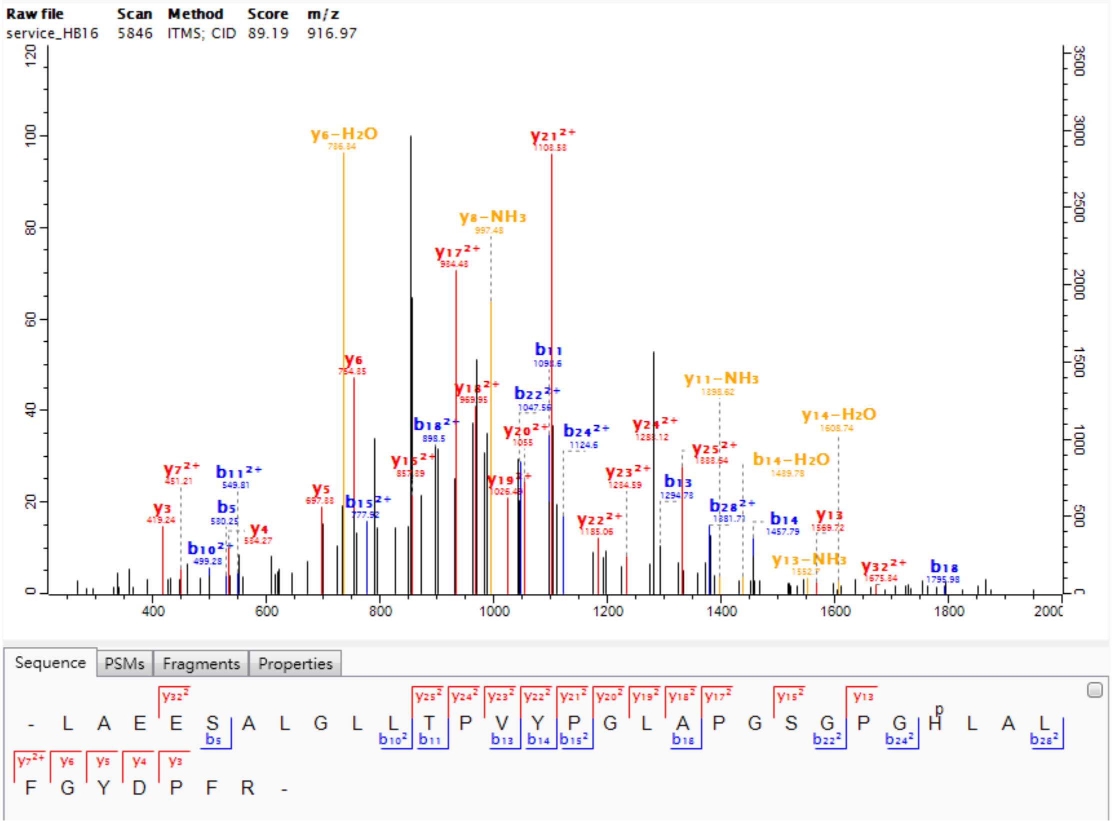
Raw file Scan Method Score m/z
service_HB16 5846 ITMS; CID 89.19 916.97
y6−H2O
y21 2+
y8−NH3
y17 2+
y6
b11
y18 2+
b22 2+
y11−NH3
y14−H2O
b18 2+
y20 2+
b24 2+
y24 2+
y25 2+
y7 2+
b11 2+
y15 2+
b14−H2O
y23 2+
y19 2+
b13
y5
b15 2+
y3
b5
b28 2+
y22 2+
b14
y13
y4
b10 2+
y13−NH3
y32 2+
b18
Sequence PSMs Fragments Properties
- L A E E S A L G L L T P V Y P G L A P G S G P G H L A L
F G Y D P F R -

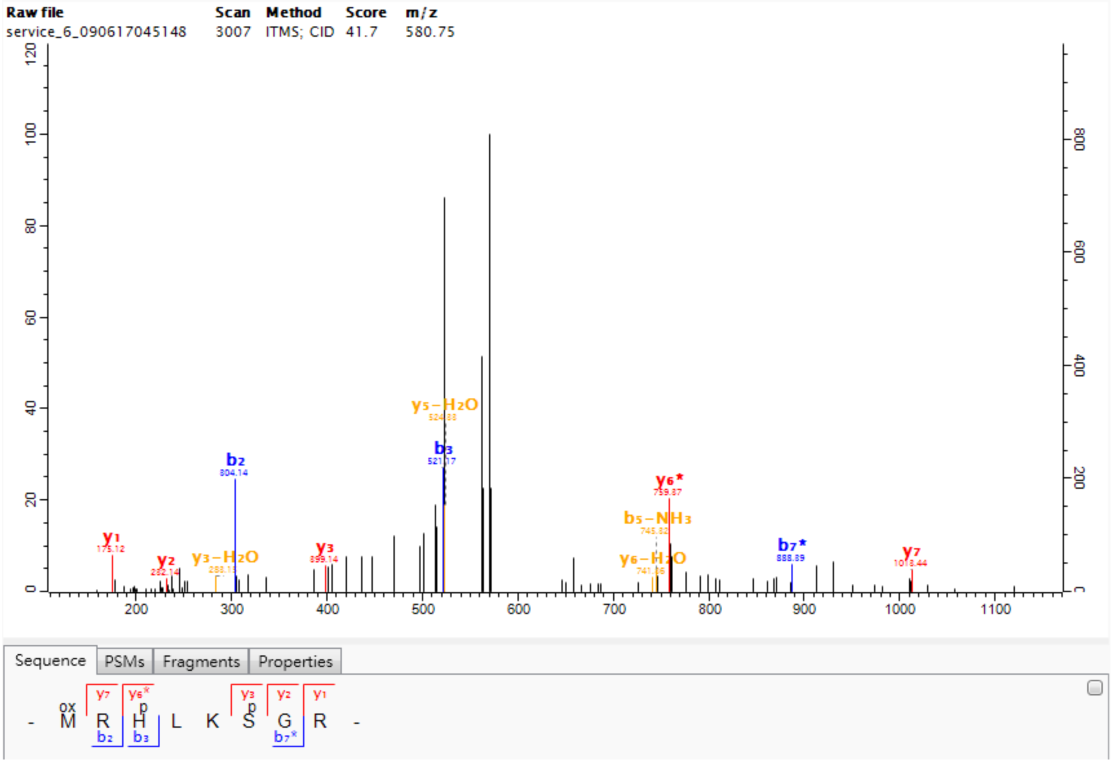
Raw file Scan Method Score m/z
service_6_090617045148 3007 ITMS; CID 41.7 580.75
y5−H2O
b3
b2
y6*
b5−NH3
y1
b7*
y2
y3−H2O
y3
y6−H2O
y7
Sequence PSMs Fragments Properties
- M R H L K S G R -

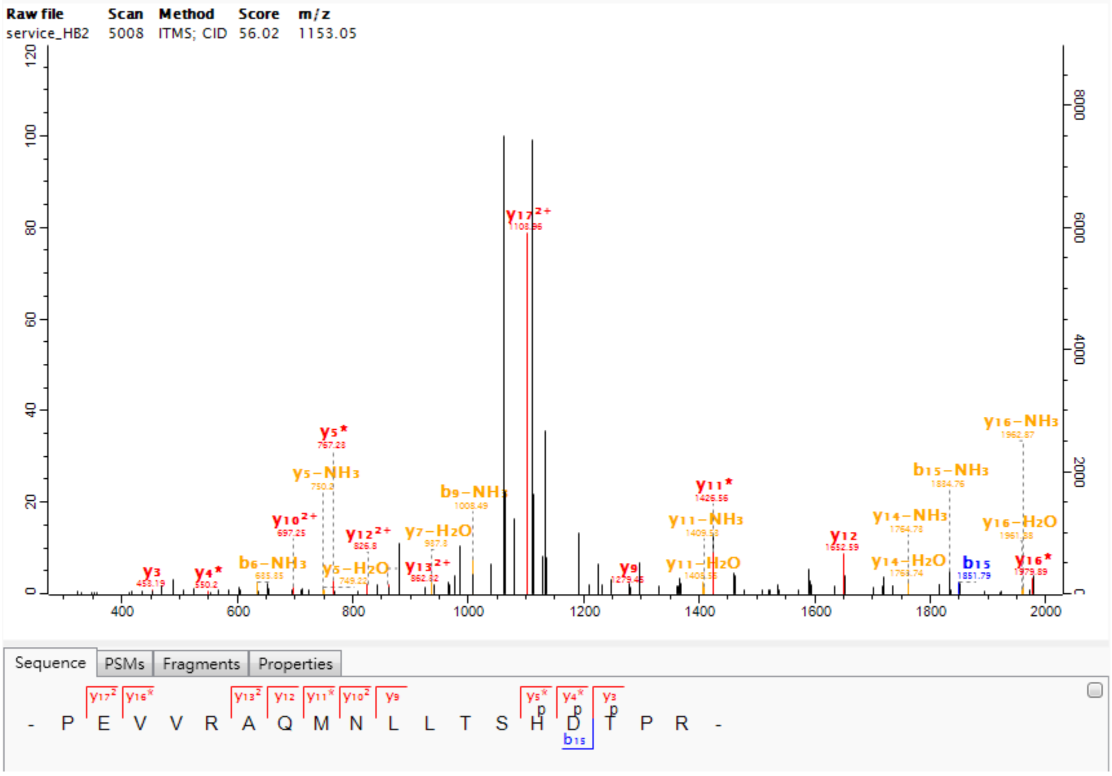
Raw file Scan Method Score m/z
service_HB2 5008 ITMS; CID 56.02 1153.05
y17 2+
y5*
y5−NH3
b9−NH3
y10 2+
y12 2+
y7−H2O
y11*
y11−NH3
y12
y14−NH3
b15−NH3
y16−NH3
y16−H2O
y3
y4*
b6−NH3
y5−H2O
y13 2+
y9
y11−H2O
y14−H2O
b15
y16*
Sequence
PSMs
Fragments
Properties
- P E V V R A Q M N L L T S H D T P R -

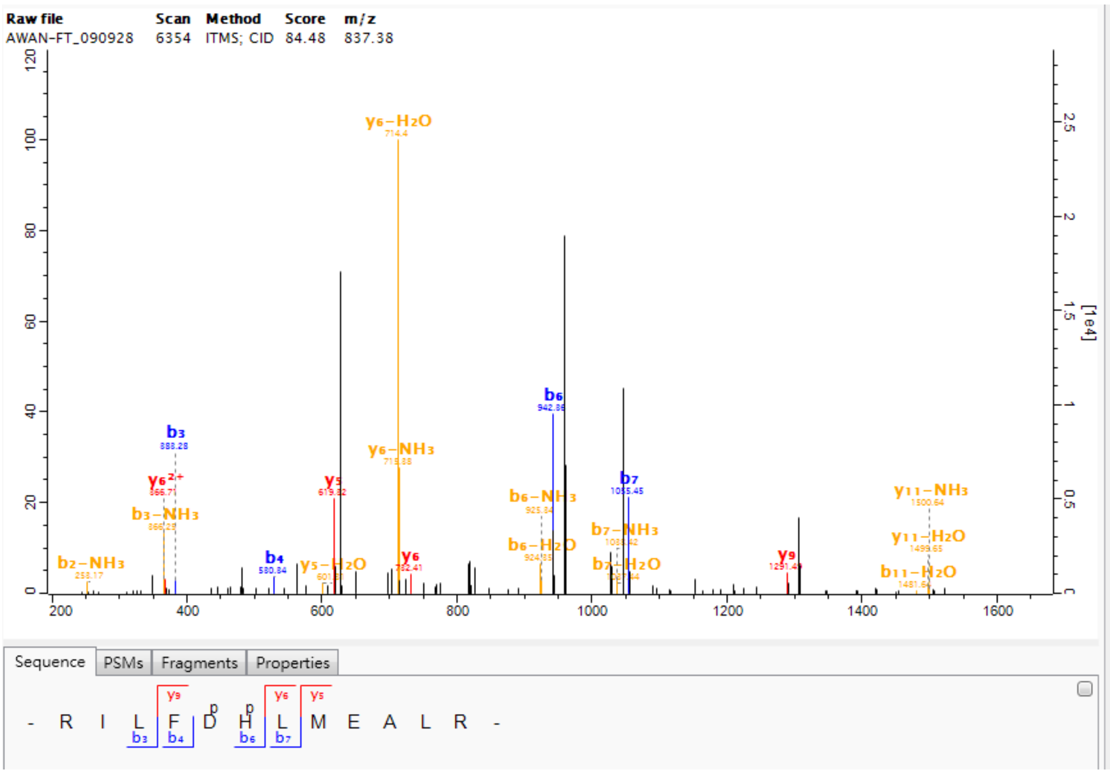
Raw file Scan Method Score m/z
AWAN-FT_090928 6354 ITMS; CID 84.48 837.38
y6−H2O
b3
y6 2+
b3−NH3
y5
y6−NH3
b6
b7
b6−NH3
b6−H2O
b7−NH3
b7−H2O
b2−NH3
b4
y5−H2O
y6
y9
y11−NH3
y11−H2O
b11−H2O
Sequence
PSMs
Fragments
Properties
- R I L F D H L M E A L R -

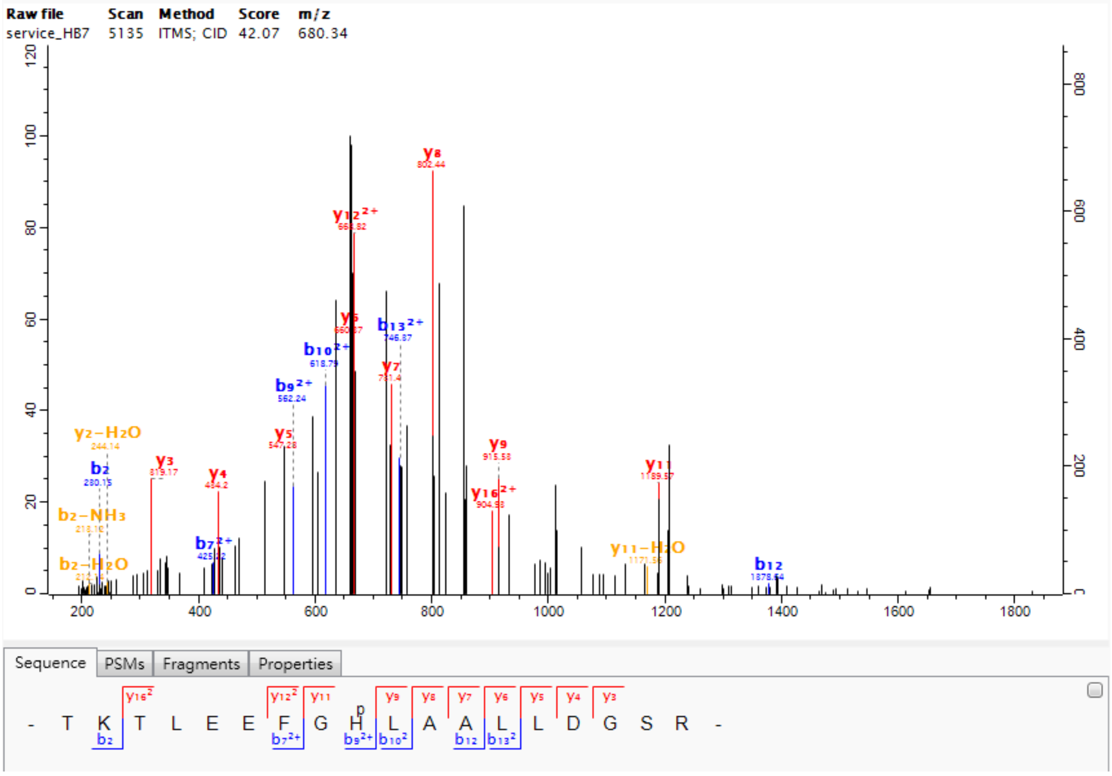

Raw file Scan Method Score m/z
service_HB7 5135 ITMS; CID 42.07 680.34
y8
802.44
b13 2+
746.87
b10 2+
618.79
b9 2+
562.24
y5
547.28
y9
915.58
y11
1189.57
y11-H2O
b12
y4
y3
319.17
b2
230.15
y2-H2O
244.14
b2-NH3
b2-H2O
b7 2+
y16 2+
y12 2+
y6
y7
200 400 600 800 1000 1200 1400 1600 1800
Sequence PSMs Fragments Properties
- T K T L E E F G H L A L L D G S R -

Methanosarcina mazei N2M9705 (methanogenic archaeon)

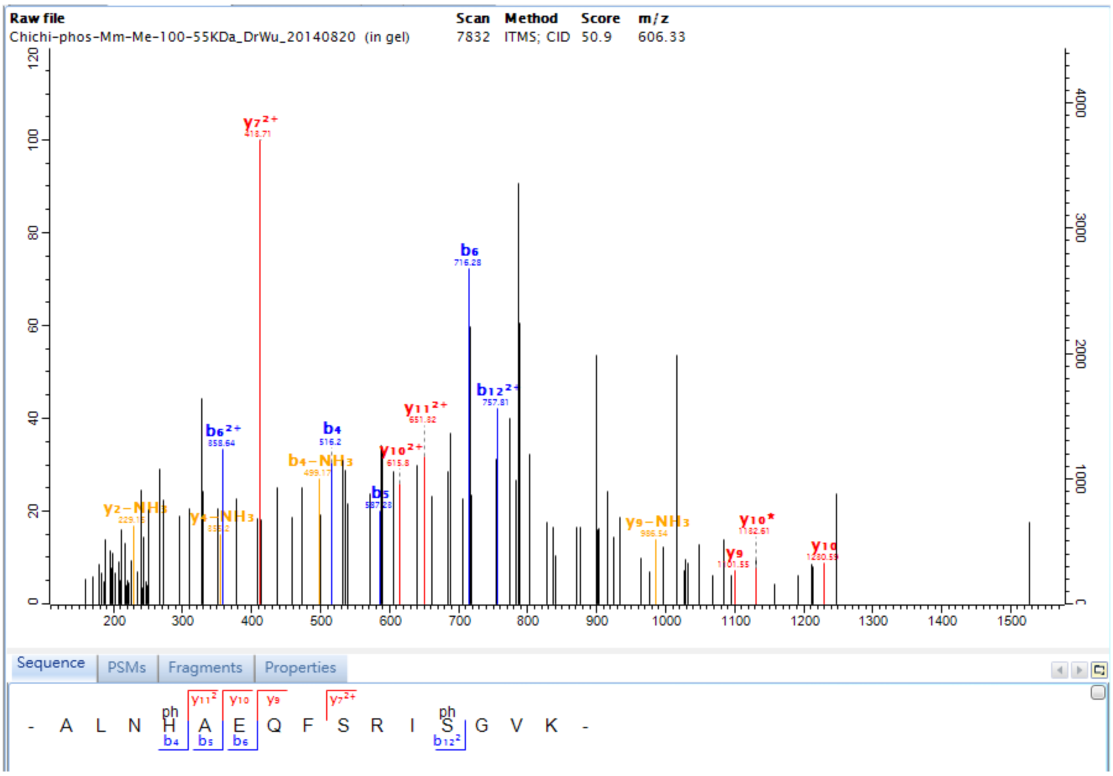

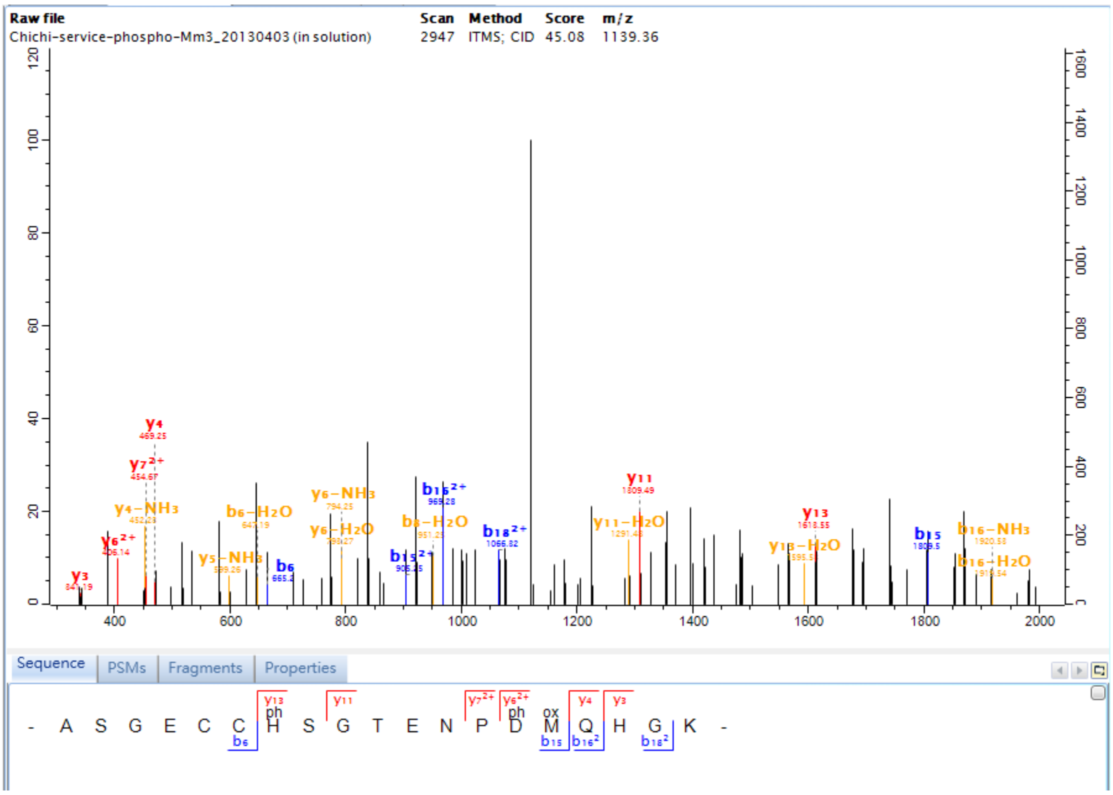

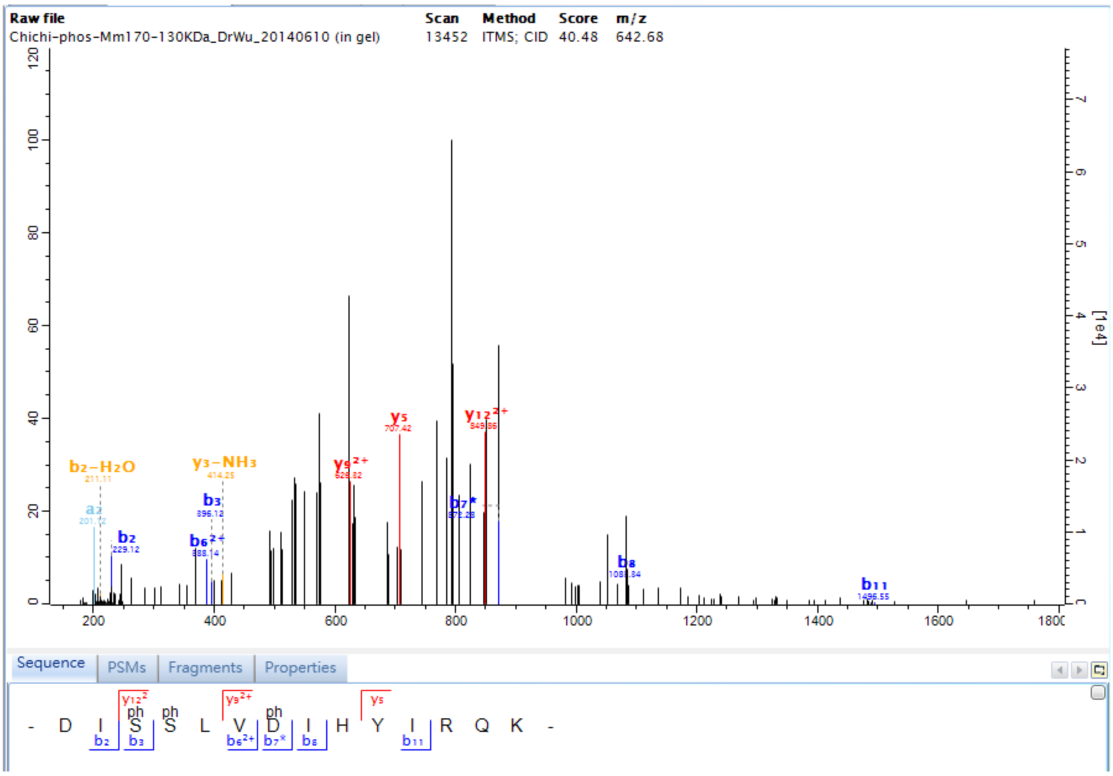

Raw file
Chichi-phos-Mm170-130KDa_DrWu_20140610 (in gel)
Scan
13452
Method
ITMS; CID
Score
40.48
m/z
642.68
[1e4]
b2-H2O
y3-NH3
a2
b2
b3
b6 2+
y9 2+
y5
y12 2+
b7*
b8
b11
Sequence
PSMs
Fragments
Properties
- D I S S L V D I H Y I R Q K -
ph
ph
ph
y12 2
y9 2+
y5
b2
b3
b6 2+
b7*
b8
b11

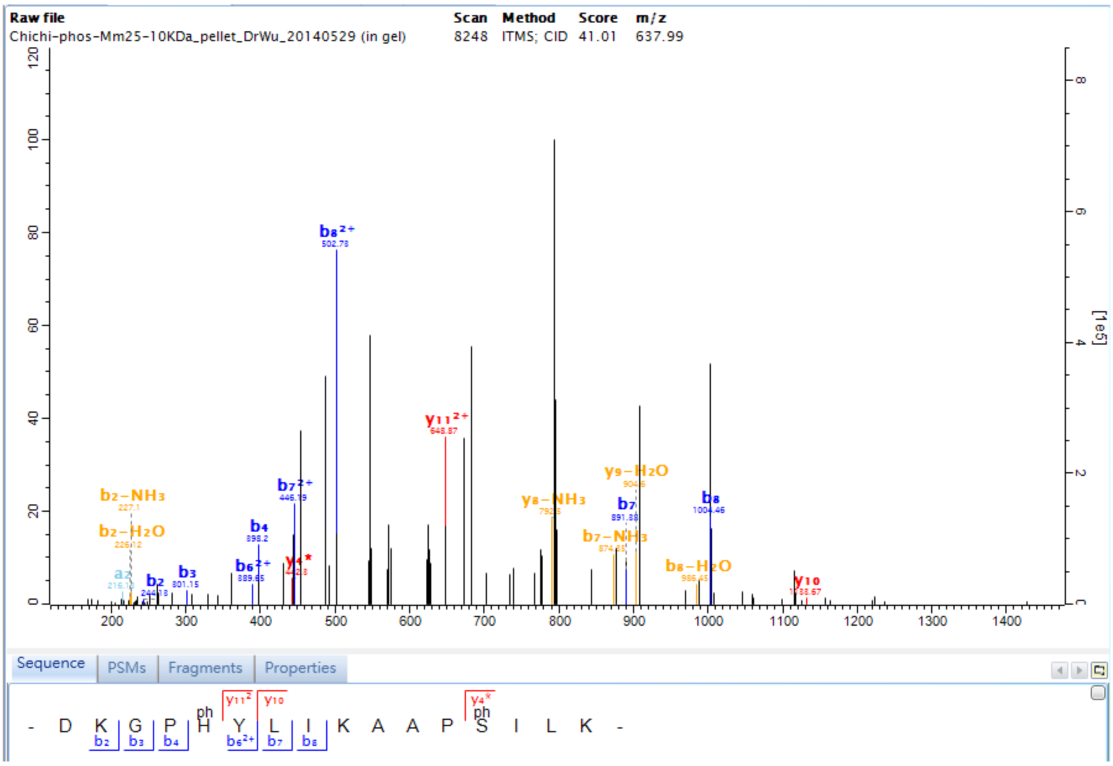

Raw file
Chichi-phos-Mm25-10KDa_pellet_DrWu_20140529 (in gel)
Scan
8248
Method
ITMS; CID
Score
41.01
m/z
637.99
[1e5]
b8 2+
y11 2+
b2-NH3
b2-H2O
a2
b2
b3
b4
b6 2+
b7 2+
y8-NH3
y9-H2O
b7
b7-NH3
b8
b8-H2O
y10
Sequence
PSMs
Fragments
Properties
- D K G P H Y L I K A A P S I L K -
ph
ph
y11 2
y10
y4*
b2
b3
b4
b6 2+
b7
b8

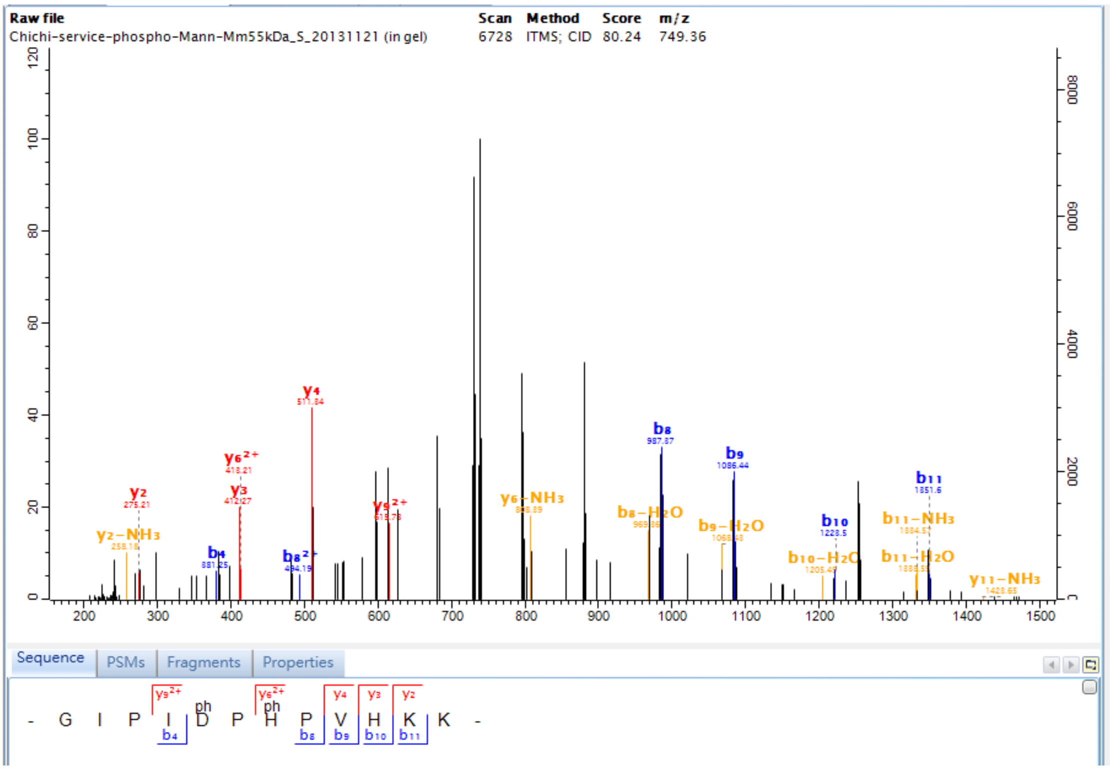
Raw file
Chichi-service-phospho-Mann-Mm55kDa_S_20131121 (in gel)
Scan Method Score m/z
6728 ITMS; CID 80.24 749.36
Sequence
PSMs
Fragments
Properties
- G I P I D P H P V H K K -

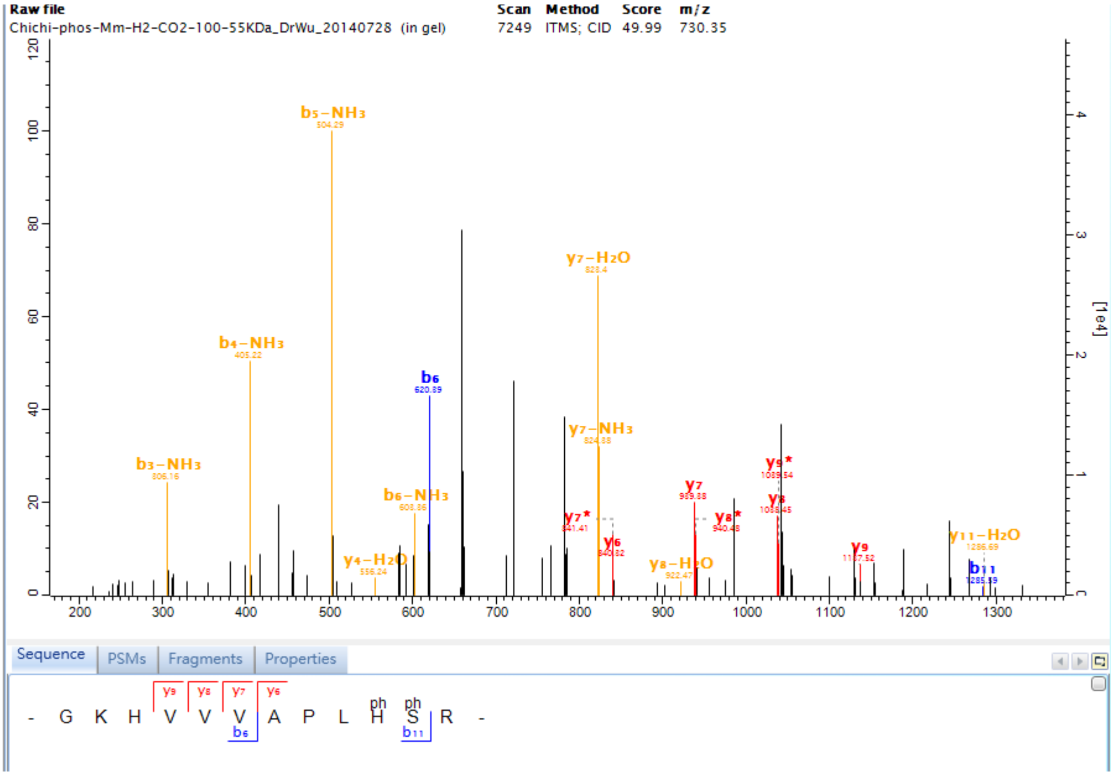
Raw file
Chichi-phos-Mm-H2-CO2-100-55KDa_DrWu_20140728 (in gel)
Scan Method Score m/z
7249 ITMS; CID 49.99 730.35
Sequence
PSMs
Fragments
Properties
- G K H V V V A P L H S R -

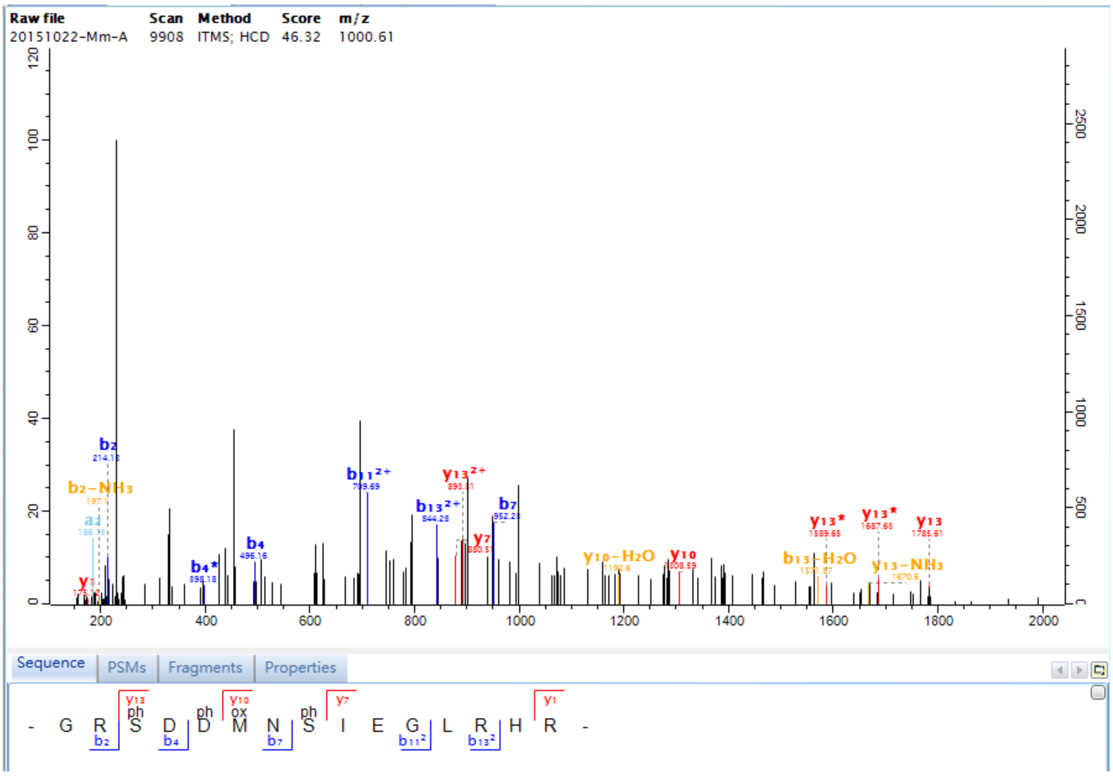
Raw file Scan Method Score m/z
20151022-Mm-A 9908 ITMS; HCD 46.32 1000.61
Sequence PSMs Fragments Properties
- G R S D D M N S I E G L R H R -

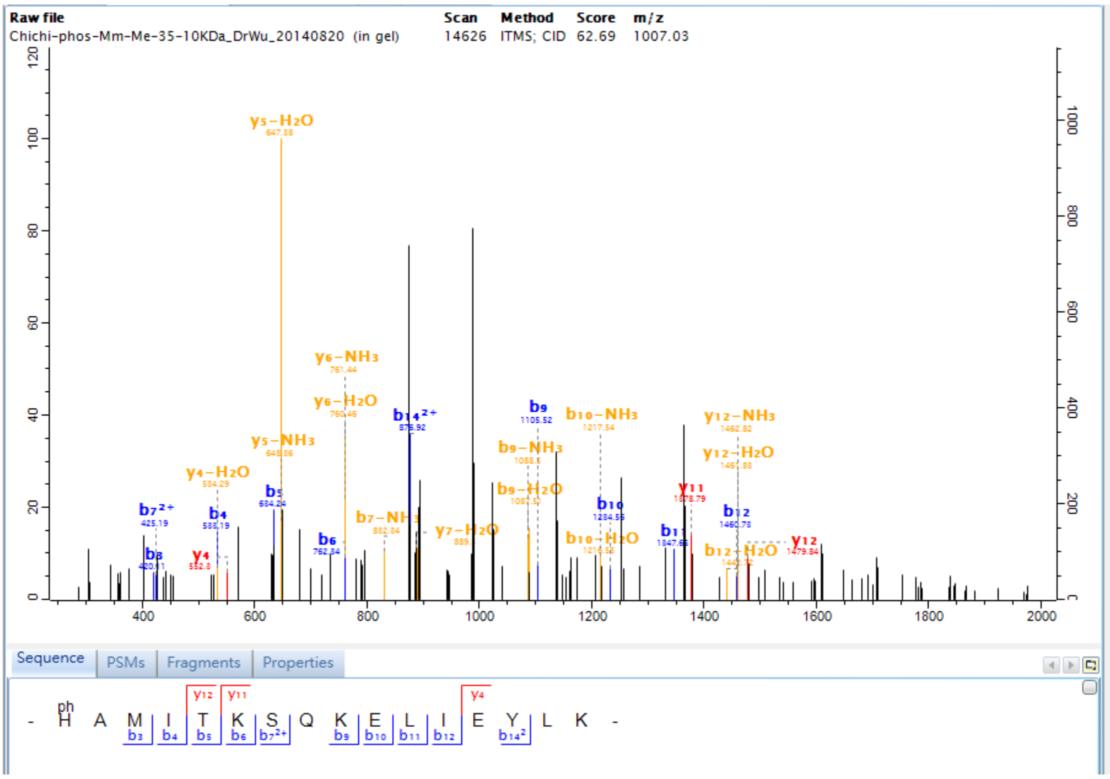
Raw file Scan Method Score m/z
Chichi-phos-Mm-Me-35-10KDa_DrWu_20140820 (in gel) 14626 ITMS; CID 62.69 1007.03
Sequence PSMs Fragments Properties
- H A M I T K S Q K E L I E Y L K -

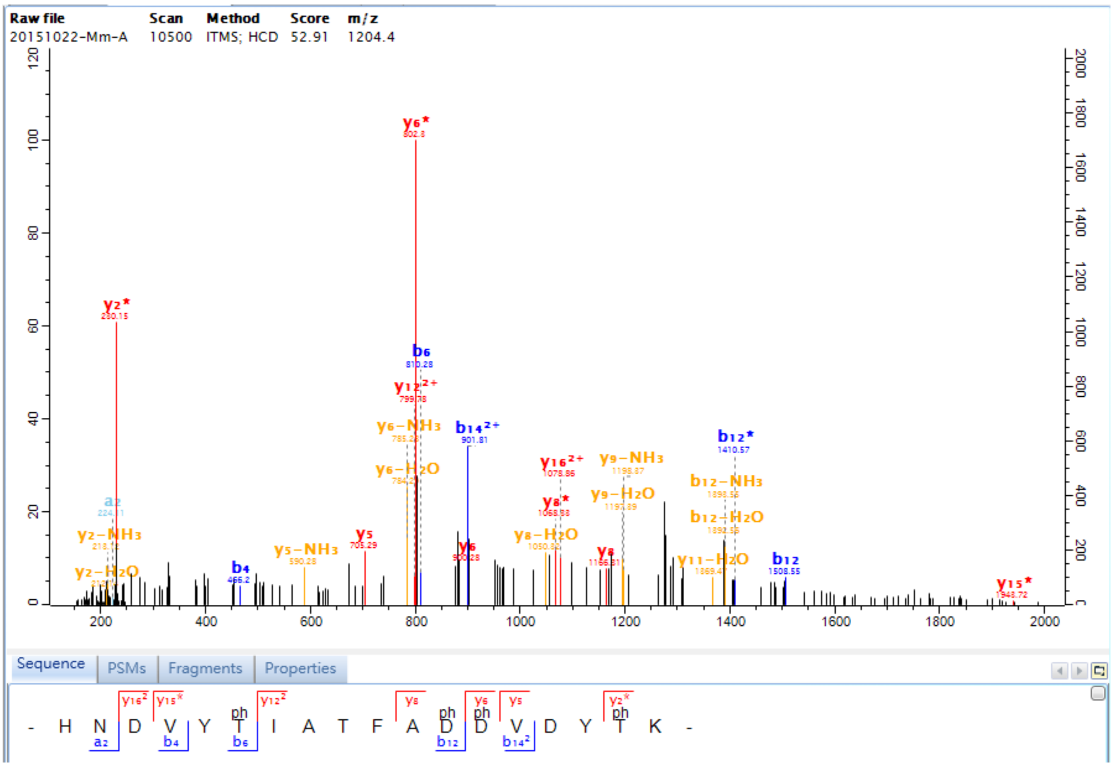
Raw file Scan Method Score m/z
20151022-Mm-A 10500 ITMS; HCD 52.91 1204.4
Sequence PSMs Fragments Properties
- H N D V Y T I A T F A D D V D Y T K -

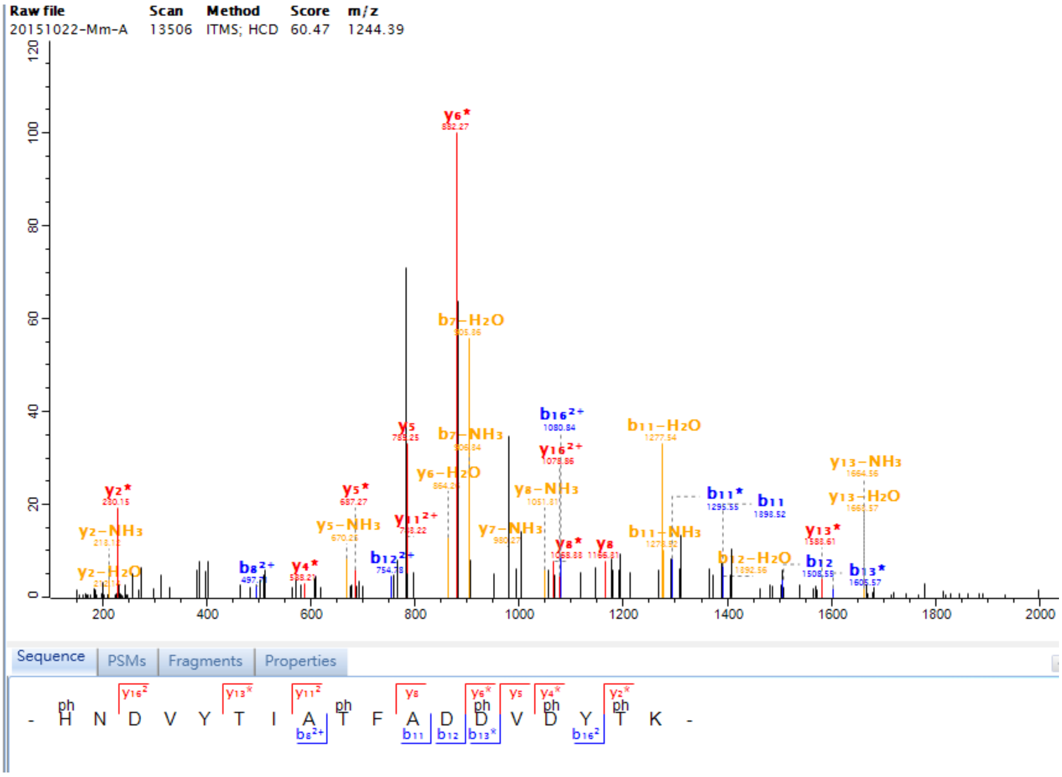
Raw file Scan Method Score m/z
20151022-Mm-A 13506 ITMS; HCD 60.47 1244.39
Sequence PSMs Fragments Properties
- H N D V Y T I A T F A D D V D Y T K -

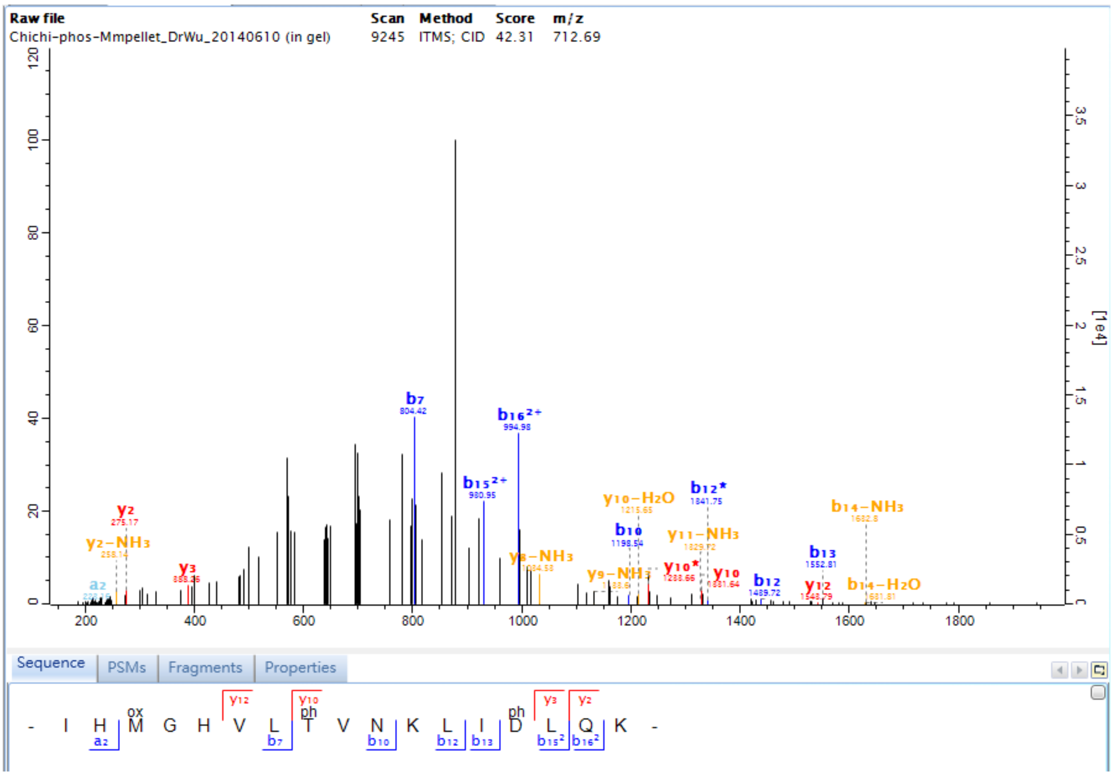
Raw file
Chichi-phos-Mmpellet_DrWu_20140610 (in gel)
Scan
9245
Method
ITMS; CID
Score
42.31
m/z
712.69
b7
804.42
b16 2+
994.98
b15 2+
y10-H2O
b12*
b14-NH3
y2
y2-NH3
y3
b10
y11-NH3
y8-NH3
b13
y9-NH3
y10*
y10
b12
y12
b14-H2O
[1e4]
Sequence
PSMs
Fragments
Properties
- I H M G H V L T V N K L I D L Q K -

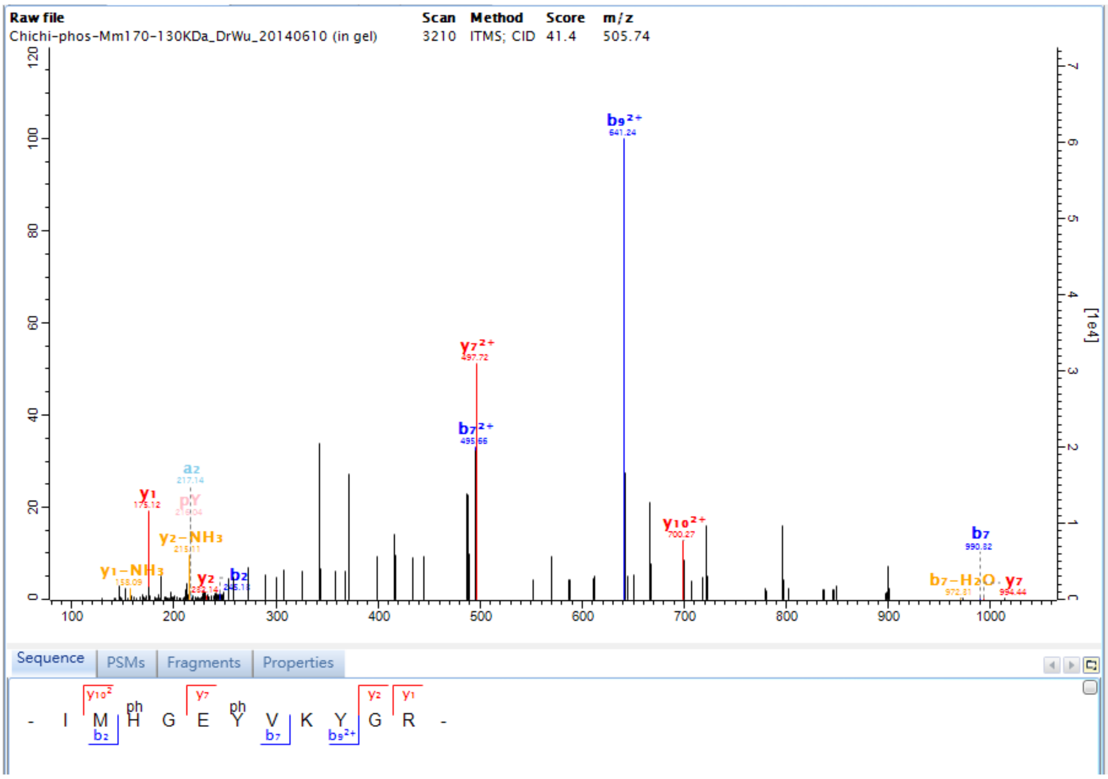
Raw file
Chichi-phos-Mm170-130KDa_DrWu_20140610 (in gel)
Scan
3210
Method
ITMS; CID
Score
41.4
m/z
505.74
b9 2+
641.24
y7 2+
497.72
b7 2+
a2
y1
pY
y2-NH3
y1-NH3
y2
b2
y10 2+
700.27
b7
990.82
b7-H2O
y7
[1e4]
Sequence
PSMs
Fragments
Properties
- I M H G E Y V K Y G R -

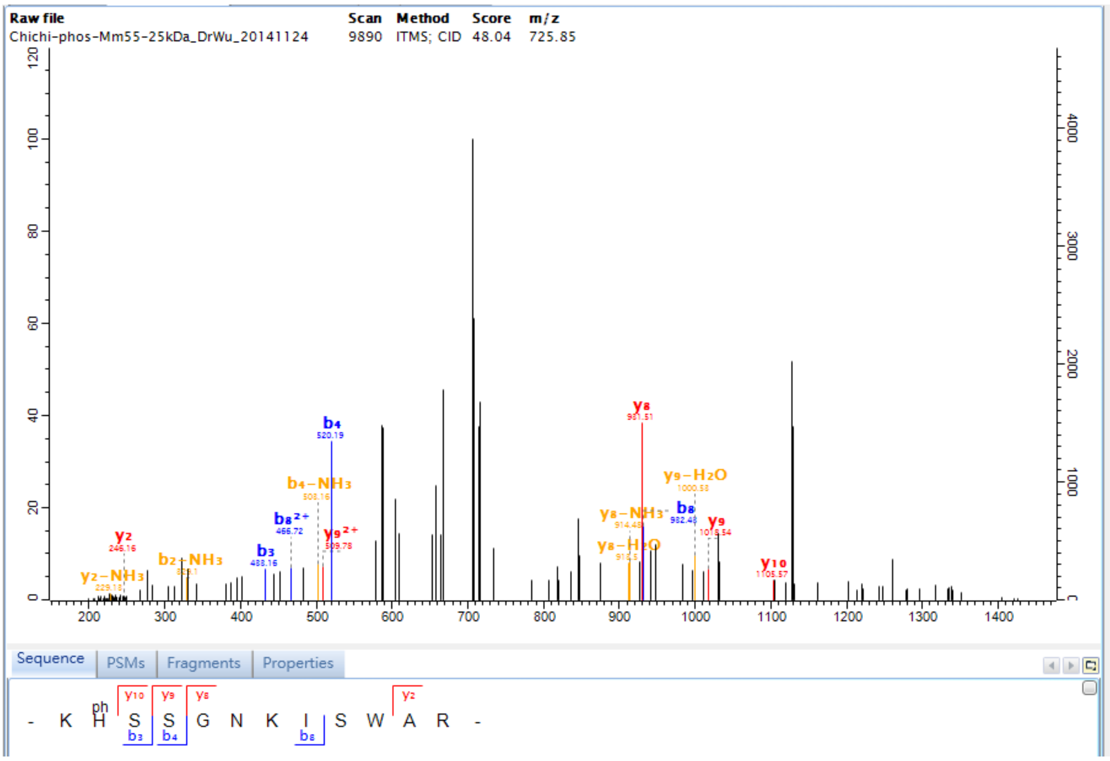
Raw file
Chichi-phos-Mm55-25kDa_DrWu_20141124
Scan Method Score m/z
9890 ITMS; CID 48.04 725.85
y8
b4
b4-NH3
y9-H2O
b8
y9
y10
b8 2+
y9 2+
b3
y2
b2-NH3
y2-NH3
y8-NH3
y8-H2O
Sequence
PSMs
Fragments
Properties
- K H S S G N K I S W A R -

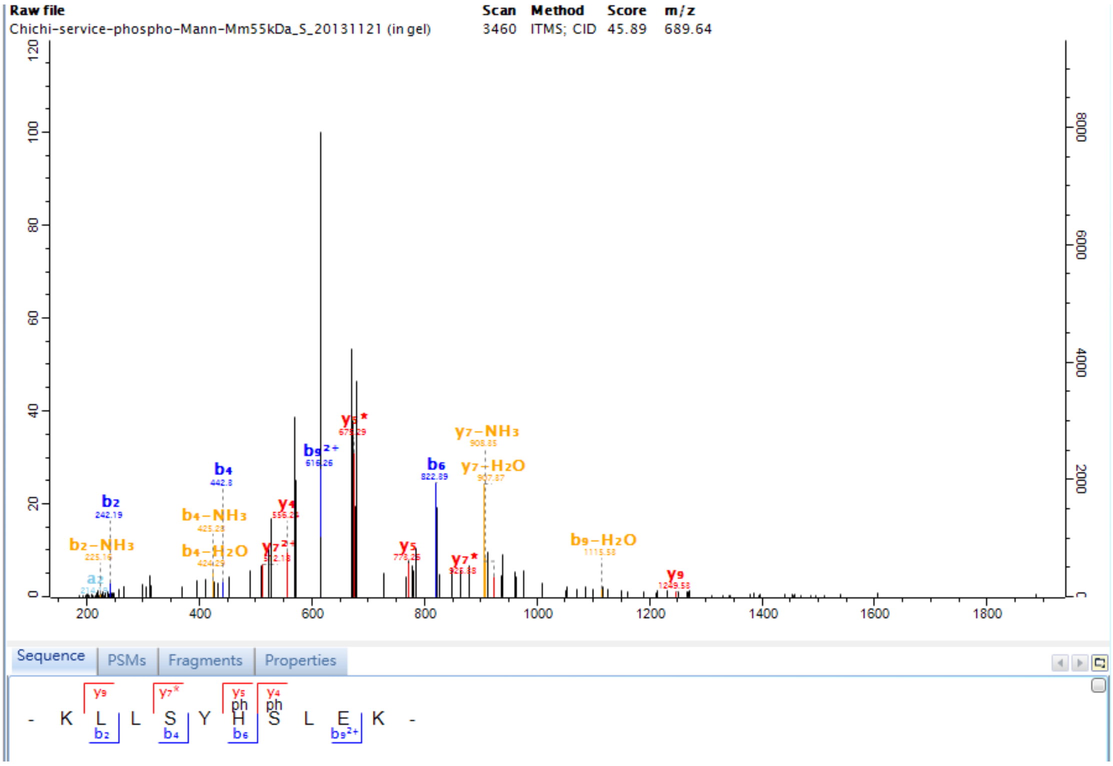
Raw file
Chichi-service-phospho-Mann-Mm55kDa_S_20131121 (in gel)
Scan Method Score m/z
3460 ITMS; CID 45.89 689.64
b2
b2-NH3
b4
b4-NH3
b4-H2O
b9 2+
b6
y7-NH3
y7-H2O
b9-H2O
y9
y5
y7*
Sequence
PSMs
Fragments
Properties
- K L L S Y H S L E K -

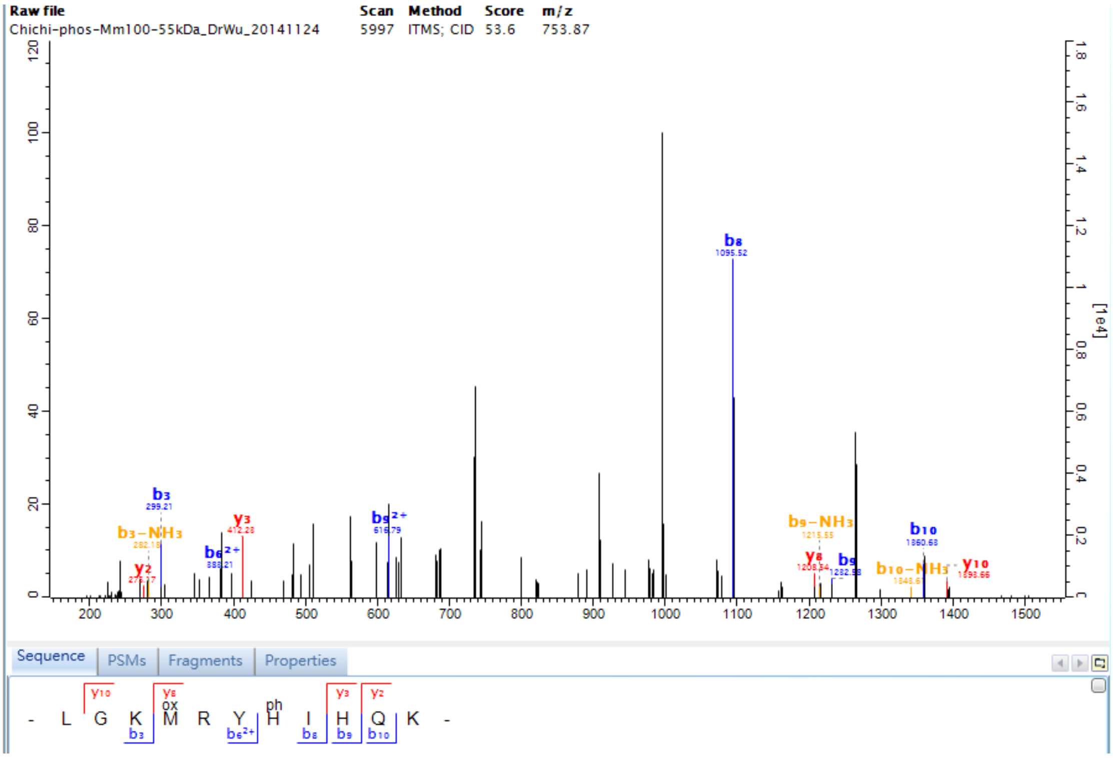
Raw file
Chichi-phos-Mm100-55kDa_DrWu_20141124
Scan 5997
Method ITMS; CID
Score 53.6
m/z 753.87
[1e4]
Sequence
PSMs
Fragments
Properties
- L G K M R Y H I H Q K -

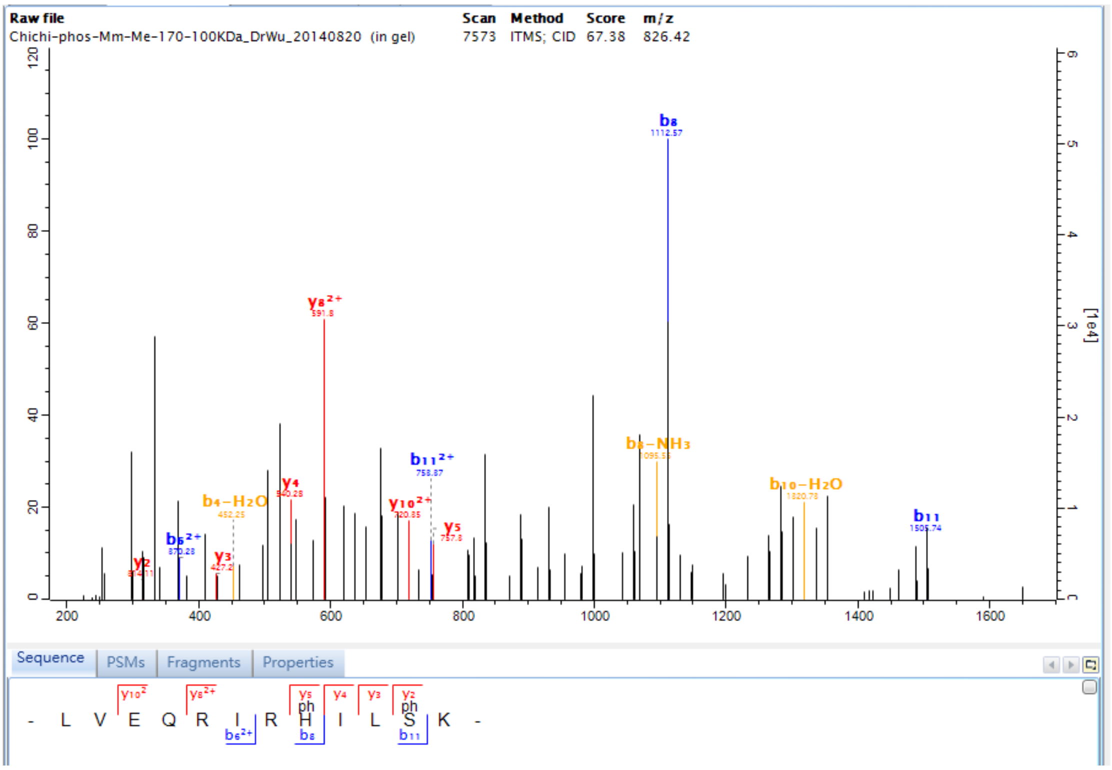
Raw file
Chichi-phos-Mm-Me-170-100KDa_DrWu_20140820 (in gel)
Scan 7573
Method ITMS; CID
Score 67.38
m/z 826.42
[1e4]
Sequence
PSMs
Fragments
Properties
- L V E Q R I R H I L S K -

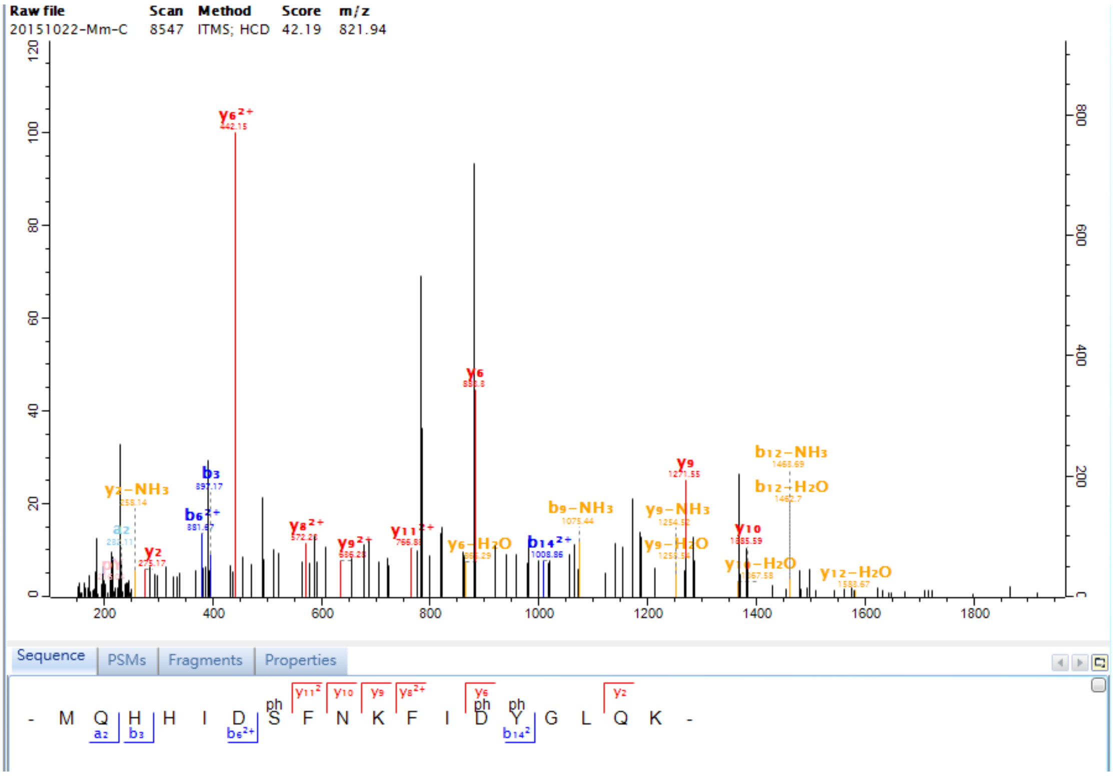
Raw file Scan Method Score m/z
20151022-Mm-C 8547 ITMS; HCD 42.19 821.94
Sequence PSMs Fragments Properties
- M Q H H I D S F N K F I D Y G L Q K -

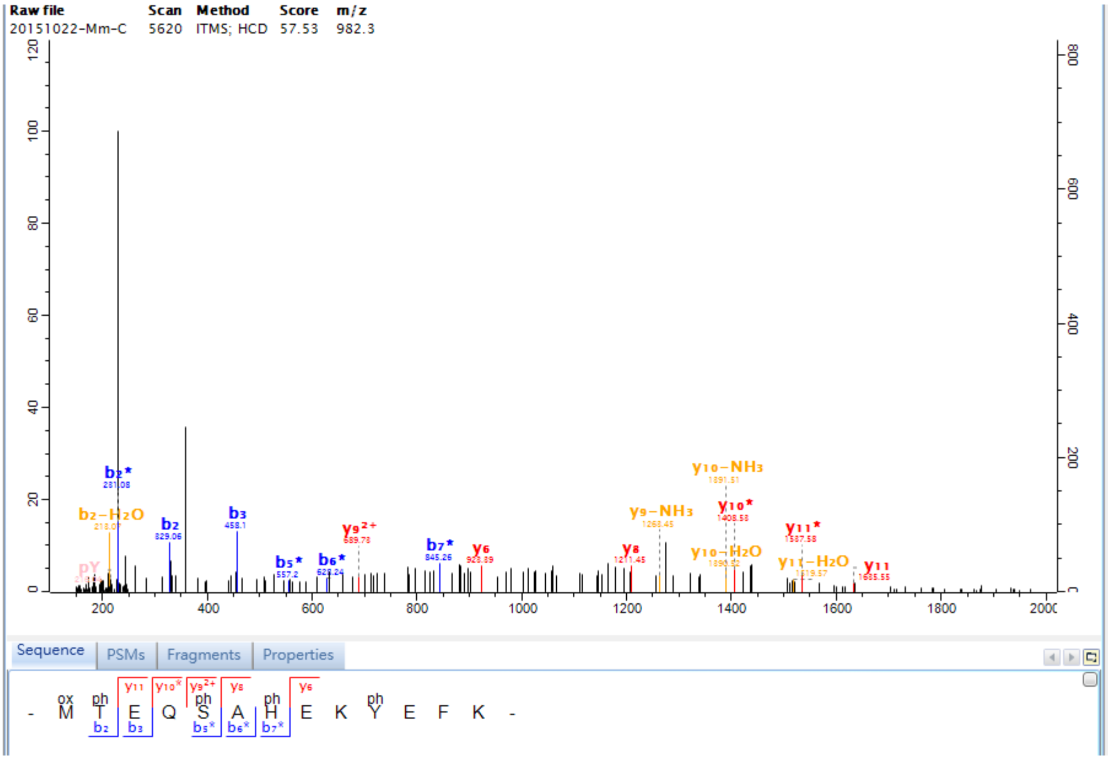
Raw file Scan Method Score m/z
20151022-Mm-C 5620 ITMS; HCD 57.53 982.3
Sequence PSMs Fragments Properties
- M T E Q S A H E K Y E F K -

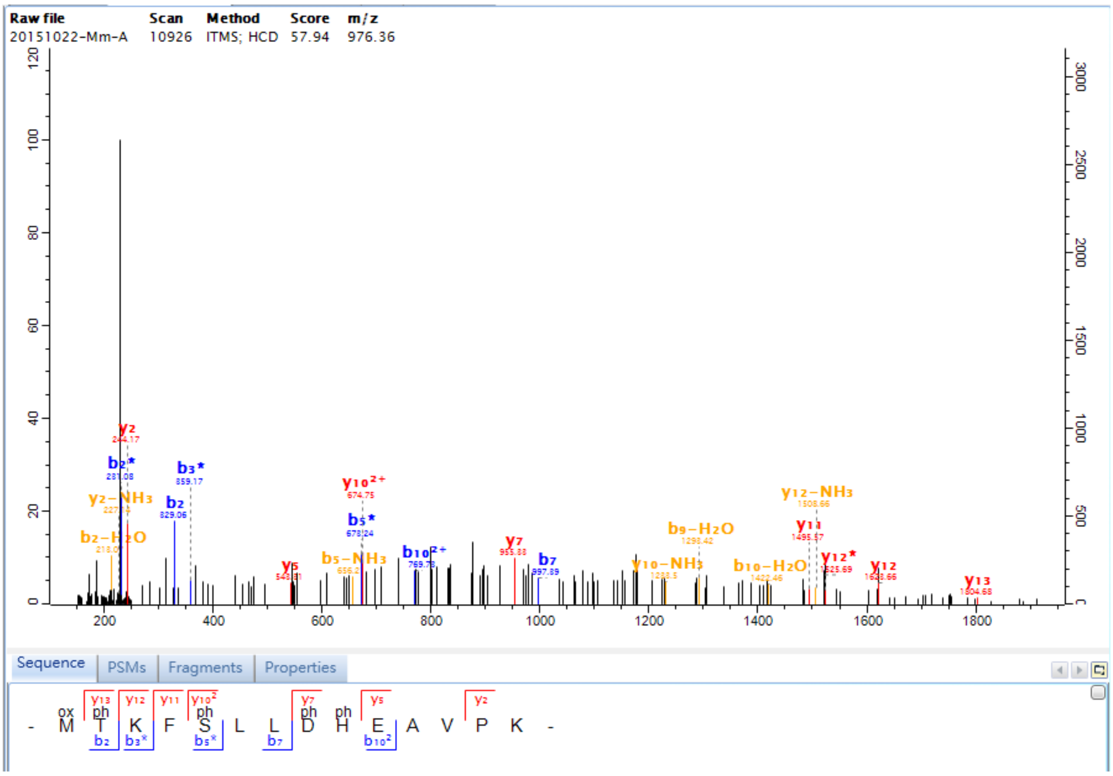
Raw file
20151022-Mm-A
Scan
10926
Method
ITMS; HCD
Score
57.94
m/z
976.36
Sequence
PSMs
Fragments
Properties
- M T K F S L L D H E A V P K -

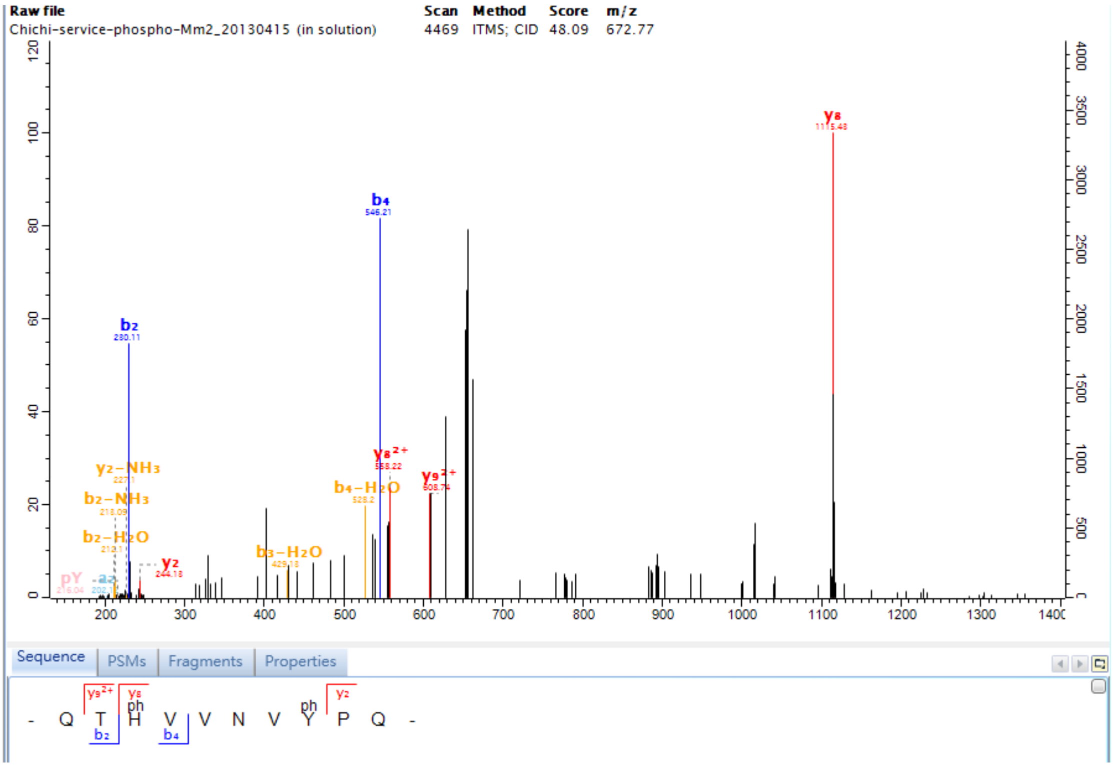
Raw file
Chichi-service-phospho-Mm2_20130415 (in solution)
Scan
4469
Method
ITMS; CID
Score
48.09
m/z
672.77
Sequence
PSMs
Fragments
Properties
- Q T H V V N V Y P Q -

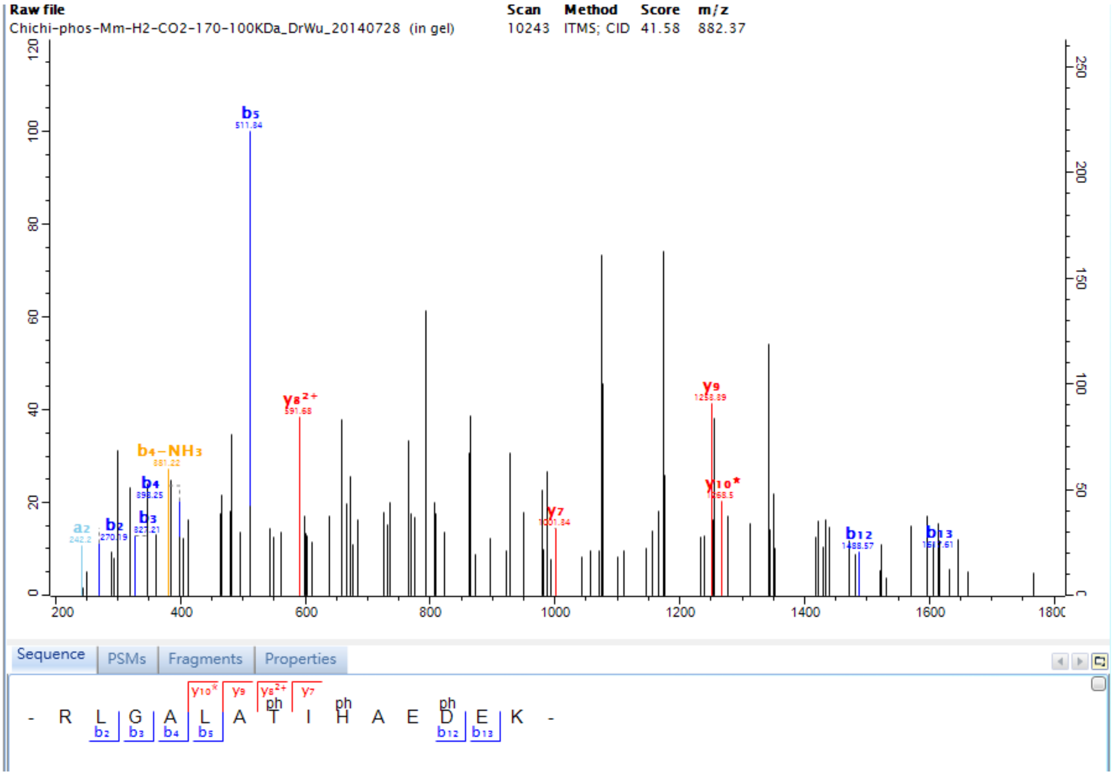
Raw file
Chichi-phos-Mm-H2-CO2-170-100KDa_DrWu_20140728 (in gel)
Scan 10243
Method ITMS; CID
Score 41.58
m/z 882.37
Sequence
PSMs
Fragments
Properties
- R L G A L A T I H A E D E K -

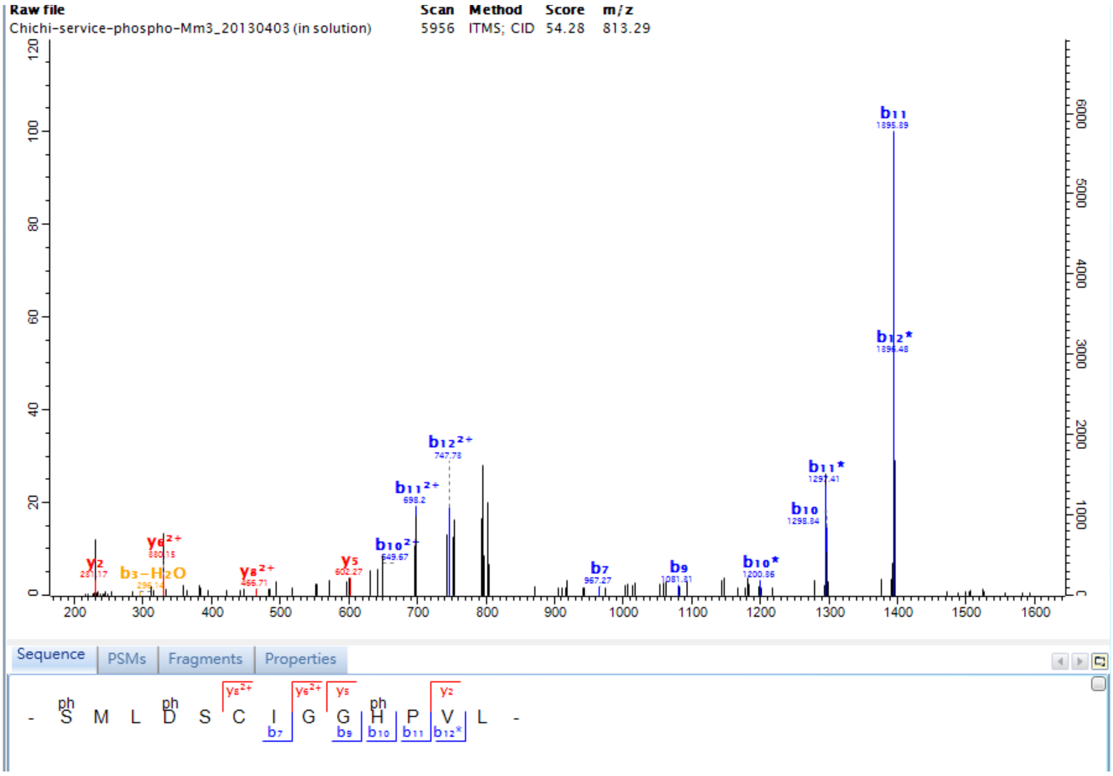
Raw file
Chichi-service-phospho-Mm3_20130403 (in solution)
Scan 5956
Method ITMS; CID
Score 54.28
m/z 813.29
Sequence
PSMs
Fragments
Properties
- S M L D S C I G G H P V L -

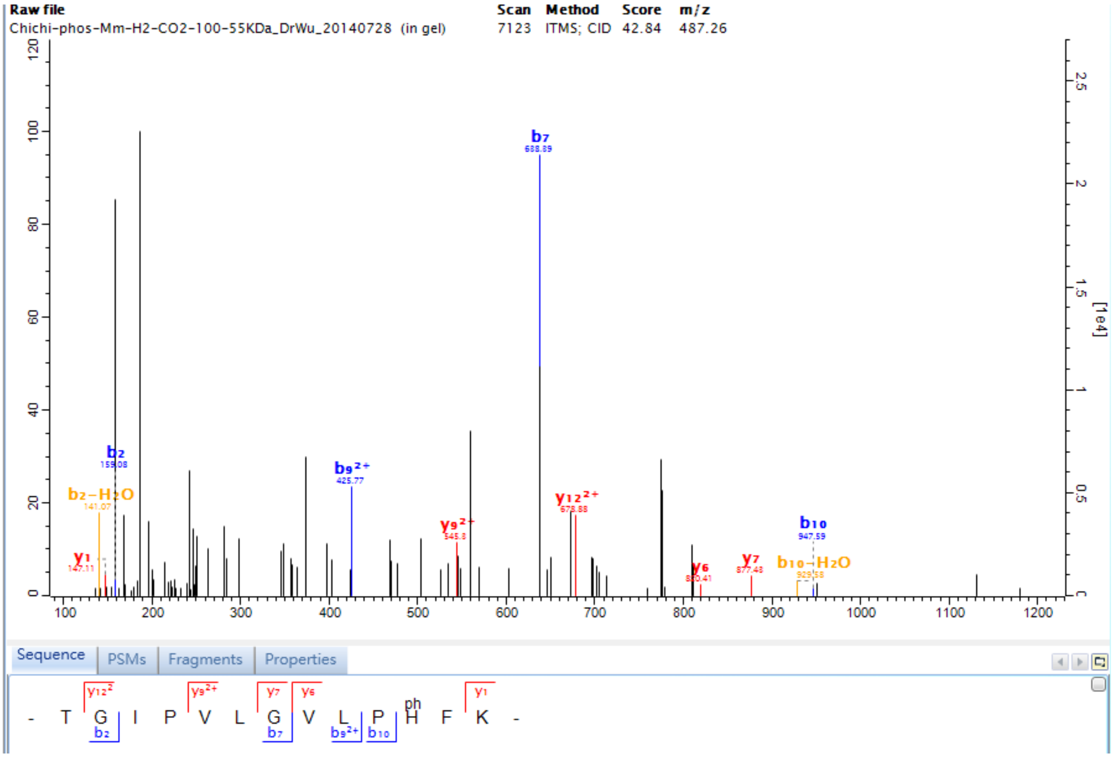
Raw file
Chichi-phos-Mm-H2-CO2-100-55KDa_DrWu_20140728 (in gel)
Scan Method Score m/z
7123 ITMS; CID 42.84 487.26
b7
b9 2+
b2
b2-H2O
y1
y9 2+
y12 2+
b10
y6
y7
b10-H2O
[1e4]
Sequence
PSMs
Fragments
Properties
- T G I P V L G V L P H F K -

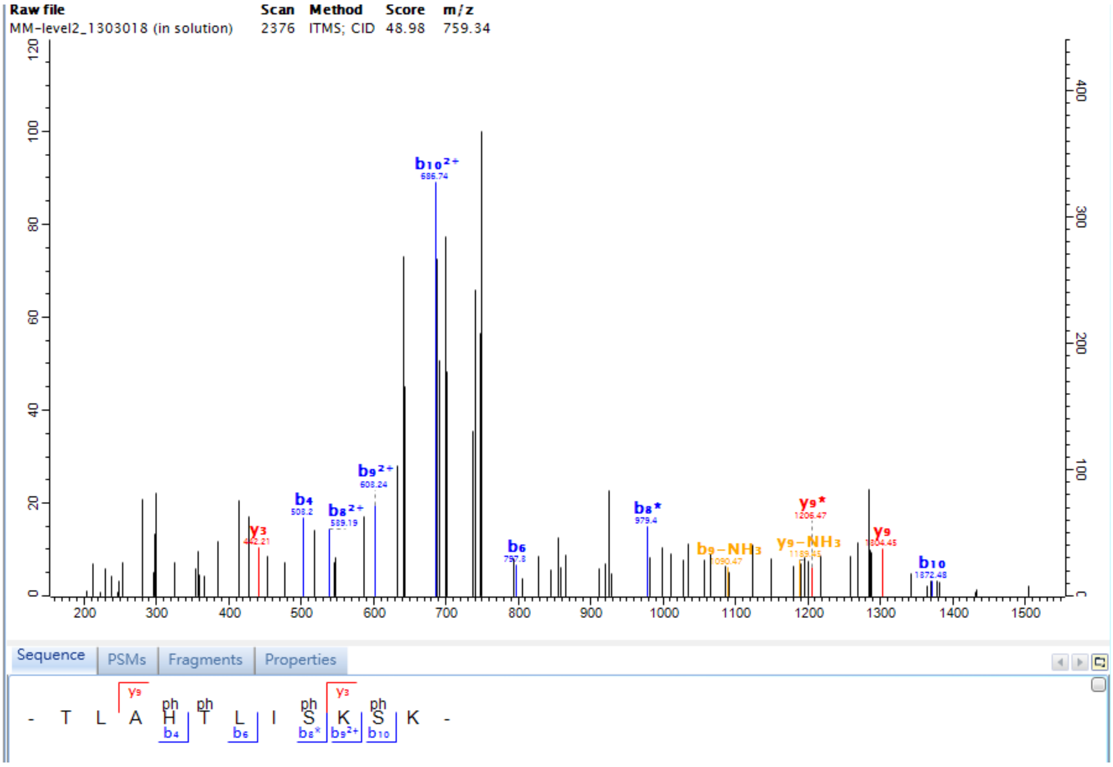
Raw file
MM-level2_1303018 (in solution)
Scan Method Score m/z
2376 ITMS; CID 48.98 759.34
b10 2+
b9 2+
b4
b8 2+
y3
b6
b8*
b9-NH3
y9*
y9-NH3
y9
b10
Sequence
PSMs
Fragments
Properties
- T L A H T L I S K S K -

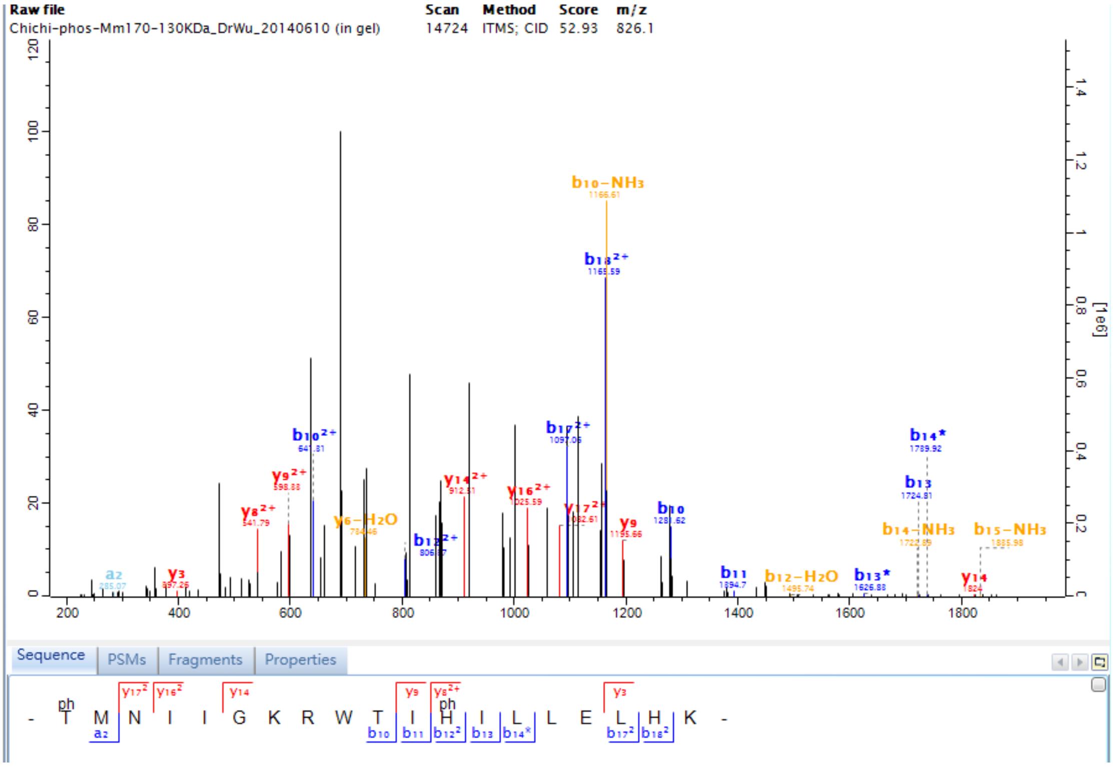
Raw file
Chichi-phos-Mm170-130KDa_DrWu_20140610 (in gel)
Scan 14724
Method ITMS; CID
Score 52.93
m/z 826.1
b10-NH3
b18 2+
b10 2+
y9 2+
y8 2+
y6-H2O
b17 2+
y14 2+
y16 2+
y17 2+
b12 2+
y9
b10
b14*
b13
b14-NH3
b15-NH3
a2
y3
b11
b12-H2O
b13*
y14
[1e6]
Sequence
PSMs
Fragments
Properties
- T M N I I G K R W T I H I L L E L H K -

Methanohalophilus portucalensis FDF1[T] (methanogenic archaeon)

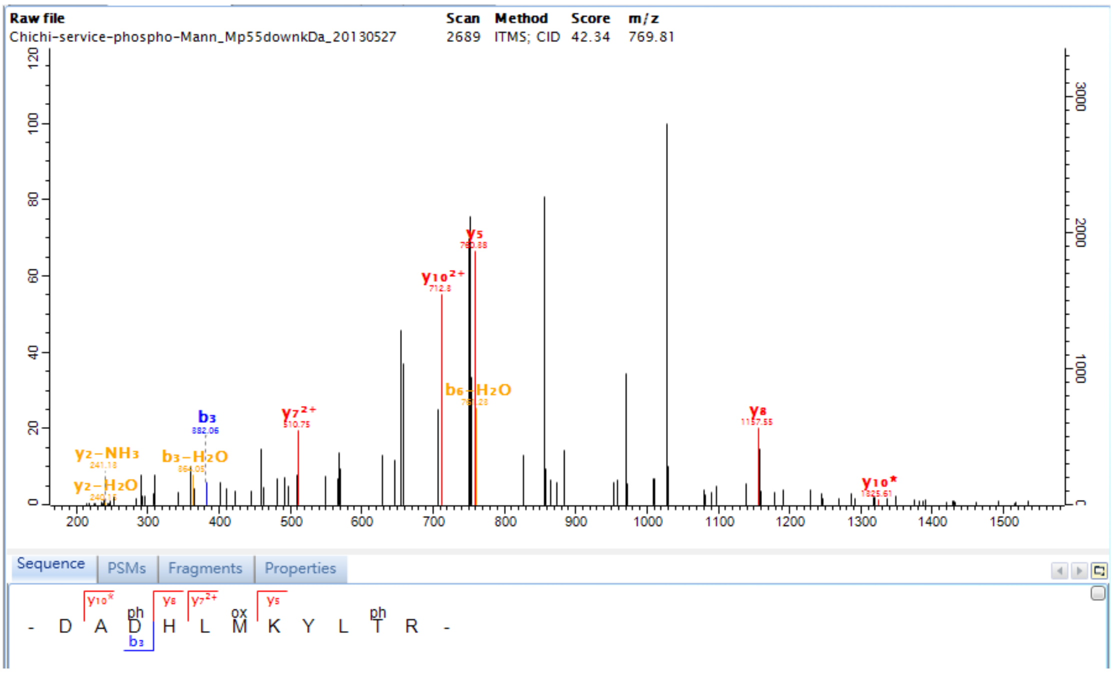

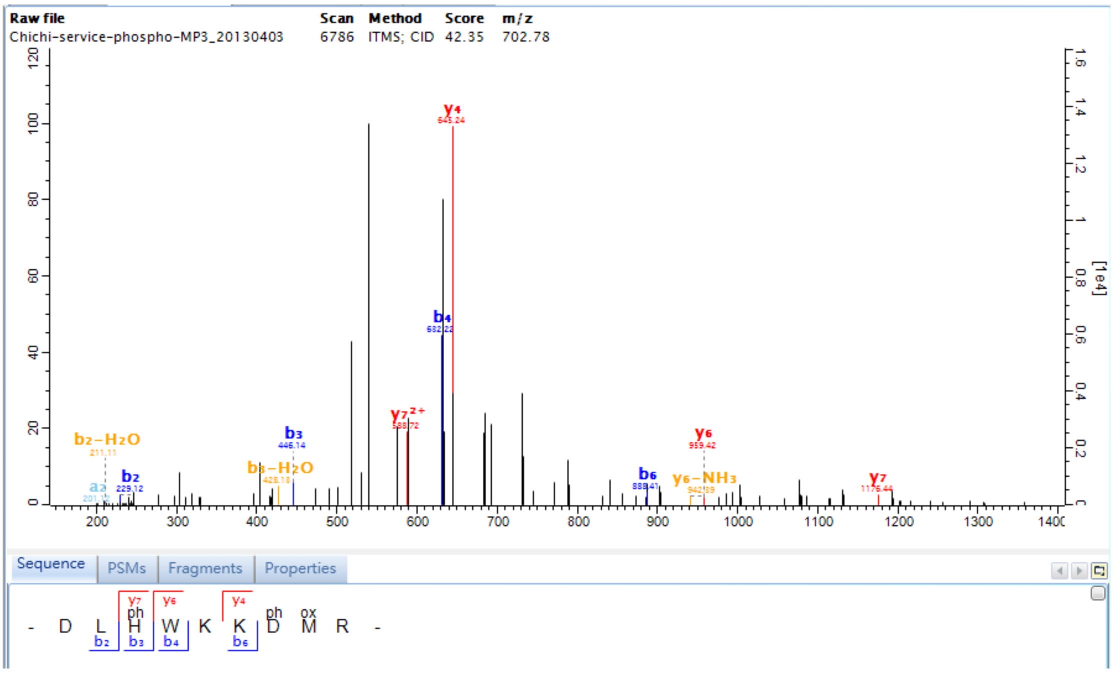

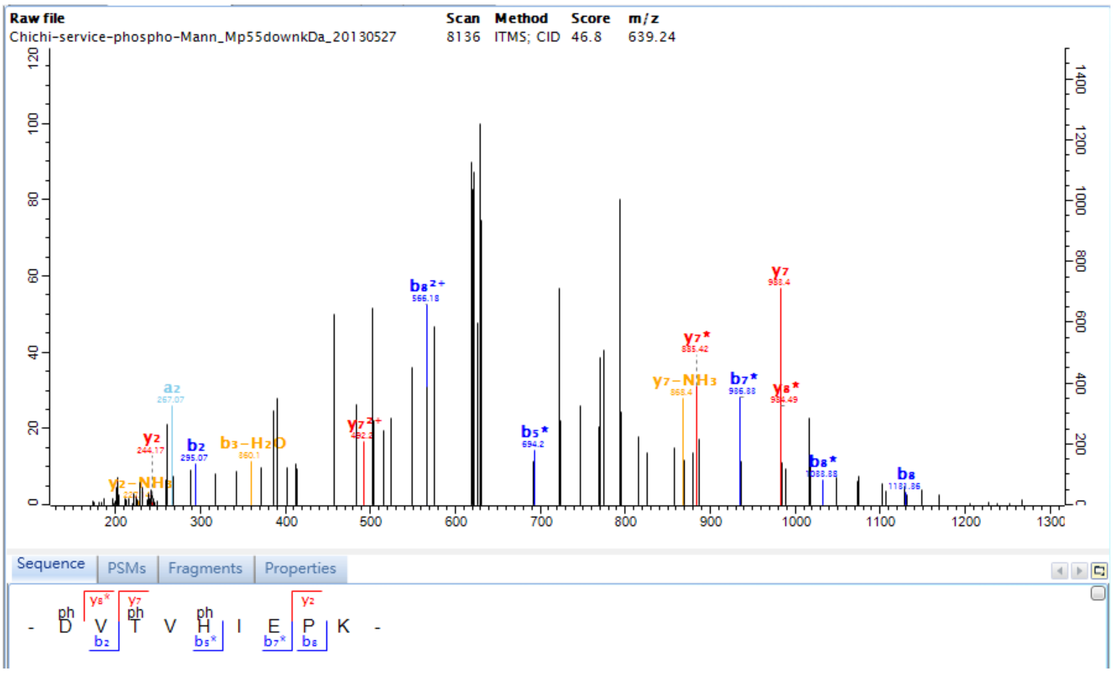
Raw file
Chichi-service-phospho-Mann_Mp55downkDa_20130527
Scan Method Score m/z
8136 ITMS; CID 46.8 639.24
Sequence PSMs Fragments Properties
- D V T V H I E P K -

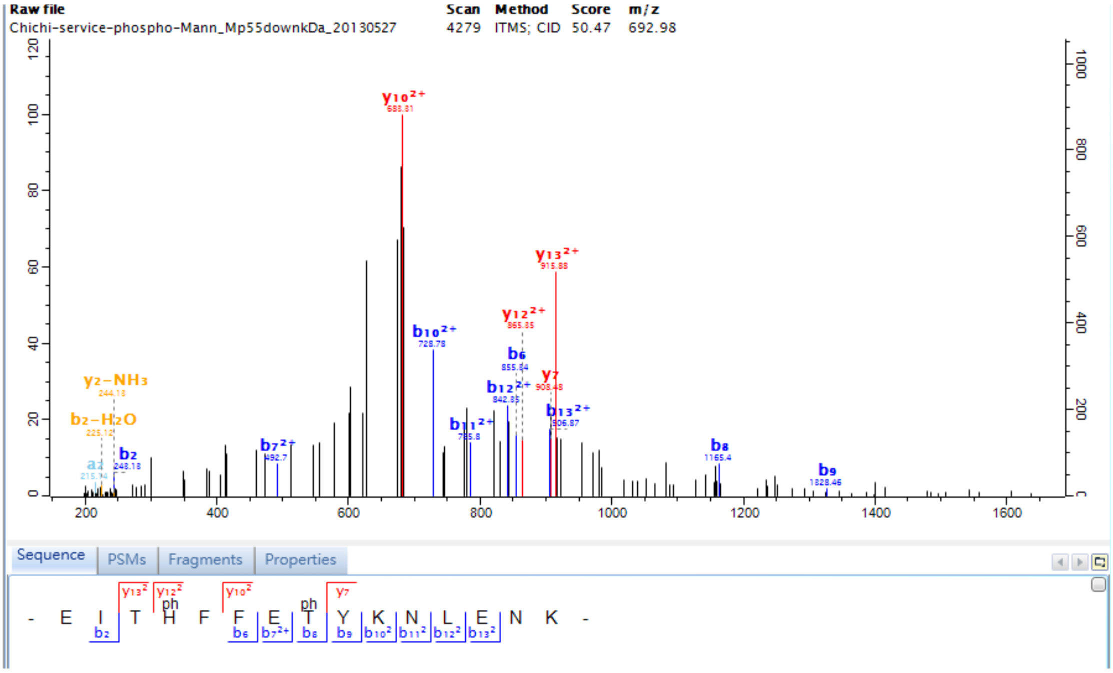
Raw file
Chichi-service-phospho-Mann_Mp55downkDa_20130527
Scan Method Score m/z
4279 ITMS; CID 50.47 692.98
Sequence PSMs Fragments Properties
- E I T H F F E T Y K N L E N K -

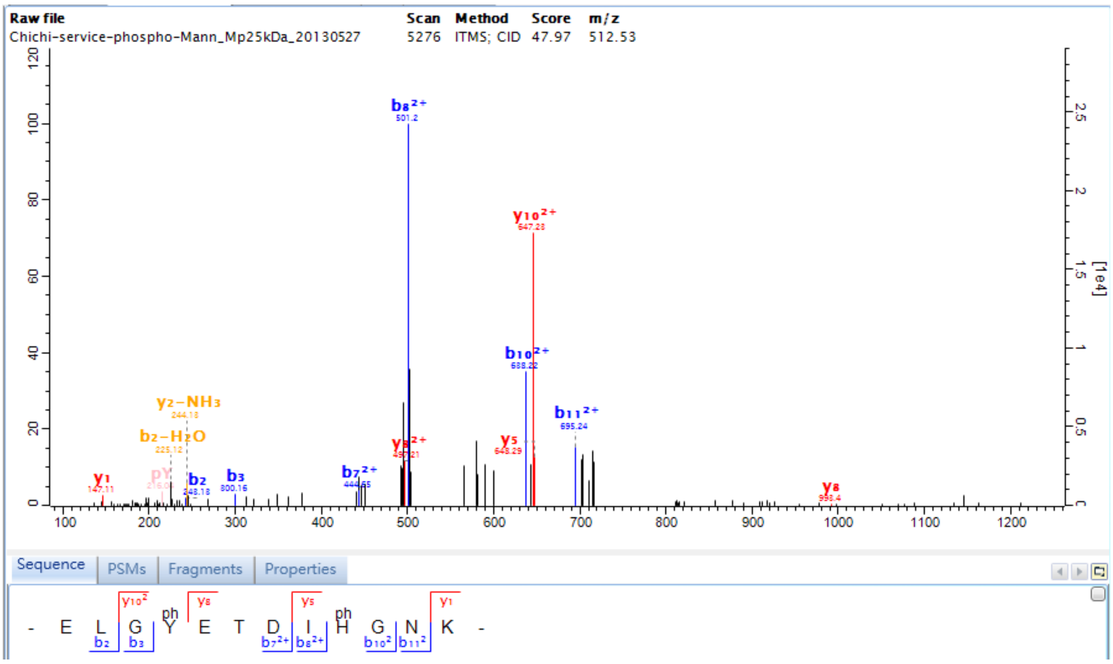
Raw file
Chichi-service-phospho-Mann_Mp25kDa_20130527
Scan Method Score m/z
5276 ITMS; CID 47.97 512.53
b8 2+ 501.2
y10 2+ 647.28
b10 2+
b11 2+ 695.24
y2-NH3
b2-H2O
y1
b2
b3 300.16
b7 2+
y5
y8
[1e4]
Sequence PSMs Fragments Properties
- E L G Y E T D I H G N K -

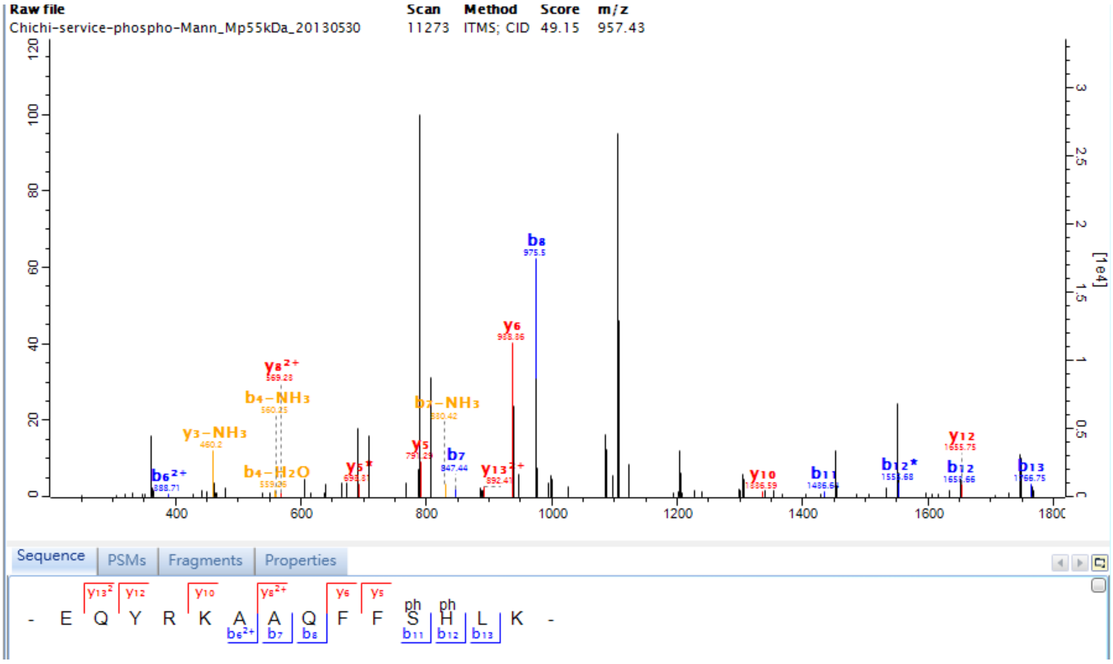
Raw file
Chichi-service-phospho-Mann_Mp55kDa_20130530
Scan Method Score m/z
11273 ITMS; CID 49.15 957.43
b8 975.5
y6
y8 2+
b4-NH3
y3-NH3
b7-NH3
b4-H2O
b6 2+
y5
b7
y10
b11
b12
y12
b13
[1e4]
Sequence PSMs Fragments Properties
- E Q Y R K A A Q F F S H L K -

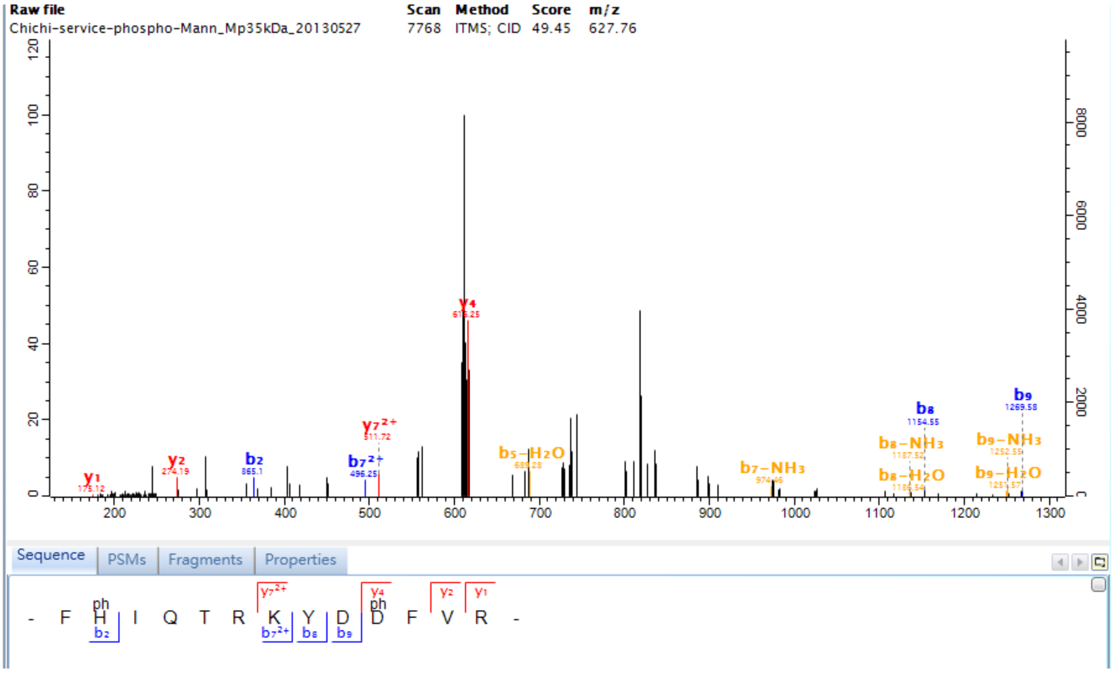
Raw file
Chichi-service-phospho-Mann_Mp35kDa_20130527
Scan Method Score m/z
7768 ITMS; CID 49.45 627.76
y4
y7²⁺
b7²⁺
b2
y2
y1
b5-H2O
b7-NH3
b8
b8-NH3
b8-H2O
b9
b9-NH3
b9-H2O
Sequence
PSMs
Fragments
Properties
- F H I Q T R K Y D D F V R -

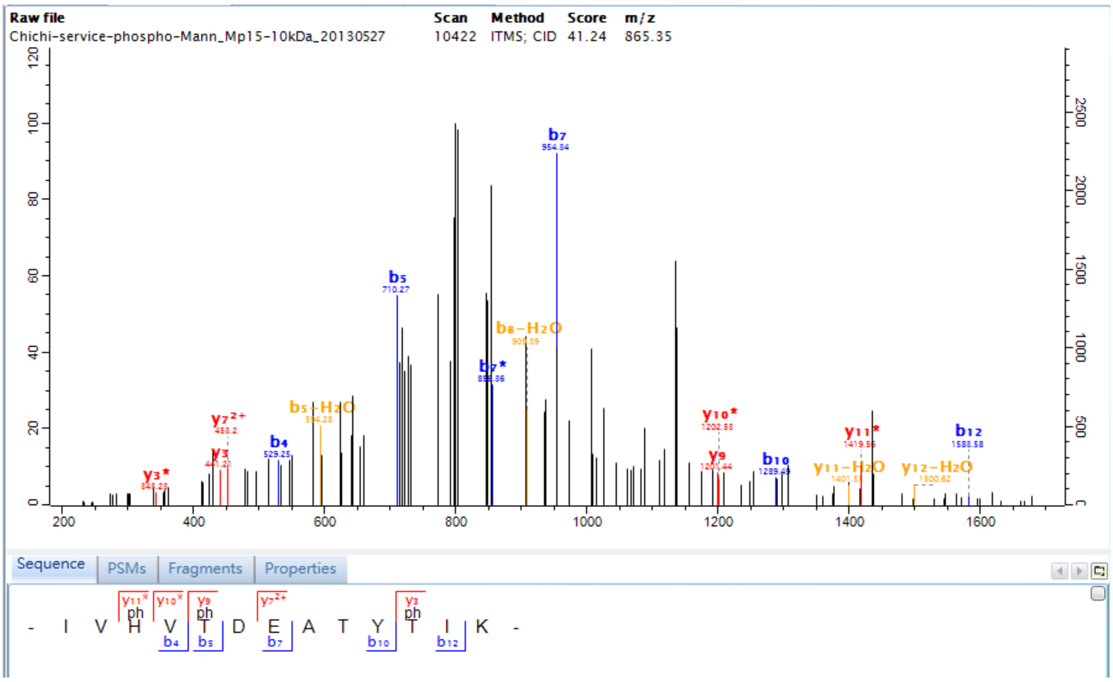
Raw file
Chichi-service-phospho-Mann_Mp15-10kDa_20130527
Scan Method Score m/z
10422 ITMS; CID 41.24 865.35
b7
b5
b8-H2O
b7*
b5-H2O
y7²⁺
y3
y3*
b4
y10*
y9
b10
y11*
y11-H2O
y12-H2O
b12
Sequence
PSMs
Fragments
Properties
- I V H V T D E A T Y T I K -

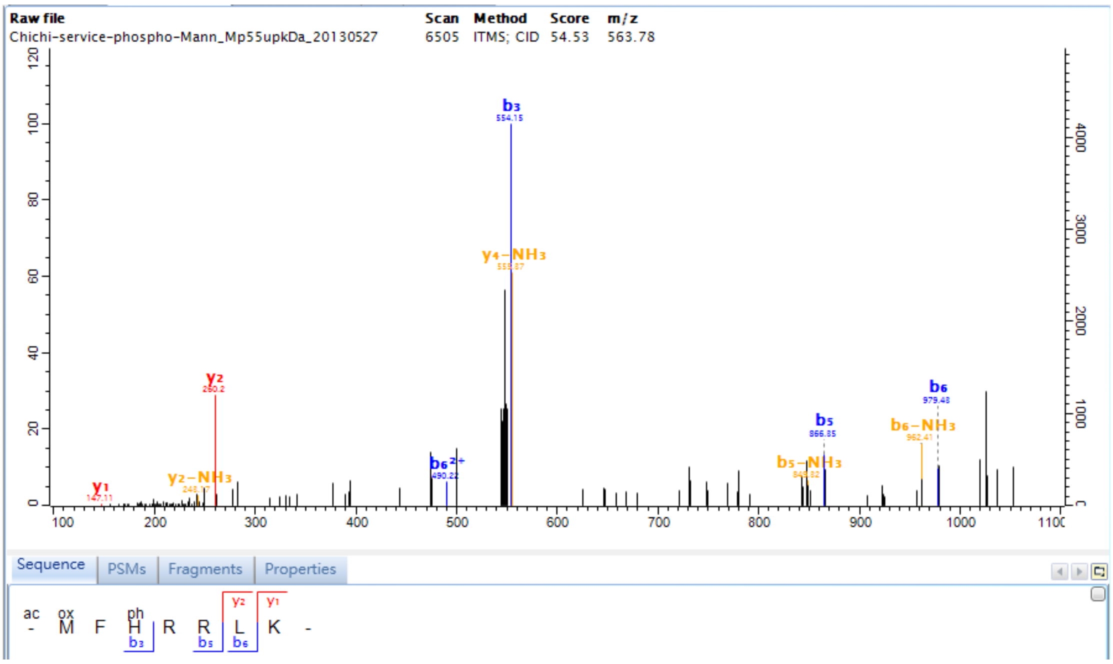
Raw file
Chichi-service-phospho-Mann_Mp55upkDa_20130527
Scan Method Score m/z
6505 ITMS; CID 54.53 563.78
b3
y4-NH3
y2
b5
b6
b6-NH3
b5-NH3
y2-NH3
y1
Sequence PSMs Fragments Properties
ac - M F H R R L K -
ox ph
b3 b5 b6 y2 y1

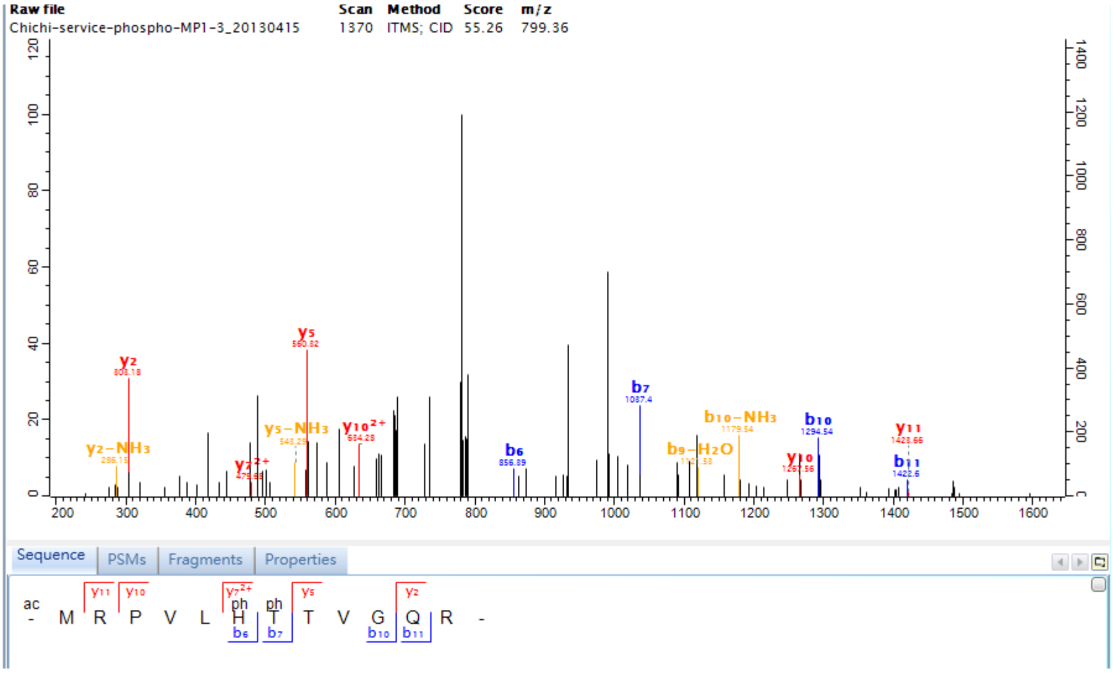
Raw file
Chichi-service-phospho-MP1-3_20130415
Scan Method Score m/z
1370 ITMS; CID 55.26 799.36
y2
y5
y2-NH3
y5-NH3
y10 2+
b6
b7
b9-H2O
b10-NH3
b10
y10
y11
b11
Sequence PSMs Fragments Properties
ac - M R P V L H T T V G Q R -
ph ph
y11 y10 y7 2+ y5 y2
b6 b7 b10 b11

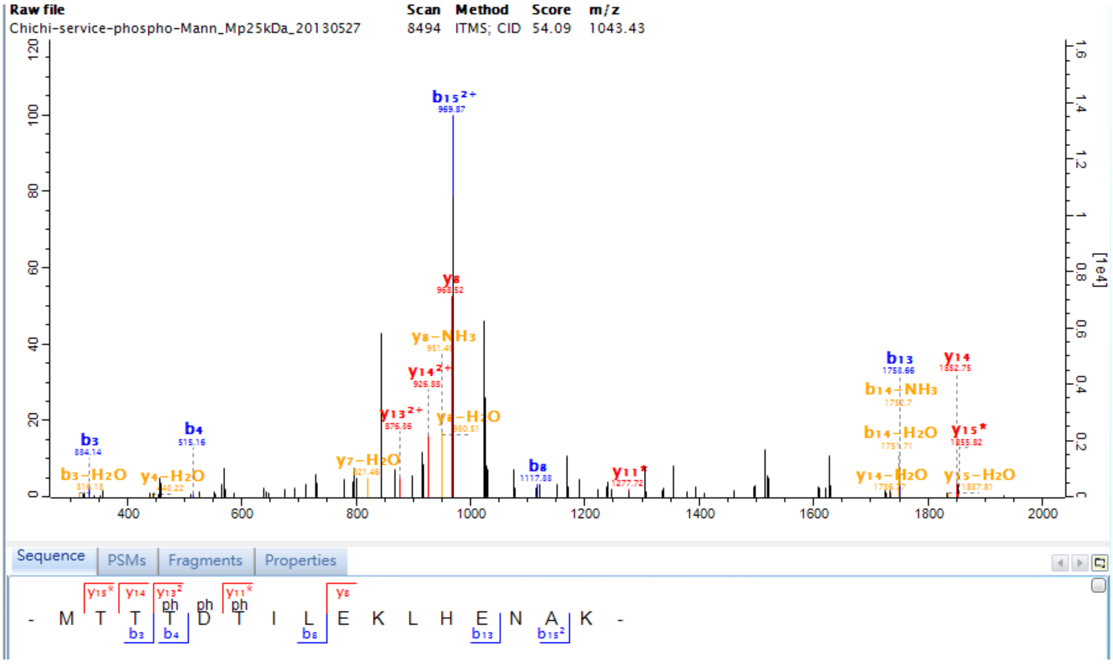
Raw file
Chichi-service-phospho-Mann_Mp25kDa_20130527
Scan
8494
Method
ITMS; CID
Score
54.09
m/z
1043.43
[1e4]
b15 2+
y8
y8-NH3
y14 2+
y13 2+
y8-H2O
y7-H2O
b13
y14
b14-NH3
b14-H2O
y15*
y14-H2O
y15-H2O
b3
b4
b3-H2O
y4-H2O
b8
y11*
Sequence
PSMs
Fragments
Properties
- M T T T D T I L E K L H E N A K -
ph ph ph

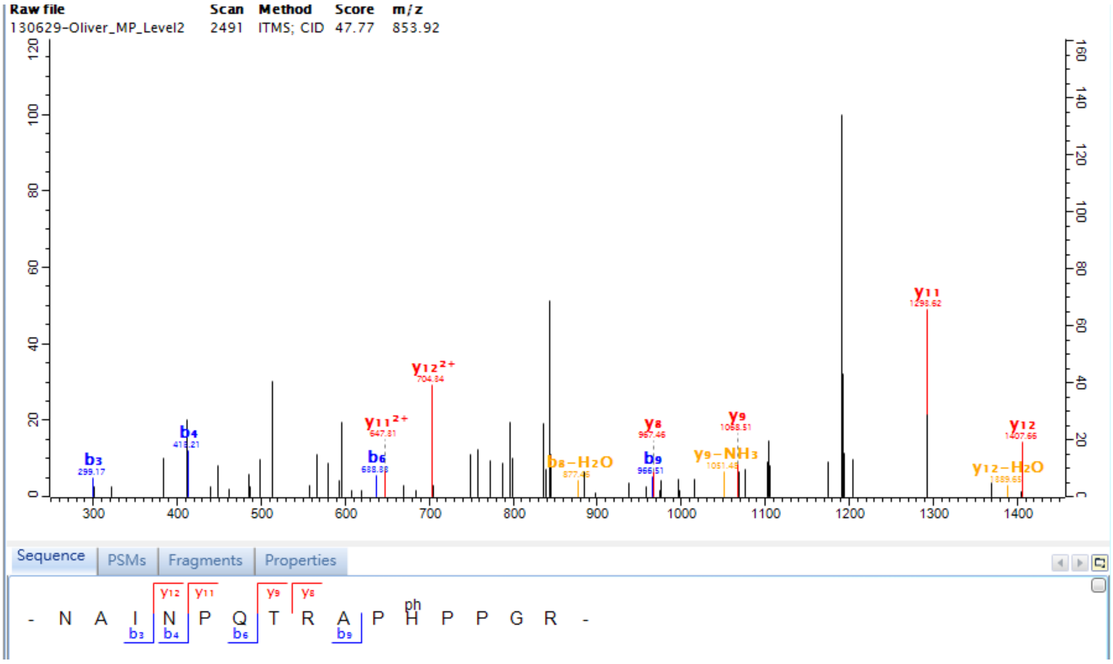
Raw file
130629-Oliver_MP_Level2
Scan
2491
Method
ITMS; CID
Score
47.77
m/z
853.92
y11
y12 2+
y11 2+
y8
y9
y9-NH3
b8-H2O
y12
y12-H2O
b3
b4
b6
b9
Sequence
PSMs
Fragments
Properties
- N A I N P Q T R A P H P P G R -
ph

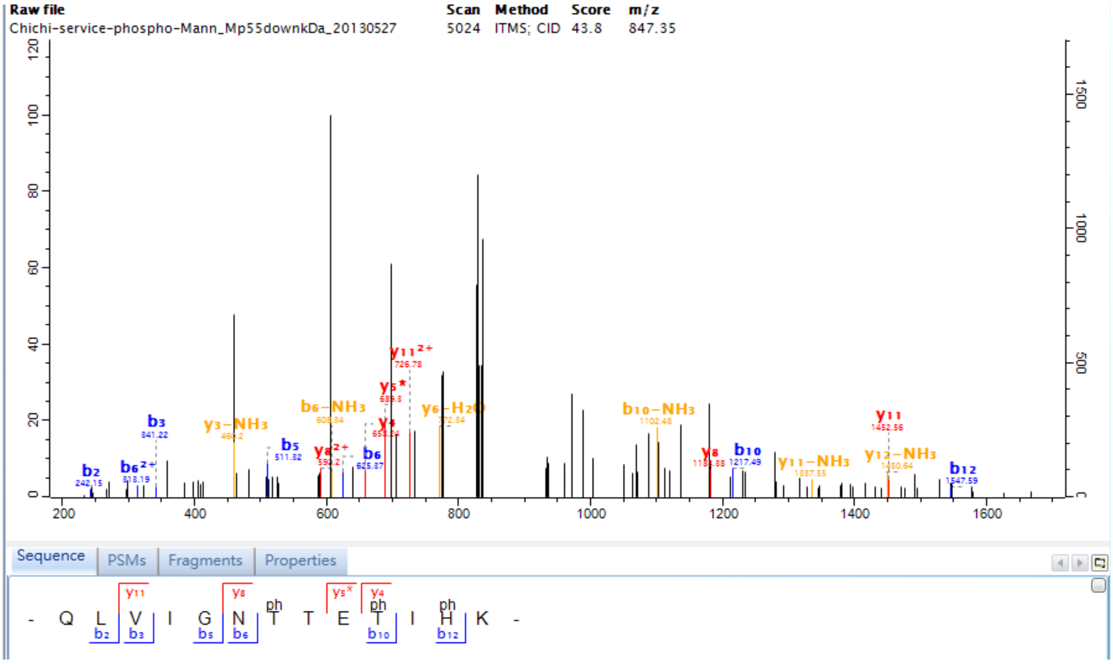
Raw file
Chichi-service-phospho-Mann_Mp55downkDa_20130527
Scan Method Score m/z
5024 ITMS; CID 43.8 847.35
Sequence
PSMs
Fragments
Properties
- Q L V I G N T T E T I H K -

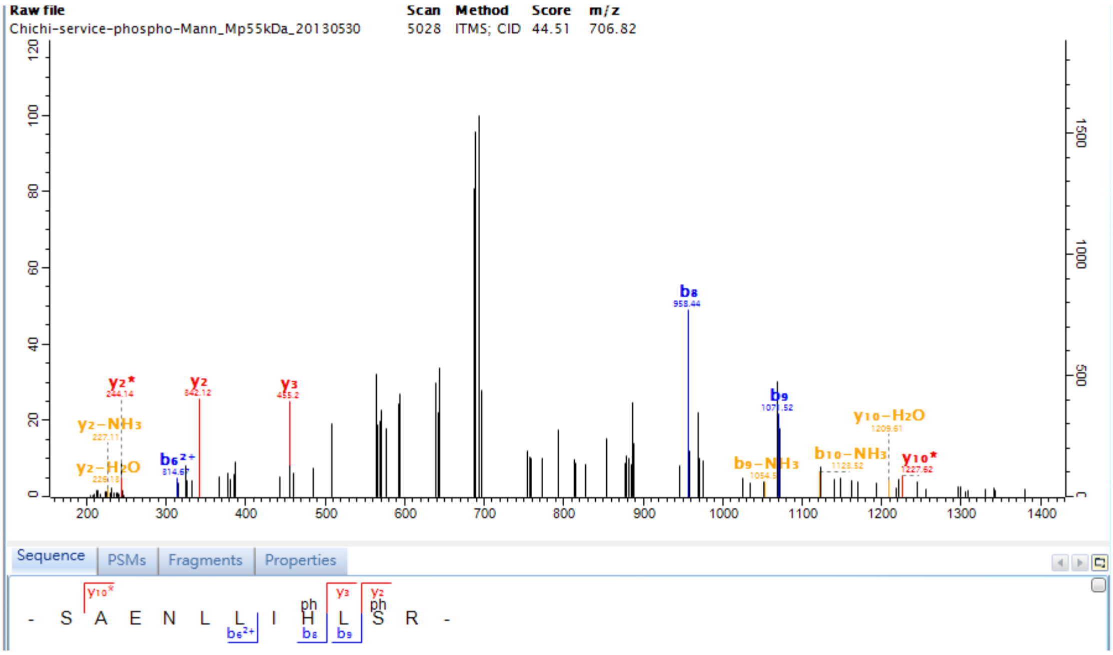
Raw file
Chichi-service-phospho-Mann_Mp55kDa_20130530
Scan Method Score m/z
5028 ITMS; CID 44.51 706.82
Sequence
PSMs
Fragments
Properties
- S A E N L L I H L S R -

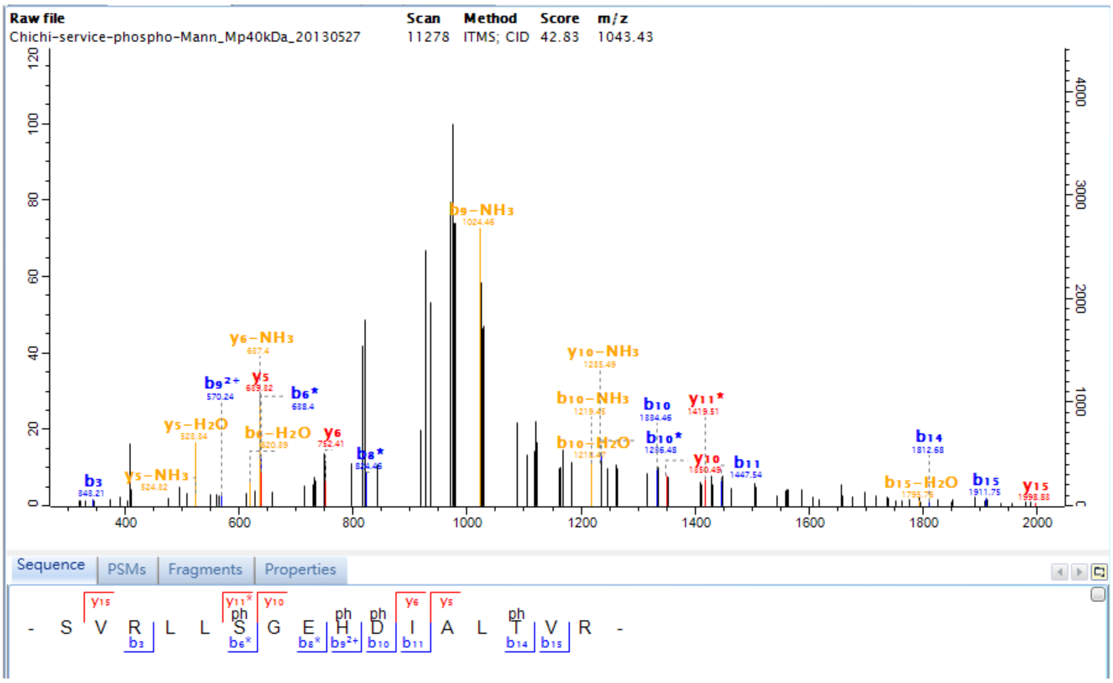
Raw file
Chichi-service-phospho-Mann_Mp40kDa_20130527
Scan Method Score m/z
11278 ITMS; CID 42.83 1043.43
b9-NH3
y6-NH3
y10-NH3
y5
b9 2+
b6*
y5-H2O
b6-H2O
y6
b8*
b10-NH3
b10
y11*
b10-H2O
b10*
y10
b11
b14
y5-NH3
b3
b15-H2O
b15
y15
Sequence
PSMs
Fragments
Properties
- S V R L L S G E H D I A L T V R -

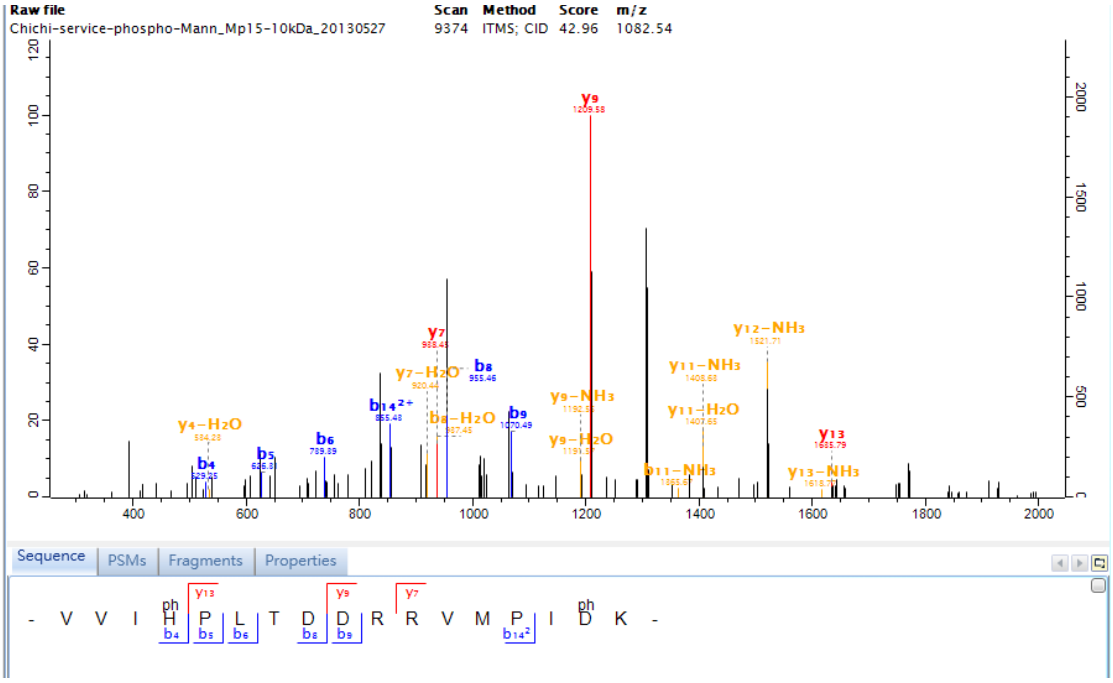
Raw file
Chichi-service-phospho-Mann_Mp15-10kDa_20130527
Scan Method Score m/z
9374 ITMS; CID 42.96 1082.54
y9
y12-NH3
y7
y7-H2O
b8
y11-NH3
b14 2+
y9-NH3
y11-H2O
b9
b8-H2O
y4-H2O
b6
y9-H2O
y13
b5
b4
b11-NH3
y13-NH3
Sequence
PSMs
Fragments
Properties
- V V I H P L T D D R R V M P I D K -
