# Supplementary material for: Site-specific His/Asp phosphoproteomic analysis of prokaryotes reveals putative targets for drug resistance
Source: BMC Microbiol. 2017 May 25;17:123. doi: 10.1186/s12866-017-1034-2 (PMC5445275; doi:10.1186/s12866-017-1034-2)
Supplement: Supplementary file 2 — Phospho probabilities, process method, leading protein identifier, UniProt No. (if available), protein description, and protein functional class of the identified phosphopeptides (PDF 539 kb). [file 12866_2017_1034_MOESM2_ESM.pdf]

Table S1. Identified phosphopeptides in the nine prokaryotes.

	Phospho (HDSTY) Probabilities	Process Method	Leading Protein	UniProt #	Protein Description	Functional Class
Ab01	D(1)FY(0.805)IT(0.165)H(0.024)ELIH(0.006)H(0.001)R	solution; HAMMOC	470.191.peg.2776		Probable transmembrane protein	Cellular processes
Ab02	DGSSVD(0.002)PAD(0.076)D(0.076)GT(0.23)RH(0.533)IRPED(0.045)GD(0.036)PIEIDE	solution; TiO_2	470.191.peg.1655		Uncharacterized protein	Unknown
Ab03	D(0.005)IH(0.996)ANCVRY(1)GT(1)K	solution; HAMMOC	470.191.peg.121		Coenzyme F420-dependent N5, N10-methylene tetrahydromethanopterin reductase	Metabolism (others, energy)
Ab04	D(0.091)IH(0.905)H(0.923)T(0.924)LS(0.092)H(0.042)EQT(0.023)QIQGR	solution; HAMMOC	470.191.peg.2774		Cys regulon transcriptional activator CysB	Signal transduction
Ab05	D(0.001)NH(0.936)S(0.863)VS(0.396)Y(0.805)GLK	solution; HAMMOC	470.191.peg.1581		Outer membrane protein E	Transport and binding proteins
Ab06	D(0.014)PS(0.874)H(0.874)VRNY(0.244)S(0.995)IK	solution; TiO_2	470.191.peg.3066		SAM-dependent methyltransferase YafE (UbiE paralog)	Cellular processes
Ab07	D(0.001)T(0.001)H(0.001)GMVLLD(0.997)NFEAVQR	solution; HAMMOC	470.191.peg.2189		Monoamine oxidase (1.4.3.4)	Metabolism (amino acid)
Ab08	D(0.271)T(0.246)H(0.778)Y(0.762)S(0.944)VRKIAK	solution; HAMMOC	470.191.peg.898		Histidine decarboxylase (EC 4.1.1.22)	Metabolism (amino acid)
Ab09	D(0.011)VH(0.986)RLVY(0.034)D(0.137)IMND(0.832)K	solution; HAMMOC	470.191.peg.2052		Twitching motility protein PilT	Transport and binding proteins
Ab10	EAQH(1)RFGH(1)VPDK	solution; HAMMOC	470.191.peg.2989		Protein RtcB	Metabolism (others)
Ab11	ELSPD(1)QMFD(1)H(1)VLK	solution; HAMMOC	470.191.peg.576		Long-chain-fatty-acid-CoA ligase (EC 6.2.1.3)	Metabolism (others)
Ab12	ES(0.006)LH(0.994)IPS(1)S(1)S(1)ERK	solution; HAMMOC	470.191.peg.2849		RNA polymerase sigma-54 factor RpoN	Transcription
Ab13	H(0.987)FCY(0.143)LPAS(0.736)S(0.09)KY(0.063)FS(0.981)K	solution; HAMMOC	470.191.peg.1743		Uncharacterized protein	Unknown
Ab14	HLKEH(1)PH(1)K	solution; HAMMOC	470.191.peg.2325		Acetyl-coenzyme A synthetase (EC 6.2.1.1)	Metabolism (others, energy)
Ab15	H(0.003)Y(0.013)FY(0.043)QILLD(0.953)MFED(0.988)K	solution; HAMMOC	470.191.peg.937		Inner membrane protein YfiN	Transport and binding proteins
Ab16	IAPWH(1)GR	solution; TiO_2	470.191.peg.289		Mobile element protein	Cellular processes (gene mobility)
Ab17	IS(0.988)GED(0.394)QPH(0.704)CH(0.91)LMFS(0.004)LK	solution; HAMMOC	470.191.peg.354		Plasmid mobilization protein	Cellular processes (gene mobility)
Ab18	KFNAAH(1)D(1)LELS(1)K	solution; HAMMOC	470.191.peg.2332		Phage tail length tape-measure protein 1	Cellular processes (gene mobility)
Ab19	LH(1)LKQS(1)PPS(1)K	solution; HAMMOC	470.191.peg.2656		Uncharacterized protein	Unknown

	Phospho (HDSTY) Probabilities	Process Method	Leading Protein	UniProt #	Protein Description	Functional Class
Ab20	LVD(0.983)D(0.989)H(0.024)GT(0.992)D(0.012)PLKK	solution; HAMMOC	470.191.peg.1568		putative DNA helicase	Cellular processes
Ab21	MID(0.77)H(0.17)VNMD(0.06)ALSK	solution; HAMMOC	470.191.peg.2398		Transcription factor AraC	Transcription
Ab22	MID(0.985)H(0.914)VNMD(0.101)ALSK	solution; TiO_2	470.191.peg.2398		Transcription factor AraC	Transcription
Ab23	MID(0.547)H(0.878)VNMD(0.601)ALS(0.974)K	solution; HAMMOC	470.191.peg.2398		Transcription factor AraC	Transcription
Ab24	MID(0.325)H(0.811)VNMD(0.863)ALS(1)K	solution; HAMMOC	470.191.peg.2398		Transcription factor AraC	Transcription
Ab25	MKD(0.901)H(0.091)EIMS(0.099)T(0.909)K	solution; HAMMOC	470.191.peg.1942		Taurine-binding periplasmic protein TauA	Transport and binding proteins
Ab26	MPT(0.081)H(0.919)QYRQSK	solution; HAMMOC	470.191.peg.315		Outer membrane protein	Transport and binding proteins
Ab27	MS(1)EIS(1)FERLH(1)QFFCK	solution; HAMMOC	470.191.peg.239		Uncharacterized protein	Unknown
Ab28	MS(0.23)LY(0.557)T(0.214)T(0.205)GH(0.794)PVVD(0.001)K	solution; HAMMOC	470.191.peg.2787		Uncharacterized protein	Unknown
Ab29	MVVS(1)T(1)H(1)PIY(1)LIAK	solution; HAMMOC	470.191.peg.750		Zinc ABC transporter, periplasmic-binding protein ZnuA	Transport and binding proteins
Ab30	ND(0.131)AEH(0.939)Y(0.93)AVWLGR	solution; HAMMOC	470.191.peg.1441		Sulfatase modifying factor 1 precursor (C-alpha-formyglycine- generating enzyme 1)	Cellular processes
Ab31	NLH(1)T(1)D(1)S(1)NALALK	solution; TiO_2	470.191.peg.2534		Sulfate transport system permease protein CysW	Transport and binding proteins
Ab32	QH(0.868)FS(0.833)LAT(0.298)GRCLEDK	solution; HAMMOC	470.191.peg.2110		Nitrite reductase [NAD(P)H] large subunit (EC 1.7.1.4)	Metabolism (others, energy)
Ab33	RNYLY(0.004)LAH(0.999)S(0.997)KLR	solution; TiO_2	470.191.peg.1641		Sugar transpoter	Transport and binding proteins
Ab34	S(0.115)EH(0.011)T(0.115)GPFMLMQD(0.759)NIGR	solution; HAMMOC	470.191.peg.1979		Uncharacterized protein	Unknown
Ab35	S(1)FIAET(1)VFS(1)H(1)PS(1)K	solution; HAMMOC	470.191.peg.1479		Uncharacterized protein	Unknown
Ab36	S(0.636)LY(0.291)T(0.607)T(0.607)GH(0.858)PVVDK	solution; HAMMOC	470.191.peg.2787		Uncharacterized protein	Unknown
Ab37	S(0.008)QLH(0.994)S(0.998)RVMQQAS(1)K	solution; HAMMOC	470.191.peg.2402		ABC1 family protein	Transport and binding proteins
Ab38	S(0.239)RD(0.823)VS(0.921)FKH(0.367)GS(0.65)GPR	solution; HAMMOC	470.191.peg.281		3-carboxymuconate cyclase	Cellular processes
Ab39	T(0.003)S(0.012)LAH(0.87)H(0.129)AT(0.987)ENR	solution; HAMMOC	470.191.peg.2451		Topoisomerase IV subunit A (EC 5.99.1.-)	Transcription

	Phospho (HDSTY) Probabilities	Process Method	Leading Protein	UniProt #	Protein Description	Functional Class
Ab40	VH(0.999)IVS(0.001)IEQDES(1)KD	solution; HAMMOC	470.191.peg.2447		CBS domain protein	Unknown
Ab41	VID(0.962)T(0.819)VAS(0.094)S(0.093)MRH(0.016)ND(0.007)D(0.005)Y(0.002) VITLEDGTK	solution; HAMMOC	470.191.peg.2272		Uncharacterized protein	Unknown
Ap01	D(0.99)EKRPLGIPT(0.018)IQVLH(0.783)H(0.208)R	solution; HAMMOC	gi|423062531	K1WR40	Uncharacterized protein	Unknown
Ap02	H(0.976)IPH(0.009)AKS(0.175)PGT(0.84)NVK	solution; HAMMOC	gi|423067554	K1VPX5	WD-40 repeat protein	Cellular processes
Ap03	H(1)QQGVVKR	solution; HAMMOC	gi|423062703	K1WFA9	Uncharacterized protein	Unknown
Ap04	LIPNS(1)H(1)LR	solution; HAMMOC	gi|423062151	K1W2U9	GTPase EngC	Metabolism (others, energy)
Ap05	LLKY(1)H(1)VVK	solution; HAMMOC	gi|423067034	K1W0W3	Phytoene desaturase	Metabolism (others)
Ap06	LPIAD(0.978)Y(0.976)PAPILRH(0.045)LQPYMEGR	solution; HAMMOC	gi|423067713	K1VZL1	Uncharacterized protein	Unknown
Ap07	MWKD(0.063)EIVEEIH(0.937)QIR	solution; HAMMOC	gi|423064385	K1W772	Uncharacterized protein	Unknown
Ap08	NLT(0.019)VVGGIH(0.982)VRLS(1)PD(1)R	solution; HAMMOC	gi|423062440	K1XG38	Phosphoglucomutase/phosphomannomutase	Metabolism (carbohydrate)
Ap09	QRTEGLADGIIVTPS(0.153)H(0.841)NPPS(0.005)D(0.001)GGFK	solution; HAMMOC	gi|423067440	K1W040	Phosphoglucomutase alpha-D-glucose phosphate-specific	Metabolism (carbohydrate)
Ap10	RS(0.001)LFRY(0.003)LIQQH(0.979)T(0.784)AT(0.151)AY(0.082)K	solution; HAMMOC	gi|423066653	K1X2U2	Alpha/beta hydrolase fold protein	Protein folding and modifications
Ap11	S(0.996)H(0.952)H(0.05)GGY(0.002)GVIQPR	solution; HAMMOC	gi|423062459	K1WSH3	Sigma 54 modulation protein/ribosomal protein S30EA	Metabolism (others)
Ap12	SRVH(1)S(1)MLRIK	solution; HAMMOC	gi|423063280	K1WPM3	Response regulator receiver sensor signal transduction histidine kinase	Signal transduction
Ap13	T(0.125)H(0.858)AT(0.009)D(0.006)ILT(0.001)LGR	solution; HAMMOC	gi|423064550	K1W7L7	Chromophore lyase CpcT/CpeT (EC 4.-.-.-)	Protein folding and modifications
Ap14	VH(0.849)ICT(0.151)IQGLVK	solution; HAMMOC	gi|423062780	K1XE41	Type III restriction protein res subunit	Cellular processes
Hp01	ACVD(0.117)LT(0.252)S(0.79)D(0.82)Y(0.604)MH(0.208)D(0.208)K	gel; HAMMOC		O24875	ATP-dependent C1p protease (clpA)	Protein folding and modifications
Hp02	DGLVRLS(0.006)VGIEH(0.007)EQD(0.007)LLED(0.979)LEQAFAK	gel; HAMMOC		P56069	cystathionine gamma-synthase (metB)	Cellular processes
Hp03	DVYQALFLH(0.958)KH(0.042)LK	gel; HAMMOC		A0A0B5FMF4.1	Uncharacterized protein	Unknown
Hp04	EQVVALQH(1)QR	gel; HAMMOC		I9TTL2	NAD(P)H-flavin oxidoreductaseoxidoreductase	Metabolism (others, energy)

	Phospho (HDSTY) Probabilities	Process Method	Leading Protein	UniProt #	Protein Description	Functional Class
Hp05	FS(0.001)GLIH(0.999)QIAK	gel; HAMMOC		O26094	riboflavin synthase alpha subunit (ribC)	Metabolism (others)
Hp06	GH(0.993)T(0.931)AY(0.076)LDEVR	gel; HAMMOC		O25047	Uncharacterized protein	Unknown
Hp07	GLVGH(1)APLKLQD(0.98)LQKAS(0.02)K	gel; HAMMOC		O25066	Para-aminobenzoate synthetase PabB	Metabolism (others)
Hp08	H(1)VLRIQKLD(0.589)ES(0.411)LK	gel; HAMMOC		O25298	galactosidase acetyltransferase (lacA)	Metabolism (others, energy)
Hp09	ILVS(0.121)LAVLS(0.202)H(0.492)S(0.069)AH(0.127)AVKT(0.992)H(0.998)NLER	gel; HAMMOC		O25395	iron(III) dicitrate transport protein (fecA)	Transport and binding proteins
Hp10	KIAH(0.003)LKAPS(0.108)S(0.264)H(0.626)IINGY(0.475)AH(0.762)S(0.762)NAR	gel; HAMMOC		P55975	translation elongation factor EF-Ts (tsf)	Translation
Hp11	KLH(1)QILVD(1)R	gel; HAMMOC		A0A0B5FYS2.1	Uncharacterized protein	Unknown
Hp12	LS(1)H(1)KFND(1)ER	gel; HAMMOC		V5NQK6	ubiquinol cytochrome c oxidoreductase	Metabolism (others, energy)
Hp13	MDNQKIT(0.999)H(1)QNIT(0.001)QK	gel; HAMMOC		A0A0B5FX52.1	Uncharacterized protein	Unknown
Hp14	MKLQGNMH(1)QPK	gel; HAMMOC		O25125	Uncharacterized protein	Unknown
Hp15	MLD(0.99)FIQELS(0.043)T(0.476)PH(0.476)VRD(0.016)FFLLFLR	gel; HAMMOC		A0A083YDP5	flagellar biosynthetic protein (fliR)	Cellular processes
Hp16	NKKFS(0.004)VLVY(0.998)H(0.998)K	gel; HAMMOC		P55986	RNA pseudouridine synthase	Metabolism (others)
Hp17	NKKMH(1)VNVQK	gel; HAMMOC		O25170	MscS family protein, Small-conductance mechanosensitive channel	Signal transduction (Stress)
Hp18	NKRIGAD(0.003)H(0.003)S(0.025)AQH(0.969)GMELGVK	gel; HAMMOC		O24870	Outer membrane protein Omp2	Cellular processes
Hp19	QEGLLVQAH(0.981)VS(0.24)MRY(0.778)K	gel; HAMMOC		O25664	Bifunctional enzyme IspD/IspF, bifunctional 2-C-methyl-D-erythritol 4-phosphate cytidylyltransferase	Cellular processes
Hp20	RAFIQAH(0.91)AKD(0.09)VK	gel; HAMMOC		P55992	DNA gyrase, sub B (gyrB)	Transcription
Hp21	S(0.001)FIPPQH(0.923)T(0.053)MLGIT(0.023)R	gel; HAMMOC		O25395	iron(III) dicitrate transport protein (fecA)	Transport and binding proteins
Hp22	S(1)GVGLT(1)PLFH(1)GGEH(1)MNGR	gel; HAMMOC		O25008	cysteine desulfurase (IscS)	Cellular processes
Hp23	VAH(1)KMGMNR	gel; HAMMOC		O26037	ATP-binding protein	Cellular processes

	Phospho (HDSTY) Probabilities	Process Method	Leading Protein	UniProt #	Protein Description	Functional Class
Hp24	VKINH(1)EH(1)AKLAS(1)VVK	gel; HAMMOC		P55986	RNA pseudouridine synthase	Metabolism (others)
Kp01	EMS(1)EKLQAH(1)AEK	solution; antibody	238896470	A6TDU9	Agmatinase	Metabolism (amino acid)
Kp02	GGRT(0.195)CH(0.805)AAIIAR	solution; antibody	238895214	A0A016R9E2.1	phosphoenolpyruvate synthase	Metabolism (others)
Kp03	HNLPHNSLNFVFH(0.813)GGS(0.014)GS(0.086)S(0.086)AQEIK	solution; antibody	238896464	A0A031SA15.1	fructose-bisphosphate aldolase	Metabolism (others)
Kp04	IFT(0.05)IS(0.055)S(0.967)AKT(0.966)FH(0.961)K	solution; antibody	238896590	A0A0E1C826	Uncharacterized protein	Unknown
Kp05	MDS(1)KIH(1)R	solution; antibody	238893972	A0A059YV87.1	Uncharacterized protein	Unknown
Kp06	MLFQH(0.784)FS(0.216)LR	solution; antibody	238895506	A4GZC4	MATE family transport protein YeeO	Transport and binding proteins
Kp07	VH(0.81)H(0.151)AY(0.005)D(0.016)S(0.016)D(0.001)R	solution; antibody	238896752	A0A016R423.1	ribonuclease G	Cellular processes
Mm01	ALNH(1)AEQFS(0.409)RIS(0.591)GVK	gel; TiO_2	21228639	Q8PU20	DNA-directed RNA polymerase subunit F	Transcription
Mm02	AS(0.014)GECCH(0.492)S(0.492)GT(0.215)ENPD(0.784)MQH(0.003)GK	solution; TiO_2	21226269	Q8Q0G9	Cobalt-zinc-cadmium resistance protein	Cellular processes
Mm03	D(0.052)IS(0.948)S(0.953)LVD(0.802)IH(0.197)Y(0.048)IRQK	gel; TiO_2	21227235	Q8PXT3	UTP--glucose-1-phosphate uridylyltransferase	Metabolism (carbohydrate)
Mm04	D(0.005)KGPH(0.817)Y(0.178)LIKAAPS(1)ILK	gel; TiO_2	21228207	Q8PV69	putative glycosyltransferase	Metabolism (carbohydrate)
Mm05	ENGH(1)IY(1)R	solution; TiO_2	21226271	Q8Q0G7	Sensory transduction histidine kinase	signal transduction
Mm06	GIPID(1)PH(1)PVHKK	gel; TiO_2	21228521	Q8PUB6	DNA gyrase, subunit B	Transcription
Mm07	GKHVVVAPLH(1)S(1)R	gel; TiO_2	21227211	Q8PXV7	Conserved protein	Unknown
Mm08	GRS(0.571)D(0.57)D(0.89)MNS(0.957)IEGLRH(0.012)R	solution; HAMMOC	161485677	M1Q384	Uncharacterized protein	Unknown
Mm09	H(0.999)AMIT(0.001)KSQKELIEYLK	gel; TiO_2	21228860	Q8PTF7	Uncharacterized protein	Unknown
Mm10	H(0.121)ND(0.095)VY(0.146)T(0.531)IAT(0.317)FAD(0.795)D(0.873)VD(0.076)Y (0.068)T(0.98)K	solution; HAMMOC	21228770	Q8PTP5	Cell division protein DivIC (FtsB)	Cellular processes
Mm11	H(0.353)ND(0.353)VY(0.235)T(0.352)IAT(0.423)FAD(0.292)D(0.974)VD(0.537)Y (0.482)T(1)K	solution; HAMMOC	21228770	Q8PTP5	Cell division protein DivIC (FtsB)	Cellular processes
Mm12	IH(0.096)MGH(0.041)VLT(0.894)VNKLID(0.968)LQK	gel; TiO_2	74550296	Q8PVK0	Tyrosine--tRNA ligase	Translation
Mm13	IMH(0.998)GEY(0.971)VKY(0.03)GR	gel; TiO_2	21229356	Q8PTU7	Uncharacterized protein	Unknown
Mm14	KH(0.773)S(0.222)S(0.005)GNKISWAR	gel; TiO_2	21228716	Q8PUV5	Dolichyl-phosphate glucose synthetase	Metabolism (carbohydrate)

	Phospho (HDSTY) Probabilities	Process Method	Leading Protein	UniProt #	Protein Description	Functional Class
Mm15	KLLS(0.025)Y(0.097)H(0.889)S(0.989)LEK	gel; TiO_2	21228325	Q8PZB5	Transposase	Cellular processes
Mm16	LGKMRY(0.107)H(0.845)IH(0.047)QK	gel; TiO_2	21226681	Q8PXU9	Polysaccharide deacetylase	Cellular processes
Mm17	LVEQRIRH(1)ILS(1)K	gel; TiO_2	21227219	Q8PUQ9	DNA-directed RNA polymerase beta chain	Transcription
Mm18	MQH(0.059)H(0.168)ID(0.168)S(0.608)FNKFID(0.999)Y(0.999)GLQK	solution; HAMMOC	21228375	Q8PT38	Response regulator	signal transduction
Mm19	MT(1)EQS(1)AH(1)EKY(1)EFK	solution; HAMMOC	23822025	Q8PX75	Translation termination factor aRF1	Translation
Mm20	MT(1)KFS(1)LLD(1)H(1)EAVPK	solution; HAMMOC	23822082	Q8PUQ8	DNA-directed RNA polymerase subunit H	Transcription
Mm21	QT(0.001)H(0.999)VVNVY(1)PQ	solution; TiO_2	21226269	Q8PSX8	glycosyltransferase	Metabolism (carbohydrate)
Mm22	S(0.687)MLD(0.788)S(0.529)CIGGH(0.997)PVL	solution; TiO_2	21228982	Q8PZX8	transcriptional regulator, MarR family	Transcription
Mm23	TGIPVLGVLPH(1)FK	gel; TiO_2	34395563	Q8Q0P3	Probable cobyric acid synthase	Cellular processes
Mp01	D(0.134)AD(0.866)H(0.02)LMKY(0.439)LT(0.542)R	gel; TiO_2	294496380	D5E7U0	Translation initiation factor 2 subunit beta	Translation
Mp02	D(0.014)LH(0.986)WKKD(1)MR	solution; HAMMOC	294496535	D5E895	Deoxyhypusine synthase	Cellular processes
Mp03	D(1)VT(1)VH(1)IEPK	gel; TiO_2	294495652	D5EBE9	Cation diffusion facilitator family transporter	Transport and binding proteins
Mp04	EIT(0.185)H(0.82)FFET(0.918)Y(0.076)KNLENK	gel; TiO_2	294495989	D5E6P9	Inorganic pyrophosphatase	Cellular processes
Mp05	ELGY(0.913)ET(0.053)D(0.053)IH(0.98)GNK	gel; TiO_2	294495295	D5EAE2	DNA polymerase II large subunit	Transcription
Mp06	EQY(0.004)RKAAQFFS(0.998)H(0.998)LK	gel; TiO_2	294495986	D5E6P6	glycosyltransferase involved in cell wall biogenesis	Cellular processes
Mp07	FH(0.858)IQT(0.13)RKY(0.011)D(0.003)D(0.998)FVR	gel; TiO_2	294496075	D5E6Y5	FAD-dependent pyridine nucleotide-disulfide oxidoreductase	Metabolism (others, energy)
Mp08	IVH(0.879)VT(0.754)D(0.436)EAT(0.093)Y(0.093)T(0.744)IK	gel; TiO_2	294494753	D5E8U2	Uncharacterized protein	Unknown
Mp09	MFH(1)RRLK	solution; HAMMOC	294495553	D5EB50	phosphate ABC transporter, PhoT	Transport and binding proteins
Mp10	MRPVLH(0.993)T(0.995)T(0.012)VGQR	solution; HAMMOC	294495112	D5E9V9	Uncharacterized protein	Unknown
Mp11	MT(0.003)T(0.017)T(0.989)D(0.988)T(0.973)ILEKLH(0.03)ENAK	gel; TiO_2	294495136	D5E9Y3	Uncharacterized protein	Unknown
Mp12	QLVIGNT(0.639)T(0.432)ET(0.949)IH(0.98)K	gel; TiO_2	294495798	D5EBU5	methyltransferase	Cellular processes
Mp13	S(0.004)AENLLIH(0.996)LS(1)R	gel; TiO_2	294495831	D5EBX8	Cobaltochelatase CobN subunit	Cellular processes
Mp14	S(0.275)VRLLS(0.747)GEH(0.988)D(0.99)IALT(1)VR	gel; TiO_2	294494975	D5E9H2	transcriptional regulator, AsnC family	Transcription
Mp15	VVIH(0.951)PLT(0.038)D(0.011)D(0.007)RRVMPID(0.993)K	gel; TiO_2	294495109	D5E9V6	CbbQ/NirQ/NorQ domain protein	Cellular processes

	Phospho (HDSTY) Probabilities	Process Method	Leading Protein	UniProt #	Protein Description	Functional Class
Mt01	D(1)ALH(1)ERAIMLR	solution; HAMMOC	Mtai_v1c02000		glycerol-3-phosphate dehydrogenase	Metabolism (carbohydrate)
Mt02	D(1)LPGVRY(1)H(1)IVR	gel; HAMMOC	Mtai_v1c05130		ribosomal protein S12	Translation
Mt03	EH(1)LLIWD(1)KRR	solution; HAMMOC	Mtai_v1c19090		Uncharacterized protein	Unknown
Mt04	FQH(0.044)APD(0.964)AS(0.994)FD(0.999)K	solution; HAMMOC	Mtai_v1c14590		peptidase M29 aminopeptidase II	Protein folding and modifications
Mt05	GPD(0.999)H(0.999)QAVVELPQPT(0.002)GQLVLGHNR	solution; HAMMOC	Mtai_v1c25070		asparagine synthase (glutamine-hydrolyzing)	Metabolism (amino acid)
Mt06	H(1)GIVFID(0.263)D(0.071)EVQT(0.666)GIGR	solution; HAMMOC	Mtai_v1c01870		4-aminobutyrate aminotransferase	Cellular processes
Mt07	H(0.998)H(0.998)LERGS(0.937)LVID(0.066)PLTLGELHK	solution; HAMMOC	Mtai_v1c15250		Uncharacterized protein	Unknown
Mt08	H(0.56)S(0.56)T(0.881)ALLVH(0.764)GEPGIGKT(0.235)R	gel; HAMMOC	Mtai_v1c03890		transcriptional activator domain-containing protein	Transcription
Mt09	IT(0.949)Y(0.051)AARIH(0.074)D(0.925)IGK	solution; HAMMOC	Mtai_v1c04100		metal dependent phosphohydrolase	Cellular processes
Mt10	KS(0.023)LH(0.977)LGER	solution; HAMMOC	Mtai_v1c12930		Uncharacterized protein	Unknown
Mt11	KVH(1)AKALY(1)VR	solution; HAMMOC	Mtai_v1c17220		Polyphosphate kinase	signal transduction
Mt12	LS(1)H(1)PGVVQVFD(1)IGEEEGR	solution; HAMMOC	Mtai_v1c21170		serine/threonine protein kinase	signal transduction
Mt13	MNFH(1)S(1)VLR	solution; HAMMOC	Mtai_v1c19840		Uncharacterized protein	Unknown
Mt14	MPH(0.115)T(0.136)AT(0.88)ID(0.869)RNK	gel; HAMMOC	Mtai_v1c01190		Aminotransferase class-III	Cellular processes
Mt15	PGS(0.001)VT(0.035)PH(0.802)T(0.677)KFEAS(0.539)VY(0.946)VLK	gel; HAMMOC	Mtai_v1c05160		translation elongation factor Tu	Translation
Mt16	PT(1)LGVVS(1)RFAH(1)QK	gel; HAMMOC	Mtai_v1c25160		glycogen/starch synthase, ADP-glucose type	Metabolism (carbohydrate)
Mt17	QY(0.001)S(0.004)GFD(0.976)ARVT(0.018)VLGH(0.001)IQR	solution; HAMMOC	Mtai_v1c03330		6-phosphofructokinase	Metabolism (carbohydrate)
Mt18	RAH(0.997)LT(0.003)PR	gel; HAMMOC	Mtai_v1c13860		putative peroxidase-related enzyme	signal transduction
Mt19	VIH(1)QIGAK	gel; HAMMOC	Mtai_v1c10130		Uncharacterized protein	Unknown
Mt20	VLEAH(1)PAR	solution; HAMMOC	Mtai_v1c13250		(Uracil-5)-methyltransferas	Translation

	Phospho (HDSTY) Probabilities	Process Method	Leading Protein	UniProt #	Protein Description	Functional Class
Mt21	Y(0.001)Y(0.001)S(0.504)T(0.504)RD(0.977)PH(0.013)K	gel; HAMMOC	Mtai_v1c20190		threonine synthase	Metabolism (amino acid)
Tt01	AAGGAMLT(0.025)AS(0.025)H(0.949)NPPQYLGVK	solution; TiO_2	46198599	Q5SKJ3	phosphoglucomutase/phosphomannomutase	Metabolism (carbohydrate)
Tt02	ADGVLLT(0.042)PS(0.051)H(0.895)NPPED(0.011)GGFK	solution; TiO_2	46199932	Q72H65	phosphoglucomutase	Metabolism (carbohydrate)
Tt03	ADGVLLT(0.003)PS(0.19)H(0.77)NPPED(0.037)GGFKYNPPTGGPANAR	solution; TiO_2	46199932	Q72H65	phosphoglucomutase	Metabolism (carbohydrate)
Tt04	D(0.996)H(0.996)VAGMD(0.97)VLIT(0.799)T(0.238)AQVPGR	solution; TiO_2	46200082	Q72GR8	NAD/NADP transhydrogenase alpha subunit 1	Metabolism (others, energy)
Tt05	H(0.801)LT(0.224)RT(0.332)GY(0.289)T(0.306)LGT(0.046)PT(0.001)Y(0.001)MA PEQAK	solution; TiO_2	46200158	Q72GJ2	serine/threonine protein kinase	signal transduction
Tt06	ISQELAERHYAEH(1)R	solution; TiO_2	46200100	Q72GQ0	nucleoside diphosphate kinase	Cellular processes
Tt07	LAEESALGLLTPVYPGLAPGS(0.054)GPGH(0.943)LALFGY(0.002)D(0.001)PFR	solution; TiO_2	46200190	Q72GG0	3-phosphonopyruvate decarboxylase	Metabolism (carbohydrate)
Tt08	MRH(1)LKS(1)GR	solution; TiO_2	46199601	Q72I33	50S ribosomal protein L17	Translation
Tt09	PEVVRAQMNLLT(0.315)S(0.328)H(0.856)D(0.575)T(0.927)PR	solution; TiO_2	46199500	Q72ID4	putative pullulanase	Metabolism (carbohydrate)
Tt10	RILFD(1)H(1)LMEALR	solution; TiO_2	46199168	Q5SIY7	tryptophanyl-tRNA synthetase	Translation
Tt11	T(0.017)KT(0.053)LEEFGH(0.929)LAALLDGSR	solution; TiO_2	46199331	Q72IV2	Uncharacterized protein	Unknown
Vv01	CLNLGMND(1)H(1)IS(1)K	solution; HAMMOC	37676244	Q7MET6	Uncharacterized protein	Unknown
Vv02	D(0.002)IQD(0.866)MH(0.132)FMLQK	solution; HAMMOC	37678662	Q7MP86	dimethyladenosine transferase	Transcription
Vv03	GLS(0.035)LS(0.076)GGD(0.945)PLH(0.945)PANVAD(0.999)ILRLVK	gel; TiO_2	161486681	Q8D731.1	anaerobic ribonucleoside-triphosphate reductase activating protein	Cellular processes
Vv04	GRLS(1)LS(1)H(1)LK	solution; HAMMOC	37676069	Q7MFB1	flavodoxin reductase	Cellular processes
Vv05	H(1)FRLRGS(1)S(1)MS(1)IK	solution; HAMMOC	37676262	Q7MER8	pilus assembly protein TadG	Cellular processes
Vv06	H(0.99)QLD(0.01)KS(0.983)QFKD(0.017)Q	gel; TiO_2	37677310	Q7MBT7	putative two-component response regulator	Signal transduction
Vv07	H(0.173)VIT(0.985)H(0.885)T(0.969)VLT(0.969)EFFNT(0.012)T(0.008)K	solution; HAMMOC	37676899	Q7MCZ7	transcriptional regulator	Transcription
Vv08	H(0.912)Y(0.409)LVPD(0.507)FD(0.172)KVWDR	gel; TiO_2	37680761	Q7MIE0	sodium-dependent transporter	Transport and binding proteins

	Phospho (HDSTY) Probabilities	Process Method	Leading Protein	UniProt #	Protein Description	Functional Class
Vv09	IMVVCGH(0.99)GLGT(0.413)S(0.602)LMMEMS(0.995)IK	solution; HAMMOC	37676992	Q7MCQ4	Putative sugar phosphotransferase component II B	Metabolism (carbohydrate)
Vv10	IS(0.005)H(0.003)S(0.003)S(0.01)GKMKLGAS(0.99)S(0.99)D(0.999)	solution; HAMMOC	37679260	Q7M7K1	3-hydroxyacyl-ACP dehydratase;putative (3R)-hydroxymyristoyl-(acyl carrier protein) dehydratase	Cellular processes
Vv11	KKELPT(0.984)H(0.984)D(0.041)S(0.991)K	gel; TiO_2	37676244	Q7MET6	Uncharacterized protein	Unknown
Vv12	LDKKH(0.186)IED(0.796)ND(0.257)VS(0.991)AY(0.77)	solution; HAMMOC	37680466	Q7MJ80	cytochrome d ubiquinol oxidase, subunit I	Cellular processes
Vv13	LLNRH(1)AR	gel; TiO_2	37680602	Q7MIU5	transcriptional regulator	Transcription
Vv14	LPH(0.832)S(0.167)T(0.007)LEIS(0.994)K	gel; TiO_2	37678856	Q7MNP3	Oxidoreductase Tas, aldo/keto reductase family	Cellular processes
Vv15	LS(1)LH(1)WFLK	solution; HAMMOC	37679836	Q7MKX5	NAD-specific glutamate dehydrogenase	Metabolism (others, energy)
Vv16	MD(0.123)T(0.93)T(0.667)LS(0.762)H(0.548)AD(0.941)H(0.916)S(0.112)K	solution; HAMMOC	37676781	Q7MDB5	Branched-chain amino acid permease, ABC transporter AzlC	Transport and binding proteins
Vv17	MD(0.153)T(0.942)T(0.756)LS(0.767)H(0.406)AD(0.839)H(0.656)S(0.481)K	solution; HAMMOC	37676781	Q7MDB5	Branched-chain amino acid permease, ABC transporter AzlC	Transport and binding proteins
Vv18	MD(0.96)T(0.35)T(0.339)LS(0.047)H(0.003)AD(0.005)H(0.005)S(0.296)KT(0.993)RR	solution; HAMMOC	37676781	Q7MDB5	Branched-chain amino acid permease, ABC transporter AzlC	Transport and binding proteins
Vv19	MH(0.008)CPFCS(0.449)END(0.799)T(0.744)K	solution; HAMMOC	161486648	Q7MN57	ribonucleotide reductase transcriptional regulator NrdR	Transcription
Vv20	MSNPITKLLH(0.016)LKH(0.984)ET(1)QR	gel; TiO_2	37675973	Q7MFK7	RelB protein	Transcription
Vv21	MT(0.989)T(0.017)H(0.01)CCQMH(0.984)MNK	solution; HAMMOC	37680945	Q7MHV6	polyA polymerase	Transcription
Vv22	NLS(1)H(1)QVEEQFKS(1)FS(1)K	solution; HAMMOC	37676737	Q7MDF9	methyl-accepting chemotaxis protein	Signal transduction
Vv23	NRGT(1)S(1)VH(0.999)FWPD(0.001)T(0.001)K	solution; HAMMOC	37678772	Q7MNX7	DNA topoisomerase IV, B subunit	Transcription
Vv24	PIQAT(1)S(1)H(1)FMT(1)KVS(0.001)KS(0.999)K	solution; HAMMOC	37680273	Q7MJS1	methyl-accepting chemotaxis protein	Signal transduction
Vv25	PLLT(1)ILNVD(0.916)VT(0.917)H(0.169)LRS(0.998)K	solution; HAMMOC	37678899	Q7MNK1	Chaperone protein ClpB	Protein folding and modifications
Vv26	PMMY(0.214)T(0.98)H(0.852)Y(0.954)AT(1)K	solution; HAMMOC	37679283	Q7MMH2	transglutaminase-like enzyme	Metabolism (carbohydrate)
Vv27	PNQH(0.005)QY(0.023)FGH(0.972)PR	solution; HAMMOC	37679189	Q7MMR2	putative DeoR family transcriptional regulator	Transcription
Vv28	PT(0.001)RFVQD(0.984)NH(0.627)S(0.627)KS(0.76)K	gel; TiO_2	37678487	Q7MPQ9	dTDP-6-deoxy-D-xylo-4-hexulose-3,5-epimerase	Cellular processes
Vv29	QD(0.186)H(0.814)EALAS(1)FIVR	gel; TiO_2	37677207	Q7MC40	Uncharacterized protein	Unknown
Vv30	QH(0.77)AVFT(0.213)PED(0.017)VEVLSKEPLFQGFFK	gel; TiO_2	37678768	Q7MNY1	MutT/nudix family protein	Cellular processes

	Phospho (HDSTY) Probabilities	Process Method	Leading Protein	UniProt #	Protein Description	Functional Class
Vv31	S(0.071)FH(0.926)LGANS(0.497)NS(0.488)IS(0.017)LQLKNMR	gel; TiO_2	37679159	Q7MMU2	polar flagellin FlaE	Cellular processes
Vv32	S(1)FLPH(0.993)QD(0.001)EEKRS(0.006)R	solution; HAMMOC	37680808	Q7MI93	glycerol kinase	Metabolism (carbohydrate)
Vv33	S(0.024)RIVAT(0.488)S(0.488)VVGEVNH(1)VIGR	gel; TiO_2	37675735	Q7MG94	HTH-type transcriptional regulator MalT	Transcription
Vv34	T(1)FNEH(1)QLS(1)D(1)IVAK	solution; HAMMOC	37681059	Q7MHJ4	pyrroline-5-carboxylate reductase	Metabolism (carbohydrate)
Vv35	T(0.983)IKD(0.981)AEVH(0.019)Y(0.017)IAFNS(0.454)T(0.454)FLT(0.092)PKVAR	gel; TiO_2	37681062	Q7MHJ1	Uncharacterized protein	Unknown
Vv36	TIVQLGH(0.016)NMD(0.984)KK	gel; TiO_2	37676790	Q7MDA6	Uncharacterized protein	Unknown
Vv37	T(1)MQNH(1)D(1)D(1)QKRK	solution; HAMMOC	37676201	Q7MEX9	Uncharacterized protein	Unknown
Vv38	TQVH(0.992)GNS(0.966)LED(0.919)FH(0.122)R	solution; HAMMOC	37679296	Q7M7K0	Deoxyguanosinetriphosphate triphosphohydrolase	Metabolism (others, energy)
Vv39	VH(1)S(1)QVD(1)IT(1)LS(1)K	solution; HAMMOC	37680857	Q7MI44	Uncharacterized protein	Unknown
Vv40	VT(1)FVS(1)ND(1)Y(1)H(1)LK	solution; HAMMOC	37676157	Q7MF23	Uncharacterized protein	Unknown
