# Supplementary material for: Site-specific His/Asp phosphoproteomic analysis of prokaryotes reveals putative targets for drug resistance
Source: BMC Microbiol. 2017 May 25;17:123. doi: 10.1186/s12866-017-1034-2 (PMC5445275; doi:10.1186/s12866-017-1034-2)
Supplement: Supplementary file 3 — The relative abundances of amino acids between −10 and +10 positions of the phosphorylated or non-phosphorylated His/Asp (PDF 377 kb). [file 12866_2017_1034_MOESM3_ESM.pdf]

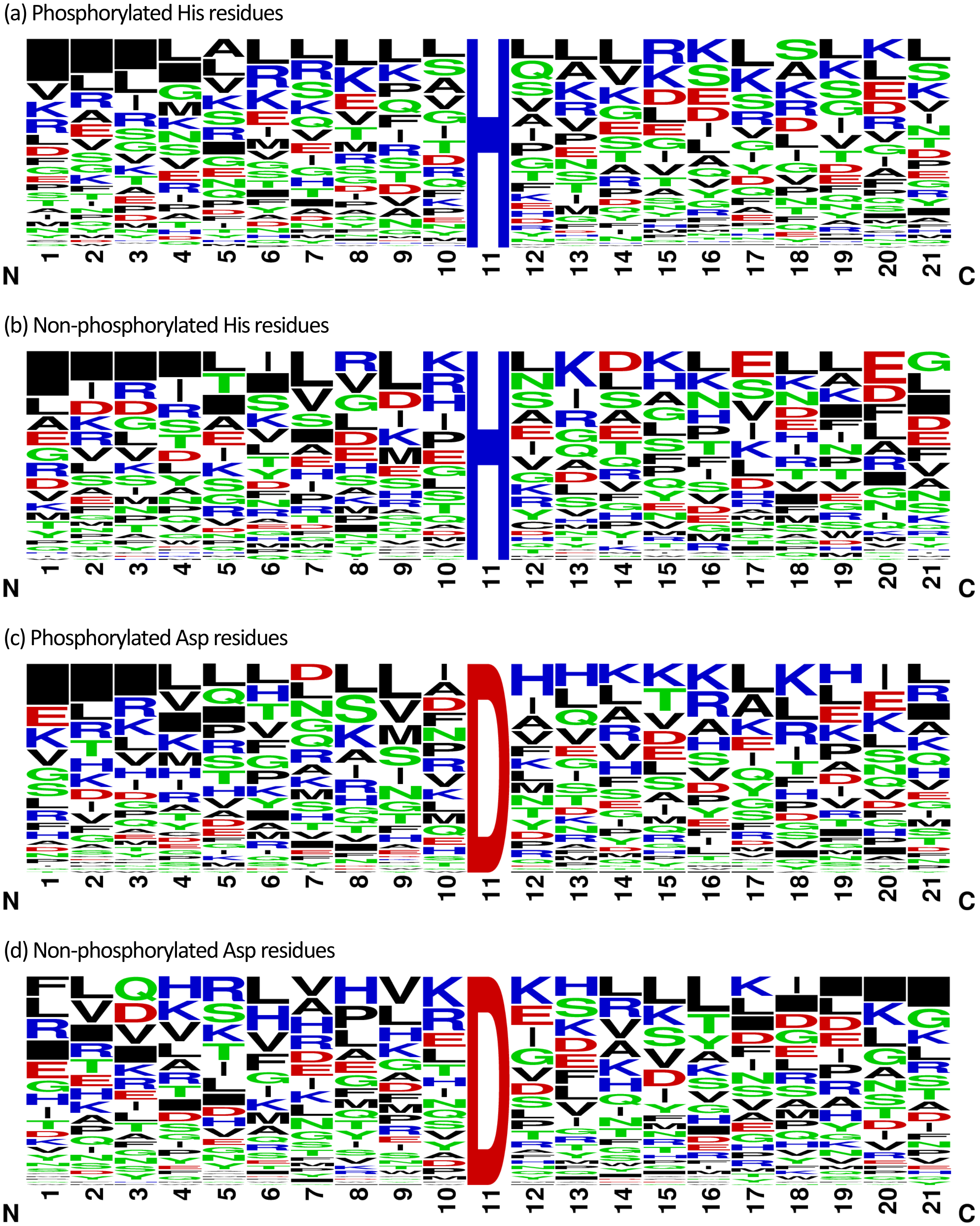

Figure S1. Relative abundances of amino acids between -10 and +10 positions of the phosphorylated or non-phosphorylated His/Asp.
