# Supplementary material for: Site-specific His/Asp phosphoproteomic analysis of prokaryotes reveals putative targets for drug resistance
Source: BMC Microbiol. 2017 May 25;17:123. doi: 10.1186/s12866-017-1034-2 (PMC5445275; doi:10.1186/s12866-017-1034-2)
Supplement: Supplementary file 4 — Homology model of H. pylori FecA (PDF 359 kb). [file 12866_2017_1034_MOESM4_ESM.pdf]

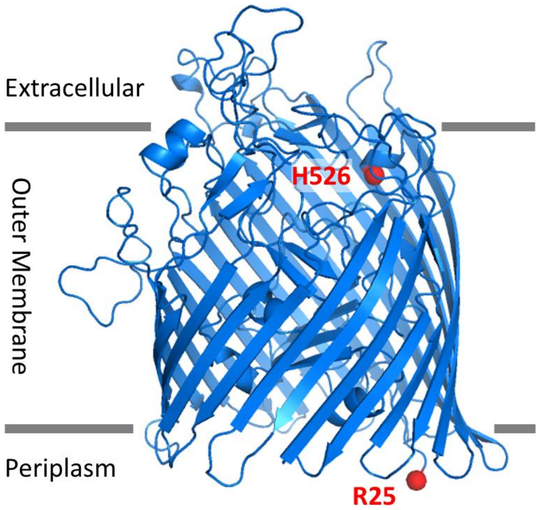

Figure S2. Homology model of *H. pylori* FecA based on 1PNZ generated by SWISS-MODEL (https://swissmodel.expasy.org/).
